# 1,3-Dibromo-5,5-dimethylhydantoin as promoter for glycosylations using thioglycosides

**DOI:** 10.3762/bjoc.13.195

**Published:** 2017-09-22

**Authors:** Fei-Fei Xu, Claney L Pereira, Peter H Seeberger

**Affiliations:** 1 Department of Biomolecular Systems, Max Planck Institute of Colloids and Interfaces, Am Mühlenberg 1, 14476 Potsdam, Germany; 2 Department of Chemistry and Biochemistry, Freie Universität Berlin, Arnimallee 22, 14195 Berlin, Germany; 3 Vaxxilon Deutschland GmbH, Magnusstraße 11, 12489 Berlin, Germany

**Keywords:** automated glycan assembly, 1,3-dibromo-5,5-dimethylhydantoin, glycosylation, promoter, thioglycosides

## Abstract

1,3-Dibromo-5,5-dimethylhydantoin (DBDMH), an inexpensive, non-toxic and stable reagent, is a competent activator of thioglycosides for glycosidic bond formation. Excellent yields were obtained when triflic acid (TfOH) or trimethylsilyl trifluoromethanesulfonate (TMSOTf) were employed as co-promoters in solution or automated glycan assembly on solid phase.

## Introduction

Thioglycosides are versatile glycosylating agents that are commonly used in oligosaccharide synthesis due to their accessibility, stability, compatibility with various reaction conditions, and orthogonality to other donors [1–5]. Different electrophilic/thiophilic reagents have been developed as promoters to activate thioglycoside donors [3,6–18]. However, most of those activators are expensive and toxic [5,17,19]. Poor solubility complicates the use of some promoters during automated glycan assembly [20–23], while the instability of some activators in solution requires them to be freshly prepared prior to use [24–26]. Here, we describe a promoter system based on the commercially available, inexpensive 1,3-dibromo-5,5-dimethylhydantoin (DBDMH) for the activation of thioglycosides.

DBDMH, a white to pale-brown powder that is readily soluble in most organic solvents, including dichloromethane, is sold under the trade name Brom-55 and used as swimming pool sanitizer, as industrial brominating agent for ethylene propylene diene monomer rubber to improve ozone resistance, as additive in plastics to promote photodegradation and as a fungicide to preserve fresh fruits [27]. In synthetic chemistry, DBDMH acts as a thiophilic activator in the conversion of dithioacetals to the corresponding *O,O*-acetals [28–30], as well as in the synthesis of heparin mimetics [31]. We considered DBDMH as a readily available alternative promoter for glycosylations involving thioglycosides.

## Results and Discussion

Initially, the capability of DBDMH to activate thioglycoside **1** [32] in order to glycosylate the primary hydroxy group present in D-glucose acceptor **2** [33] was explored without any additives (Table 1, entry 1). This initial experiment furnished disaccharide **3**, albeit in modest yield (43%). When TfOH or TMSOTf (10 mol %) were added as co-promoter, the yield increased to more than 90% (Table 1, entries 2 and 3). Next, the amount of the reagent required for activation was studied (Table 1, entries 3–5). Substoichiometric amounts of DBDMH (0.7 equiv) in the presence of co-promoter suffice to produce the disaccharide efficiently. The DBDMH/TfOH activation system is temperature insensitive as it furnishes the product from −78 °C to room temperature, although most disaccharide **3** is formed at −40 °C (Table 1, entries 3 and 6–9).

**Table 1:** Optimization of glycosylation conditions using DBDMH as promoter.

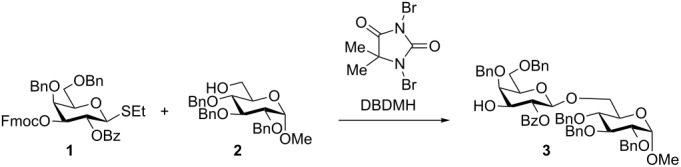				

Entry^a^	DBDMH (equiv^b^)	Co-promoter (10 mol %^b^)	*T* (°C)	Yield^c^ (%)

1	0.7	–	−40	43
2	0.7	TMSOTf	−40	93
3	0.7	TfOH	−40	92
4	0.5	TfOH	−40	85
5	1.0	TfOH	−40	94
6	0.7	TfOH	−78	83
7	0.7	TfOH	−20	87
8	0.7	TfOH	0	88
9	0.7	TfOH	rt	79

^a^Reaction conditions: donor (51 µmol), acceptor (43 µmol), dichloromethane; quenched with triethylamine. Fmoc protecting group was removed during the quenching process in the presence of triethylamine. ^b^Equivalents calculated relative to the amount of donor. ^c^Only isolated yields are reported.

Next, the scope of the new activation system was investigated by using a variety of glycosyl donors **4**–**10** [34–38] containing C-2 participating groups to ensure 1,2-*trans-*glycoside formation (Table 2). Each glycosylating agent was reacted with D-glucose acceptors **2** (Table 2, entries 1–8) and **11** [39] (Table 2, entries 9–16) with a free hydroxy group at C-6 and C-4 position, respectively. The DBDMH/TfOH system activates glycosyl donors including neutral monosaccharides of different configurations (D-gluco **5** and **6**, D-galacto **1** and **4**, D-manno **8**, L-rhamno **9**), amino sugar **7** and uronic acid **10**. All thioglycosides reacted equally well, irrespective of their aglycons (SEt or STol). This promoter is compatible with most commonly used protecting groups, except some electron-rich groups like 4-methoxybenzyl ethers that may be partly brominated under these conditions [40].

**Table 2:** 1,2-*Trans*-glycosylation activated by DBDMH with a variety of building blocks.

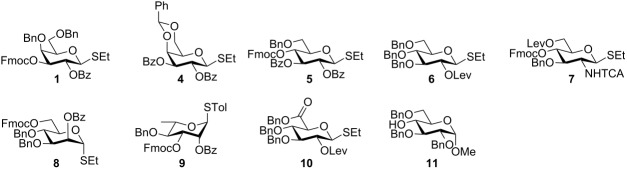							

Entry^a^	Donor	Acceptor	Yield^b^ (%)	Entry^a^	Donor	Acceptor	Yield^b^ (%)

1	**1**	**2**	92	9	**1**	**11**	88
2	**4**	**2**	95	10	**4**	**11**	88
3	**5**	**2**	98	11	**5**	**11**	87
4	**6**	**2**	94	12	**6**	**11**	89
5	**7**	**2**	91	13	**7**	**11**	60
6	**8**	**2**	96	14	**8**	**11**	89
7	**9**	**2**	91	15	**9**	**11**	86
8	**10**	**2**	39	16	**10**	**11**	45

^a^All reactions were carried out at −40 °C in dichloromethane with 0.7 equiv DBDMH and 10 mol % TfOH as promoter. ^b^Only isolated yields are reported.

To probe the scope of DBDMH/TfOH-mediated 1,2-*cis*-glycosylation, perbenzylated galactosyl donor **12** [41] (Table 3, entries 1–4) and galactosyl donor **13** [42] (Table 3, entries 5 and 6) as well as glucosyl donor **14** (Table 3, entries 7 and 8) were reacted with acceptor **2** in the presence of DBDMH. Electron-rich (‘armed’) thioglycosides [43] are more readily activated as the reaction of perbenzylated donor **12** in dichloromethane at −78 °C afforded the disaccharide with excellent yield but low stereoselectivity. The α/β ratio, determined by supercritical fluid chromatography (SFC), shifted significantly toward the α-isomer with ether [44] and toward the β-isomer when acetonitrile [45] was used as co-solvent. With all these donors, the α-stereoselectivity increased at higher temperature [46]. Donor **13**, containing a remote participating group, produced the disaccharide with better α-selectivity [22,42].

**Table 3:** 1,2-*Cis*-glycosylation activated by DBDMH.

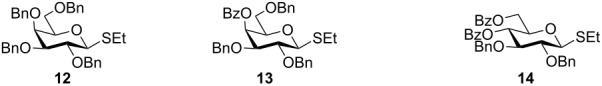						

Entry^a^	Donor	Acceptor	Solvent	*T* (°C)	Yield^b^(%)	α/β ratio^c^

1	**12**	**2**	DCM/Et_2_O^d^	−78	94	1:1.4
2	**12**	**2**	DCM	−78	94	1:2.7
3	**12**	**2**	DCM/MeCN^d^	−78	93	1:11.7
4	**12**	**2**	DCM	−40	67	1:1.3
5	**13**	**2**	DCM	−78	72	4.6:1
6	**13**	**2**	DCM	−40	50	11.8:1
7	**14**	**2**	DCM	−78	76	1:1.1
8	**14**	**2**	DCM	−40	69	1:1

^a^All reactions were carried out with 0.7 equiv DBDMH and 10 mol % TfOH as promoter. ^b^Only isolated yields are reported. ^c^Silica-2EP analytical column was used to determine the α/β ratio when using SFC. Isopropanol was used as co-solvent for the mobile phase. ^d^The ratio of solvents is 2:1 (v/v).

**Scheme 1:**
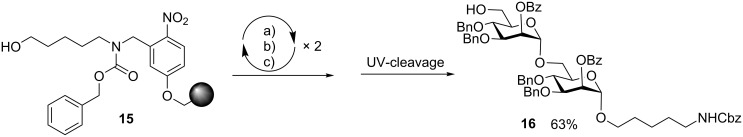
DBDMH as promotor for automated glycan assembly. Modules: a) acidic wash; b) glycosylation using DBDMH/TMSOTf, **8**; c) Fmoc deprotection.

Automated glycan assembly is the most rapid means to access complex oligosaccharides [20,47]. Ideally, stable and non-toxic reagents should be used on such instruments. The automated synthesis of disaccharide **16** served to assess the suitability of the DBDMH/TMSOTf activation system using functionalized resin **15** [48] as solid support (Scheme 1). After two coupling cycles with building block **8** followed by UV-cleavage, disaccharide **16** was obtained in 63% isolated yield.

**Scheme 2:**
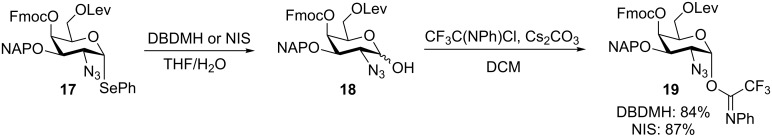
Hydrolysis of glycosyl selenide **17** with DBDMH.

Moreover, DBDMH performs as well as *N*-iodosuccinimide (NIS) in activating phenyl selenoglycoside **17** in the presence of water to furnish hemiacetal **18** en route to glycosyl imidate **19** (Scheme 2).

## Conclusion

The inexpensive reagent DBDMH has been demonstrated to be a powerful promoter for the activation of thioglycosides. This promoter is readily available, highly soluble, and shelf-stable. A variety of substrates containing diverse protecting groups have been investigated with promising results, while the stereoselectivity of the reactions follows reported trends. This promoter system was successfully used for automated glycan assembly.

## Supporting Information

File 1 Experimental details and full characterization data of all new compounds.
