# Supplementary material for: 1,3-Dibromo-5,5-dimethylhydantoin as promoter for glycosylations using thioglycosides
Source: Beilstein J Org Chem. 2017 Sep 22;13:1994–8. doi: 10.3762/bjoc.13.195 (PMC5629399; doi:10.3762/bjoc.13.195)
Supplement: File 1 — Experimental details and full characterization data of all new compounds. [file Beilstein_J_Org_Chem-13-1994-s001.pdf]

Supporting Information

for

1,3-Dibromo-5,5-dimethylhydantoin as promoter for glycosylations using thioglycosides

Fei-Fei Xu[1,2], Claney L. Pereira[1,3*] and Peter H. Seeberger[1,2*]

Address: [1]Department of Biomolecular Systems, Max Planck Institute of Colloids and Interfaces, Am Mühlenberg 1, 14476 Potsdam, Germany; [2]Department of Chemistry and Biochemistry, Freie Universität Berlin, Arnimallee 22, 14195 Berlin, Germany and [3]Vaxxilon Deutschland GmbH, Magnusstraße 11, 12489 Berlin, Germany

Email: Claney L. Pereira* - claney.pereira@vaxxilon.com; Peter H. Seeberger* - peter.seeberger@mpikg.mpg.de

* Corresponding author

Experimental details and full characterization data of all new compounds

Contents

General information **S2**

Preparation of building blocks **S2**

General glycosylation procedure **S7**

1,2-*Trans* glycosylation **S8**

1,2-*Cis* glycosylation **S18**

Solid-phase automated glycan assembly **S20**

Hydrolysis of glycosyl selenide **S23**

α/β selectivity determined using SFC **S25**

NMR spectra **S30**

References **S79**

General information

Chemicals were purchased as reagent grade and used without further purification unless stated otherwise. Anhydrous solvents were obtained from Waters Dry Solvent systems. Reactions were monitored by thin-layer chromatography (TLC) analysis, which was visualized by UV light (254 nm) and TLC sugar stain (1% (v/v) 3-methoxyphenol, 30% (v/v) sulfuric acid in ethanol). Flash column chromatography was performed on Kieselgel 60 with 230–400 mesh (Sigma-Aldrich, St. Louis, USA). ^{1}H NMR, ^{13}C NMR spectra were recorded on a 400 or 600 MHz Varian spectrometer at room temperature. Chemical shifts (in ppm) were calibrated with the solvent residual peak. Coupling constants (*J*) are reported in Hertz (Hz). Optical rotations (OR) were measured with a Schmidt & Haensch UniPol L 1000 at 589 nm and concentration (c) expressed in g/100 mL. High-resolution mass spectrometry (HRMS) was performed by Waters Xevo Q-Tof mass spectrometer. The α/β ratio was determined by supercritical fluid chromatography (SFC) from Waters.

Preparation of building blocks

Ethyl 2-*O*-benzoyl-4,6-di-*O*-benzyl-3-*O*-(9-fluorenylmethoxycarbonyl)-1-thio-β-D-galactopyranoside [1] (1)

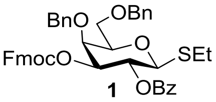

1

^{1}H NMR (400 MHz, $CDCl_3$) δ 8.08 – 8.00 (m, 2H), 7.68 (m, 2H), 7.58 – 7.48 (m, 1H), 7.48 – 7.27 (m, 16H), 7.11 (m, 2H), 5.75 (t, *J* = 9.9 Hz, 1H), 5.07 (dd, *J* = 10.0, 3.0 Hz, 1H), 4.79 (d, *J* = 11.5 Hz, 1H), 4.60 (d, *J* = 9.9 Hz, 1H), 4.51 (d, *J* = 11.8 Hz, 2H), 4.46 (d, *J* = 11.7 Hz, 1H), 4.30 (dd, *J* = 10.4, 7.2 Hz, 1H), 4.21 (dd, *J* = 10.4, 7.8 Hz, 1H), 4.14 (d, *J* = 3.1 Hz, 1H), 4.06 (t, *J* = 7.4 Hz, 1H), 3.82 (t, *J* = 6.5 Hz, 1H), 3.72 – 3.62 (m, 2H), 2.74 (m, 2H), 1.23 (t, *J* = 7.5 Hz, 3H).
NMR data was in accordance with previously reported values [1].

Methyl 2,3,4-tri-*O*-benzyl-α-D-glucopyranoside [2] (2)

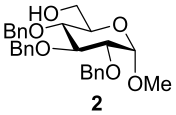

2

^{1}H NMR (400 MHz, $CDCl_3$) δ 7.39 – 7.26 (m, 15H), 4.99 (d, *J* = 10.9 Hz, 1H), 4.89 (d, *J* = 11.0 Hz, 1H), 4.86 – 4.78 (m, 2H), 4.68 – 4.63 (m, 2H), 4.56 (d, *J* = 3.5 Hz, 1H), 4.01 (t, *J* = 9.2 Hz, 1H), 3.77 (dd, *J* = 11.7, 2.6 Hz, 1H), 3.72 – 3.61 (m, 2H), 3.56 – 3.46 (m, 2H), 3.37 (s, 3H).

NMR data was in accordance with previously reported values [2].

Ethyl 2,3-di-*O*-benzoyl-4,6-*O*-benzylidene-1-thio-β-D-galactopyranoside [3] (4)

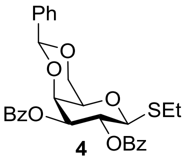

4

^{1}H NMR (600 MHz, $CDCl_3$) δ 8.02 – 7.93 (m, 4H), 7.56 – 7.46 (m, 4H), 7.42 – 7.33 (m, 7H), 5.96 (t, *J* = 9.9 Hz, 1H), 5.54 (s, 1H), 5.40 (dd, *J* = 10.0, 3.5 Hz, 1H), 4.74 (d, *J* = 9.9 Hz, 1H), 4.63 (dd, *J* = 3.6, 1.0 Hz, 1H), 4.42 (dd, *J* = 12.4, 1.6 Hz, 1H), 4.10 (dd, *J* = 12.4, 1.7 Hz, 1H), 3.73 (s, 1H), 2.95 (dq, *J* = 12.2, 7.4 Hz, 1H), 2.81 (dq, *J* = 12.2, 7.5 Hz, 1H), 1.31 (t, *J* = 7.5 Hz, 3H).

NMR data was in accordance with previously reported values [3].

Ethyl 2,3-di-*O*-benzoyl-6-*O*-benzyl-4-*O*-(9-fluorenylmethoxycarbonyl)-1-thio-β-D-glucopyranoside[4] (5)

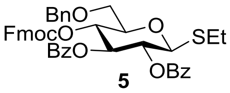

5

^{1}H NMR (400 MHz, $CDCl_3$) δ 7.96 (d, *J* = 7.5 Hz, 2H), 7.87 (d, *J* = 7.5 Hz, 2H), 7.71 (d, *J* = 7.4 Hz, 2H), 7.51 (t, *J* = 7.3 Hz, 1H), 7.48 – 7.21 (m, 15H), 7.17 (t, *J* = 7.5 Hz, 1H), 5.80 (t, *J* = 9.4 Hz, 1H), 5.49 (t, *J* = 9.6 Hz, 1H), 5.23 (t, *J* = 9.7 Hz, 1H), 4.76 (d, *J* = 10.1 Hz, 1H), 4.61 (d, *J* = 12.1 Hz, 1H), 4.56 (d, *J* = 12.0 Hz, 1H), 4.30 – 4.17 (m,

1H), 4.08 (t, *J* = 8.9 Hz, 1H), 4.01 – 3.90 (m, 2H), 3.74 (s, 2H), 2.87 – 2.68 (m, 2H), 1.28 (t, *J* = 7.5 Hz, 3H).

NMR data was in accordance with previously reported values [4].

Ethyl 3,4,6-tri-*O*-benzyl-2-*O*-levulinoyl-1-thio-β-D-glucopyranoside (6)

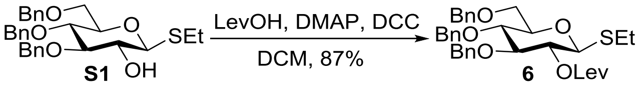

To a solution of ethyl 3,4,6-tri-*O*-benzyl-1-thio-β-D-glucopyranoside [5] (**S1**, 84 mg, 0.37 mmol) in DCM (4 mL) was added levulinic acid (75 µL, 0.74 mmol) followed by DMAP (45 mg, 0.37 mmol) and *N,N'*-dicyclohexylcarbodiimide (DCC, 153 mg, 0.74 mmol). The reaction mixture was stirred for 4 h at room temperature. The reaction was then diluted with DCM and washed with saturated aq $NaHCO_3$ solution. The organic layer was dried over Na_2SO_4, filtered and concentrated. The residue was purified by flash column chromatography to afford **6** (191 mg, 0.32 mmol, 87%) as white solid.

$[\alpha]_D^{25}$ -5.88 (c 1.97, $CHCl_3$); ^{1}H NMR (400 MHz, $CDCl_3$) δ 7.38 – 7.29 (m, 13H), 7.20 (dd, *J* = 7.2, 2.4 Hz, 2H), 5.11 – 5.01 (m, 1H), 4.82 (d, *J* = 10.9 Hz, 2H), 4.76 (d, *J* = 11.4 Hz, 1H), 4.63 (d, *J* = 12.1 Hz, 1H), 4.61 – 4.55 (m, 2H), 4.39 (d, *J* = 10.0 Hz, 1H), 3.82 – 3.68 (m, 4H), 3.53 (ddd, *J* = 7.7, 4.4, 2.2 Hz, 1H), 2.82 – 2.63 (m, 4H), 2.62 – 2.47 (m, 2H), 2.19 (s, 3H), 1.29 (t, *J* = 7.4 Hz, 3H); ^{13}C NMR (101 MHz, $CDCl_3$) δ 206.3, 171.7, 138.3, 138.2, 138.0, 128.5 (3C), 128.1, 128.0 (2C), 127.8, 127.7, 84.4, 83.5, 79.5, 77.9, 75.3, 75.2, 73.5, 72.2, 68.9, 38.0, 30.0, 28.2, 24.0, 15.1; HRMS (ESI) calcd for $C_{34}H_{40}O_7SNa$ $[M+Na]^+$ 615.2387; found: 615.2401.

Ethyl 3-*O*-benzyl-6-*O*-levulinoyl-4-*O*-(9-fluorenylmethoxycarbonyl)-2-*N*-trichloroacetyl-1-thio-β-D-glucosaminopyranoside [6] (7)

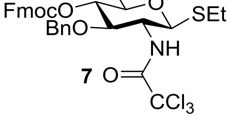

^{1}H NMR (400 MHz, $CDCl_3$) δ 7.68 (dd, *J* = 7.5, 3.7 Hz, 2H), 7.63 – 7.41 (m, 2H), 7.36 – 7.26 (m, 2H), 7.24 – 7.19 (m, 2H), 7.15 – 7.05 (m, 5H), 6.86 (d, *J* = 7.7 Hz, 1H), 4.98 (d, *J* = 10.3 Hz, 1H), 4.84 (dd, *J* = 9.9, 9.0 Hz, 1H), 4.54 (s, 2H), 4.42 (dd, *J* = 10.5, 6.8 Hz, 1H), 4.31 – 4.09 (m, 5H), 3.69 (ddd, *J* = 10.0, 5.2, 2.8 Hz, 1H), 3.56 (td, *J* = 10.1, 7.9 Hz, 1H), 2.73 – 2.58 (m, 4H), 2.56 – 2.49 (m, 2H), 2.09 (s, 3H), 1.28 – 1.12 (m, 3H).

NMR data was in accordance with previously reported values [6].

Ethyl 2-*O*-benzoyl-3,4-di-*O*-benzyl-6-*O*-(9-fluorenylmethoxycarbonyl)-1-thio-α-D-mannopyranoside (8)

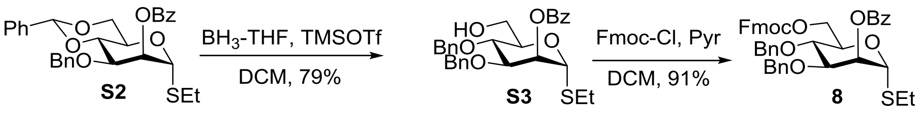

To a solution of ethyl 2-*O*-benzoyl-3-*O*-benzyl-4,6-*O*-benzylidene-1-thio-α-D-mannopyranoside [7] (**S2**, 592 mg, 1.17 mmol) in DCM (5 mL) was added 1 M solution of BH_3-THF (5.8 mL, 5.85 mmol) followed by trimethylsilyl trifluoromethanesulfonate (TMSOTf, 32 µL, 0.17 mmol). The reaction mixture was stirred for 2 h at room temperature. Triethylamine was then added to quench the reaction followed by the addition of MeOH. The reaction mixture was concentrated and purified by flash column chromatography to give ethyl 2-*O*-benzoyl-3,4-di-*O*-benzyl-1-thio-α-D-mannopyranoside (**S3**, 470 mg, 0.92 mmol, 79%) as colorless oil.
9-Fluorenylmethyl chloroformate (446 mg, 1.72 mmol) and pyridine (0.28 mL, 3.44 mmol) were added to a solution of **S3** (437 mg, 0.86 mmol) in DCM (5 mL). The reaction mixture was stirred overnight at room temperature, diluted with DCM and quenched with 1 M aq HCl solution. The organic layer was extracted, dried over Na_2SO_4, filtered and concentrated. The residue was purified by flash column chromatography to afford **8** (570 mg, 0.78 mmol, 91%) as white foam.

$[\alpha]_D^{25}$ +41.97 (c 1.16, $CHCl_3$); ^{1}H NMR (400 MHz, $CDCl_3$) δ 8.16 (dt, *J* = 8.2, 1.1 Hz, 2H), 7.80 (dd, *J* = 7.6, 1.1 Hz, 2H), 7.70 – 7.61 (m, 2H), 7.60 – 7.53 (m, 1H), 7.49 – 7.40 (m, 4H), 7.38 – 7.29 (m, 12H), 5.75 (dd, *J* = 3.0, 1.6 Hz, 1H), 5.46 (d, *J* = 1.6 Hz, 1H), 4.96 (d, *J* = 10.9 Hz, 1H), 4.82 (d, *J* = 11.2 Hz, 1H), 4.65 (d, *J* = 10.9 Hz, 1H),

4.60 (d, *J* = 11.2 Hz, 1H), 4.56 – 4.47 (m, 2H), 4.45 – 4.40 (m, 2H), 4.40 – 4.33 (m, 1H), 4.29 (t, *J* = 7.5 Hz, 1H), 4.09 (dd, *J* = 9.1, 2.9 Hz, 1H), 4.02 (t, *J* = 7.5 Hz, 1H), 2.80 – 2.59 (m, 2H), 1.33 (t, *J* = 7.4 Hz, 3H); ^{13}C NMR (101 MHz, CDCl3) δ 165.7, 155.3, 143.6, 143.4, 141.4, 138.0, 137.6, 133.4, 130.1, 129.9, 128.6 (2C), 128.5, 128.3, 128.0 (3C), 127.3, 125.4, 125.3, 120.2, 82.7, 78.8, 75.4, 74.1, 71.7, 70.7, 70.3, 70.1, 66.9, 46.9, 25.8, 15.1.; HRMS (ESI) calcd for $C_{44}H_{42}O_8SNa$ [M+Na]$^+$ 753.2493; found: 753.2503.

p-Tolyl 2-*O*-benzoyl-4-*O*-benzyl-3-*O*-(9-fluorenylmethoxycarbonyl)-1-thio-α-L-rhamnopyranoside (9)

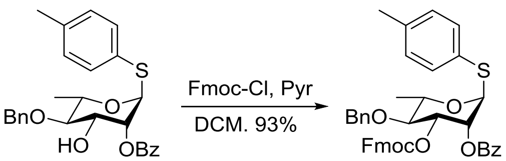

9-Fluorenylmethyl chloroformate (559 mg, 2.16 mmol) and pyridine (0.23 mL, 2.88 mmol) were added to a solution of *p*-tolyl 2-*O*-benzoyl-4-*O*-benzyl-1-thio-α-L-rhamnopyranoside [8] (**S4**, 669 mg, 1.44 mmol) in DCM (6 mL). The reaction mixture was stirred overnight at room temperature. The reaction was then diluted with DCM and quenched with 1 M aq HCl solution. The organic layer was extracted, dried over Na_2SO_4, filtered and concentrated. The residue was purified by flash column chromatography to afford **9** (932 mg, 1.34 mmol, 93%) as white foam.

$[\alpha]_D^{25}$ -46.86 (c 0.93, $CHCl_3$); ^{1}H NMR (400 MHz, $CDCl_3$) δ 8.17 – 7.98 (m, 2H), 7.74 (ddd, *J* = 7.7, 2.3, 1.1 Hz, 2H), 7.67 – 7.60 (m, 1H), 7.55 (ddd, *J* = 7.6, 2.1, 1.0 Hz, 2H), 7.49 (t, *J* = 7.8 Hz, 2H), 7.42 – 7.30 (m, 9H), 7.23 (td, *J* = 7.5, 1.1 Hz, 1H), 7.19 – 7.11 (m, 3H), 5.89 (dd, *J* = 3.2, 1.7 Hz, 1H), 5.49 (d, *J* = 1.6 Hz, 1H), 5.29 (dd, *J* = 9.7, 3.2 Hz, 1H), 4.88 (d, *J* = 11.1 Hz, 1H), 4.71 (d, *J* = 11.1 Hz, 1H), 4.61 – 4.51 (m, 1H), 4.42 (dq, *J* = 9.4, 6.2 Hz, 1H), 4.33 – 4.24 (m, 2H), 3.76 (t, *J* = 9.5 Hz, 1H), 2.33 (s, 3H), 1.43 (d, *J* = 6.1 Hz, 3H); ^{13}C NMR (101 MHz, CDCl3) δ 165.6, 154.3, 143.7, 143.2, 141.4, 141.3, 138.3, 137.9, 133.6, 132.7, 130.1 (2C), 129.7 (2C), 128.6 (2C), 128.1, 128.0 (2C), 127.9, 127.3, 127.2, 125.5, 125.2, 120.1 (2C), 86.2, 78.8, 76.8, 75.4, 72.1, 70.4, 69.1, 46.8, 21.3, 18.1; HRMS (ESI) calcd for $C_{42}H_{38}O_7SNa$ [M+Na]$^+$ 709.2230; found: 709.2238.

Benzyl (ethyl 3,4-di-*O*-benzyl-2-*O*-levulinoyl-1-thio-β-D-glucopyranosid)uronate [9] **(10)**

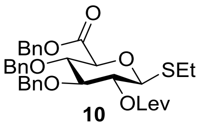

10

^{1}H NMR (400 MHz, $CDCl_3$) δ 7.52 – 7.17 (m, 13H), 7.16 – 7.00 (m, 2H), 5.16 (s, 2H), 5.05 (t, *J* = 9.5 Hz, 1H), 4.76 (d, *J* = 11.4 Hz, 1H), 4.73 – 4.63 (m, 2H), 4.45 (d, *J* = 10.7 Hz, 1H), 4.40 (d, *J* = 10.0 Hz, 1H), 3.95 (d, *J* = 9.7 Hz, 1H), 3.89 (t, *J* = 9.2 Hz, 1H), 3.68 (t, *J* = 8.9 Hz, 1H), 2.80 – 2.57 (m, 4H), 2.57 – 2.39 (m, 2H), 2.15 (s, 3H), 1.22 (t, *J* = 7.4 Hz, 3H).

NMR data was in accordance with previously reported values [9].

Methyl 2,3,6-tri-*O*-benzyl-α-D-glucopyranoside [10] (11)

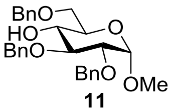

11

^{1}H NMR (400 MHz, $CDCl_3$) δ 7.40 – 7.26 (m, 15H), 5.01 (d, *J* = 11.4 Hz, 1H), 4.78 (d, *J* = 12.1 Hz, 1H), 4.74 (d, *J* = 11.4 Hz, 1H), 4.66 (d, *J* = 12.1 Hz, 1H), 4.63 (d, *J* = 3.5 Hz, 1H), 4.59 (d, *J* = 12.2 Hz, 1H), 4.54 (d, *J* = 12.2 Hz, 1H), 3.79 (t, *J* = 9.1 Hz, 1H), 3.74 – 3.66 (m, 3H), 3.61 (t, *J* = 9.1 Hz, 1H), 3.54 (dd, *J* = 9.5, 3.5 Hz, 1H), 3.39 (s, 3H).

NMR data was in accordance with previously reported values [10].

General glycosylation procedure

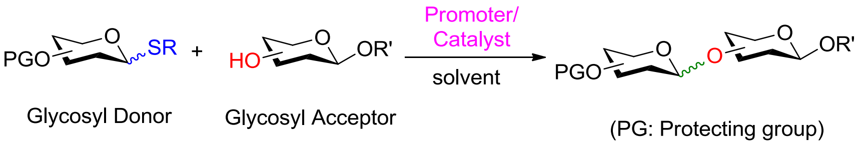

Both donor (51 µmol) and acceptor (43 µmol) were co-evaporated three times with anhydrous toluene and kept under high vacuum for 1 h. The mixture was dissolved in the indicated solvent (3 mL) followed by the addition of activated molecular sieves (AW-300). The solution was stirred for 10 min at room temperature and cooled down to the indicated temperature. DBDMH (37 µmol) and TfOH (0.522 µmol) were added and the mixture was stirred for one hour. Then the reaction was quenched with Et_3N, diluted with DCM and extracted with 10% aq $Na_2S_2O_3$ solution. The aqueous phase was washed with DCM twice and the combined organic layer was dried over Na_2SO_4, filtered and concentrated. The residue was purified by flash column chromatography to obtain pure disaccharide.

1,2-*Trans* glycosylation

Table S1: 1,2-*Trans* glycosylation activated by DBDMH with a variety of building blocks

Entry	Donor	Acceptor	Product[a]	Solvents for flash column chromatography	Yield (%)
1	**1**	**2**	**3**	20-25% ethyl acetate in hexanes	92
2	**4**	**2**	**S5**	30% ethyl acetate in hexanes	95
3	**5**	**2**	**S6**	5% acetone in toluene	98
4	**6**	**2**	**S7**	25% ethyl acetate in hexanes	94
5	**7**	**2**	**S8**	50% ethyl acetate in hexanes	91
6	**8**	**2**	**S9**	25% ethyl acetate in hexanes	96
7	**9**	**2**	**S10**	20% ethyl acetate in hexanes	91
8	**10**	**2**	**S11**	4% acetone in toluene	39
9	**1**	**11**	**S12**	25% ethyl acetate in hexanes	88
10	**4**	**11**	**S13**	28% ethyl acetate in hexanes	88
11	**5**	**11**	**S14**	25% ethyl acetate in hexanes	87
12	**6**	**11**	**S15**	30% ethyl acetate in hexanes	89
13	**7**	**11**	**S16**	20% acetone in toluene	60
14	**8**	**11**	**S17**	25% ethyl acetate in hexanes	89
15	**9**	**11**	**S18**	20% ethyl acetate in hexanes	86
16	**10**	**11**	**S19**	30% ethyl acetate in hexanes	45

[a]Fmoc protecting groups were removed during the quenching process because of triethylamine.

Methyl 2-*O*-benzoyl-4,6-di-*O*-benzyl-β-D-galactopyranosyl-(1→6)-2,3,4-tri-*O*-benzyl-α-D-glucopyranoside (3)

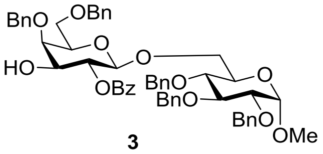

3

$[\alpha]_D^{25}$ +7.95 (c 1.63, $CHCl_3$); ^{1}H NMR (400 MHz, $CDCl_3$) δ 8.00 (dd, *J* = 8.2, 1.1 Hz, 2H), 7.54 – 7.45 (m, 1H), 7.41 – 7.24 (m, 25H), 7.19 – 7.09 (m, 2H), 5.34 (dd, *J* = 10.0, 8.0 Hz, 1H), 4.92 (d, *J* = 10.9 Hz, 1H), 4.78 – 4.68 (m, 4H), 4.62 – 4.50 (m, 5H), 4.48 – 4.42 (m, 2H), 4.13 (d, *J* = 9.1 Hz, 1H), 3.97 (d, *J* = 3.5 Hz, 1H), 3.91 (t, *J* = 9.3 Hz, 1H), 3.83 – 3.64 (m, 6H), 3.48 – 3.34 (m, 2H), 3.19 (s, 3H), 2.44 (d, *J* = 10.0 Hz, 1H); ^{13}C NMR (101 MHz, $CDCl_3$) δ 166.7, 139.0, 138.4, 138.3, 138.2, 137.8, 133.2, 129.9 (2C), 128.7, 128.6, 128.5, 128.4 (2C), 128.2, 128.1 (2C), 128.0 (3C), 127.7 (2C), 127.6, 101.4 ($^1J_{C-H}$ = 168 Hz), 97.9 ($^1J_{C-H}$ = 172 Hz), 82.1, 80.0, 77.7, 75.7, 74.8, 74.0, 73.7 (2C), 73.5, 73.4, 69.7, 68.3, 68.1, 55.1; HRMS (ESI) calcd for $C_{55}H_{58}O_{12}Na$ $[M+Na]^+$ 933.3826; found: 933.3779.

Methyl 2,3-di-*O*-benzoyl-4,6-*O*-benzylidene-β-D-galactopyranosyl-(1→6)-2,3,4-tri-*O*-benzyl-α-D-glucopyranoside (S5)

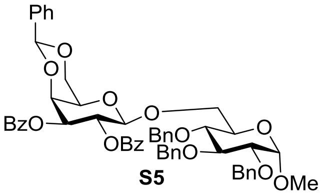

S5

Prepared from compounds **4** and **2**. $[\alpha]_D^{25}$ +94.2 (c 1.15, $CHCl_3$); ^{1}H NMR (400 MHz, $CDCl_3$) δ 7.95 – 7.80 (m, 4H), 7.46 – 7.07 (m, 26H), 5.83 (dd, *J* = 10.4, 8.0 Hz, 1H), 5.46 (s, 1H), 5.26 (dd, *J* = 10.4, 3.6 Hz, 1H), 4.82 (d, *J* = 10.9 Hz, 1H), 4.68 – 4.58 (m, 3H), 4.56 – 4.45 (m, 3H), 4.39 – 4.23 (m, 3H), 4.12 (d, *J* = 9.1 Hz, 1H), 4.06 – 3.98 (m, 1H), 3.82 (t, *J* = 9.2 Hz, 1H), 3.71 – 3.59 (m, 2H), 3.52 (s, 1H), 3.34 – 3.22 (m, 2H), 3.10 (s, 3H); ^{13}C NMR (101 MHz, $CDCl_3$) δ 166.4, 165.2, 139.0, 138.4, 138.3, 137.6, 133.5, 133.1, 130.1, 129.8 (2C), 129.2, 129.0, 128.5 (2C), 128.4 (2C), 128.2 (2C), 128.0 (2C), 127.8, 127.7, 127.6, 126.4, 101.8($^1J_{C-H}$ = 156 Hz), 100.9, 97.8 ($^1J_{C-H}$

= 168 Hz), 82.1, 80.0, 77.8, 75.6, 74.8, 73.7, 73.4, 72.9, 69.8, 69.2, 69.0, 68.2, 66.7, 55.1; HRMS (ESI) calcd for $C_{55}H_{54}O_{13}Na$ $[M+Na]^+$ 945.3462; found: 945.3466.

Methyl 2,3-di-*O*-benzoyl-6-*O*-benzyl-β-D-glucopyranosyl-(1→6)-2,3,4-tri-*O*-benzyl-α-D-glucopyranoside (S6)

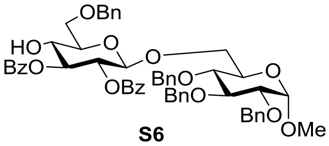

S6

Prepared from compounds **5** and **2**. $[\alpha]_D^{25}$ +37.6 (c 1.93, $CHCl_3$); ^1H NMR (400 MHz, $CDCl_3$) δ 7.88 (dd, *J* = 8.3, 1.2 Hz, 2H), 7.79 (dd, *J* = 8.3, 1.2 Hz, 2H), 7.48 – 7.37 (m, 1H), 7.28 – 7.07 (m, 23H), 7.01 – 6.91 (m, 2H), 5.47 – 5.34 (m, 2H), 4.81 (d, *J* = 10.9 Hz, 1H), 4.69 – 4.56 (m, 3H), 4.56 – 4.47 (m, 3H), 4.43 – 4.35 (m, 2H), 4.19 (d, *J* = 11.1 Hz, 1H), 4.05 (d, *J* = 8.8 Hz, 1H), 3.86 (t, *J* = 9.0 Hz, 1H), 3.83 – 3.75 (m, 3H), 3.67 – 3.56 (m, 3H), 3.35 (dd, *J* = 9.7, 3.5 Hz, 1H), 3.32 – 3.27 (m, 1H), 3.13 (s, 3H); ^{13}C NMR (101 MHz, $CDCl_3$) δ 167.2, 165.2, 138.9, 138.3, 137.7, 133.5, 133.2, 130.1, 129.8, 129.4, 129.1, 128.6 (2C), 128.5, 128.4 (2C), 128.2 (2C), 128.1, 128.0, 127.9, 127.7, 127.6, 101.2 ($^1J_{C\text{-}H}$ = 164 Hz), 98.1 ($^1J_{C\text{-}H}$ = 172 Hz), 82.0, 79.8, 77.5, 76.6, 75.7, 74.8, 74.6, 73.9, 73.5, 71.5 (2C), 70.4, 69.6, 68.3, 55.1; HRMS (ESI) calcd for $C_{55}H_{56}O_{13}Na$ $[M+Na]^+$ 947.3619; found: 947.3572.

Methyl 3,4,6-tri-*O*-benzyl-2-*O*-levulinoyl-β-D-glucopyranosyl-(1→6)-2,3,4-tri-*O*-benzyl-α-D-glucopyranoside (S7)

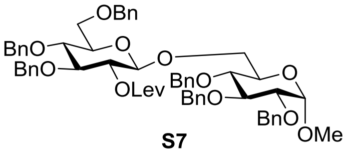

S7

Prepared from compounds **6** and **2**. $[\alpha]_D^{25}$ +13.1 (c 2.02, $CHCl_3$); ^1H NMR (400 MHz, $CDCl_3$) δ 7.29 – 7.17 (m, 28H), 7.09 (dd, *J* = 7.1, 2.5 Hz, 2H), 4.97 (td, *J* = 7.9, 2.3 Hz, 1H), 4.89 (d, *J* = 10.9 Hz, 1H), 4.76 (d, *J* = 10.9 Hz, 1H), 4.74 – 4.66 (m, 4H), 4.62 (d, *J* = 11.4 Hz, 1H), 4.58 (d, *J* = 12.1 Hz, 1H), 4.53 – 4.41 (m, 5H), 4.32 (d, *J* = 8.0 Hz, 1H), 4.00 (dd, *J* = 10.7, 1.6 Hz, 1H), 3.89 (t, *J* = 9.3 Hz, 1H), 3.73 – 3.64 (m, 1H),

3.64 – 3.53 (m, 5H), 3.45 (dd, J = 9.6, 3.5 Hz, 1H), 3.43 – 3.33 (m, 2H), 3.27 (s, 3H), 2.59 – 2.50 (m, 1H), 2.49 – 2.38 (m, 1H), 2.34 (td, J = 6.8, 3.3 Hz, 2H), 1.96 (s, 3H); ^{13}C NMR (101 MHz, $CDCl_3$) δ 206.1, 171.4, 138.9, 138.4, 138.3, 138.2, 138.0, 128.6, 128.5, 128.5, 128.2, 128.1 (2C), 128.0 (2C), 127.9, 127.8 (2C), 127.7, 101.0 ($^1J_{C-H}$ = 156 Hz), 98.1 ($^1J_{C-H}$ = 168 Hz), 83.1, 82.1, 80.0, 78.1, 77.9, 75.8, 75.5, 75.1, 74.9, 73.6, 73.5 (2C), 69.9, 68.9, 68.1, 55.2, 37.9, 29.9, 28.1; HRMS (ESI) calcd for $C_{60}H_{66}O_{13}Na$ $[M+Na]^+$ 1017.4401; found: 1017.4335.

Methyl 3-*O*-benzyl-6-*O*-levulinoyl-2-*N*-trichloroacetyl-β-D-glucosaminopyranosyl-(1→6)-2,3,4-tri-*O*-benzyl-α-D-glucopyranoside (S8)

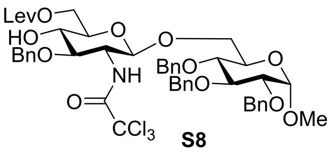

S8

Prepared from compounds **7** and **2**. $[\alpha]_D^{25}$ -0.32 (c 1.85, $CHCl_3$); ^{1}H NMR (400 MHz, $CDCl_3$) δ 7.34 – 7.12 (m, 20H), 6.84 (d, J = 7.6 Hz, 1H), 4.90 (d, J = 11.1 Hz, 1H), 4.79 (d, J = 11.1 Hz, 1H), 4.75 – 4.64 (m, 5H), 4.56 (d, J = 12.2 Hz, 1H), 4.52 – 4.42 (m, 3H), 4.13 (dd, J = 12.2, 2.1 Hz, 1H), 4.01 – 3.84 (m, 3H), 3.69 – 3.62 (m, 1H), 3.58 (dd, J = 10.9, 4.1 Hz, 1H), 3.54 – 3.32 (m, 5H), 3.28 (s, 3H), 2.91 (d, J = 4.2 Hz, 1H), 2.66 (t, J = 6.4 Hz, 2H), 2.49 (t, J = 6.4 Hz, 2H), 2.09 (s, 3H); ^{13}C NMR (101 MHz, $CDCl_3$) δ 206.9, 173.6, 161.9, 139.0, 138.5, 138.2, 138.1, 128.7, 128.6, 128.5, 128.4, 128.2 (3C), 128.0, 127.9, 127.8 (2C), 127.6, 99.6 ($^1J_{C-H}$ = 168 Hz), 98.1 ($^1J_{C-H}$ = 172 Hz), 92.6, 82.1, 79.7, 79.1, 77.6, 75.6, 75.0, 74.9, 74.1, 73.4, 71.1, 69.6, 67.9, 63.2, 58.4, 55.4, 38.1, 29.9, 28.0; HRMS (ESI) calcd for $C_{48}H_{54}Cl_3NO_{13}Na$ $[M+Na]^+$ 980.2558; found: 980.2511.

Methyl 2-*O*-benzoyl-3,4-di-*O*-benzyl-α-D-mannopyranosyl-(1→6)-2,3,4-tri-*O*-benzyl-α-D-glucopyranoside (S9)

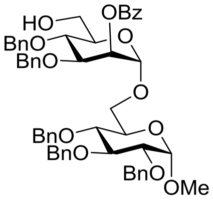

S9

Prepared from compounds **8** and **2**. $[\alpha]_D^{25}$ +21.3 (c 1.77, $CHCl_3$); ^1H NMR (400 MHz, $CDCl_3$) δ 8.11 – 8.02 (m, 2H), 7.63 – 7.54 (m, 1H), 7.46 (t, *J* = 7.7 Hz, 2H), 7.40 – 7.15 (m, 25H), 5.60 (dd, *J* = 3.1, 1.9 Hz, 1H), 5.02 – 4.94 (m, 2H), 4.93 – 4.86 (m, 2H), 4.79 (d, *J* = 11.4 Hz, 2H), 4.75 (d, *J* = 11.6 Hz, 1H), 4.69 (d, *J* = 12.1 Hz, 1H), 4.65 – 4.58 (m, 2H), 4.55 (d, *J* = 11.6 Hz, 1H), 4.49 (d, *J* = 11.2 Hz, 1H), 4.05 – 3.90 (m, 3H), 3.79 (dd, *J* = 11.2, 4.6 Hz, 1H), 3.75 – 3.60 (m, 5H), 3.56 (dd, *J* = 9.6, 3.5 Hz, 1H), 3.44 (dd, *J* = 9.9, 8.9 Hz, 1H), 3.32 (s, 3H); ^{13}C NMR (101 MHz, $CDCl_3$) δ 165.7, 138.8, 138.5, 138.3, 138.2, 137.9, 133.4, 130.0 (2C), 128.6 (2C), 128.5 (2C), 128.3, 128.2 (3C), 128.1 (2C), 127.8 (3C), 127.7, 98.2 ($^1J_{C-H}$ = 176 Hz), 98.0 ($^1J_{C-H}$ = 172 Hz), 82.2, 80.2, 77.7, 77.6, 75.9, 75.3, 75.1, 74.0, 73.5, 72.1, 71.5, 69.8, 69.0, 66.3, 62.1, 55.3; HRMS (ESI) calcd for $C_{55}H_{58}O_{12}Na$ $[M+Na]^+$ 933.3826; found: 933.3831.

Methyl 2-*O*-benzoyl-4-*O*-benzyl-α-L-rhamnopyranosyl-(1→6)-2,3,4-tri-*O*-benzyl-α-D-glucopyranoside (S10)

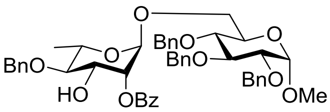

S10

Prepared from compounds **9** and **2**. $[\alpha]_D^{25}$ +30.9 (c 1.36, $CHCl_3$); ^1H NMR (400 MHz, $CDCl_3$) δ 8.05 – 8.00 (m, 2H), 7.63 – 7.53 (m, 1H), 7.49 – 7.43 (m, 2H), 7.38 – 7.16 (m, 20H), 5.30 (dd, *J* = 3.5, 1.7 Hz, 1H), 4.98 (d, *J* = 10.9 Hz, 1H), 4.90 (d, *J* = 11.1 Hz, 1H), 4.84 – 4.79 (m, 2H), 4.79 – 4.75 (m, 2H), 4.72 (d, *J* = 11.1 Hz, 1H), 4.66 (d, *J* = 12.1 Hz, 1H), 4.60 – 4.53 (m, 2H), 4.18 (dd, *J* = 9.4, 3.5 Hz, 1H), 3.98 (t, *J* = 9.2 Hz, 1H), 3.86 – 3.76 (m, 2H), 3.73 (ddd, *J* = 10.1, 5.3, 1.7 Hz, 1H), 3.55 – 3.48 (m, 2H), 3.46 (d, *J* = 9.3 Hz, 1H), 3.42 (d, *J* = 9.6 Hz, 1H), 3.37 (s, 3H), 1.33 (d, *J* = 6.3

Hz, 3H); ^{13}C NMR (101 MHz, $CDCl_3$) δ 166.3, 138.8, 138.3 (2C), 138.2, 133.5, 130.0, 129.9, 128.7, 128.6 (3C), 128.5, 128.3, 128.2, 128.1 (2C), 127.9, 127.7, 98.0 ($^{1}J_{C-H}$ = 172 Hz), 97.8 ($^{1}J_{C-H}$ = 172 Hz), 82.2, 81.8, 80.1, 77.8, 75.9, 75.4, 75.2, 73.5, 73.3, 70.6, 70.1, 67.6, 66.5, 55.3, 18.2; HRMS (ESI) calcd for $C_{48}H_{52}O_{11}Na$ $[M+Na]^+$ 827.3407; found: 827.3413.

Methyl (benzyl 3,4-di-*O*-benzyl-2-*O*-levulinoyl-β-D-glucopyranosyluronate)-(1→6)-2,3,4-tri-*O*-benzyl-α-D-glucopyranoside (S11)

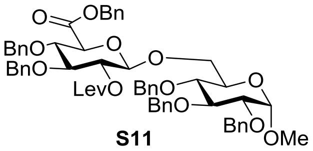

S11

Prepared from compounds **10** and **2**. $[\alpha]_D^{25}$ +2.79 (c 1.28, $CHCl_3$); ^{1}H NMR (400 MHz, $CDCl_3$) δ 7.27 – 7.15 (m, 28H), 7.02 (dd, *J* = 6.5, 3.0 Hz, 2H), 5.12 – 5.03 (m, 2H), 4.98 (dd, *J* = 9.0, 7.7 Hz, 1H), 4.89 (d, *J* = 10.9 Hz, 1H), 4.76 (d, *J* = 10.9 Hz, 1H), 4.73 – 4.63 (m, 3H), 4.62 – 4.55 (m, 3H), 4.50 (d, *J* = 3.5 Hz, 1H), 4.45 (d, *J* = 11.0 Hz, 1H), 4.41 – 4.33 (m, 2H), 3.95 (dd, *J* = 10.7, 1.5 Hz, 1H), 3.92 – 3.84 (m, 3H), 3.71 – 3.63 (m, 1H), 3.55 (td, *J* = 11.1, 10.0, 4.9 Hz, 2H), 3.44 (dd, *J* = 9.6, 3.5 Hz, 1H), 3.36 – 3.28 (m, 1H), 3.25 (s, 3H), 2.59 – 2.51 (m, 1H), 2.51 – 2.40 (m, 1H), 2.37 – 2.28 (m, 2H), 1.97 (s, 3H); ^{13}C NMR (101 MHz, $CDCl_3$) δ 206.0, 171.3, 168.1, 138.9, 138.3, 138.0, 137.8, 135.1, 128.7, 128.6 (4C), 128.5 (2C), 128.2, 128.1 (2C), 128.0 (2C), 127.9 (3C), 127.7, 101.4 ($^{1}J_{C-H}$ = 164 Hz), 98.0 ($^{1}J_{C-H}$ = 172 Hz), 82.1, 80.1, 79.3, 77.9, 75.8, 75.1, 75.0, 74.9 (2C), 73.5, 73.1, 69.9, 68.4, 67.5, 55.2, 37.8, 29.9, 28.0; HRMS (ESI) calcd for $C_{60}H_{64}O_{14}Na$ $[M+Na]^+$ 1031.4194; found: 1031.4114.

Methyl 2-*O*-benzoyl-4,6-di-*O*-benzyl-β-D-galactopyranosyl-(1→4)-2,3,6-tri-*O*-benzyl-α-D-glucopyranoside (S12)

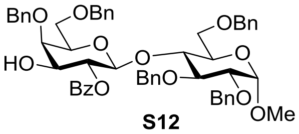

S12

Prepared from compounds **1** and **11**. $[\alpha]_D^{25}$ +2.89 (c 1.55, $CHCl_3$); ^{1}H NMR (400 MHz, $CDCl_3$) δ 7.84 (dd, *J* = 8.2, 1.1 Hz, 2H), 7.52 – 7.43 (m, 1H), 7.39 – 7.12 (m, 27H), 5.12 (dd, *J* = 10.0, 8.0 Hz, 1H), 4.94 (d, *J* = 10.9 Hz, 1H), 4.71 (d, *J* = 11.8 Hz, 2H), 4.63 (d, *J* = 11.8 Hz, 1H), 4.59 (d, *J* = 11.8 Hz, 1H), 4.57 (d, *J* = 5.0 Hz, 1H), 4.55 – 4.49 (m, 2H), 4.45 (d, *J* = 3.7 Hz, 1H), 4.33 (d, *J* = 11.7 Hz, 1H), 4.27 – 4.18 (m, 2H), 3.86 – 3.70 (m, 3H), 3.59 (dd, *J* = 10.7, 3.3 Hz, 1H), 3.52 – 3.29 (m, 7H), 3.20 (s, 3H), 2.18 (d, *J* = 10.3 Hz, 1H); ^{13}C NMR (101 MHz, $CDCl_3$) δ 166.5, 139.7, 138.5, 138.3, 138.1, 138.0, 133.3, 129.9 (2C), 128.7, 128.6 (2C), 128.5 (2C), 128.2, 128.1 (2C), 128.0, 127.9 (3C), 127.2, 100.4 ($^1J_{C\text{-}H}$ = 168 Hz), 98.5 ($^1J_{C\text{-}H}$ = 172 Hz), 80.2, 79.2, 76.7, 75.6, 75.4, 74.8, 73.7, 73.6, 73.4, 73.3, 69.7, 68.1, 67.9, 55.4; HRMS (ESI) calcd for $C_{55}H_{58}O_{12}Na$ $[M+Na]^+$ 933.3826; found: 933.3832.

Methyl 2,3-di-*O*-benzoyl-4,6-*O*-benzylidene-β-D-galactopyranosyl-(1→4)-2,3,6-tri-*O*-benzyl-α-D-glucopyranoside (S13)

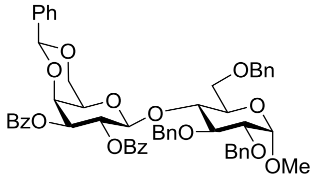

S13

Prepared from compounds **4** and **11**. $[\alpha]_D^{25}$ +58.6 (c 1.77, $CHCl_3$); ^{1}H NMR (400 MHz, $CDCl_3$) δ 7.90 – 7.84 (m, 2H), 7.84 – 7.77 (m, 2H), 7.40 – 7.14 (m, 26H), 5.72 (dd, *J* = 10.4, 8.1 Hz, 1H), 5.42 (s, 1H), 5.08 (d, *J* = 10.8 Hz, 1H), 5.00 (dd, *J* = 10.4, 3.7 Hz, 1H), 4.77 (d, *J* = 10.8 Hz, 1H), 4.73 (d, *J* = 12.2 Hz, 1H), 4.69 (d, *J* = 8.1 Hz, 1H), 4.60 – 4.50 (m, 2H), 4.45 (d, *J* = 3.6 Hz, 1H), 4.40 (d, *J* = 3.5 Hz, 1H), 4.22 – 4.15 (m, 2H), 3.90 – 3.82 (m, 3H), 3.59 (dd, *J* = 10.7, 3.3 Hz, 1H), 3.48 – 3.40 (m, 2H), 3.33 (dd, *J* = 10.7, 1.7 Hz, 1H), 3.20 (s, 3H), 3.18 (s, 1H); ^{13}C NMR (101 MHz, $CDCl_3$) δ 166.3, 165.1, 139.4, 138.5, 138.2, 137.8, 133.4, 133.3, 130.0, 129.8, 129.6, 129.3, 128.9, 128.7, 128.5 (2C), 128.3, 128.2 (3C), 128.1, 127.9, 127.3, 126.4, 100.9 ($^1J_{C\text{-}H}$ = 160 Hz), 98.4 ($^1J_{C\text{-}H}$ = 168 Hz), 80.4, 79.3, 77.6, 75.9, 73.7, 73.6, 73.1, 70.0, 69.6, 68.8, 68.1, 66.6, 55.4; HRMS (ESI) calcd for $C_{55}H_{54}O_{13}Na$ $[M+Na]^+$ 945.3462; found: 945.3466.

Methyl 2,3-di-*O*-benzoyl-6-*O*-benzyl-β-D-glucopyranosyl-(1→4)-2,3,6-tri-*O*-benzyl-α-D-glucopyranoside (S14)

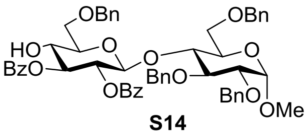

S14

Prepared from compounds **5** and **11**. $[\alpha]_D^{25}$ +20.2 (c 1.44, $CHCl_3$); ^{1}H NMR (400 MHz, $CDCl_3$) δ 7.97 – 7.90 (m, 2H), 7.87 – 7.79 (m, 2H), 7.51 – 7.21 (m, 26H), 5.32 (dd, *J* = 9.9, 7.8 Hz, 1H), 5.25 (dd, *J* = 9.9, 8.9 Hz, 1H), 4.99 (d, *J* = 11.3 Hz, 1H), 4.81 (d, *J* = 11.3 Hz, 1H), 4.75 (d, *J* = 12.2 Hz, 1H), 4.70 – 4.62 (m, 2H), 4.59 (d, *J* = 12.2 Hz, 1H), 4.52 (d, *J* = 3.7 Hz, 1H), 4.44 (d, *J* = 11.9 Hz, 1H), 4.38 (d, *J* = 11.9 Hz, 1H), 4.28 (d, *J* = 12.1 Hz, 1H), 3.92 – 3.78 (m, 3H), 3.64 – 3.50 (m, 3H), 3.49 – 3.38 (m, 3H), 3.35 (dd, *J* = 10.7, 2.0 Hz, 1H), 3.25 (s, 3H); ^{13}C NMR (101 MHz, $CDCl_3$) δ 166.9, 165.1, 139.7, 138.4, 137.9, 137.7, 133.4, 133.3, 130.0, 129.8, 129.4, 129.3, 128.9, 128.6, 128.5 (3C), 128.3, 128.2, 128.0, 127.9, 127.8, 127.6, 127.2, 100.4 ($^1J_{C-H}$ = 164 Hz), 98.5 ($^1J_{C-H}$ = 172 Hz), 80.2, 79.0, 77.1, 76.4, 75.3, 73.9, 73.7 (3C), 72.3, 72.1, 70.9, 69.6, 67.7, 55.4; HRMS (ESI) calcd for $C_{55}H_{56}O_{13}Na$ $[M+Na]^+$ 947.3619; found: 947.3624.

Methyl 3,4,6-tri-*O*-benzyl-2-*O*-levulinoyl-β-D-glucopyranosyl-(1→4)-2,3,6-tri-*O*-benzyl-α-D-glucopyranoside (S15)

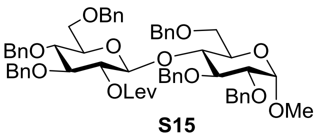

S15

Prepared from compounds **6** and **11**. $[\alpha]_D^{25}$ +10.1 (c 1.88, $CHCl_3$); ^{1}H NMR (400 MHz, $CDCl_3$) δ 7.29 – 7.10 (m, 30H), 4.98 (d, *J* = 11.4 Hz, 1H), 4.85 (dd, *J* = 9.5, 8.1 Hz, 1H), 4.71 – 4.62 (m, 5H), 4.57 – 4.48 (m, 3H), 4.45 (d, *J* = 11.0 Hz, 1H), 4.39 – 4.30 (m, 3H), 4.25 (d, *J* = 12.1 Hz, 1H), 3.84 – 3.70 (m, 3H), 3.65 – 3.51 (m, 4H), 3.46 – 3.32 (m, 3H), 3.29 (s, 3H), 3.22 (ddd, *J* = 9.8, 4.5, 1.7 Hz, 1H), 2.65 – 2.57 (m, 1H), 2.57 – 2.46 (m, 1H), 2.39 – 2.29 (m, 1H), 2.27 – 2.17 (m, 1H), 2.05 (s, 3H); ^{13}C NMR (101 MHz, $CDCl_3$) δ 206.2, 171.4, 139.8, 138.5 (3C), 138.2, 138.0, 128.7, 128.5, 128.4 (2C), 128.3, 128.2, 128.1, 128.0 (2C), 127.9, 127.8, 127.7, 127.5, 127.1, 100.2

($^{1}J_{C-H}$ = 164 Hz), 98.5 ($^{1}J_{C-H}$ = 172 Hz), 83.2, 80.4, 79.0, 78.2, 75.5, 75.4, 75.1, 74.9, 74.2, 73.8, 73.7, 73.5, 69.9, 68.9, 68.1, 55.4, 37.8, 30.0, 28.0; HRMS (ESI) calcd for $C_{60}H_{66}O_{13}Na$ $[M+Na]^+$ 1017.4401; found: 1017.4410.

Methyl 3-*O*-benzyl-6-*O*-levulinoyl-2-*N*-trichloroacetyl-β-D-glucosaminopyranosyl-(1→4)-2,3,6-tri-*O*-benzyl-α-D-glucopyranoside (S16)

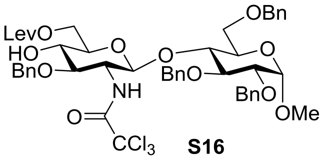

S16

Prepared from compounds **7** and **11**. $[\alpha]_D^{25}$ -22.2 (c 1.51, $CHCl_3$); ^{1}H NMR (400 MHz, $CDCl_3$) δ 7.32 – 7.18 (m, 20H), 6.42 (d, *J* = 8.1 Hz, 1H), 4.92 (d, *J* = 11.4 Hz, 1H), 4.74 (d, *J* = 11.2 Hz, 1H), 4.70 – 4.59 (m, 4H), 4.57 (d, *J* = 8.2 Hz, 1H), 4.53 – 4.47 (m, 2H), 4.44 – 4.34 (m, 2H), 3.96 (dd, *J* = 12.2, 2.1 Hz, 1H), 3.88 (t, *J* = 9.3 Hz, 1H), 3.76 (t, *J* = 9.2 Hz, 1H), 3.63 – 3.36 (m, 7H), 3.27 (s, 3H), 3.09 (dt, *J* = 9.5, 3.1 Hz, 1H), 2.99 (d, *J* = 4.2 Hz, 1H), 2.68 – 2.51 (m, 2H), 2.47 – 2.29 (m, 2H), 2.06 (s, 3H); ^{13}C NMR (101 MHz, $CDCl_3$) δ 207.0, 173.8, 161.7, 139.7, 138.4, 138.2, 138.0, 128.8, 128.7 (2C), 128.5, 128.3, 128.2, 128.1, 128.0, 127.9, 127.6, 127.2, 98.7 ($^{1}J_{C-H}$ = 164 Hz), 98.5 ($^{1}J_{C-H}$ = 168 Hz), 92.7, 80.4, 79.4, 79.0, 76.0, 75.3, 74.7, 73.9, 73.6, 73.5, 71.1, 69.6, 68.4, 63.2, 58.6, 55.5, 38.1, 29.9, 28.0; HRMS (ESI) calcd for $C_{48}H_{54}Cl_3NO_{13}Na$ $[M+Na]^+$ 980.2558; found: 980.2564.

Methyl 2-*O*-benzoyl-3,4-di-*O*-benzyl-α-D-mannopyranosyl-(1→4)-2,3,6-tri-*O*-benzyl-α-D-glucopyranoside (S17)

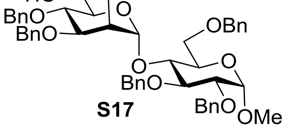

S17

Prepared from compounds **8** and **11**. $[\alpha]_D^{25}$ -3.44 (c 1.68, $CHCl_3$); ^{1}H NMR (400 MHz, $CDCl_3$) δ 8.02 – 7.91 (m, 2H), 7.62 – 7.51 (m, 1H), 7.44 (t, *J* = 7.7 Hz, 2H), 7.35 – 7.17 (m, 22H), 7.14 – 7.01 (m, 3H), 5.66 (t, *J* = 2.4 Hz, 1H), 5.50 (d, *J* = 1.9 Hz, 1H), 5.02 (d, *J* = 10.9 Hz, 1H), 4.86 (d, *J* = 10.9 Hz, 1H), 4.76 (d, *J* = 10.9 Hz, 1H), 4.73 –

4.67 (m, 2H), 4.61 – 4.55 (m, 3H), 4.53 (d, J = 1.5 Hz, 2H), 4.45 (d, J = 11.3 Hz, 1H), 4.01 – 3.91 (m, 2H), 3.87 (td, J = 9.2, 2.9 Hz, 2H), 3.76 – 3.60 (m, 6H), 3.53 (dd, J = 9.5, 3.5 Hz, 1H), 3.38 (s, 3H); ^{13}C NMR (101 MHz, $CDCl_3$) δ 165.4, 138.4, 138.3, 138.1, 138.0, 133.3, 130.0 (2C), 128.6, 128.5 (2C), 128.4 (2C), 128.3 (2C), 128.1, 128.0, 127.9, 127.7 (4C), 127.5, 99.0 ($^1J_{C-H}$ = 176 Hz), 98.0 ($^1J_{C-H}$ = 172 Hz), 81.9, 80.3, 78.2, 75.6, 75.4, 75.0, 74.0, 73.7, 73.4, 72.9, 71.6, 69.8, 69.3, 68.8, 62.0, 55.5; HRMS (ESI) calcd for $C_{55}H_{58}O_{12}Na$ $[M+Na]^+$ 933.3826; found: 933.3833.

Methyl 2-*O*-benzoyl-4-*O*-benzyl-α-L-rhamnopyranosyl-(1→4)-2,3,6-tri-*O*-benzyl-α-D-glucopyranoside (S18)

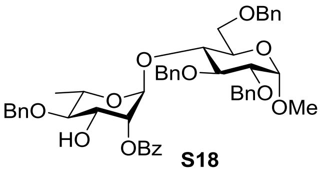

Prepared from compounds **9** and **11**. $[\alpha]_D^{25}$ +17.2 (c 1.26, $CHCl_3$); ^{1}H NMR (400 MHz, $CDCl_3$) δ 7.98 (dd, J = 8.3, 1.4 Hz, 2H), 7.59 – 7.53 (m, 1H), 7.46 – 7.41 (m, 2H), 7.35 – 7.12 (m, 20H), 5.23 (dd, J = 3.4, 1.7 Hz, 1H), 5.09 – 5.03 (m, 2H), 4.75 – 4.69 (m, 3H), 4.66 (d, J = 11.3 Hz, 1H), 4.60 – 4.52 (m, 3H), 4.49 (d, J = 11.9 Hz, 1H), 4.14 (dd, J = 9.5, 3.4 Hz, 1H), 3.99 (dd, J = 9.6, 6.2 Hz, 1H), 3.92 – 3.76 (m, 3H), 3.73 – 3.64 (m, 2H), 3.58 (dd, J = 9.2, 3.6 Hz, 1H), 3.42 – 3.36 (m, 1H), 3.35 (s, 3H), 0.97 (d, J = 6.2 Hz, 3H); ^{13}C NMR (101 MHz, $CDCl_3$) δ 166.4, 138.9, 138.4, 138.1, 137.9, 133.5, 130.0, 129.9, 128.6 (2C), 128.5, 128.4, 128.3 (2C), 128.2, 128.1 (2C), 128.0 (3C), 127.6, 127.4, 98.1 ($^1J_{C-H}$ = 172 Hz), 97.2 ($^1J_{C-H}$ = 176 Hz), 82.0, 80.5, 79.9, 75.7, 75.0, 74.8, 73.7, 73.5, 73.4, 70.2, 70.1, 68.5, 68.1, 55.4, 18.0; HRMS (ESI) calcd for $C_{48}H_{52}O_{11}Na$ $[M+Na]^+$ 827.3407; found: 827.3409.

Methyl (benzyl 3,4-di-*O*-benzyl-2-*O*-levulinoyl-β-D-glucopyranosyluronate)-(1→4)-2,3,6-tri-*O*-benzyl-α-D-glucopyranoside (S19)

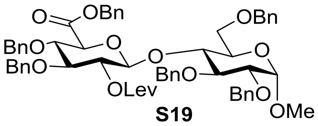

Prepared from compounds **10** and **11**. $[\alpha]_D^{25}$ -7.40 (c 0.84, $CHCl_3$); ^1H NMR (400 MHz, $CDCl_3$) δ 7.45 – 7.40 (m, 2H), 7.36 – 7.25 (m, 26H), 7.15 – 7.10 (m, 2H), 5.08 – 4.94 (m, 4H), 4.80 – 4.69 (m, 4H), 4.67 – 4.58 (m, 4H), 4.50 (d, *J* = 8.1 Hz, 1H), 4.46 – 4.40 (m, 2H), 3.96 – 3.77 (m, 5H), 3.71 (dt, *J* = 9.8, 1.8 Hz, 1H), 3.64 (dd, *J* = 10.9, 1.9 Hz, 1H), 3.50 (dd, *J* = 9.2, 3.7 Hz, 1H), 3.43 (t, *J* = 9.1 Hz, 1H), 3.40 (s, 3H), 2.79 – 2.68 (m, 1H), 2.65 – 2.54 (m, 1H), 2.48 – 2.37 (m, 1H), 2.33 – 2.25 (m, 1H), 2.15 (s, 3H); ^{13}C NMR (101 MHz, $CDCl_3$) δ 206.1, 171.2, 168.0, 139.5, 138.5, 138.2, 137.9, 137.8, 135.2, 128.8, 128.7 (2C), 128.6 (2C), 128.5, 128.4 (2C), 128.3, 128.2 (2C), 128.1 (3C), 127.9, 127.8 (2C), 127.7, 127.2, 100.6 ($^1J_{C\text{-}H}$ = 164 Hz), 98.5 ($^1J_{C\text{-}H}$ = 172 Hz), 82.1, 80.2, 79.7, 79.1, 77.3, 75.6, 75.0, 74.9, 74.6, 73.8, 73.7, 73.5, 69.7, 67.9, 67.3, 55.4, 37.8, 30.0, 27.9; HRMS (ESI) calcd for $C_{60}H_{64}O_{14}Na$ $[M+Na]^+$ 1031.4194; found: 1031.4199.

1,2-*Cis* glycosylation

Table S2: 1,2-*Cis*-glycosylation activated by DBDMH

Entry	Donor	Acceptor	Solvent	T (°C)	Product	Yield (%)	α/β ratio
1	**12**	**2**	DCM/Et_2O	-78	**S20**	94	1:1.4
2	**12**	**2**	DCM	-78	**S20**	94	1:2.7
3	**12**	**2**	DCM/MeCN	-78	**S20**	93	1:11.7
4	**12**	**2**	DCM	-40	**S20**	67	1:1.3
5	**13**	**2**	DCM	-78	**S21**	72	4.6:1
6	**13**	**2**	DCM	-40	**S21**	50	11.8:1
7	**14**	**2**	DCM	-78	**S22**	76	1:1.1
8	**14**	**2**	DCM	-40	**S22**	69	1:1

Methyl 2,3,4,6-tetra-*O*-benzyl-D-galactopyranosyl-(1→6)-2,3,4-tri-*O*-benzyl-α-D-glucopyranoside (S20)

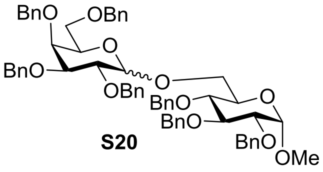

S20

Purification: flash column chromatography with 15% ethyl acetate in hexanes. ^1H NMR (400 MHz, $CDCl_3$) δ 7.41 – 7.19 (m, 35H), 5.04 – 4.93 (m, 3H), 4.89 (d, *J* = 12.0 Hz, 0.5H), 4.85 – 4.66 (m, 6.5H), 4.64 – 4.51 (m, 3.5H), 4.49 – 4.37 (m, 2H),

4.34 (d, J = 7.7 Hz, 0.5H), 4.18 (dd, J = 10.8, 2.0 Hz, 0.5H), 4.04 – 3.73 (m, 6H), 3.67 – 3.42 (m, 5.5H), 3.33 (d, J = 1.9 Hz, 3H); ^{13}C NMR (101 MHz, $CDCl_3$) δ 139.0, 138.9, 138.6, 138.5, 138.3 (2C), 138.2, 138.0, 128.6, 128.5 (4C), 128.4 (3C), 128.3 (2C), 128.1 (2C), 128.0 (2C), 127.9, 127.8 (3C), 127.7, 127.6 (2C), 127.5, 104.3 ($^1J_{C\text{-}H}$ = 160 Hz), 98.1 ($^1J_{C\text{-}H}$ = 168 Hz), 98.0 (2C, $^1J_{C\text{-}H}$ = 172 Hz), 82.4, 82.2, 82.1, 80.3, 80.0, 79.4, 78.4, 78.2, 78.1, 76.6, 75.8 (2C), 75.3, 75.2, 75.0, 74.9, 74.7, 73.6 (2C), 73.5 (2C), 73.0, 72.9, 72.7, 70.4, 70.0, 69.5, 69.0, 68.7 (2C), 66.5, 55.3, 55.2; HRMS (ESI) calcd for $C_{62}H_{66}O_{11}Na$ $[M+Na]^+$ 1009.4503; found: 1009.4510.

Methyl 4-*O*-benzoyl-2,3,6-tri-*O*-benzyl-D-galactopyranosyl-(1→6)-2,3,4-tri-*O*-benzyl-α-D-glucopyranoside (S21)

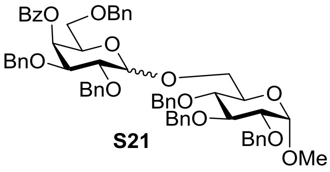

S21

Purification: flash column chromatography with 17% ethyl acetate in hexanes. ^{1}H NMR (400 MHz, $CDCl_3$) δ 8.11 – 7.94 (m, 2H), 7.56 (t, J = 7.4 Hz, 1H), 7.51 – 7.02 (m, 32H), 5.79 (d, J = 3.5 Hz, 1H), 5.06 (d, J = 3.5 Hz, 1H), 4.96 (d, J = 10.7 Hz, 1H), 4.85 – 4.42 (m, 11H), 4.40 – 4.30 (m, 1H), 4.17 (t, J = 6.5 Hz, 1H), 4.05 – 3.93 (m, 2H), 3.88 (dd, J = 10.0, 3.5 Hz, 1H), 3.82 – 3.67 (m, 3H), 3.62 – 3.39 (m, 4H), 3.34 – 3.25 (m, 3H); ^{13}C NMR (101 MHz, $CDCl_3$) δ 165.9, 139.0, 138.8, 138.5, 138.3, 138.2, 137.9, 133.1, 130.2, 130.0, 128.6 (2C), 128.5 (2C), 128.4 (3C), 128.3 (2C), 128.2, 128.1 (3C), 128.0, 127.9 (2C), 127.8, 127.7 (2C), 127.6, 127.5, 104.5 ($^1J_{C\text{-}H}$ = 160 Hz), 98.2 ($^1J_{C\text{-}H}$ = 168 Hz), 98.1 ($^1J_{C\text{-}H}$ = 172 Hz), 98.0 ($^1J_{C\text{-}H}$ = 172 Hz), 82.2, 80.2, 79.9, 79.6, 78.8, 78.1, 78.0, 75.8, 75.6, 75.4, 75.1, 75.0, 73.8, 73.6, 73.5, 73.0, 72.5, 72.0, 71.6, 70.4, 70.1, 68.8, 68.7, 68.3, 68.1, 67.4, 66.4, 55.4, 55.2; HRMS (ESI) calcd for $C_{62}H_{64}O_{12}Na$ $[M+Na]^+$ 1023.4295; found: 1023.4296.

Methyl 4,6-di-*O*-benzoyl-2,3-di-*O*-benzyl-D-glucopyranosyl-(1→6)-2,3,4-tri-*O*-benzyl-α-D-glucopyranoside (S22)

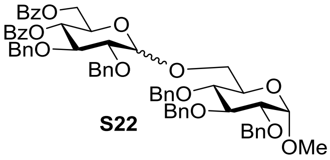

S22

Purification: flash column chromatography with 18% ethyl acetate in hexanes. ^{1}H NMR (400 MHz, $CDCl_3$) δ 8.04 – 7.92 (m, 4H), 7.62 – 7.50 (m, 2H), 7.45 – 7.25 (m, 23H), 7.21 (dd, *J* = 7.6, 1.9 Hz, 1H), 7.16 – 7.07 (m, 5H), 5.49 – 5.35 (m, 1H), 5.04 – 4.94 (m, 2H), 4.87 – 4.59 (m, 9H), 4.58 – 4.47 (m, 2H), 4.39 (dd, *J* = 12.1, 5.4 Hz, 1H), 4.28 – 4.22 (m, 1H), 4.19 (dd, *J* = 8.0, 2.0 Hz, 0.5H), 4.02 (t, *J* = 8.0 Hz, 0.5H), 4.02 (td, *J* = 9.3, 3.6 Hz, 1H), 3.90 – 3.65 (m, 4.5H), 3.61 – 3.50 (m, 1H), 3.43 – 3.38 (m, 2H), 3.35 (s, 1.5H); ^{13}C NMR (101 MHz, CDCl3) δ 166.3, 165.4, 165.3, 138.9 (2C), 138.5 (2C), 138.2 (2C), 138.0, 137.8, 133.4, 133.3, 133.1, 130.0, 129.9 (2C), 129.8, 129.7, 129.6, 128.6, 128.5 (2C), 128.4 (2C), 128.3 (3C), 128.2, 128.1 (2C), 128.0 (2C), 127.9 (2C), 127.8 (2C), 127.7 (2C), 127.6, 104.0 ($^{1}J_{C\text{-}H}$ = 160 Hz), 98.2 ($^{1}J_{C\text{-}H}$ = 172 Hz), 98.0 ($^{1}J_{C\text{-}H}$ = 172 Hz), 97.1 ($^{1}J_{C\text{-}H}$ = 172 Hz), 82.2, 82.1, 82.0, 81.6, 80.2, 80.0, 79.9, 78.2, 78.0, 75.9, 75.8, 75.4, 75.3, 75.2, 75.1, 75.0, 73.5, 73.4, 72.9, 72.0, 71.2, 70.9, 70.4, 69.8, 68.9, 67.9, 66.3, 63.6, 63.3, 55.4, 55.2; HRMS (ESI) calcd for $C_{62}H_{62}O_{13}Na$ $[M+Na]^{+}$ 1037.4088; found: 1037.4095.

Solid-phase automated glycan assembly

Building block solution: The building block **8** was co-evaporated with toluene three times, dissolved in DCM under argon and transferred to the vial which was placed on the corresponding port in the synthesizer. For each glycosylation, building block **8** (6.5 equivalents, 0.08 mmol) was dissolved in 1 mL DCM.

Acidic TMSOTf wash solution: 450 µL TMSOTf was dissolved in 40 mL DCM.

Activator solution: DBDMH (0.86 g) was dissolved in a 2:1 (v/v) mixture of anhydrous DCM and dioxane (40 mL) followed by the addition of TMSOTf (54 µL).

Fmoc deprotection solution: The solution was 20% (v/v) piperidine in DMF.

Preparation of the resin: The functionalized resin **15** [11] (40 mg, 0.0125 mmol linker) was loaded into the reaction vessel of the synthesizer and swollen in 2 mL DCM for 20 min.

Module a-Acidic TMSOTf wash: The resin is washed with 1 mL acidic solution of TMSOTf in DCM for three minutes at -20 ^{o}C.

Module b-Glycosylation using thioglycoside: For glycosylation the acidic solution is drained and thioglycoside building block (1 mL) is delivered to the reaction vessel. After the set temperature −20 °C is reached, the reaction starts with the addition of 1 mL of activator solution. The glycosylation is performed for 5 min at −20 °C and 20 min from −20 °C to 0 °C Then the solution is drained and the resin is washed with dioxane (2 mL for 20 s) and DCM (two times each with 2 mL for 25 s). The temperature of the reaction vessel is increased to 25 °C for the next module.

Module c-Fmoc deprotection: The resin is washed with DMF and then 2 mL solution of 20% piperidine in DMF for 5 min. The reaction solution was drained and the resin was washed with DMF (three times with 3 mL for 25 s) and DCM (five times each with 2 mL for 25 s).

Cleavage from solid support: After automated synthesis, the oligosaccharides were cleaved from the solid support using a continuous-flow photoreactor as described previously [11].

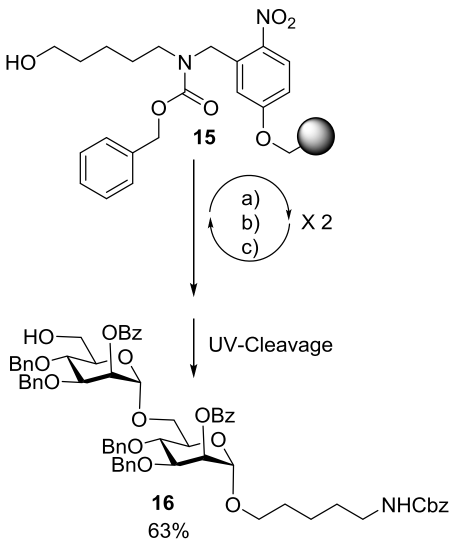

Modules: a) acidic wash; b) glycosylation using DBDMH/TMSOTf, **8**; c) Fmoc deprotection

Analytical HPLC of the crude after UV-cleavage: YMC-Diol-300-NP 5 µm (150 × 4.60 mm) column; flow rate: 1.0 mL; elution gradient: 20% ethyl acetate in hexanes for 5 min, increased to 55% at 40 min, then 100% at 45 min; detection: ELSD and UV light.

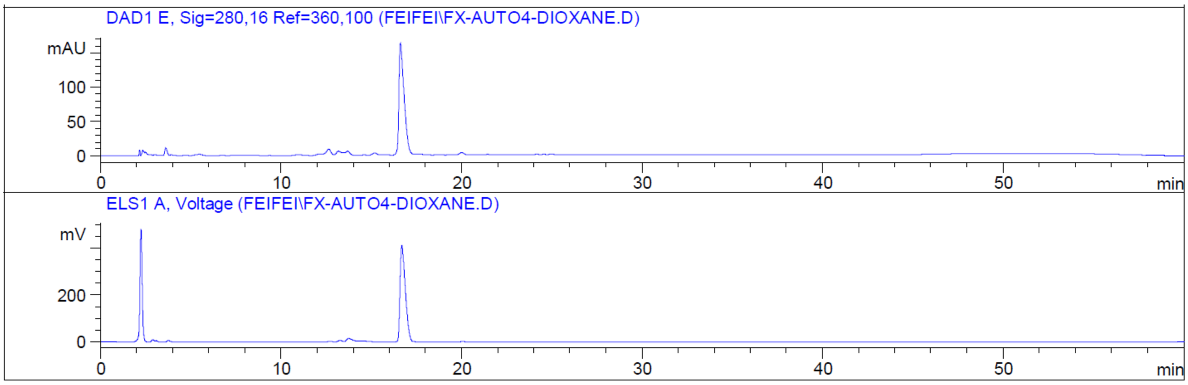

Purification: flash column chromatography with 30% ethyl acetate in hexanes

***N*-benzyloxycarbonyl-5-aminopentanyl 2-*O*-benzoyl-3,4-di-*O*-benzyl-α-D-mannopyranosyl-(1→6)-2-*O*-benzoyl-3,4-di-*O*-benzyl-α-D-mannopyranoside (16)**

$[\alpha]_D^{25}$ +9.10 (c 0.91, $CHCl_3$); ^{1}H NMR (400 MHz, $CDCl_3$) δ 8.12 (dd, *J* = 8.1, 1.7 Hz, 2H), 8.07 (dd, *J* = 8.3, 1.4 Hz, 2H), 7.62 – 7.56 (m, 1H), 7.54 – 7.44 (m, 5H), 7.33 – 7.21 (m, 23H), 7.17 – 7.11 (m, 2H), 5.73 (dd, *J* = 3.2, 1.9 Hz, 1H), 5.62 (dd, *J* = 3.3, 1.8 Hz, 1H), 5.16 – 5.05 (m, 3H), 4.94 – 4.80 (m, 5H), 4.74 (d, *J* = 11.4 Hz, 1H), 4.63 (d, *J* = 11.1 Hz, 1H), 4.56 (d, *J* = 11.0 Hz, 1H), 4.49 (d, *J* = 11.2 Hz, 1H), 4.47 (d, *J* = 11.2 Hz, 1H), 4.09 (dd, *J* = 9.4, 3.1 Hz, 2H), 4.01 – 3.60 (m, 10H), 3.47 – 3.38 (m, 1H), 3.18 (q, *J* = 7.0 Hz, 2H), 1.59 – 1.54 (m, 2H), 1.54 – 1.47 (m, 2H), 1.40 – 1.34 (m, 2H); ^{13}C NMR (101 MHz, $CDCl_3$) δ 166.0, 165.7, 138.4 (2C), 138.0, 137.8, 133.4, 130.0 (2C), 129.9, 128.7, 128.6 (2C), 128.5 (2C), 128.4, 128.3, 128.2 (3C), 128.1, 127.9, 127.8 (3C), 98.0, 97.9, 78.7, 78.0, 75.3, 74.4, 74.0, 72.2, 71.8, 71.5, 70.7, 69.1, 68.9, 67.9, 66.7, 66.3, 62.1, 41.1, 29.9, 29.2, 23.6; HRMS (ESI) calcd for $C_{67}H_{71}O_{15}NNa$ $[M+Na]^+$ 1152.4721; found: 1152.4727.

Hydrolysis of glycosyl selenide

Phenyl 2-azido-2-deoxy-4-*O*-fluorenylmethoxycarbonyl-6-*O*-levulinoyl-3-*O*-(2-naphthalenylmethyl)-1-seleno-α-D-galactopyranoside (17)

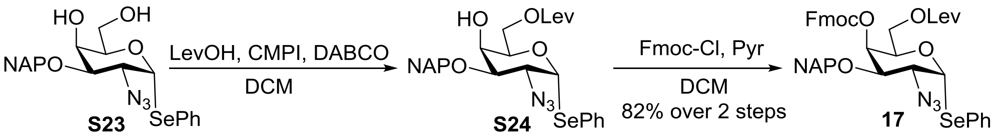

To a solution of phenyl 2-azido-2-deoxy-3-*O*-(2-naphthalenylmethyl)-1-seleno-α-D-galactopyranoside [12] (**S23**, 280 mg, 0.58 mmol) in DCM (4 mL) was added levulinic acid (0.35 mL, 3.47 mmol) followed by 2-chloro-1-methylpyridinium iodide (CMPI, 369 mg, 1.44 mmol). 1,4-Diazabicyclo[2.2.2]octane (DABCO, 246 mg, 2.2 mmol) was added after 15 min. The reaction mixture was stirred at room temperature for 24 h and then diluted with ethyl acetate, filtered through Celite and concentrated. The residue was dissolved in DCM (5 mL), followed by the addition of 9-fluorenylmethyl chloroformate (299 mg, 1.16 mmol) and pyridine (0.14 mL, 1.74 mmol). The mixture was stirred overnight at room temperature. Then the reaction was quench with 1 M aq HCl solution and diluted with DCM. The organic layer was dried over Na_2SO_4, filtered and concentrated. The residue was purified by flash column chromatography to afford **17** (380 mg, 0.47 mmol, 82%) as slightly yellow foam.

$[\alpha]_D^{25}$ +157.77 (c 1.26, $CHCl_3$); ^{1}H NMR (400 MHz, $CDCl_3$) δ 7.83 (d, *J* = 1.6 Hz, 1H), 7.79 – 7.69 (m, 5H), 7.68 – 7.62 (m, 2H), 7.57 (d, *J* = 7.5 Hz, 1H), 7.50 (dd, *J* = 8.4, 1.7 Hz, 1H), 7.47 – 7.38 (m, 4H), 7.35 – 7.28 (m, 5H), 7.23 (td, *J* = 7.5, 1.1 Hz, 1H), 6.03 (d, *J* = 5.4 Hz, 1H), 5.54 (dd, *J* = 3.2, 1.2 Hz, 1H), 4.98 (d, *J* = 10.9 Hz, 1H), 4.81 (d, *J* = 10.9 Hz, 1H), 4.68 (t, *J* = 6.5 Hz, 1H), 4.42 (dd, *J* = 10.4, 7.1 Hz, 1H), 4.36 – 4.26 (m, 2H), 4.21 (dd, *J* = 6.5, 1.6 Hz, 2H), 4.14 (t, *J* = 7.5 Hz, 1H), 3.90 (dd, *J* = 10.3, 3.1 Hz, 1H), 2.74 (t, *J* = 6.5 Hz, 2H), 2.55 (t, *J* = 6.2 Hz, 2H), 2.20 (s, 3H); ^{13}C NMR (101 MHz, $CDCl_3$) δ 206.6, 172.4, 154.9, 143.5, 143.0, 141.3, 141.2, 134.8, 134.3, 133.2, 133.1, 129.3, 128.3 (2C), 128.0, 127.9, 127.8, 127.7, 127.4, 127.2 (2C), 126.1 (3C), 125.4, 125.1, 120.1, 120.0, 84.6, 72.2, 70.5, 70.3, 69.1, 62.1, 60.5, 46.5, 38.0, 30.0, 27.8; HRMS (ESI) calcd for $C_{43}H_{39}N_3O_8SeNa$ $[M+Na]^+$ 828.1795; found: 828.1811.

_N_-Phenyltrifluoroacetimidate 2-azido-2-deoxy-4-_O_-(9-fluorenylmethoxycarbonyl)-6-_O_-levulinoyl-3-_O_-(2-naphthalenylmethyl)-α-D-galactopyranoside (19)

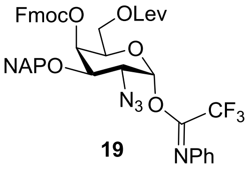

19

To a solution of monosaccharide **17** (80 mg, 0.099 mmol) in THF/water (4.2 mL, 5:1 v/v) was added DBDMH (45.5 mg, 0.16 mmol) at 0 °C. The reaction mixture was stirred at room temperature for 2 h. Then the mixture was diluted with DCM and washed with 10% aq $Na_2S_2O_3$ solution. The organic layer was dried over Na_2SO_4, filtered and concentrated to get crude **18**. The residue **18** was dissolved in DCM (4 mL) followed by the addition of (*E*)-2,2,2-trifluoro-*N*-phenylacetimidoyl chloride (47 µL, 0.298 mmol) and Cs_2CO_3 (97 mg, 0.298 mmol) at 0 °C . After 2 h, the reaction mixture was filtered and concentrated. The residue was purified by flash column chromatography to give imidate **19** (70 mg, 0.083 mmol, 84%) as colorless oil.

^{1}H NMR (400 MHz, $CDCl_3$) δ 7.85 – 7.66 (m, 6H), 7.60 (d, *J* = 7.5 Hz, 1H), 7.52 – 7.22 (m, 10H), 7.11 (t, *J* = 7.4 Hz, 1H), 6.83 (d, *J* = 7.9 Hz, 2H), 5.50 (brs, 1H), 5.42 (s, 1H), 4.94 (d, *J* = 11.5 Hz, 1H), 4.76 (d, *J* = 11.4 Hz, 1H), 4.47 – 4.15 (m, 5H), 3.99 (t, *J* = 9.4 Hz, 1H), 3.84 (s, 1H), 3.57 (d, *J* = 10.2 Hz, 1H), 2.80 – 2.41 (m, 4H), 2.12 (s, 3H); ^{13}C NMR (101 MHz, $CDCl_3$) δ 206.6, 172.3, 155.0, 143.5, 143.2, 143.1, 141.4, 141.3, 134.2, 133.2, 129.0, 128.4, 128.0, 127.9, 127.8, 127.5, 127.3 (3C), 126.2, 126.1, 125.4, 125.2, 124.7, 120.1 (2C), 119.3, 95.4, 77.6, 72.3, 71.6, 70.7, 69.2, 61.7, 61.5, 46.6, 38.0, 29.8, 28.0; HRMS (ESI) calcd for $C_{45}H_{39}F_3N_4O_9Na$ $[M+Na]^+$ 859.2561; found: 859.2566.

α/β selectivity determined using SFC

Supercritical fluid chromatography (SFC) was used to separate α/β mixture to determine α/β ratio. (Silica-2EP analytical column; Solvent: *iso*-propanol)

S20 (DCM-Et_2O, −78 ^oC)

Peak No	% Area	Area	Ret. Time	Height	Cap. Factor
1	41.158	211.8201	8.97 min	12.2529	8969
2	58.842	302.8305	10.48 min	19.9805	10475.6667

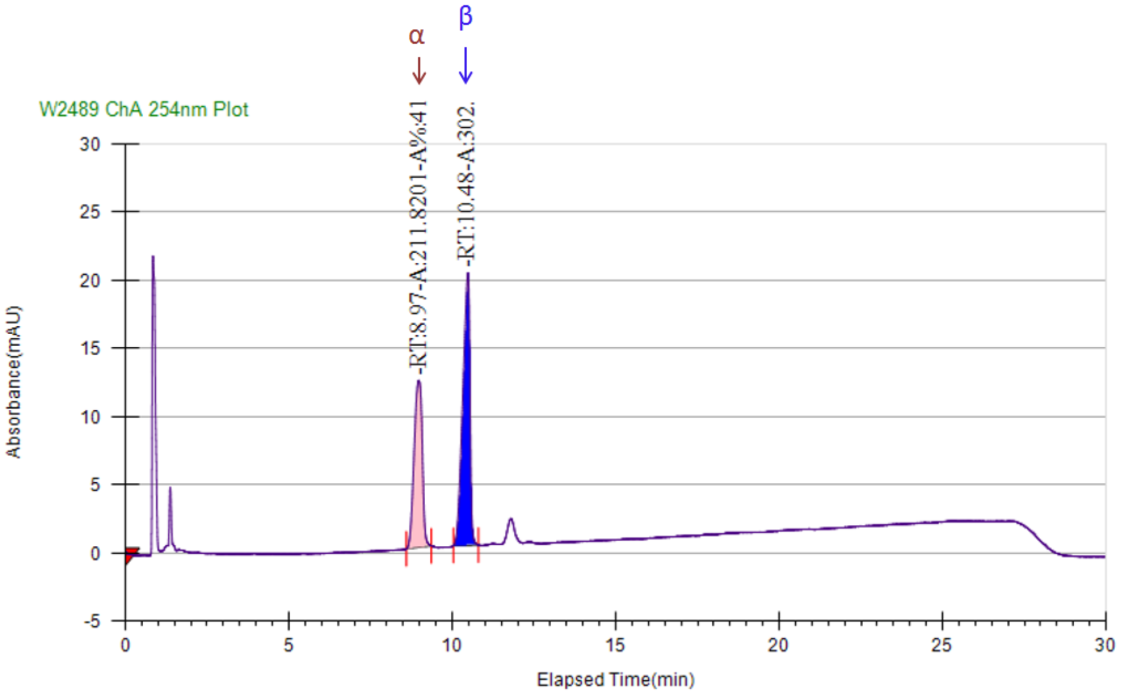

S20 (DCM, −-78 ^oC)

Peak No	% Area	Area	Ret. Time	Height	Cap. Factor
1	26.8587	69.5175	8.94 min	4.6946	8935.6667
2	73.1413	189.3094	10.43 min	14.213	10429

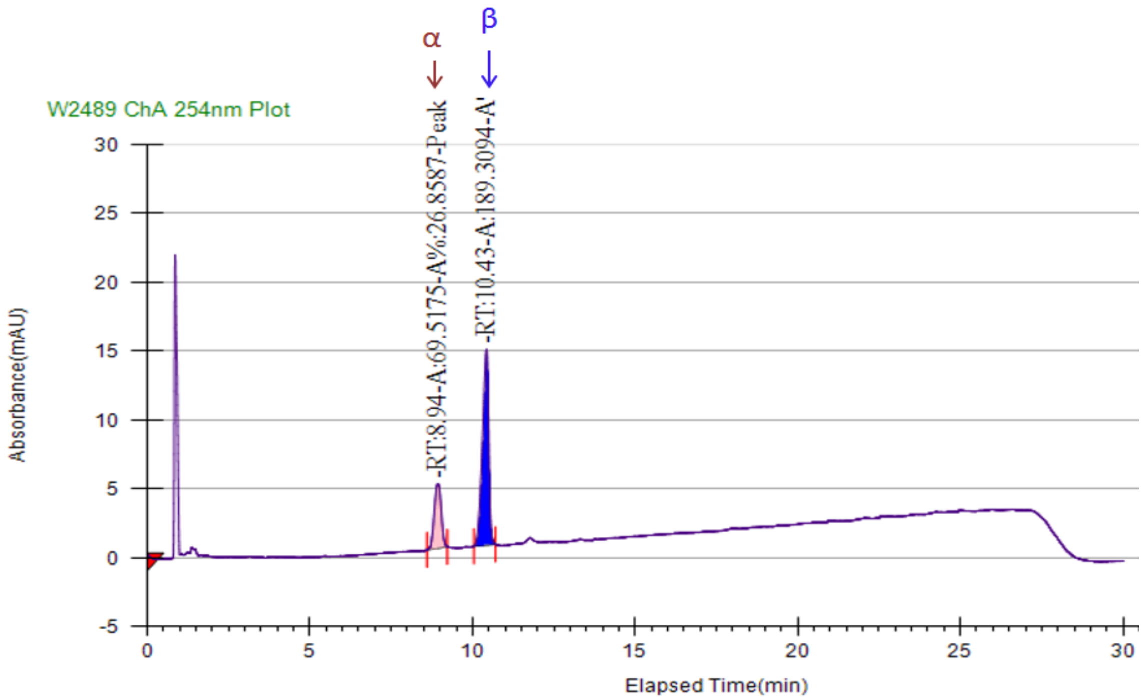

S20 (DCM-MeCN, −78 ^oC)

Peak No	% Area	Area	Ret. Time	Height	Cap. Factor
1	7.9	38.15	8.93 min	2.269	8929
2	92.1	444.7635	10.54 min	27.0358	10542.3333

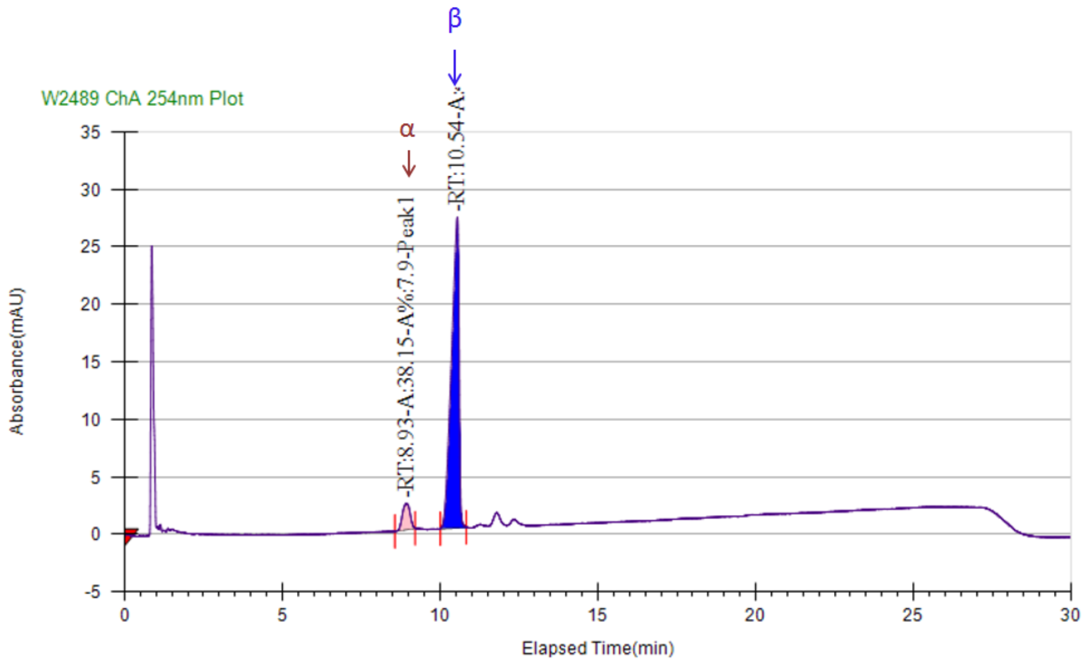

S20 (DCM, −40 °C)

Peak No	% Area	Area	Ret. Time	Height	Cap. Factor
1	43.5602	82.394	8.94 min	5.9707	8942.3333
2	56.4398	106.7559	10.37 min	7.7726	10372.3333

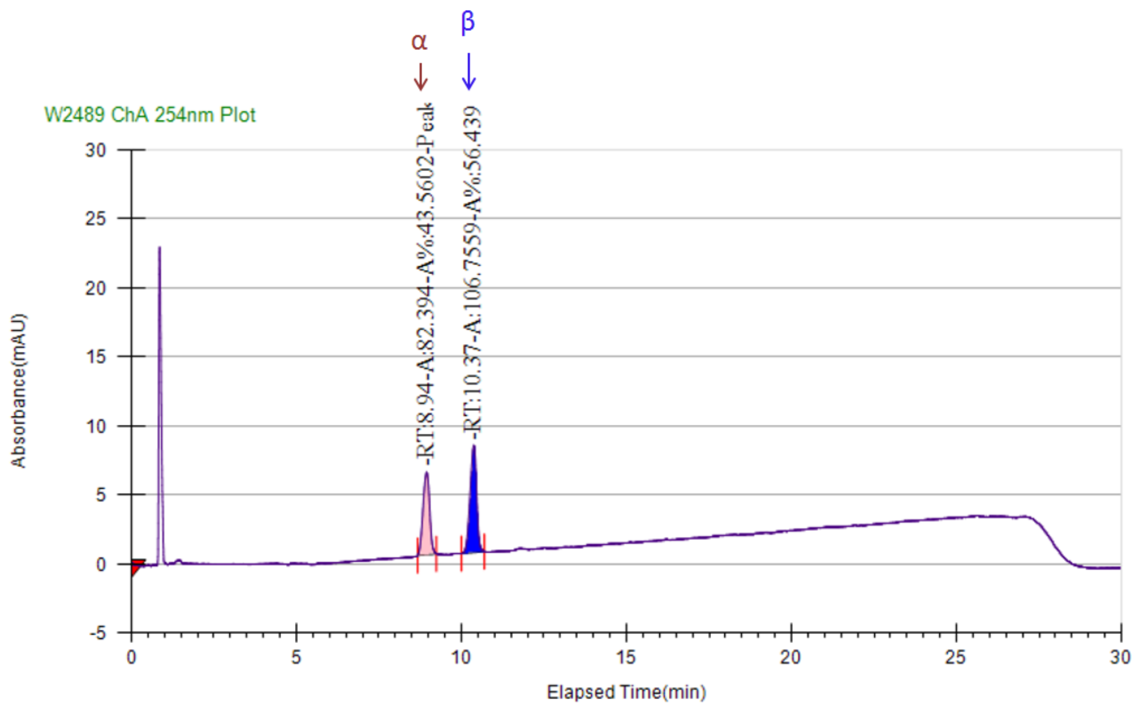

S21 (DCM, −78 °C)

Peak No	% Area	Area	Ret. Time	Height	Cap. Factor
1	82.1556	418.4055	9.75 min	28.4306	9745.6667
2	17.8444	90.8787	10.46 min	7.1931	10462.3333

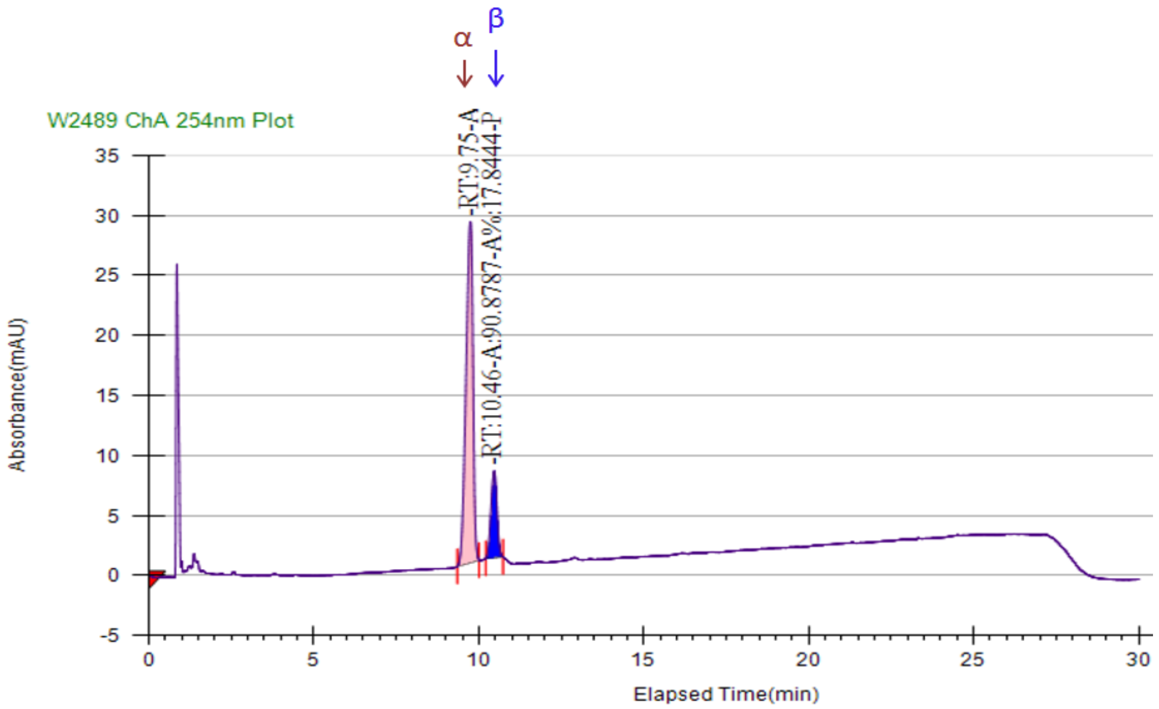

S21 (DCM, −40 ^{o}C)

Peak No	% Area	Area	Ret. Time	Height	Cap. Factor
1	92.215	356.0052	9.73 min	24.1167	9732.3333
2	7.785	30.0547	10.44 min	2.3254	10442.3333

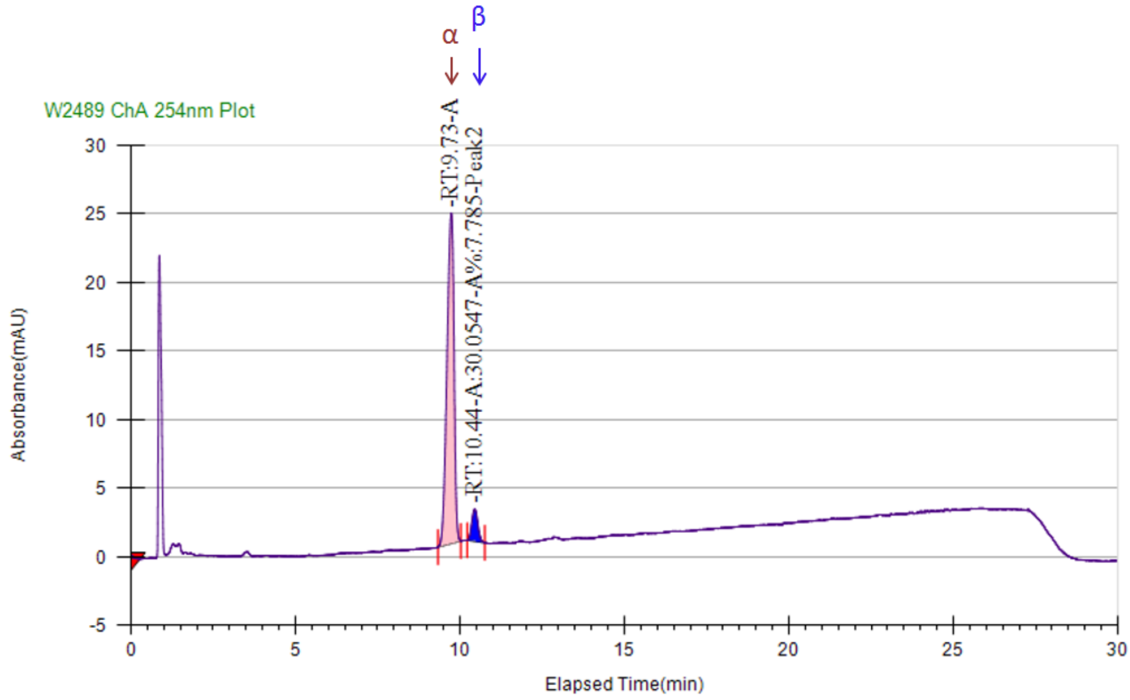

S22 (DCM, −78 °C)

Peak No	% Area	Area	Ret. Time	Height	Cap. Factor
1	47.467	314.8099	10.03 min	23.3808	10025.6667
2	52.533	348.4087	11.1 min	26.9942	11095.6667

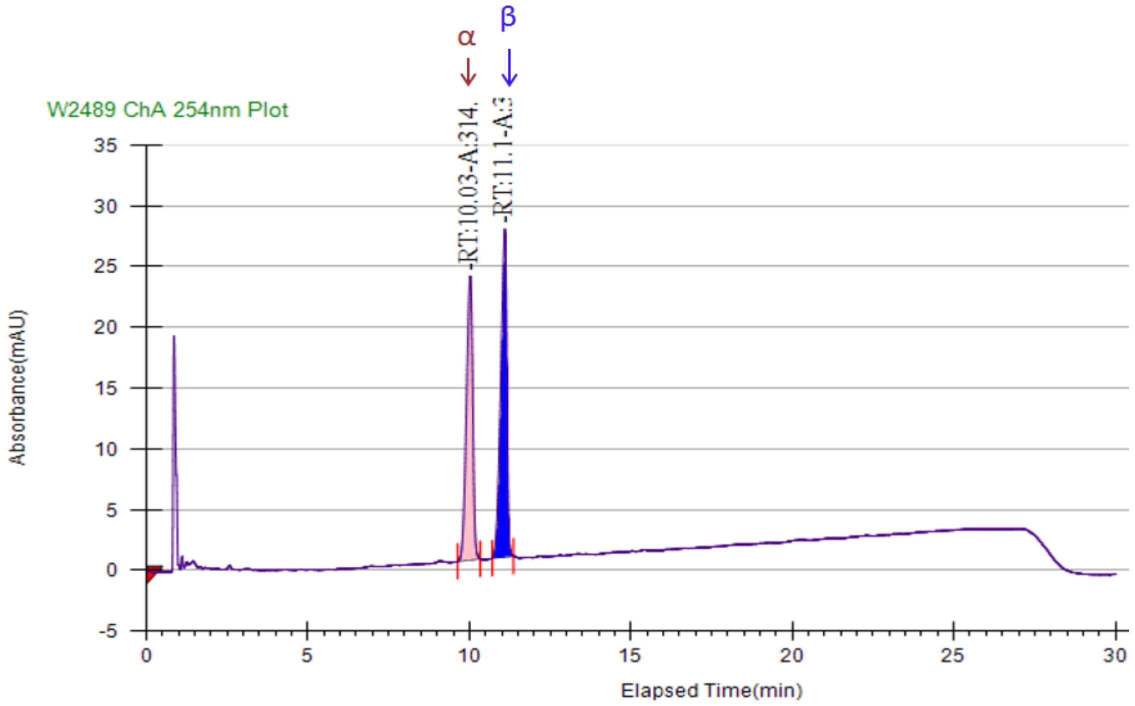

S22 (DCM, −40 °C)

Peak No	% Area	Area	Ret. Time	Height	Cap. Factor
1	48.9825	191.9155	10 min	14.3455	10002.3333
2	51.0175	199.8891	11.04 min	15.4569	11039

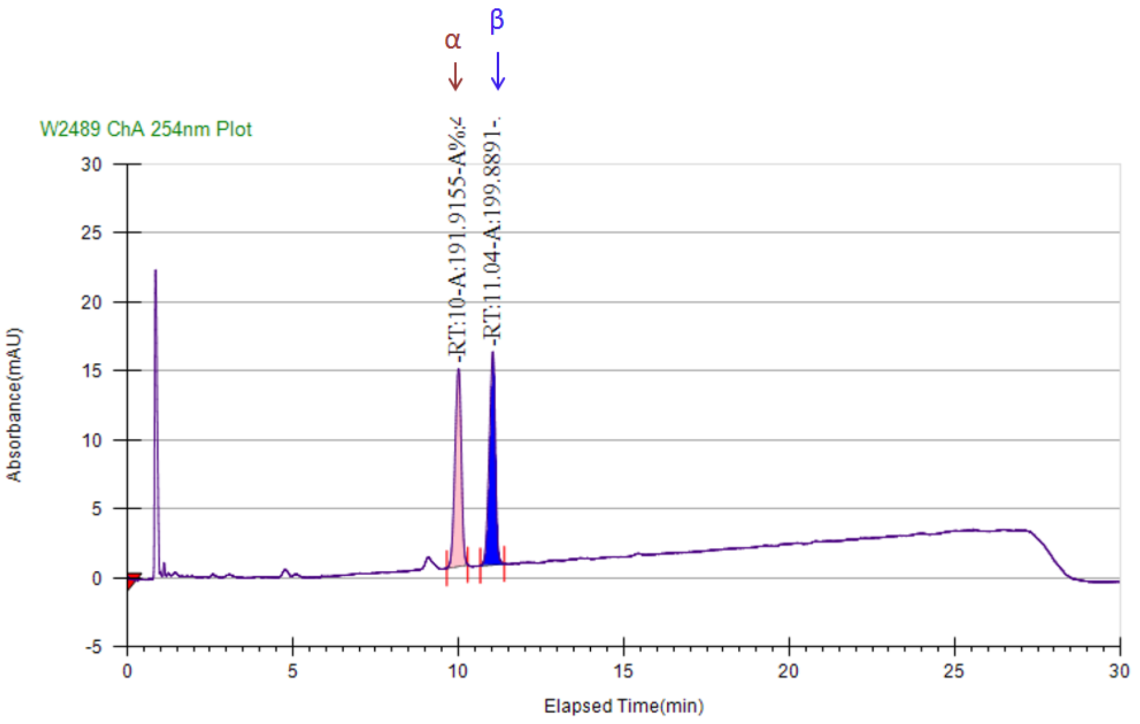

NMR spectra

Ethyl 2-*O*-benzoyl-4,6-di-*O*-benzyl-3-*O*-(9-fluorenylmethoxycarbonyl)-1-thio-β-D-galactopyranoside (1)

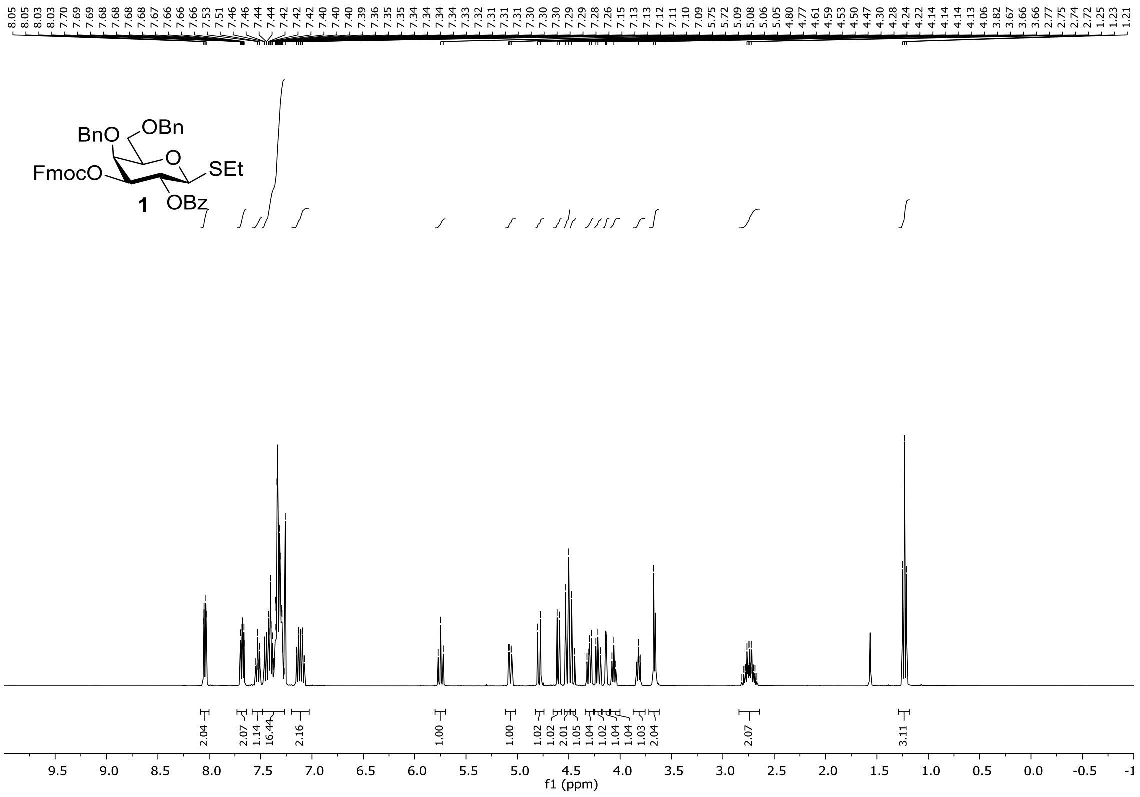

Methyl 2,3,4-tri-*O*-benzyl-α-D-glucopyranoside (2)

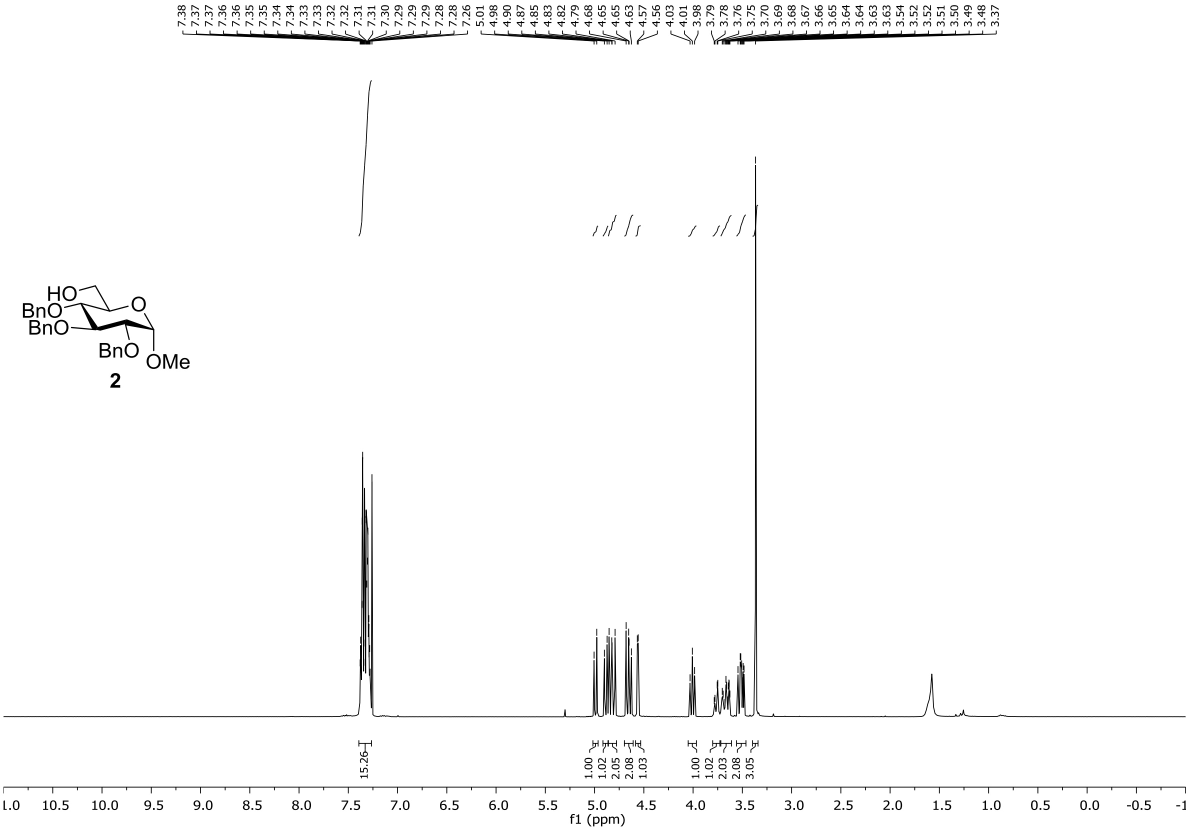

Ethyl 2,3-di-*O*-benzoyl-4,6-*O*-benzylidene-1-thio-β-D-galactopyranoside (4)

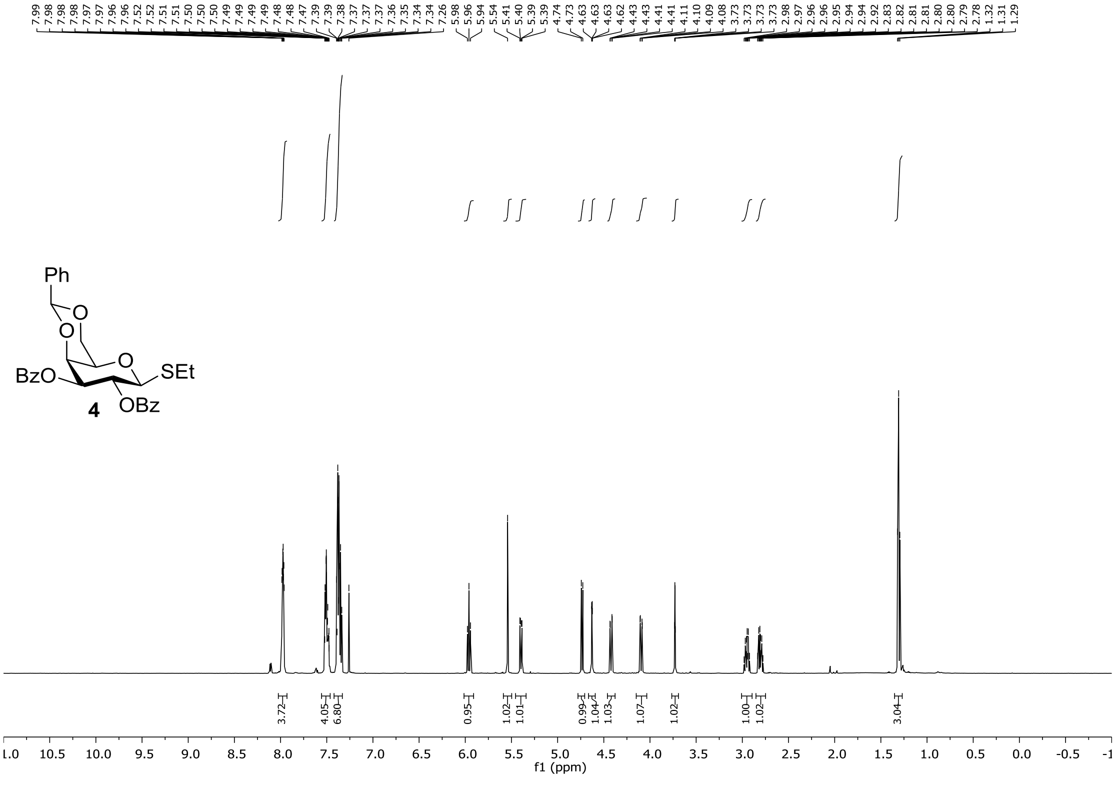

Ethyl 2,3-di-*O*-benzoyl-6-*O*-benzyl-4-*O*-(9-fluorenylmethoxycarbonyl)-1-thio-β-D-glucopyranoside (5)

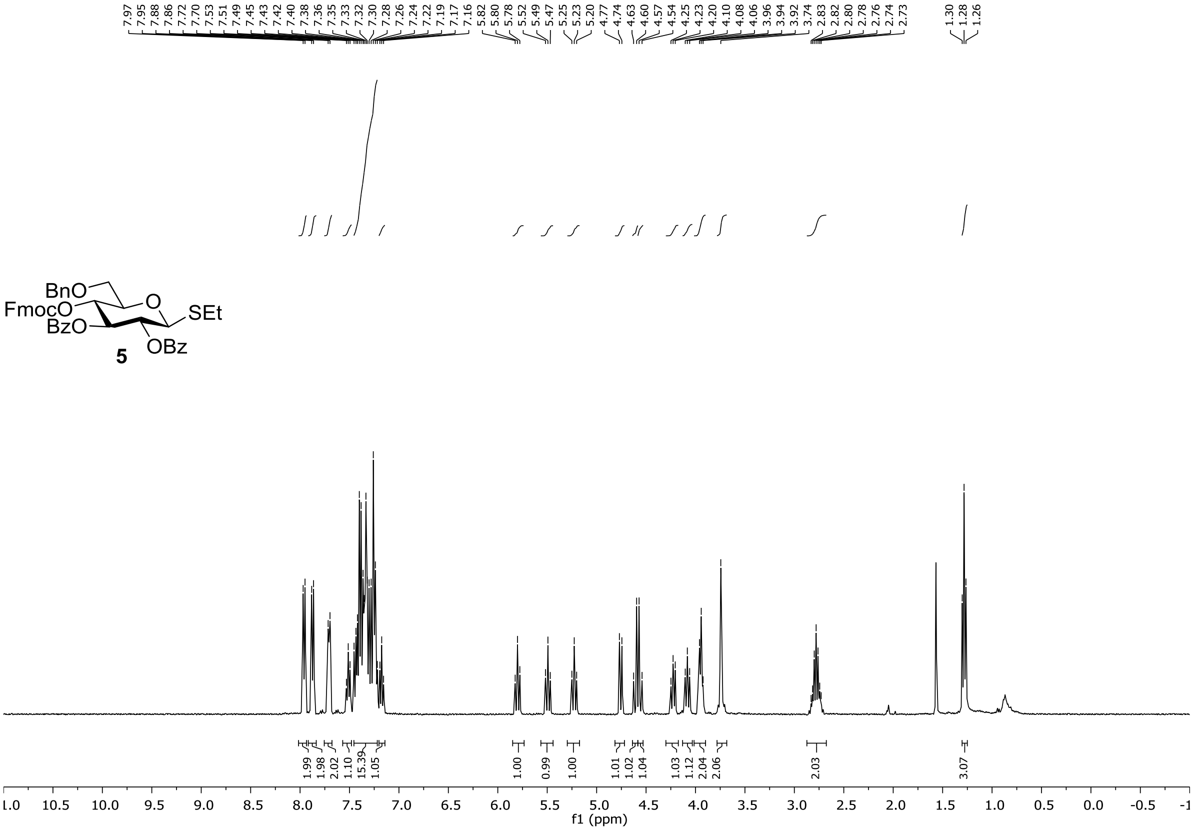

Ethyl 3,4,6-tri-*O*-benzyl-2-*O*-levulinoyl-1-thio-β-D-glucopyranoside (6)

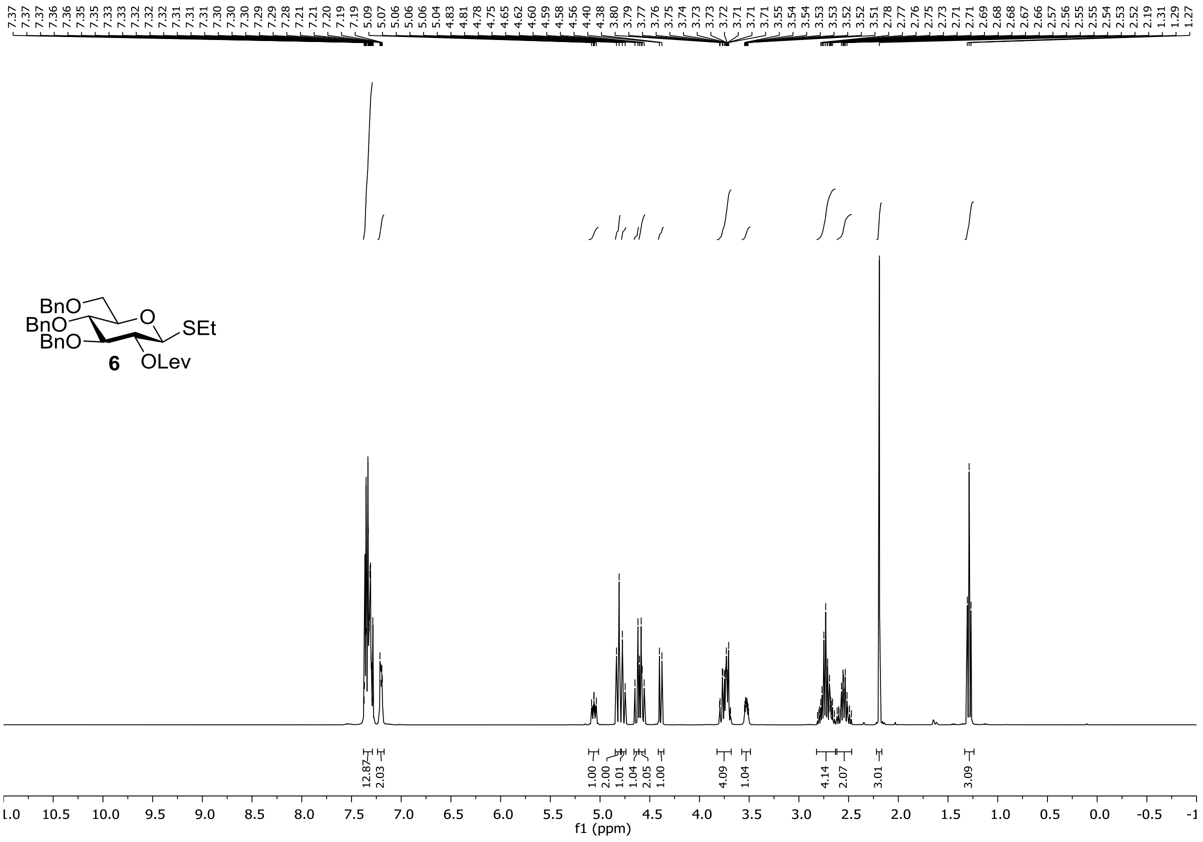

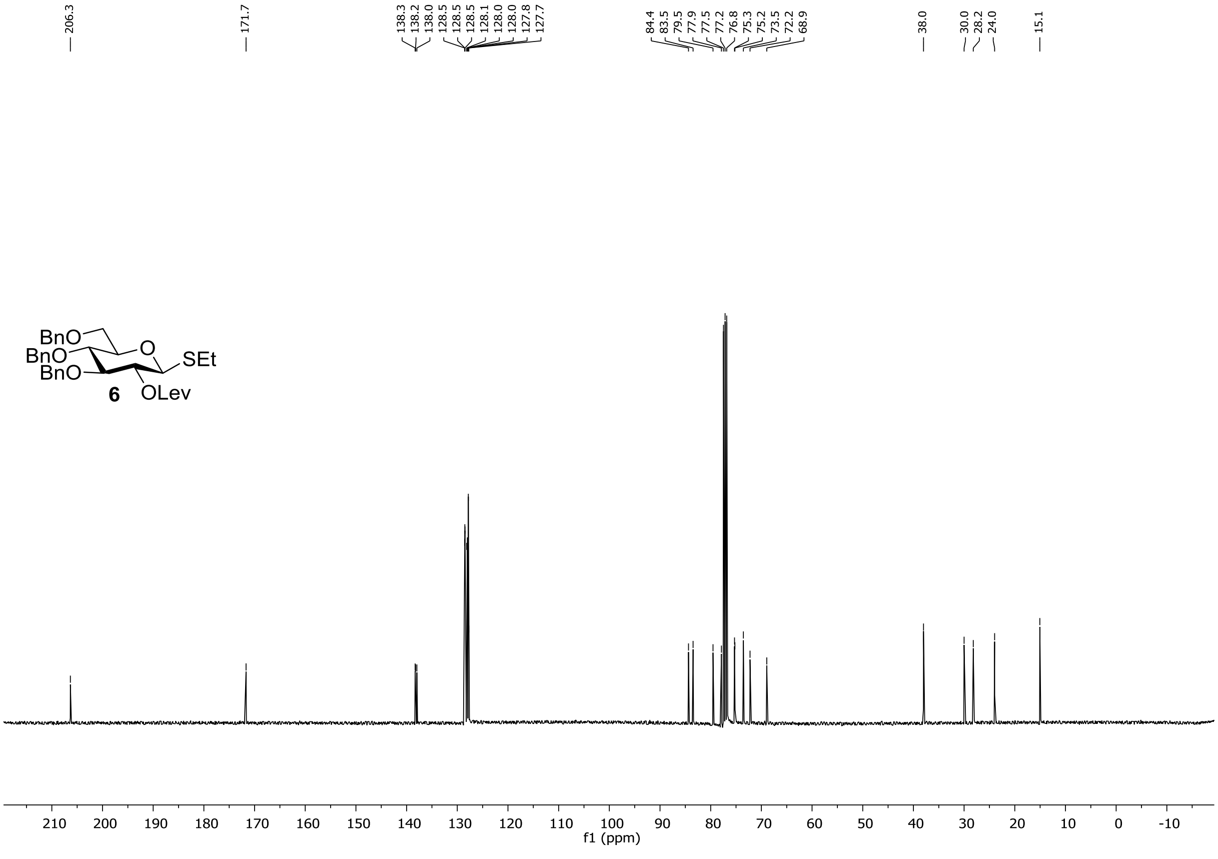

Ethyl 3-*O*-benzyl-6-*O*-levulinoyl-4-*O*-(9-fluorenylmethoxycarbonyl)-2-*N*-trichloroacetyl-1-thio-β-D-glucosaminopyranoside (7)

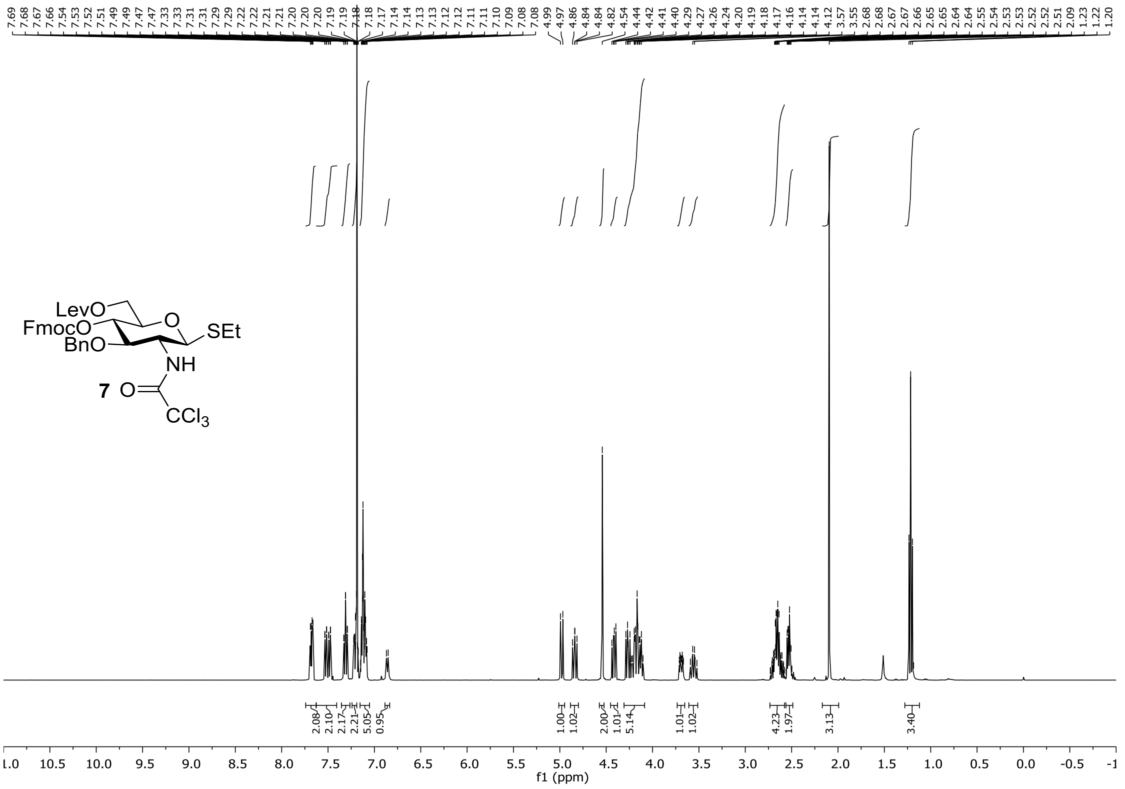

Ethyl 2-*O*-benzoyl-3,4-di-*O*-benzyl-6-*O*-(9-fluorenylmethoxycarbonyl)-1-thio-α-D-mannopyranoside (8)

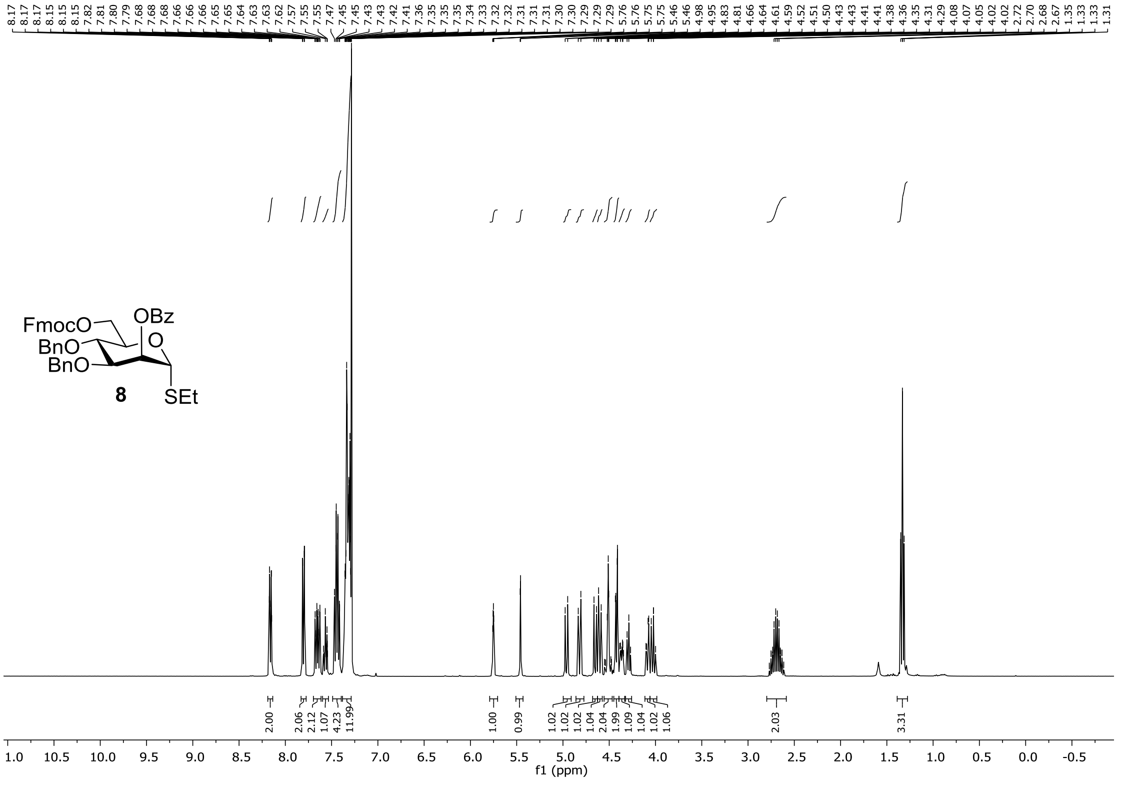

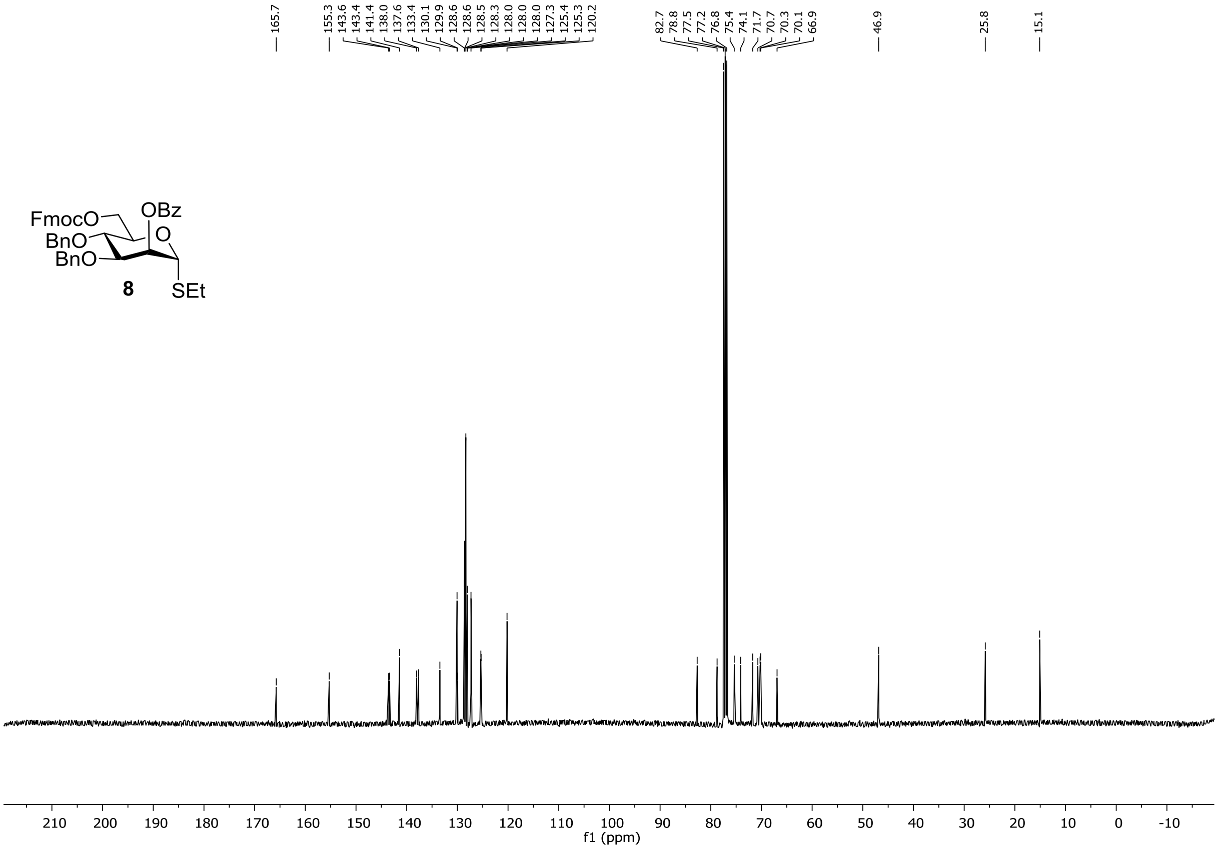

p-Tolyl 2-*O*-benzoyl-4-*O*-benzyl-3-*O*-(9-fluorenylmethoxycarbonyl)-1-thio-α-L-rhamnopyranoside (9)

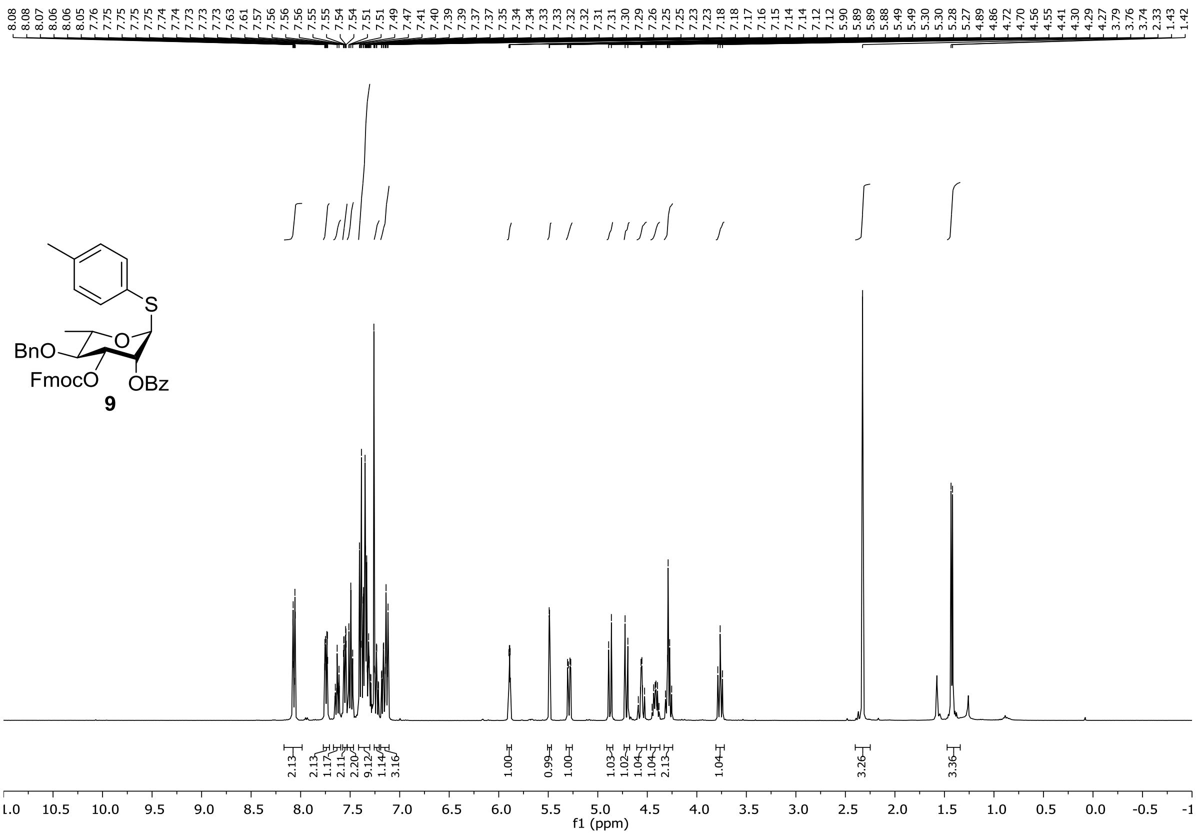

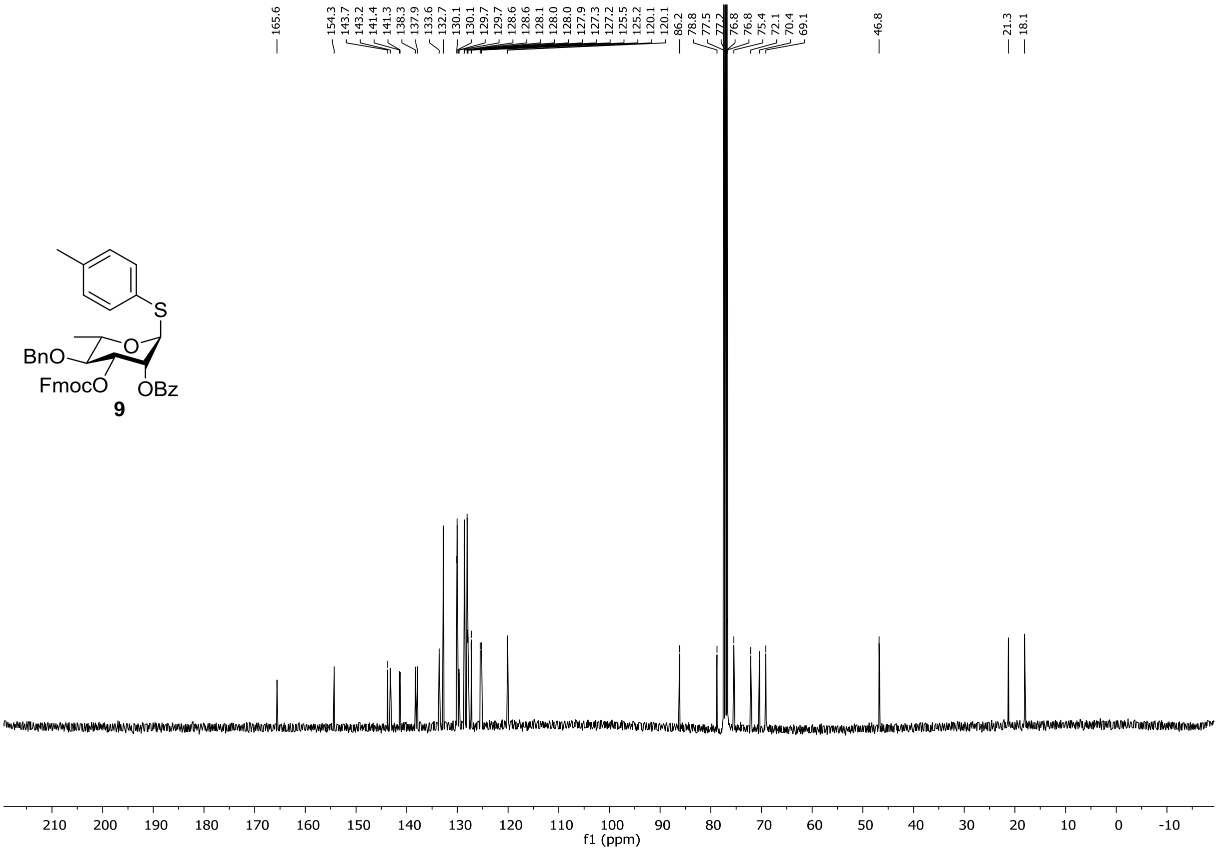

Benzyl (ethyl 3,4-di-*O*-benzyl-2-*O*-levulinoyl-1-thio-β-D-glucopyranosid)uronate (10)

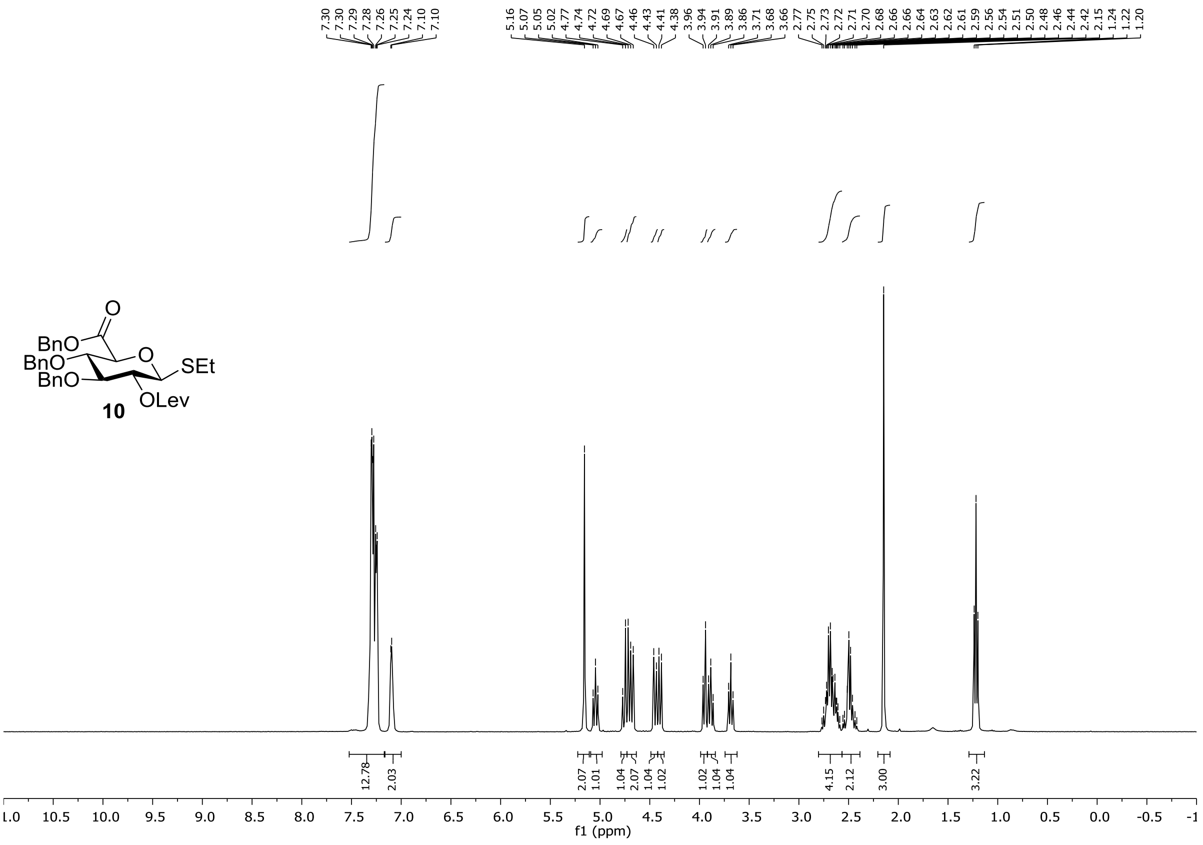

Methyl 2,3,6-tri-*O*-benzyl-α-D-glucopyranoside (11)

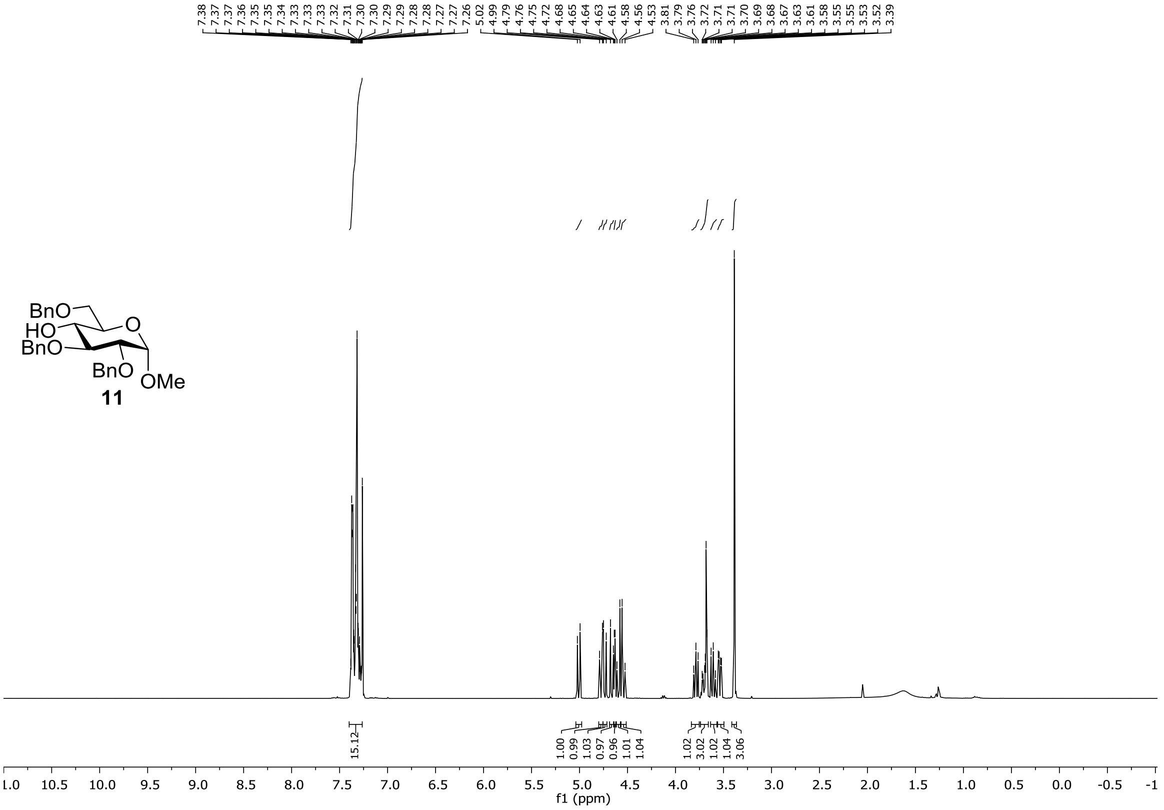

Methyl 2-*O*-benzoyl-4,6-di-*O*-benzyl-β-D-galactopyranosyl-(1→6)-2,3,4-tri-*O*-benzyl-α-D-glucopyranoside (3)

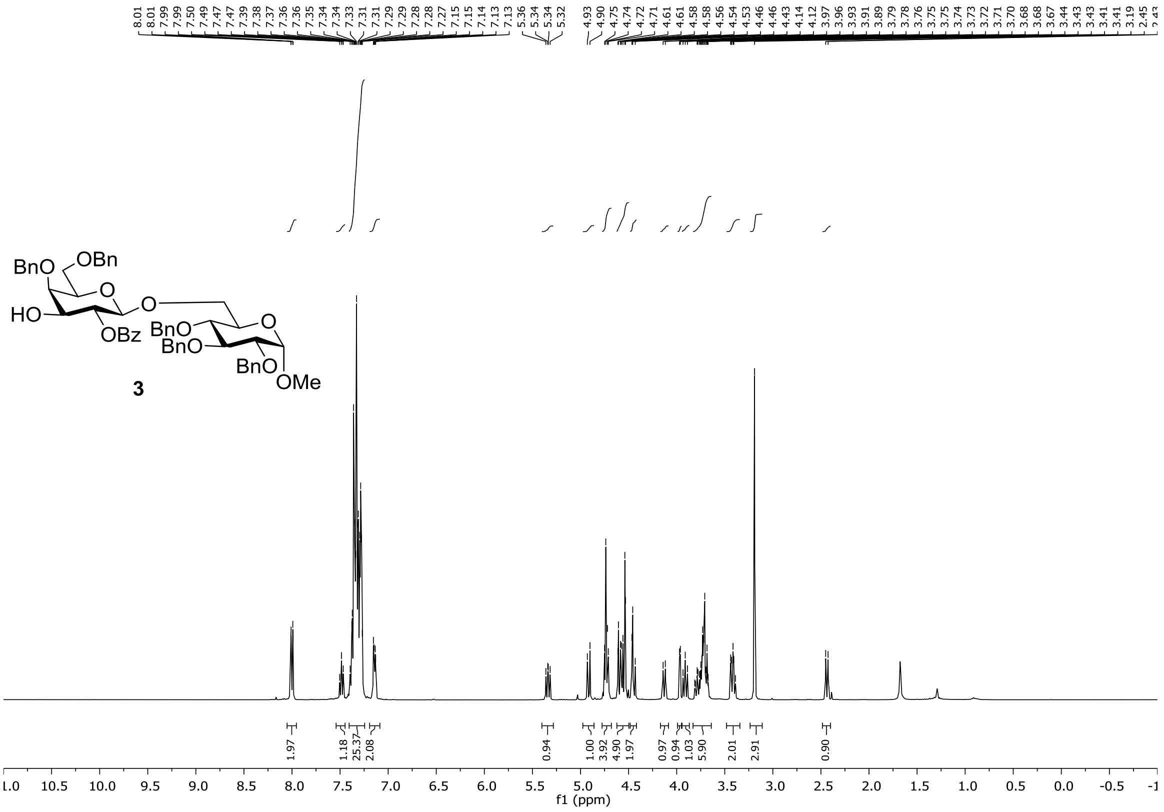

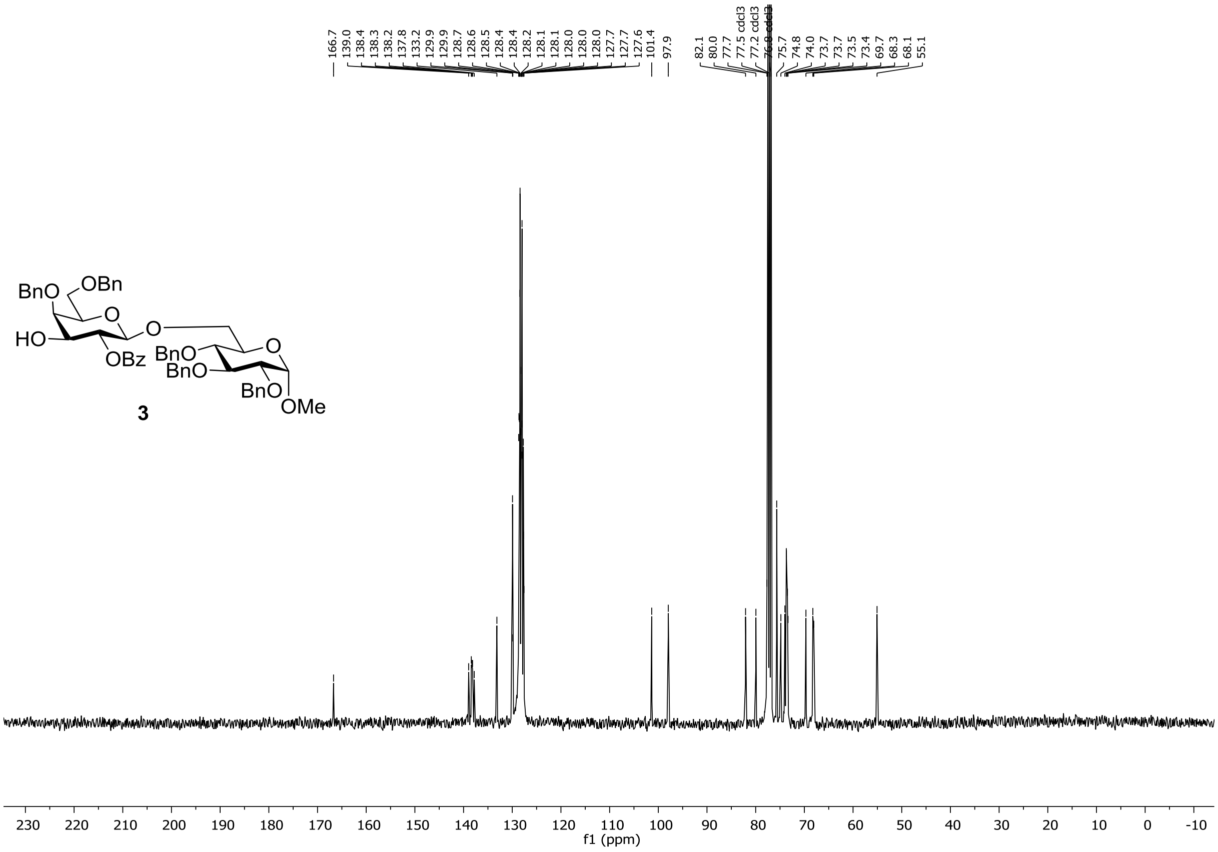
BnO OBn
O
O
HO
OBz BnO
O
BnO
BnO
OMe
3
166.7
139.0
138.4
138.3
138.2
137.8
133.2
129.9
129.9
128.7
128.6
128.5
128.4
128.4
128.2
128.1
128.1
128.0
128.0
128.0
127.7
127.7
127.6
101.4
97.9
82.1
80.0
77.7
77.5 cdcl3
77.2 cdcl3
76.8 cdcl3
75.7
74.8
74.0
73.7
73.7
73.5
73.4
69.7
68.3
68.1
55.1
230 220 210 200 190 180 170 160 150 140 130 120 110 100 90 80 70 60 50 40 30 20 10 0 -10
f1 (ppm)

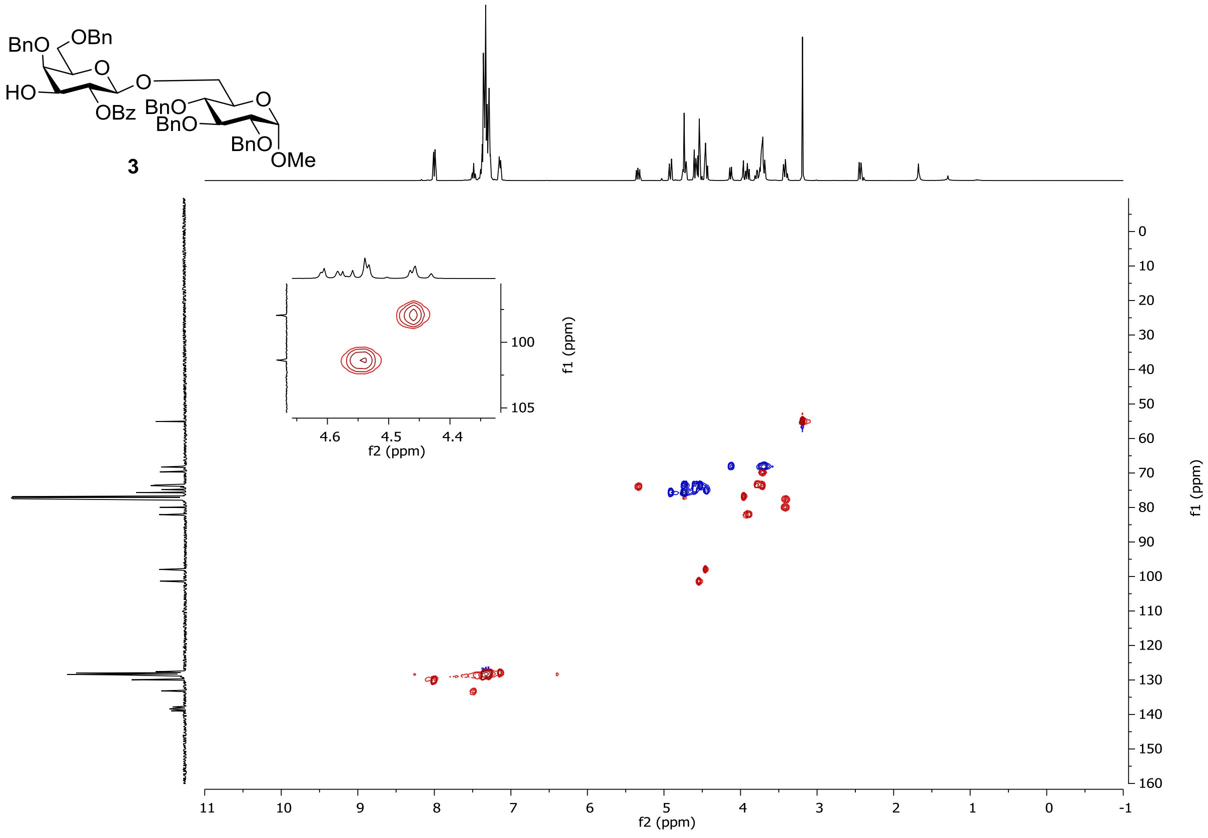
BnO OBn
O
O
HO
OBz BnO
O
BnO
BnO
OMe
3
4.6 4.5 4.4
f2 (ppm)
100
105
f1 (ppm)
0 10 20 30 40 50 60 70 80 90 100 110 120 130 140 150 160
f1 (ppm)
11 10 9 8 7 6 5 4 3 2 1 0 -1
f2 (ppm)

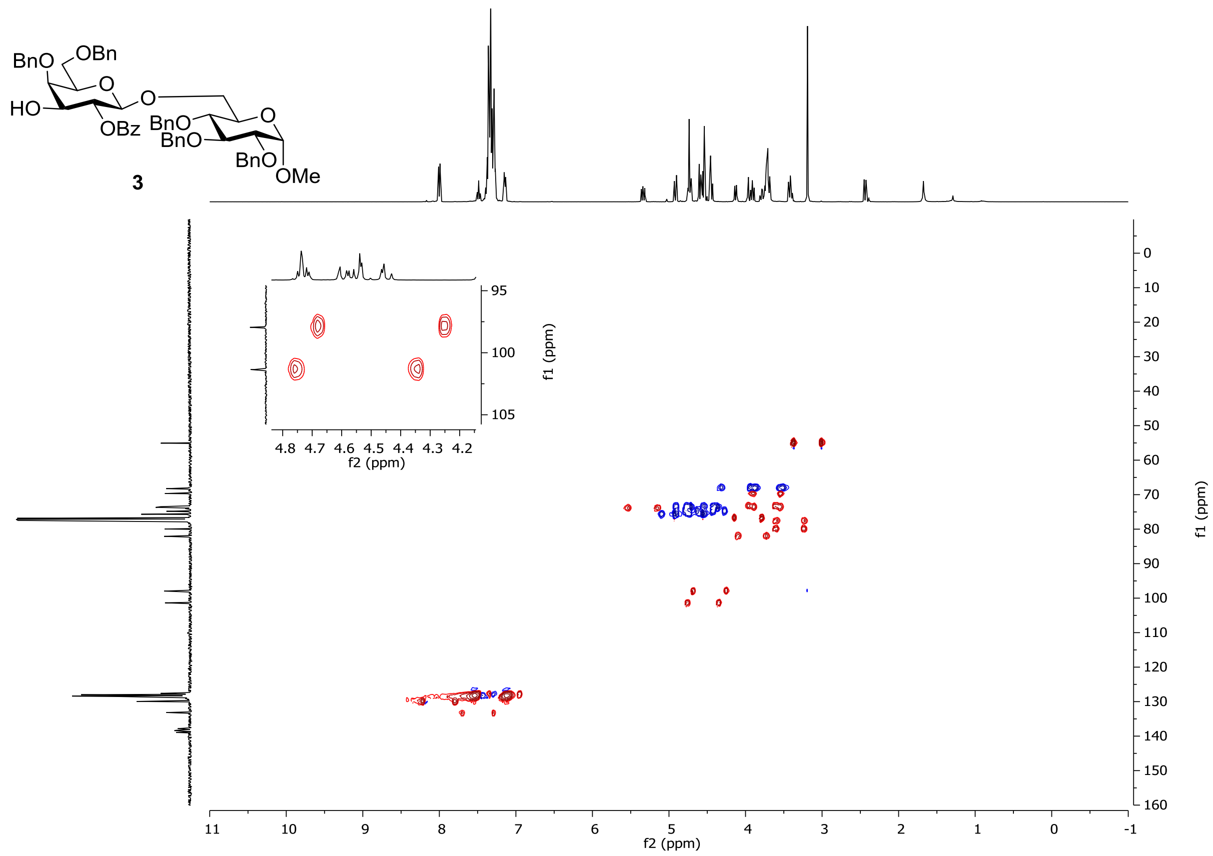

Methyl 2,3-di-*O*-benzoyl-4,6-*O*-benzylidene-β-D-galactopyranosyl-(1→6)-2,3,4-tri-*O*-benzyl-α-D-glucopyranoside (S5)

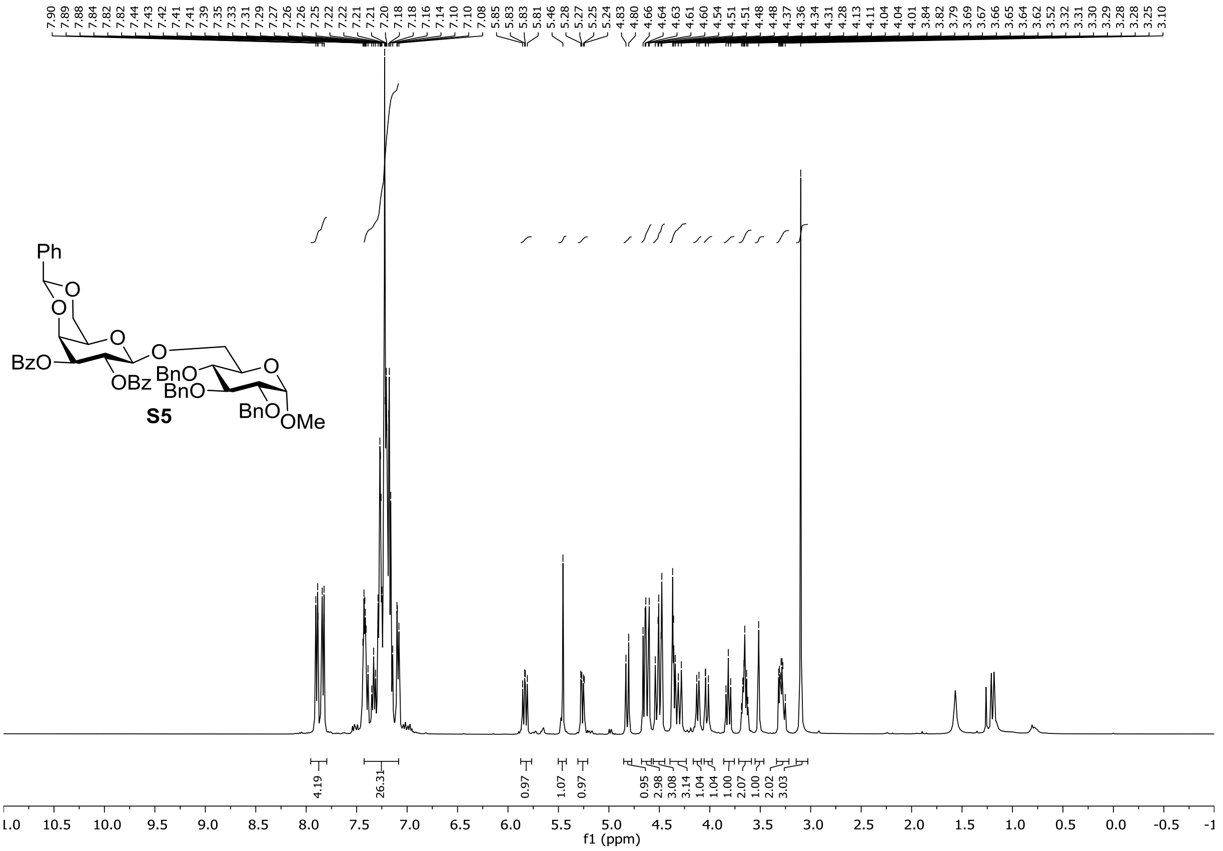

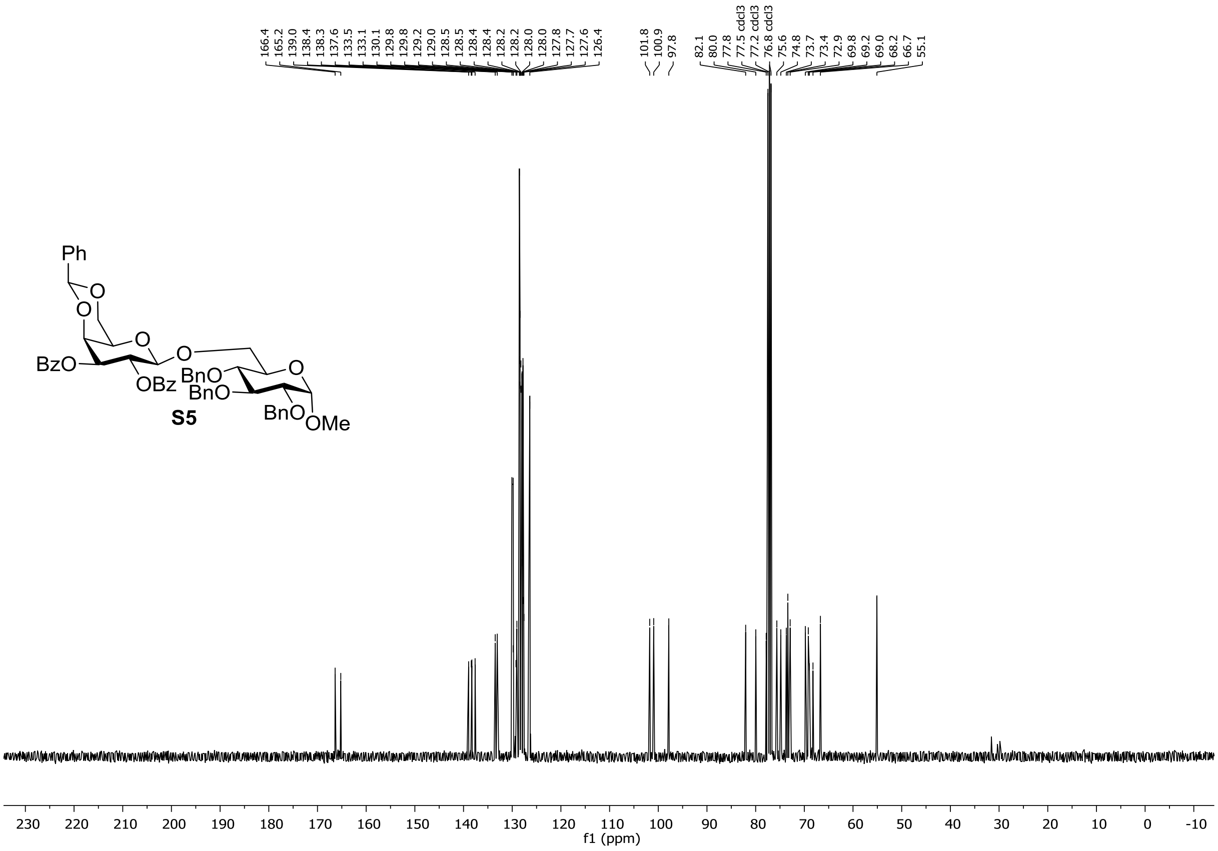

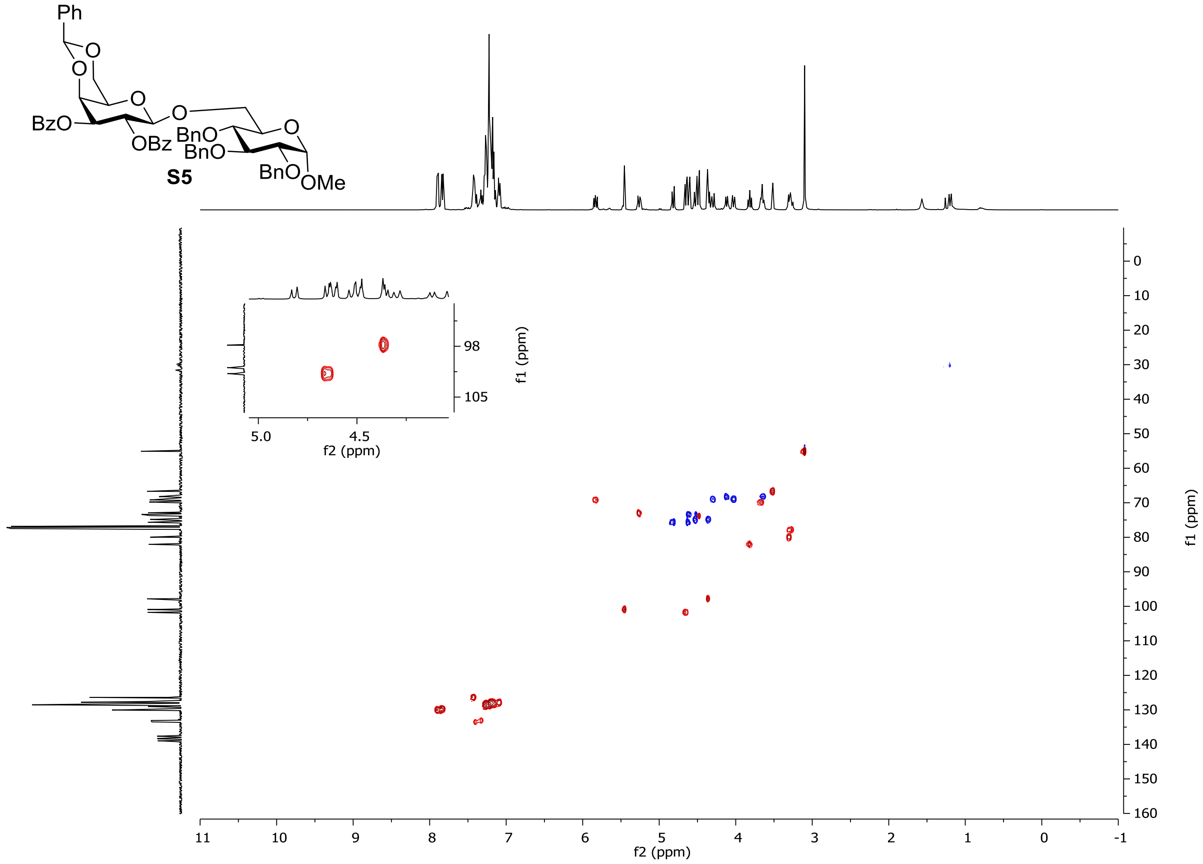

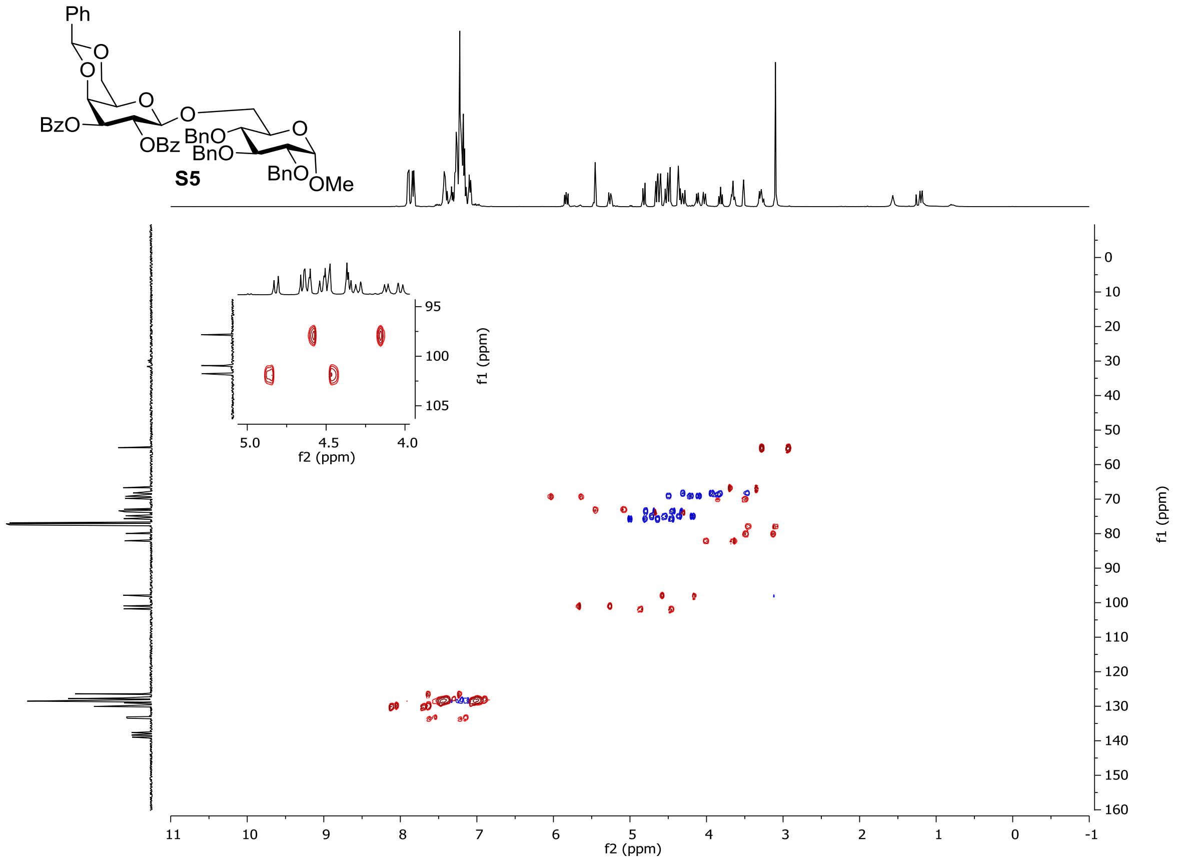

Methyl 2,3-di-*O*-benzoyl-6-*O*-benzyl-β-D-glucopyranosyl-(1→6)-2,3,4-tri-*O*-benzyl-α-D-glucopyranoside (S6)

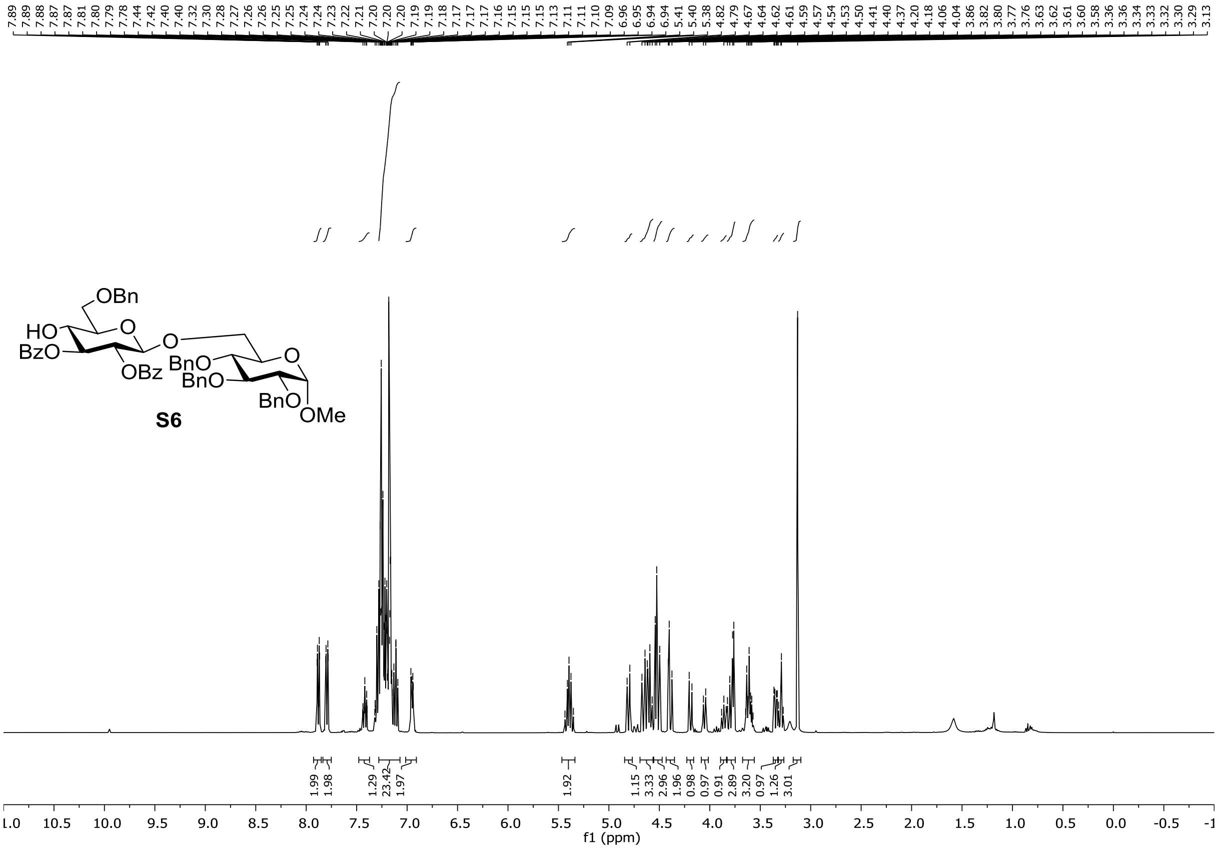

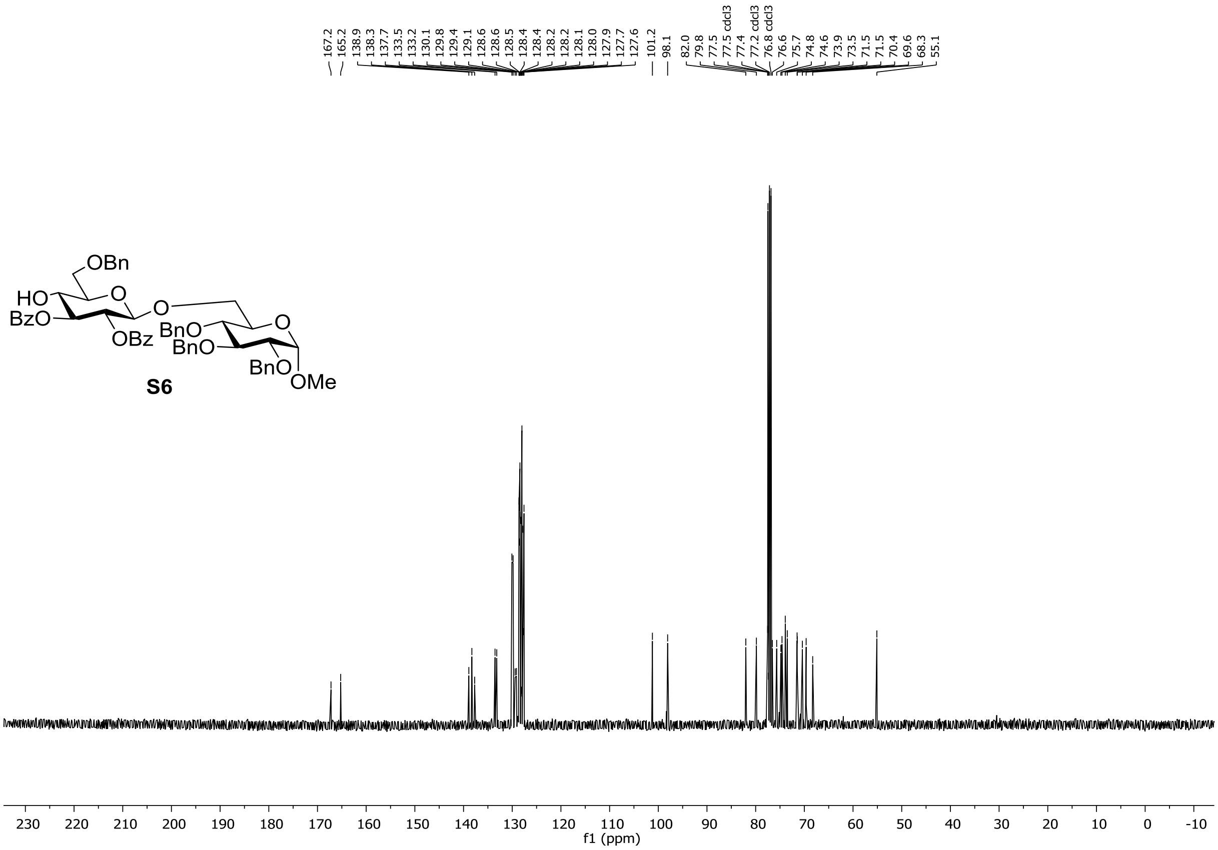
167.2
165.2
138.9
138.3
137.7
133.5
133.2
130.1
129.8
129.4
129.1
128.6
128.6
128.5
128.4
128.4
128.2
128.2
128.1
128.0
127.9
127.7
127.6
101.2
98.1
82.0
79.8
77.5 cdcl3
77.4
77.2 cdcl3
76.8 cdcl3
76.6
75.7
74.8
74.6
73.9
73.5
71.5
70.4
69.6
68.3
55.1
OBn
HO
BzO
O
O
OBz
BnO
BnO
O
BnO
OMe
S6
f1 (ppm)

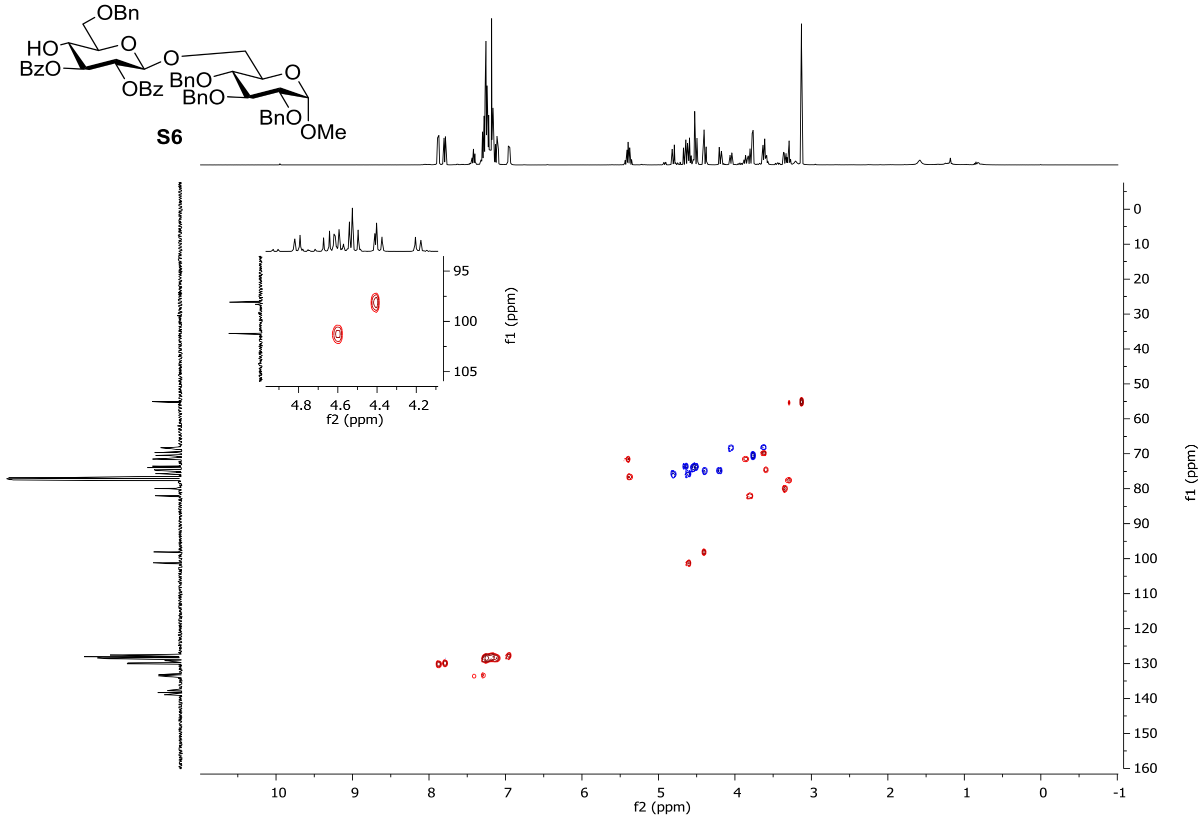
OBn
HO
BzO
O
O
OBz
BnO
BnO
O
BnO
OMe
S6
f2 (ppm)
f1 (ppm)

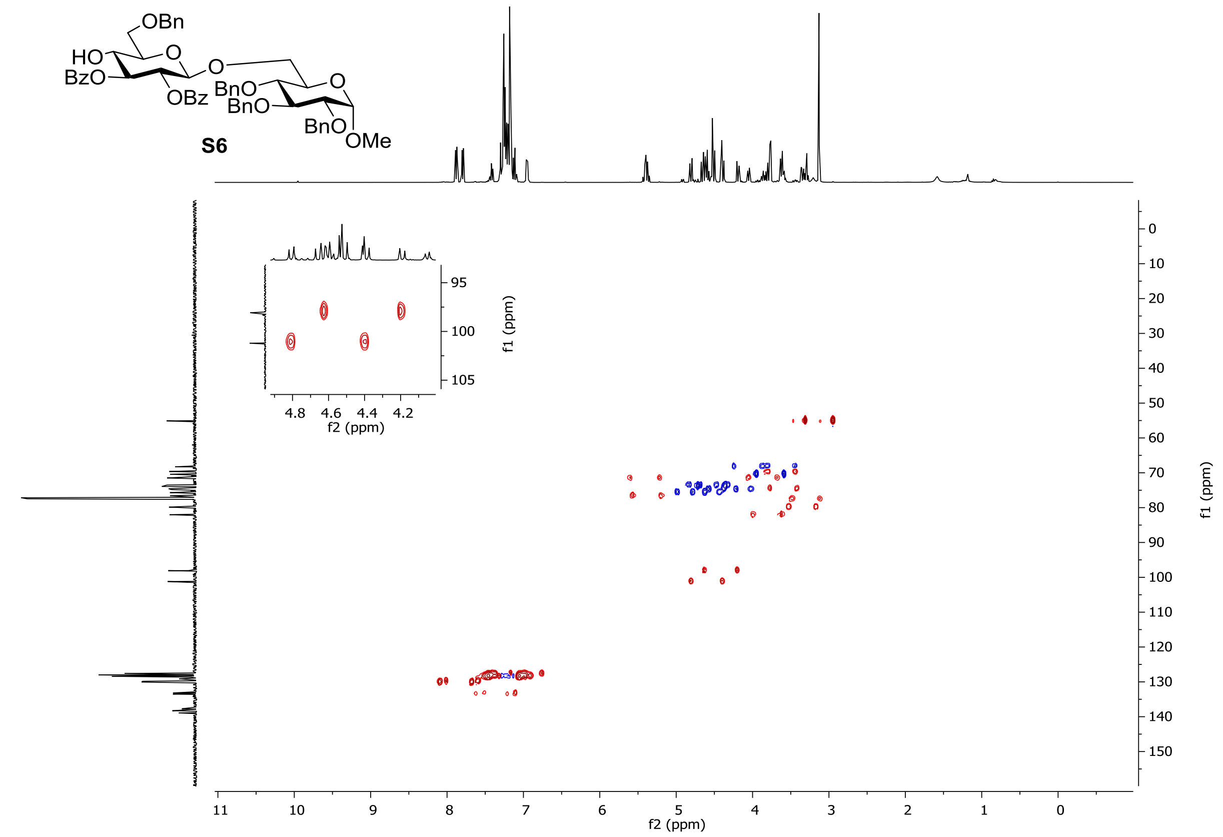

Methyl 3,4,6-tri-*O*-benzyl-2-*O*-levulinoyl-β-D-glucopyranosyl-(1→6)-2,3,4-tri-*O*-benzyl-α-D-glucopyranoside (S7)

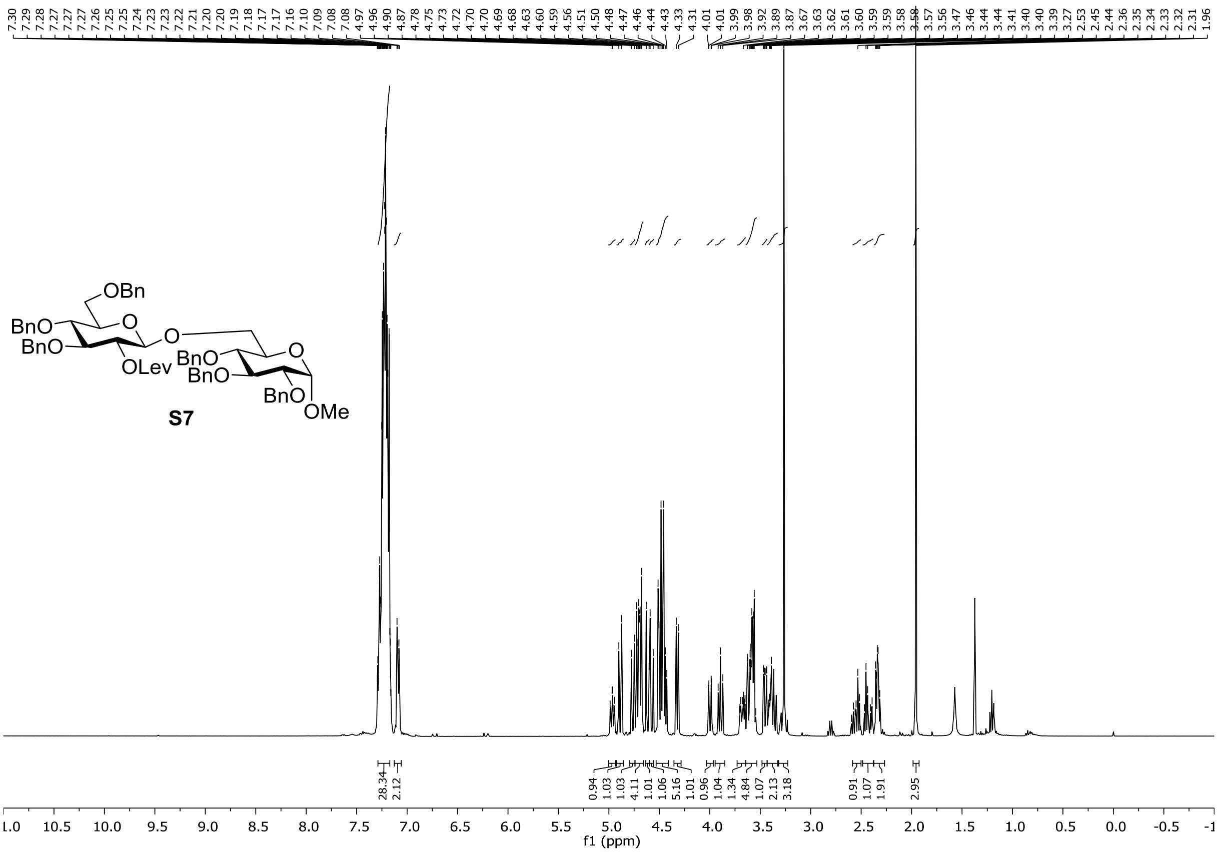

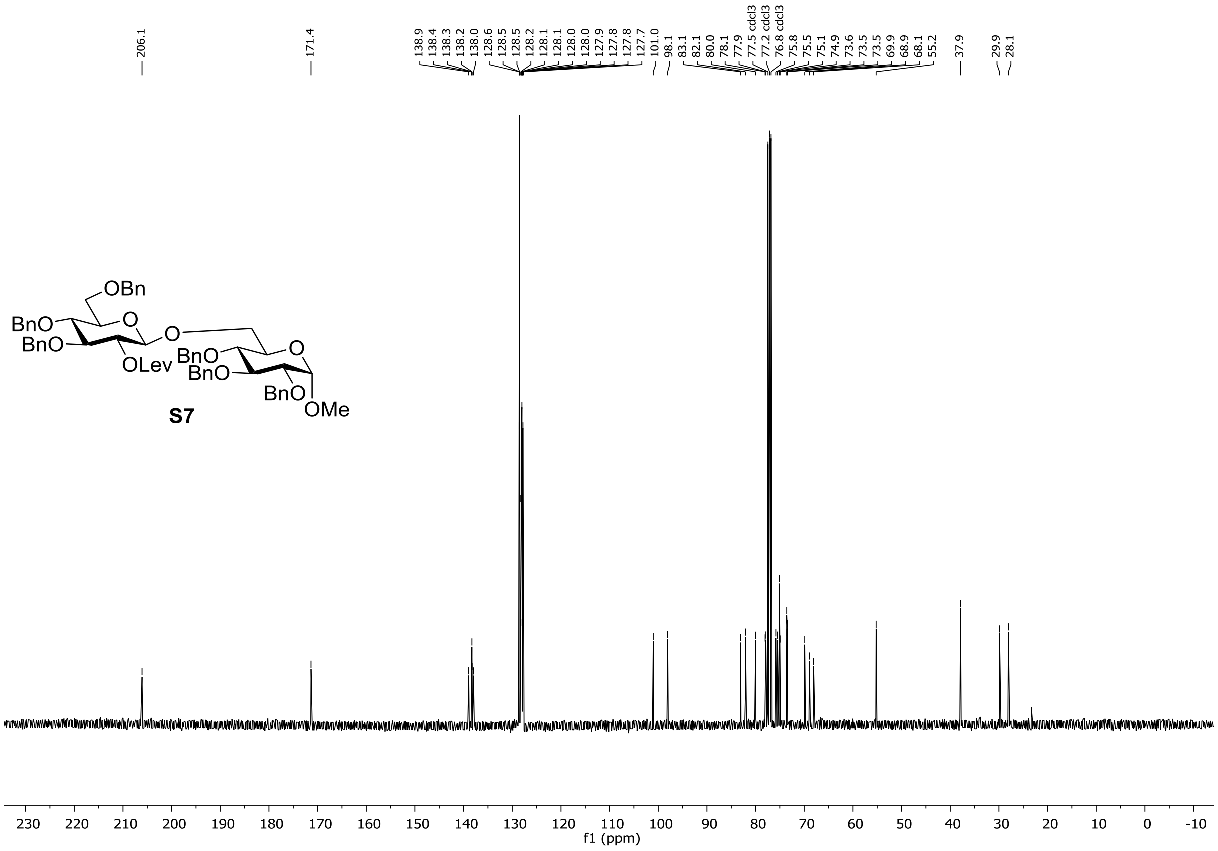
206.1
171.4
138.9
138.4
138.3
138.2
138.0
128.6
128.5
128.5
128.2
128.1
128.1
128.0
128.0
127.9
127.8
127.8
127.7
101.0
98.1
83.1
82.1
80.0
78.1
77.9
77.5 cdcl3
77.2 cdcl3
76.8 cdcl3
75.8
75.5
75.1
74.9
73.6
73.5
69.9
68.9
68.1
55.2
37.9
29.9
28.1
OBn
BnO
BnO
O
O
OLev
BnO
BnO
O
BnO
OMe
S7
f1 (ppm)

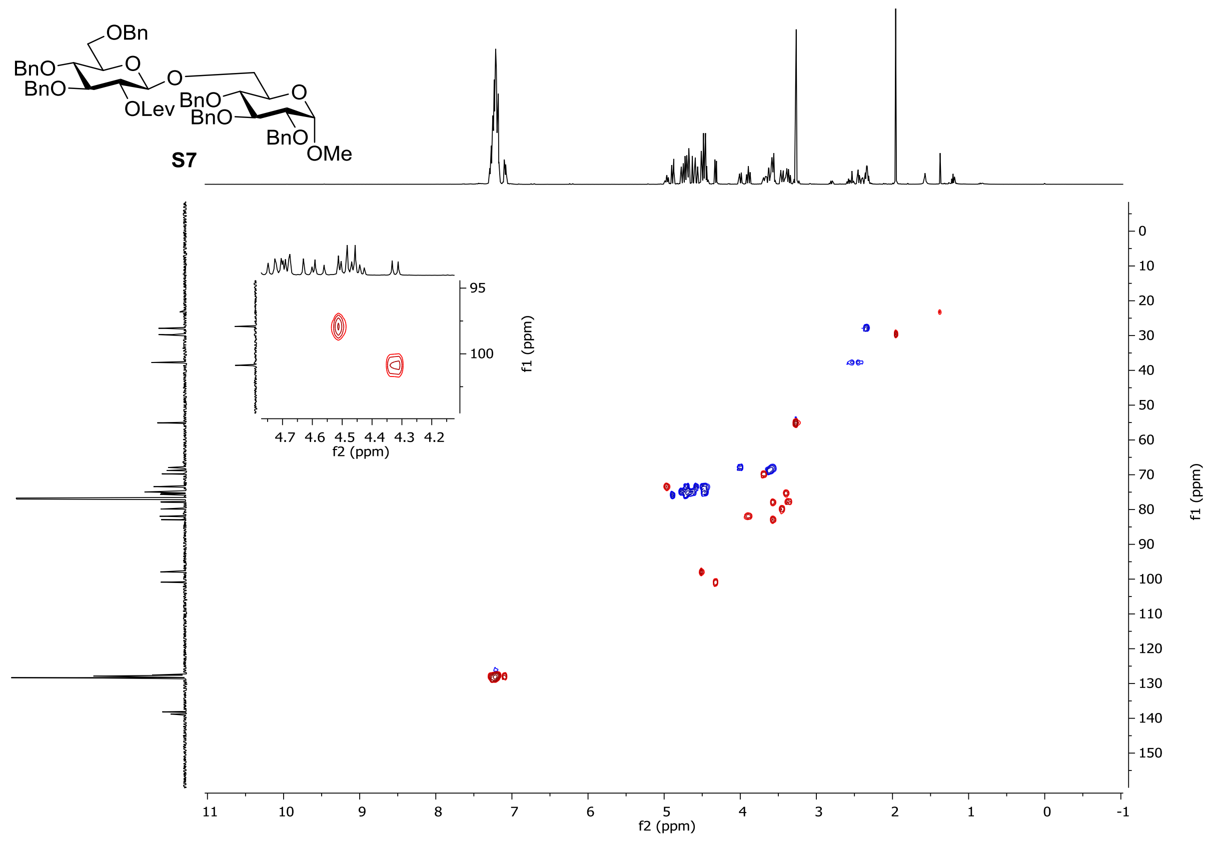
OBn
BnO
BnO
O
O
OLev
BnO
BnO
O
BnO
OMe
S7
f1 (ppm)
f2 (ppm)

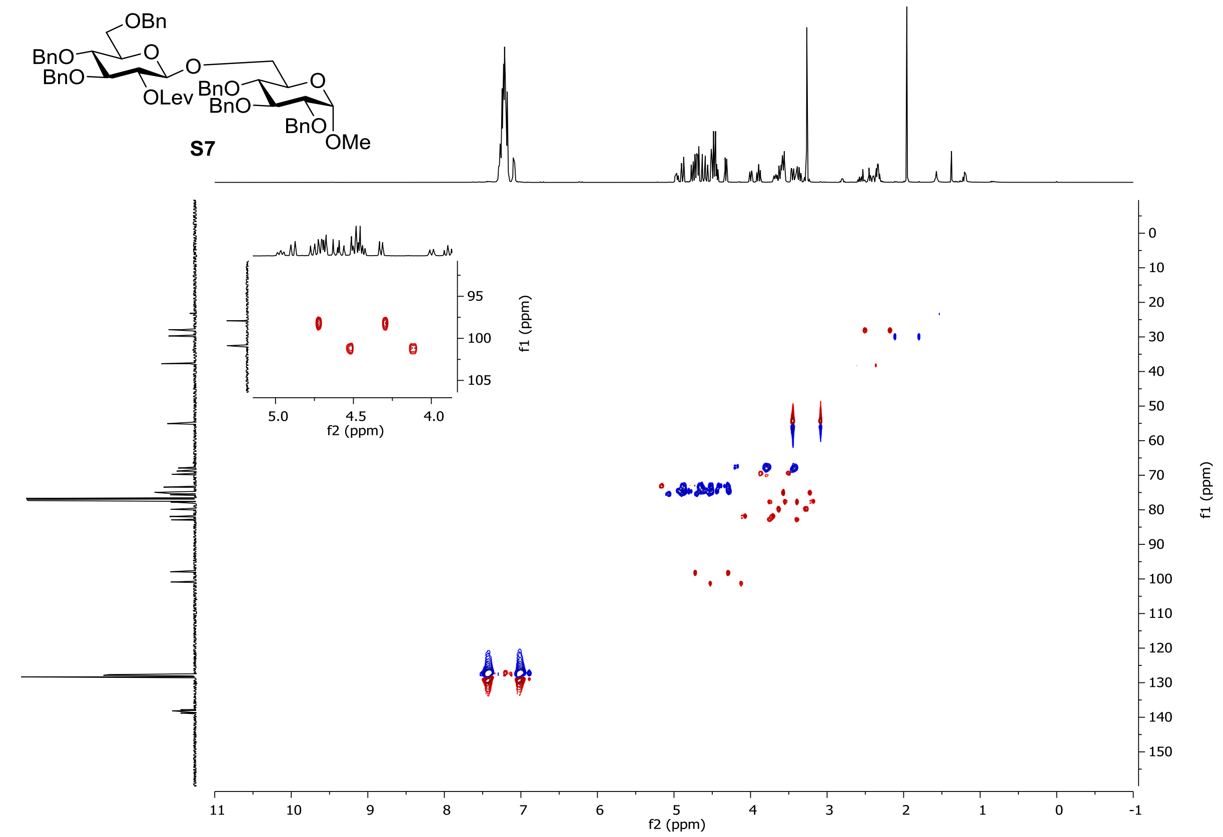

Methyl 3-*O*-benzyl-6-*O*-levulinoyl-2-*N*-trichloroacetyl-β-D-glucosaminopyranosyl-(1→6)-2,3,4-tri-*O*-benzyl-α-D-glucopyranoside (S8)

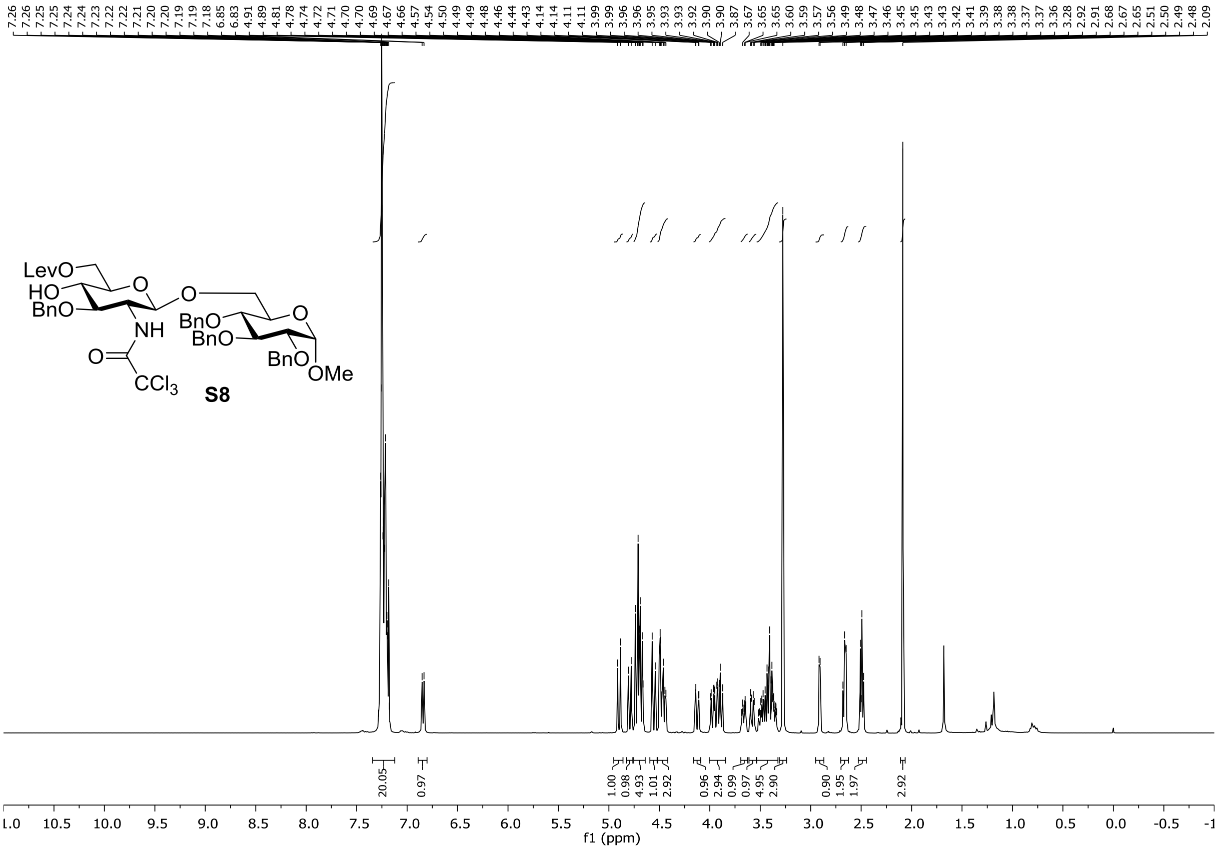

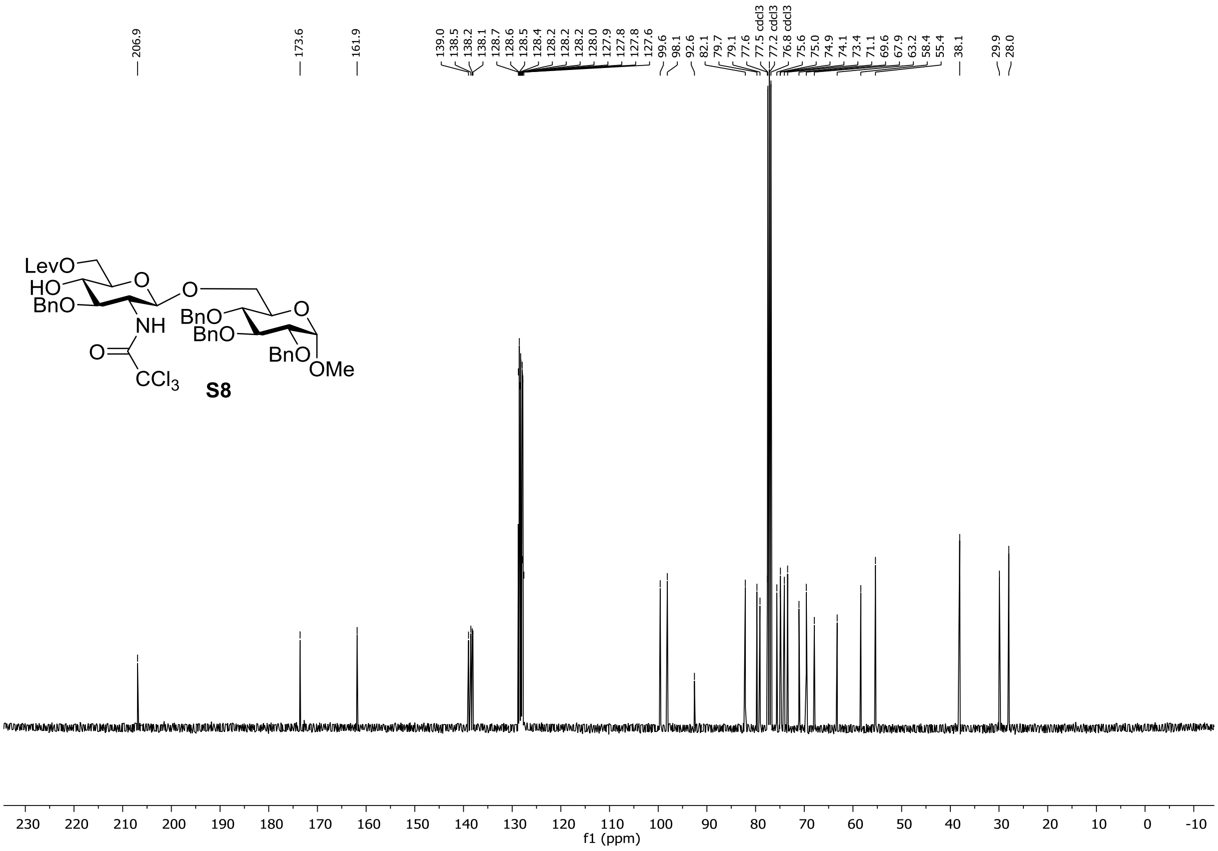
206.9
173.6
161.9
139.0
138.5
138.2
138.1
128.7
128.6
128.5
128.4
128.2
128.2
128.2
128.0
127.9
127.8
127.8
127.6
99.6
98.1
92.6
82.1
79.7
79.1
77.6
77.5 cdcl3
77.2 cdcl3
76.8 cdcl3
75.6
75.0
74.9
74.1
73.4
71.1
69.6
67.9
63.2
58.4
55.4
38.1
29.9
28.0
f1 (ppm)
S8

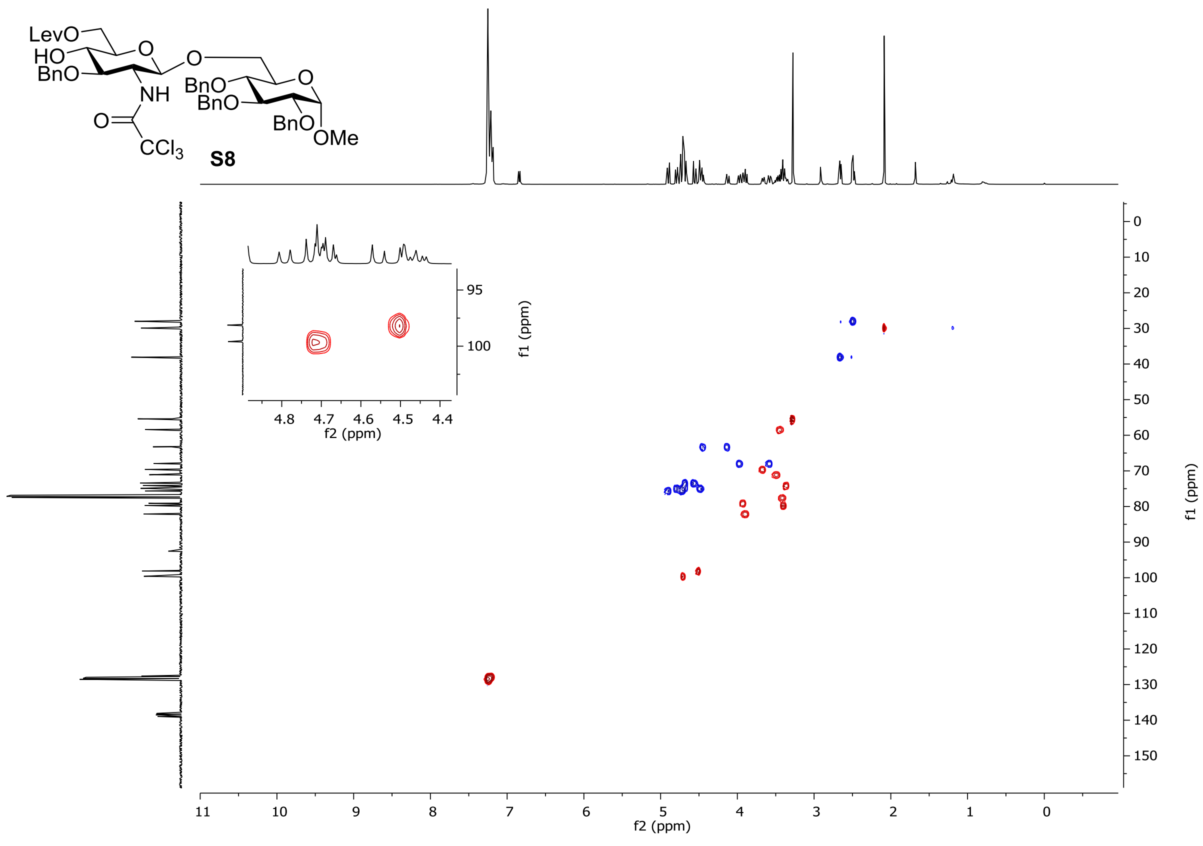
S8
f1 (ppm)
f2 (ppm)

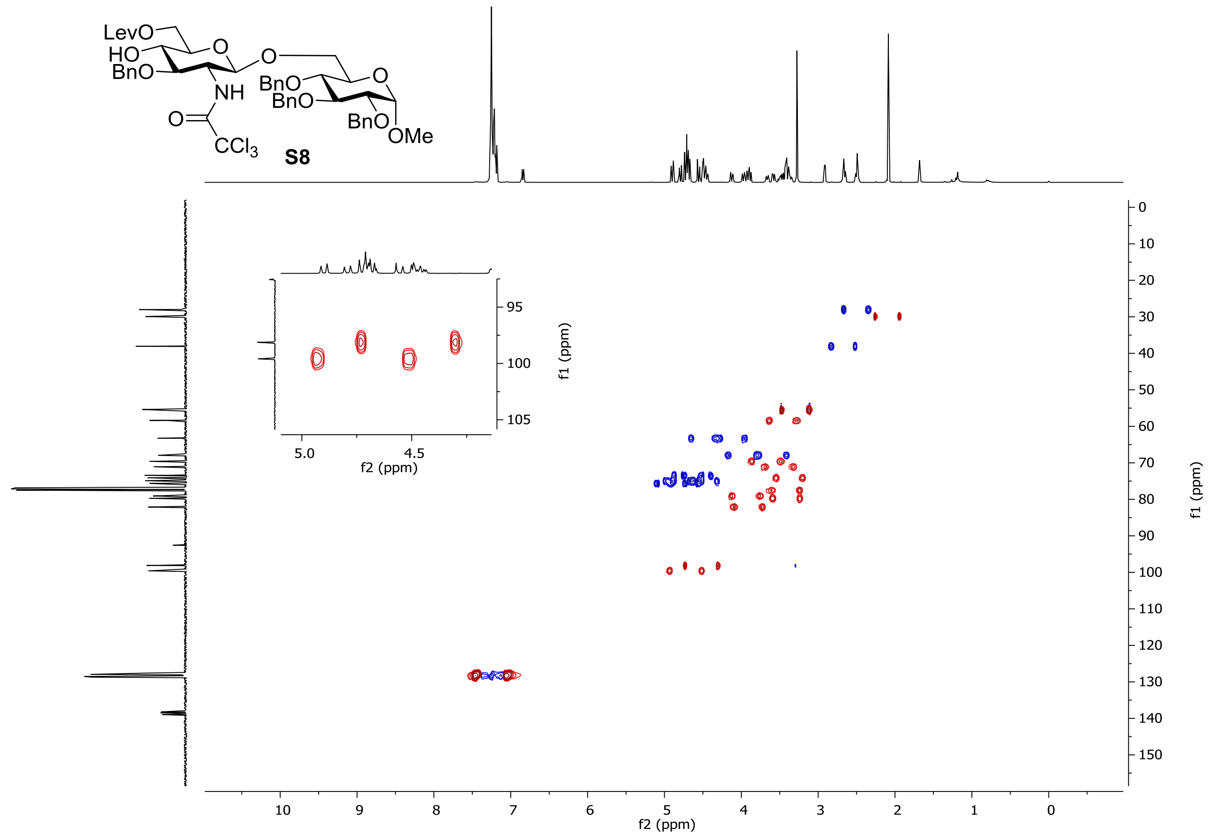

Methyl 2-*O*-benzoyl-3,4-di-*O*-benzyl-α-D-mannopyranosyl-(1→6)-2,3,4-tri-*O*-benzyl-α-D-glucopyranoside (S9)

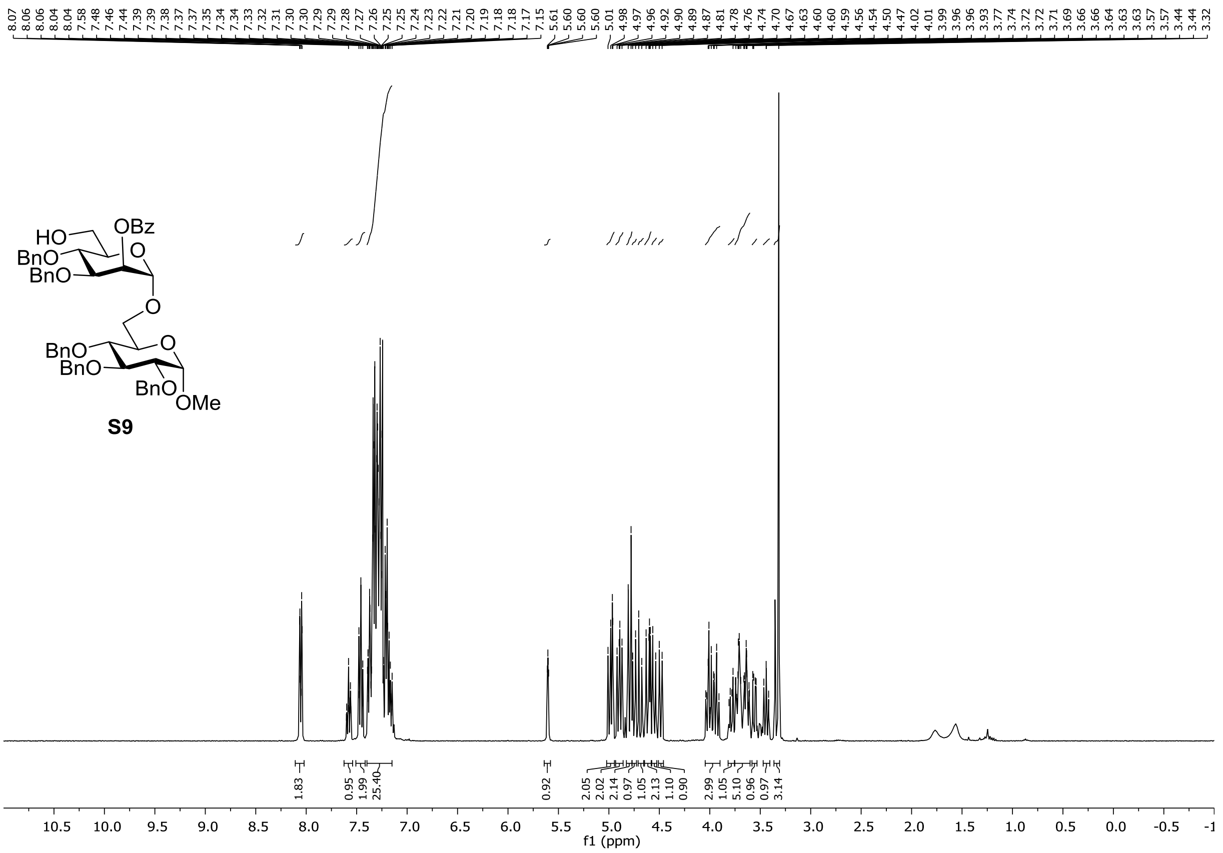

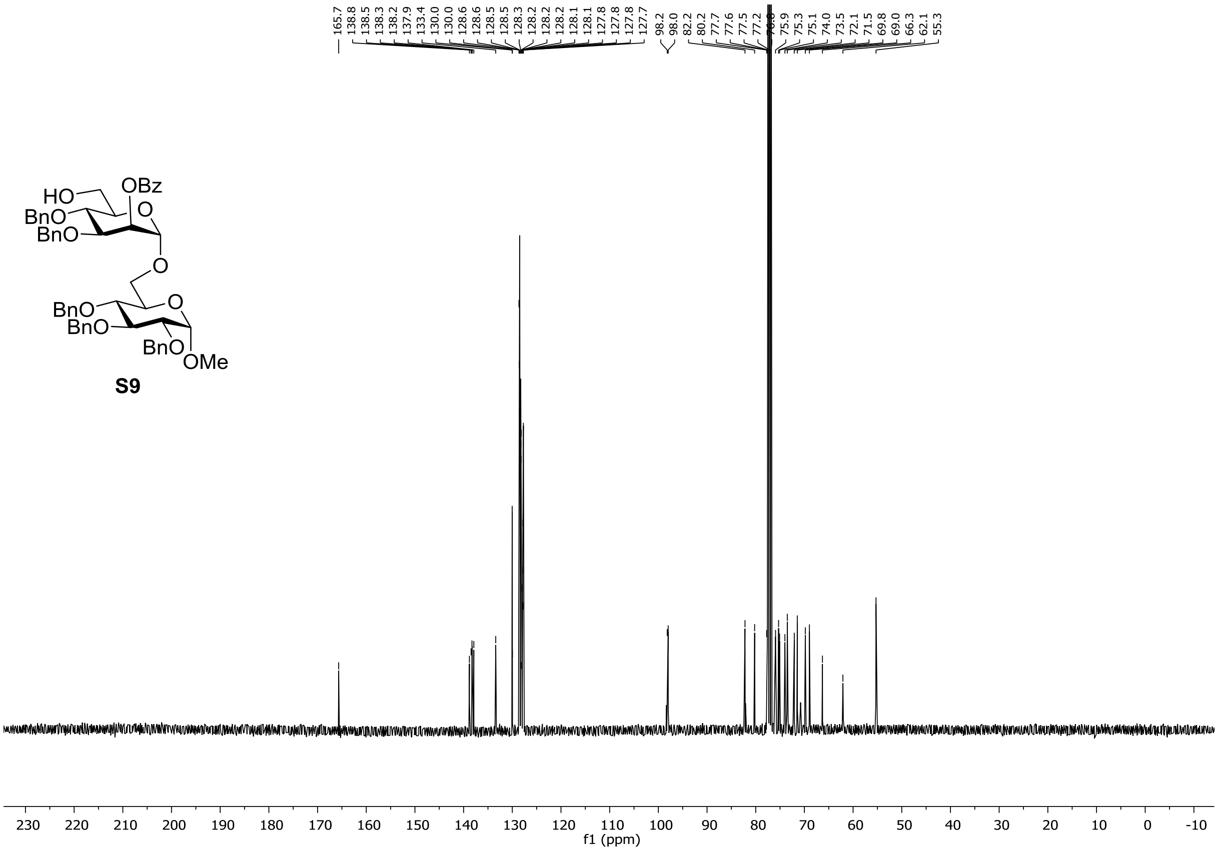

S9

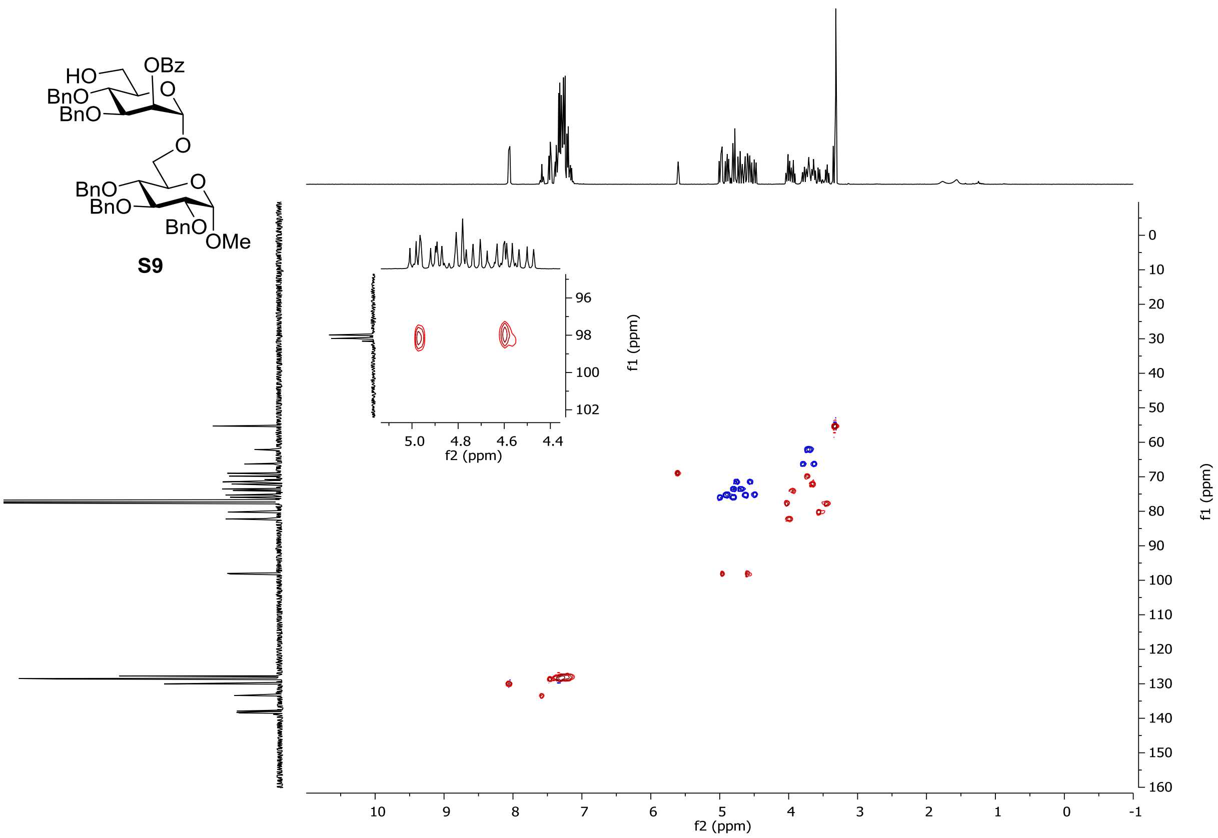

S9

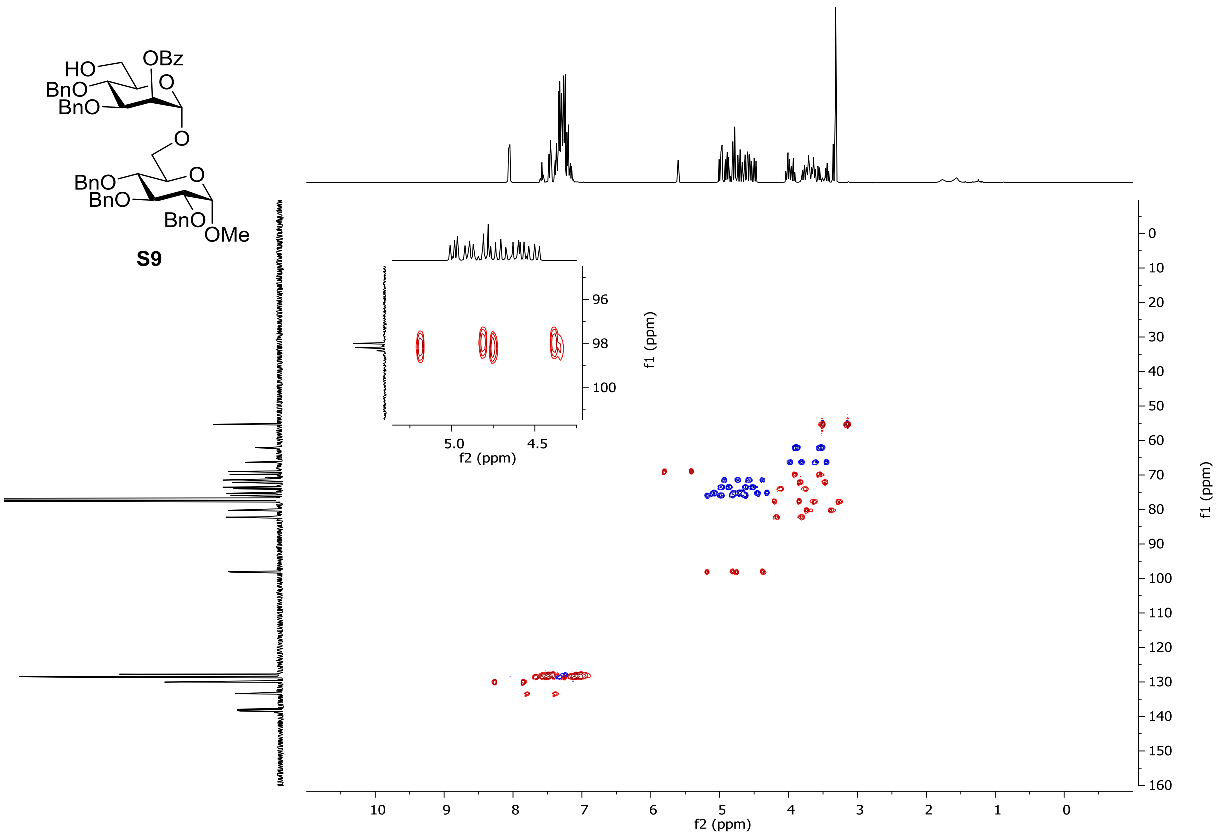

Methyl 2-*O*-benzoyl-4-*O*-benzyl-α-L-rhamnopyranosyl-(1→6)-2,3,4-tri-*O*-benzyl-α-D-glucopyranoside (S10)

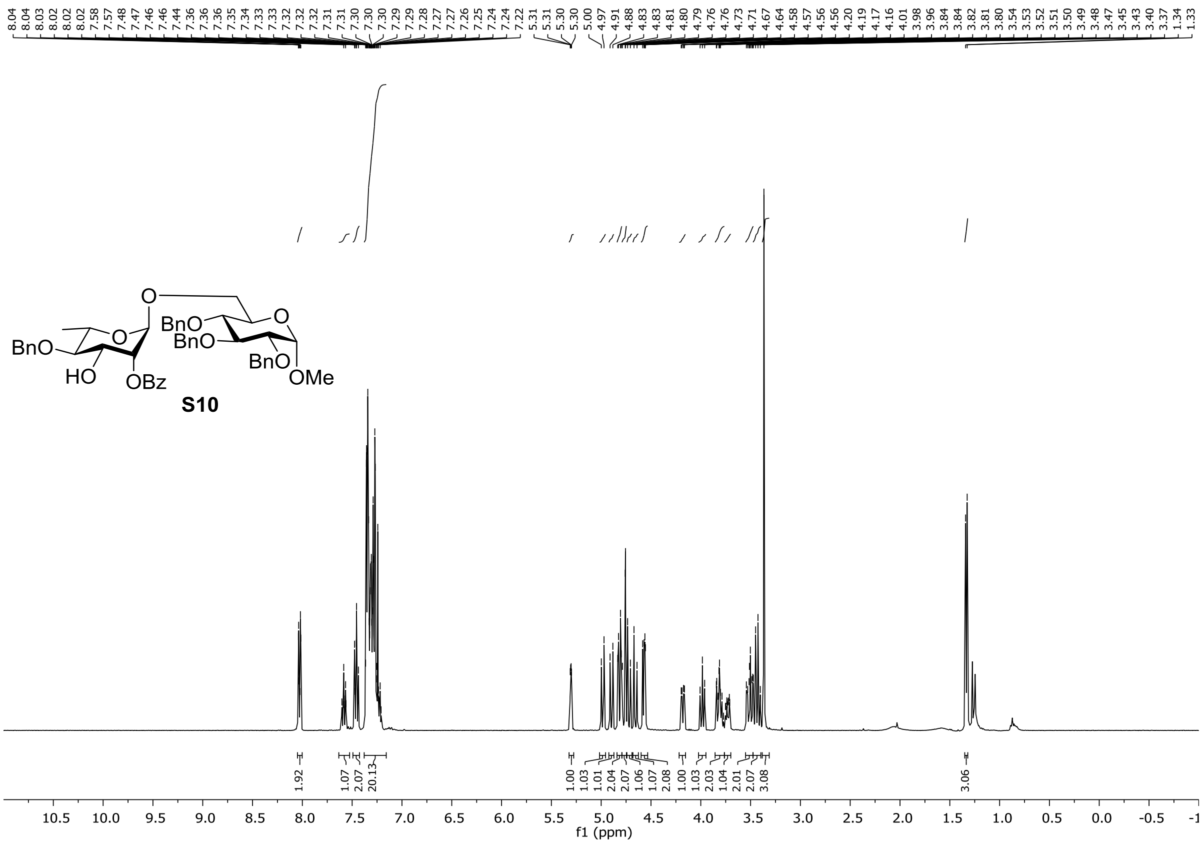

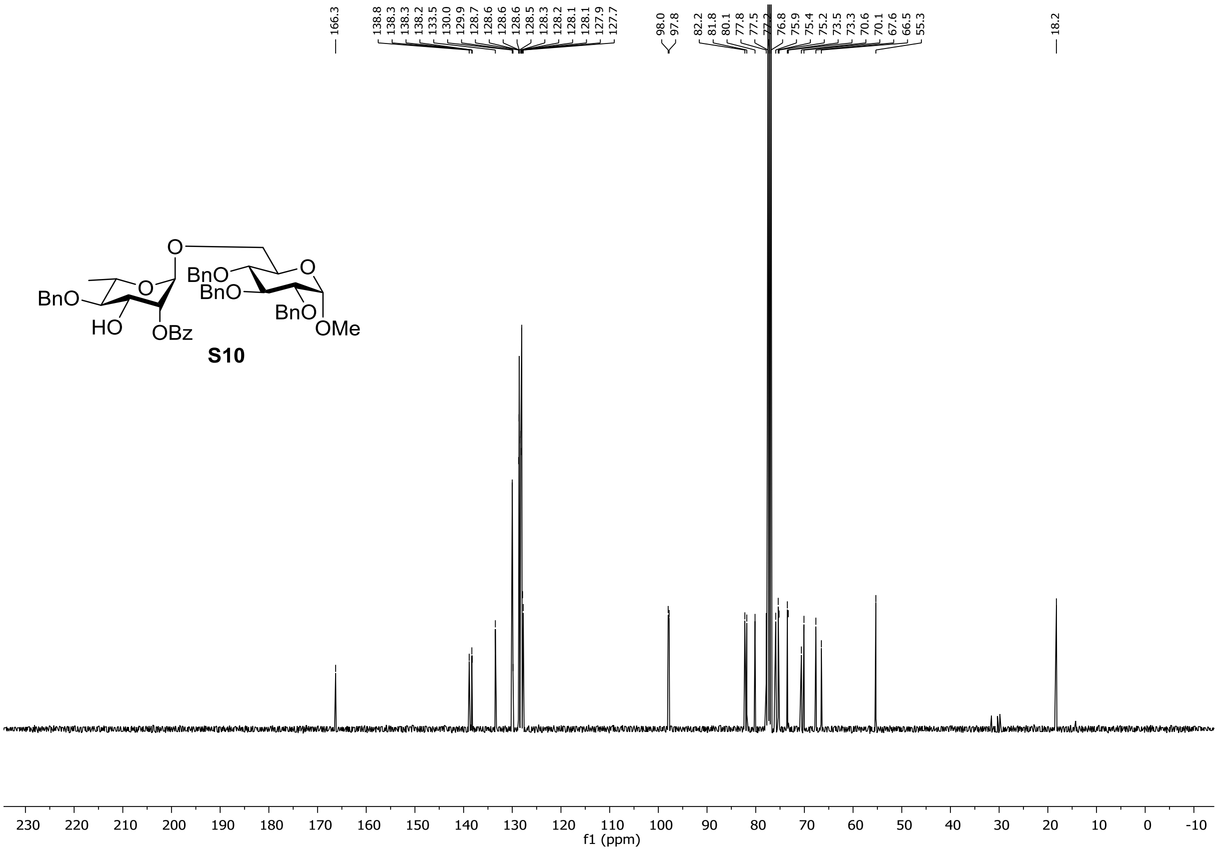

S10

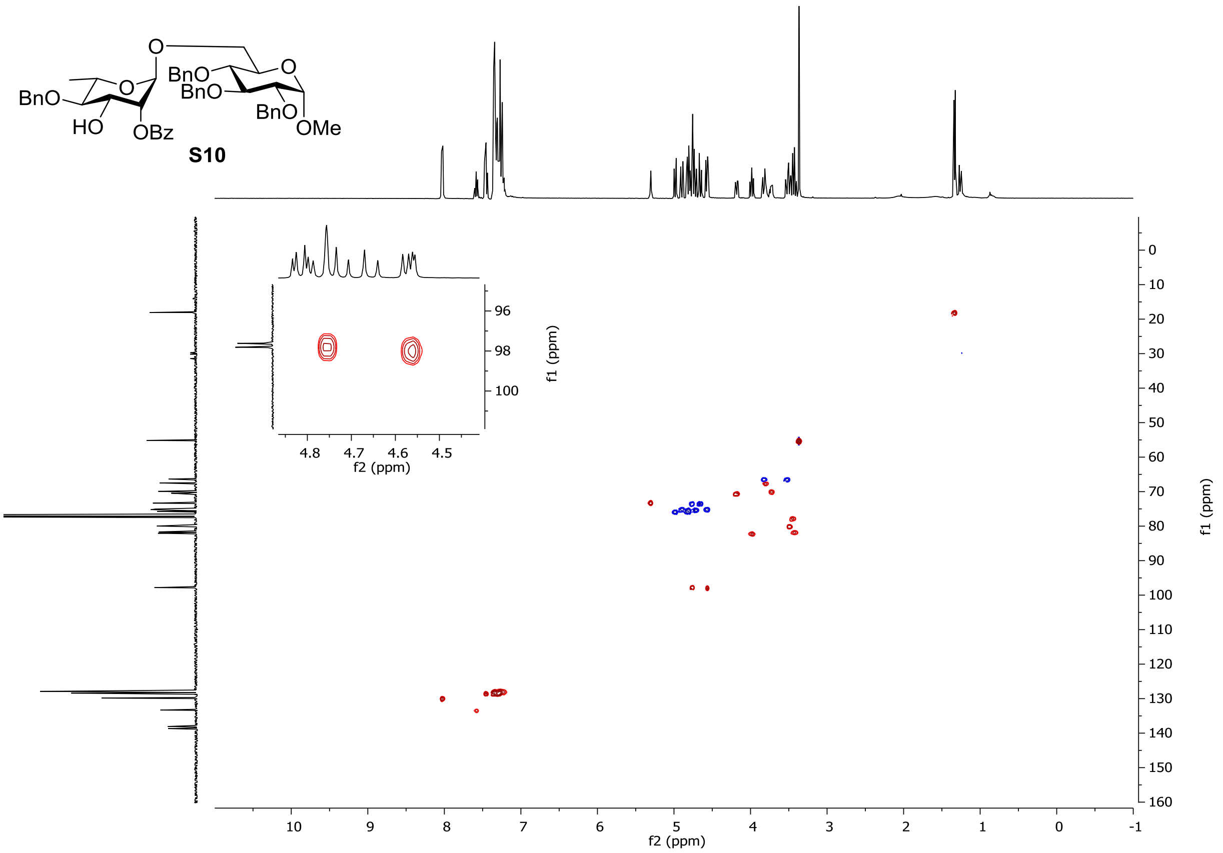

S10

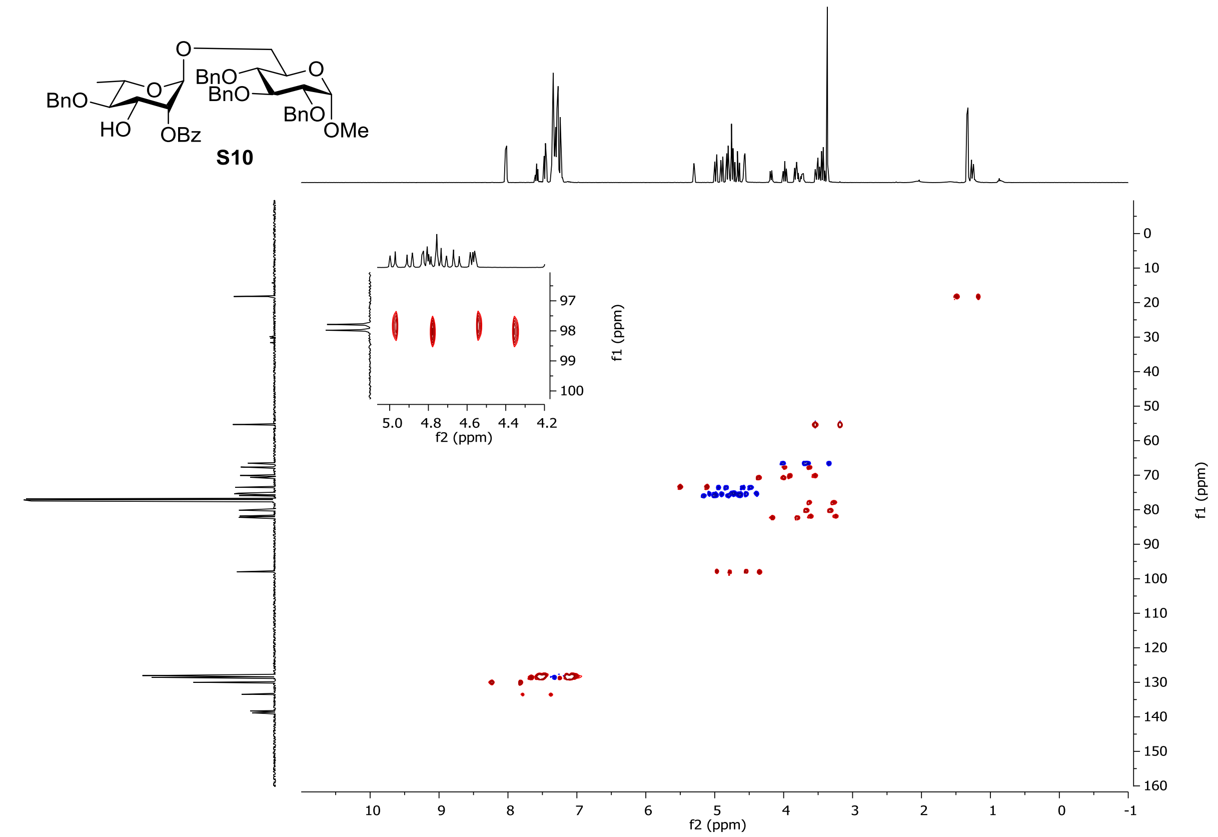

Methyl (benzyl 3,4-di-*O*-benzyl-2-*O*-levulinoyl-β-D-glucopyranosyluronate)-(1→6)-2,3,4-tri-*O*-benzyl-α-D-glucopyranoside (S11)

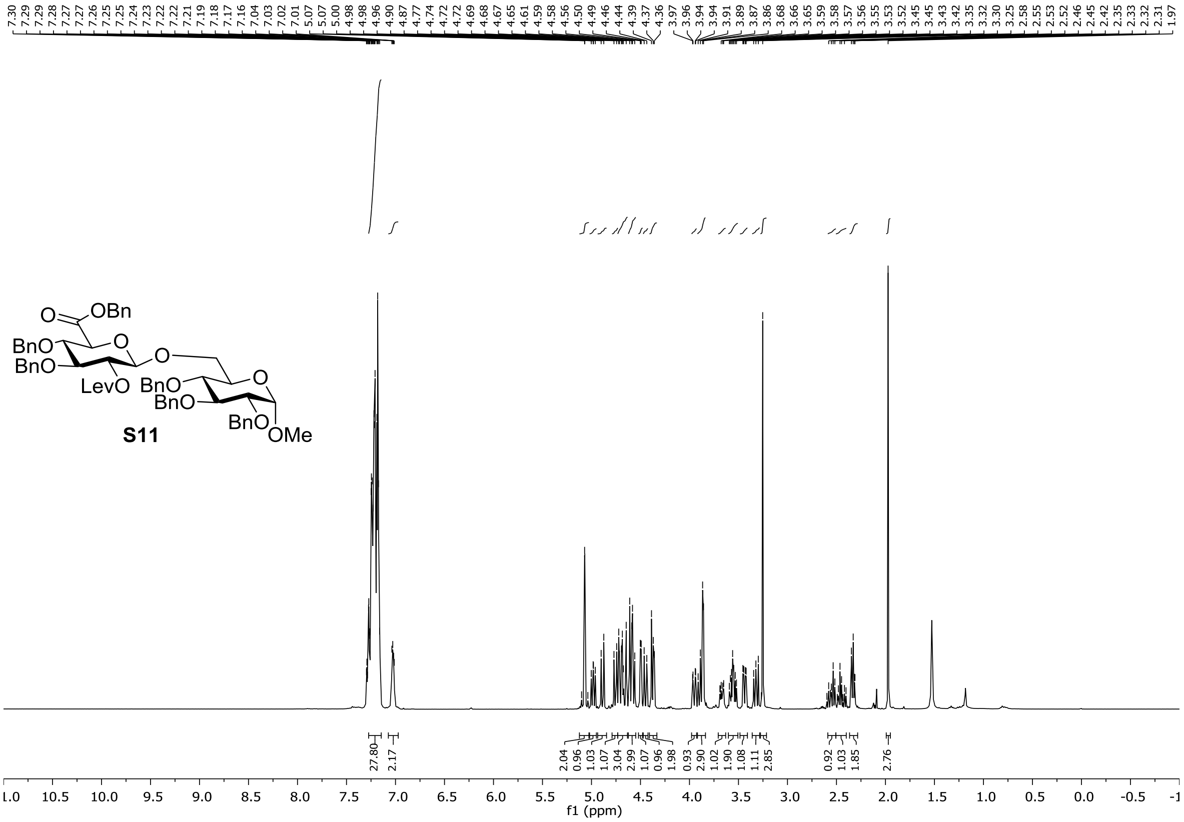

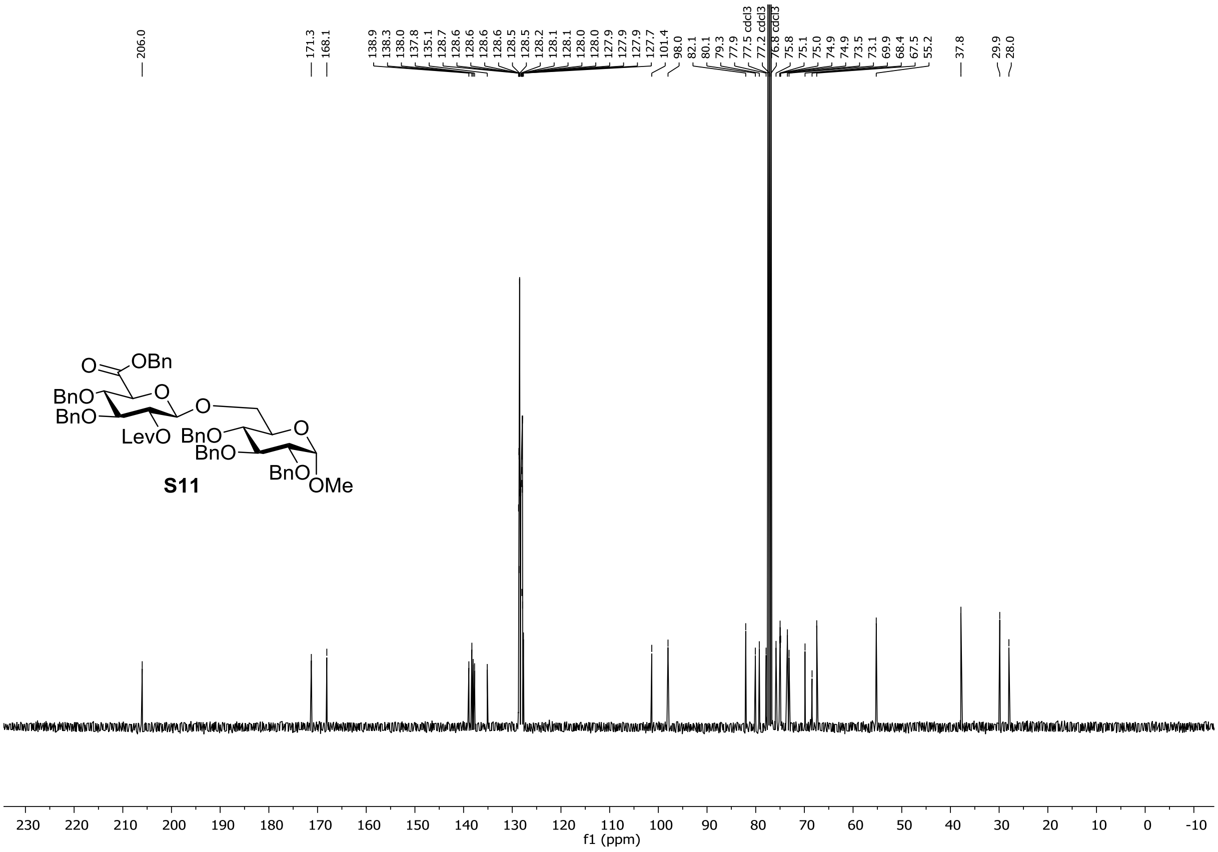

S11
f1 (ppm)

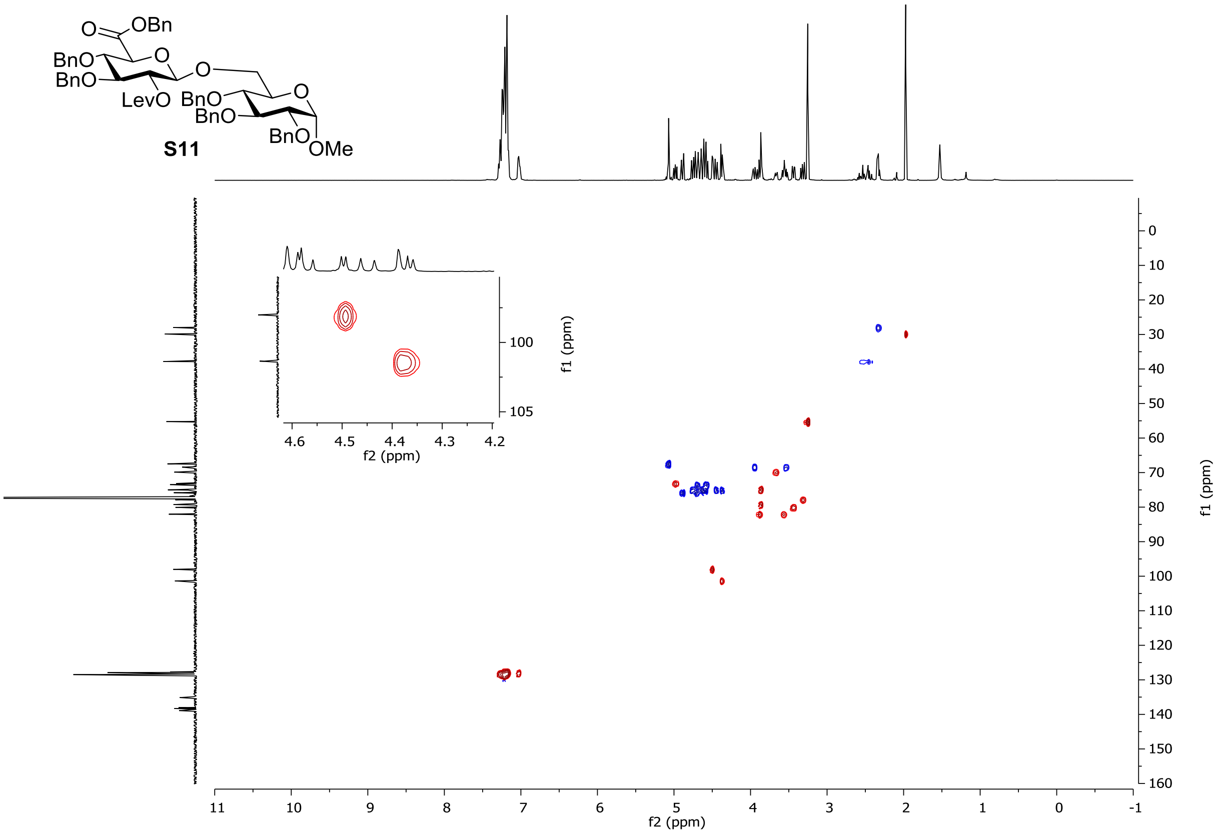

S11
f2 (ppm)
f1 (ppm)

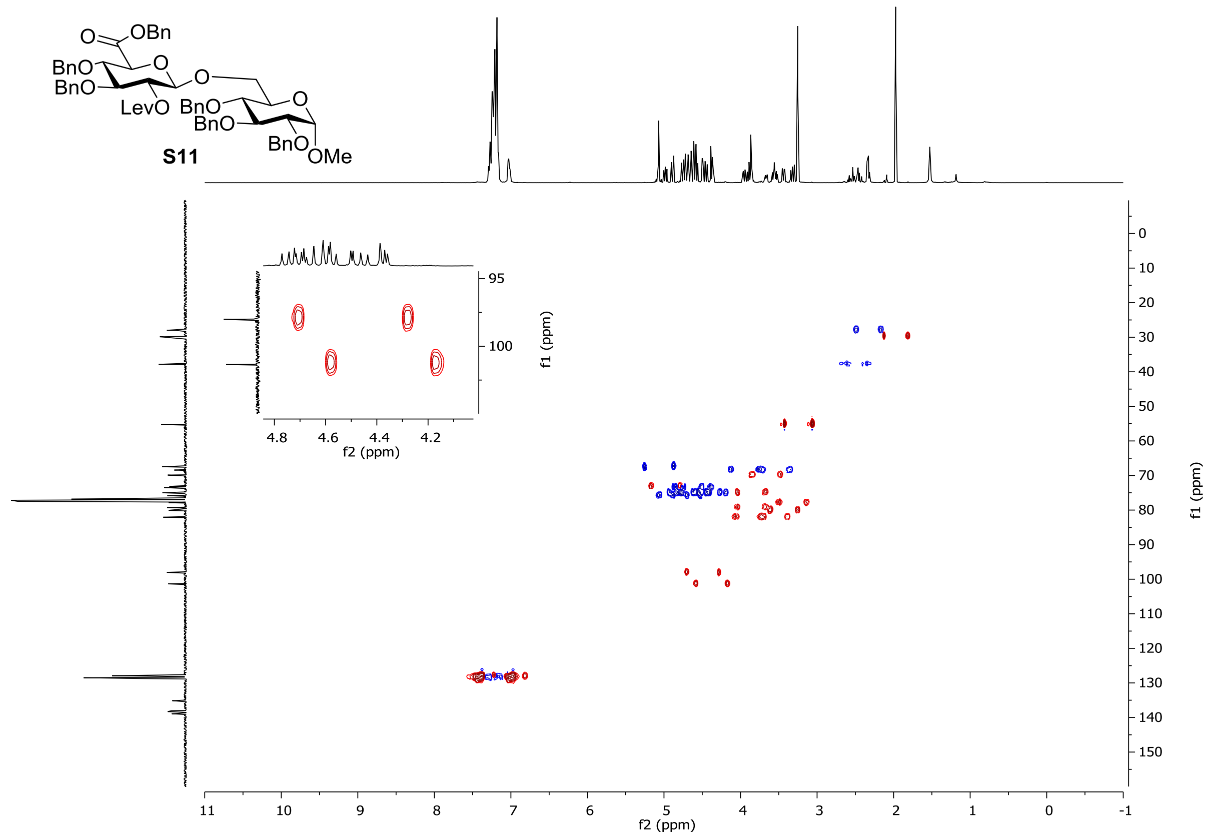

Methyl 2-*O*-benzoyl-4,6-di-*O*-benzyl-β-D-galactopyranosyl-(1→4)-2,3,6-tri-*O*-benzyl-α-D-glucopyranoside (S12)

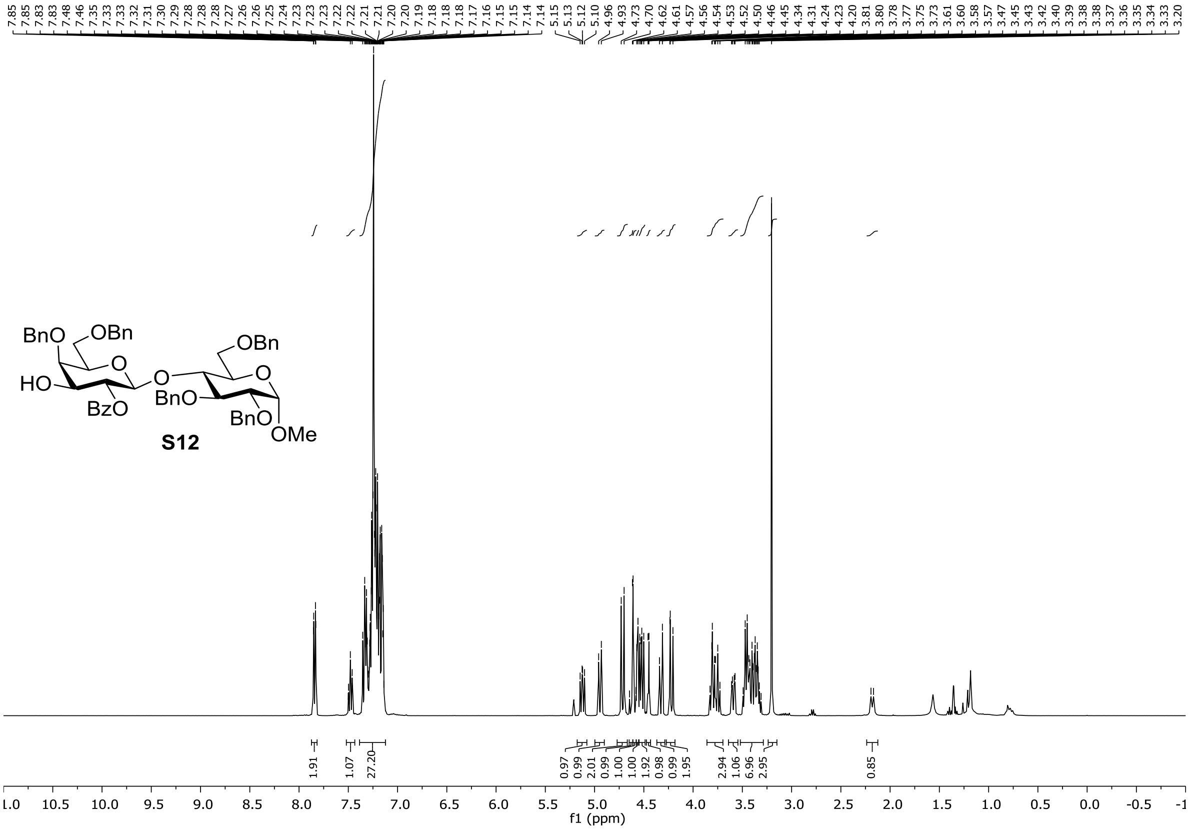

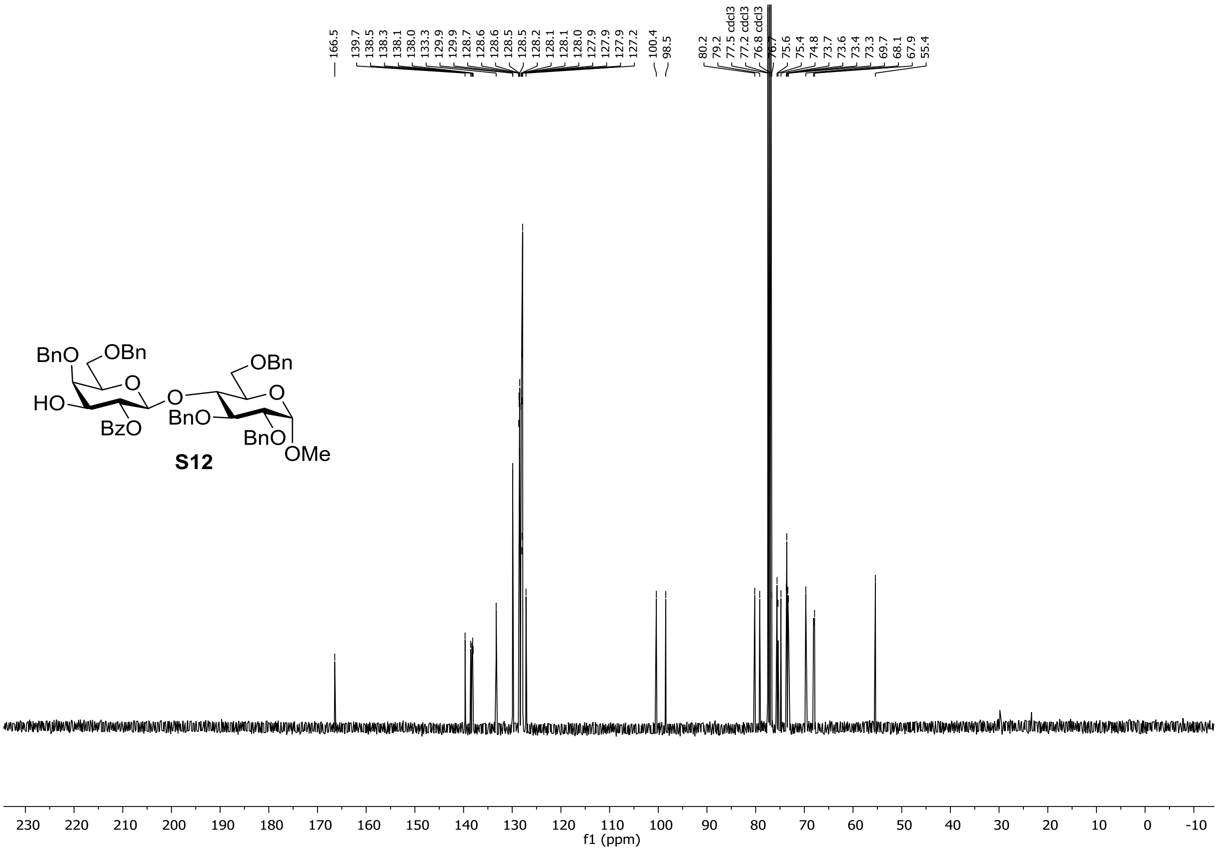
166.5
139.7
138.5
138.3
138.1
138.0
133.3
129.9
129.9
128.7
128.6
128.6
128.5
128.5
128.2
128.1
128.1
128.0
127.9
127.9
127.2
100.4
98.5
80.2
79.2
77.5 cdcl3
77.2 cdcl3
76.8 cdcl3
75.6
75.4
74.8
73.7
73.6
73.4
73.3
69.7
68.1
67.9
55.4
BnO
OBn
O
HO
BzO
O
BnO
OBn
O
BnO
OMe
S12
f1 (ppm)

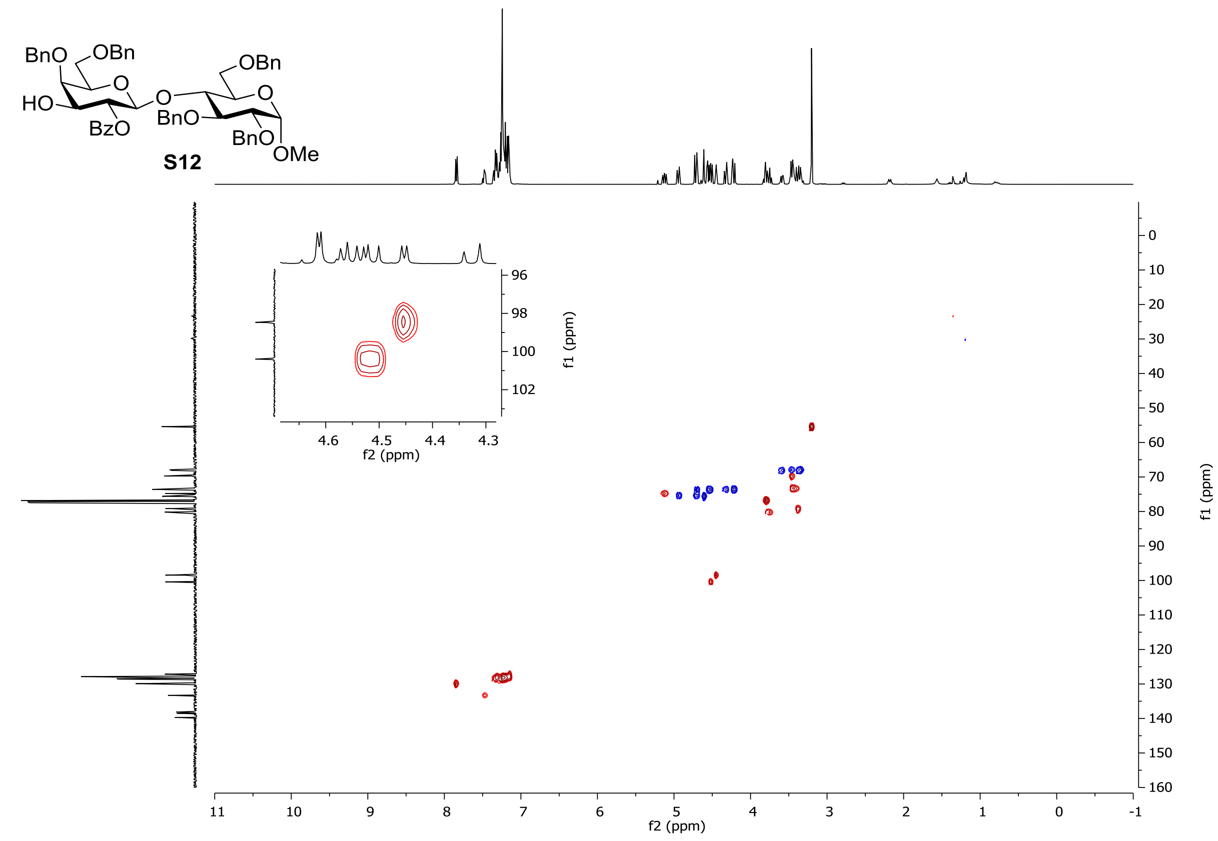
BnO
OBn
O
HO
BzO
O
BnO
OBn
O
BnO
OMe
S12
f2 (ppm)
f1 (ppm)

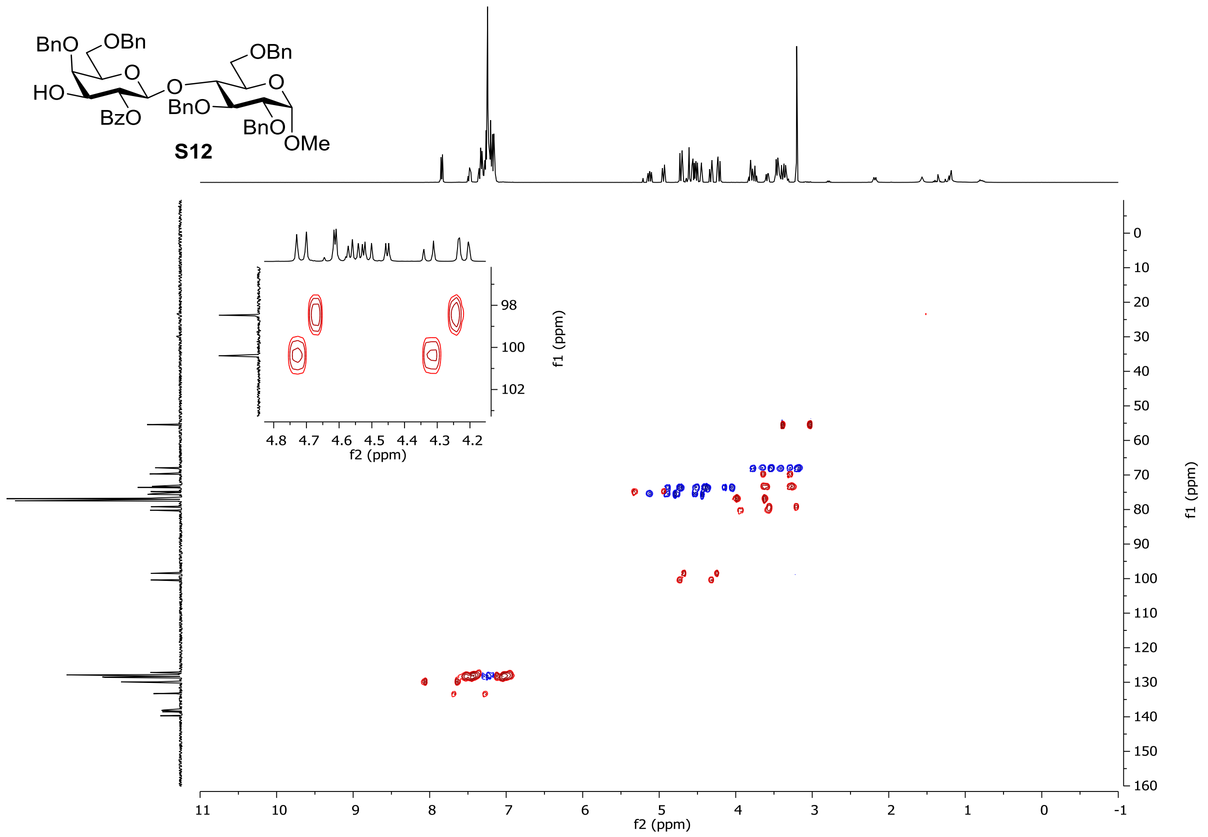

S12

Methyl 2,3-di-*O*-benzoyl-4,6-*O*-benzylidene-β-D-galactopyranosyl-(1→4)-2,3,6-tri-*O*-benzyl-α-D-glucopyranoside (S13)

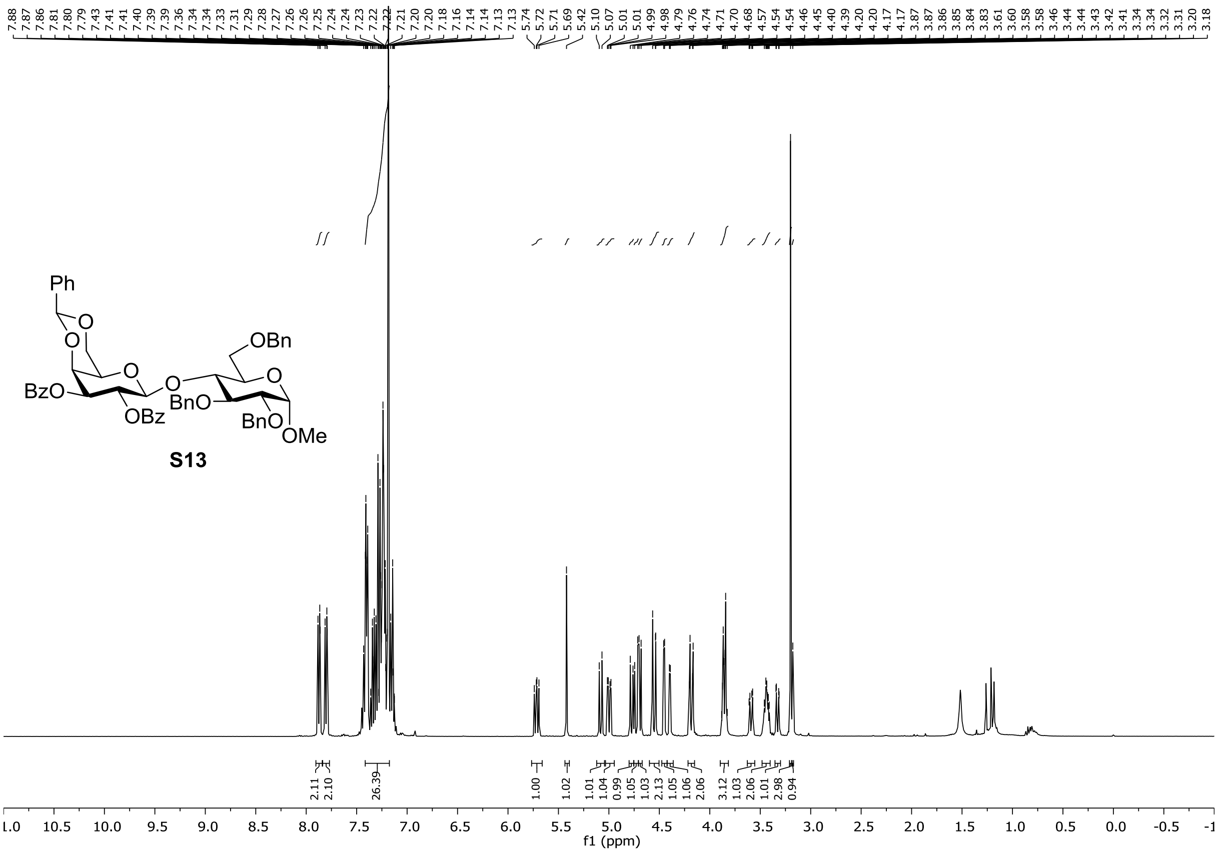

S13

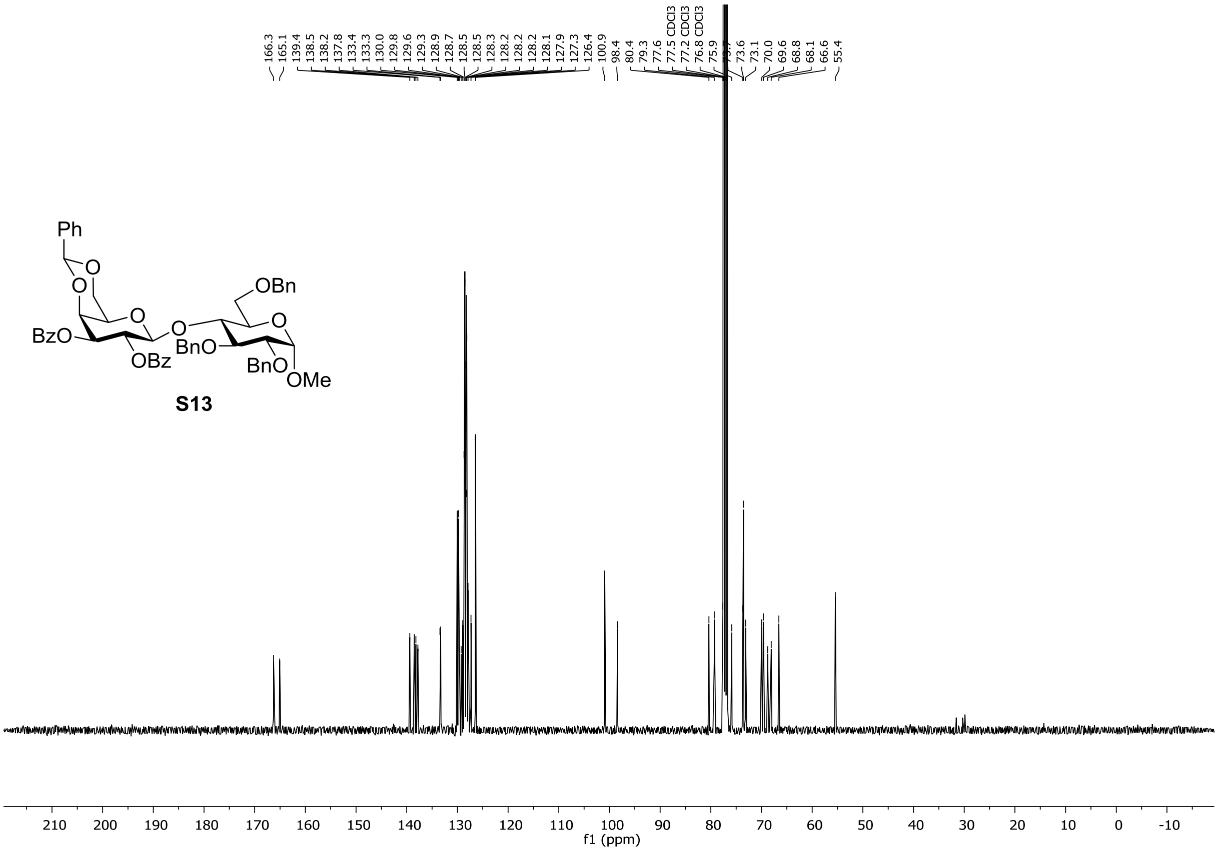
S13
166.3
165.1
139.4
138.5
138.2
137.8
133.4
133.3
130.0
129.8
129.6
129.3
128.9
128.7
128.5
128.5
128.3
128.2
128.2
128.2
128.1
127.9
127.3
126.4
100.9
98.4
80.4
79.3
77.6
77.5 CDCl3
77.2 CDCl3
76.8 CDCl3
75.9
73.6
73.1
70.0
69.6
68.8
68.1
66.6
55.4
f1 (ppm)

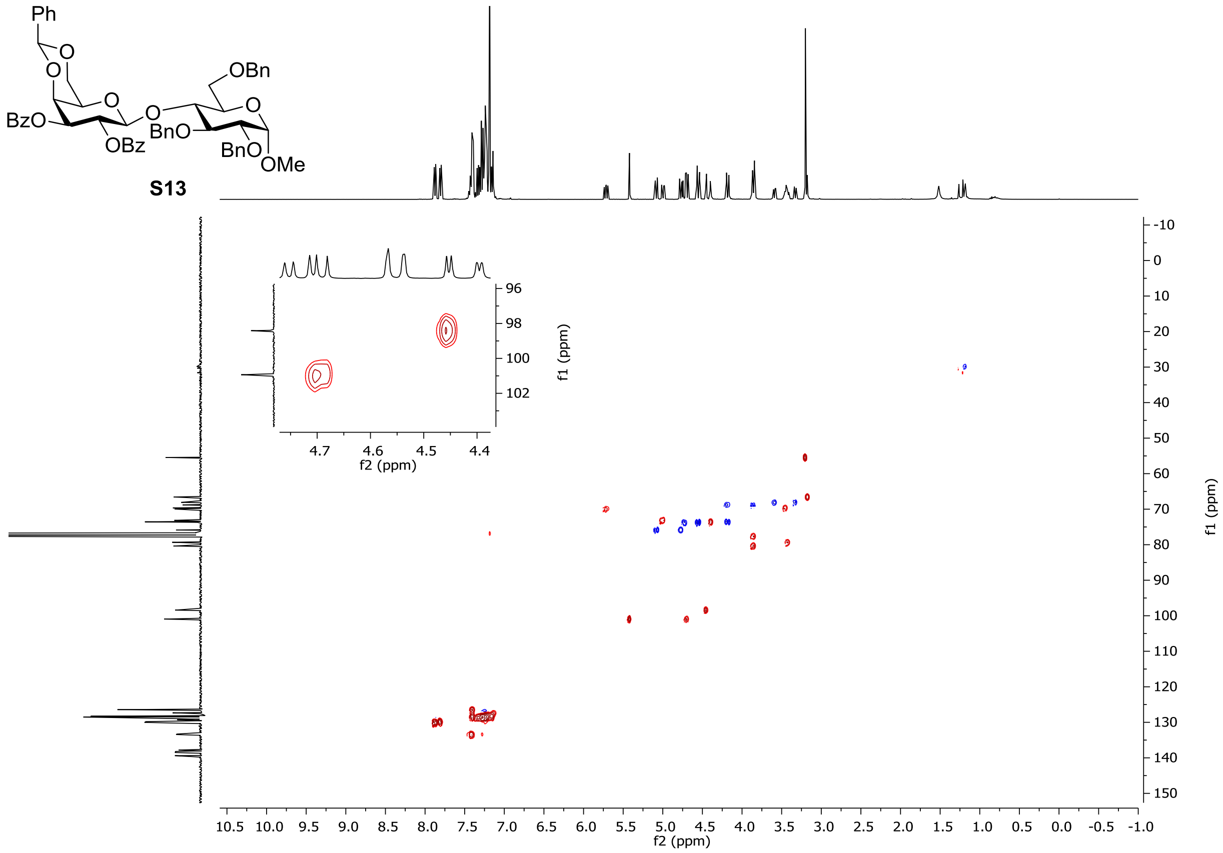
S13
f2 (ppm)
f1 (ppm)

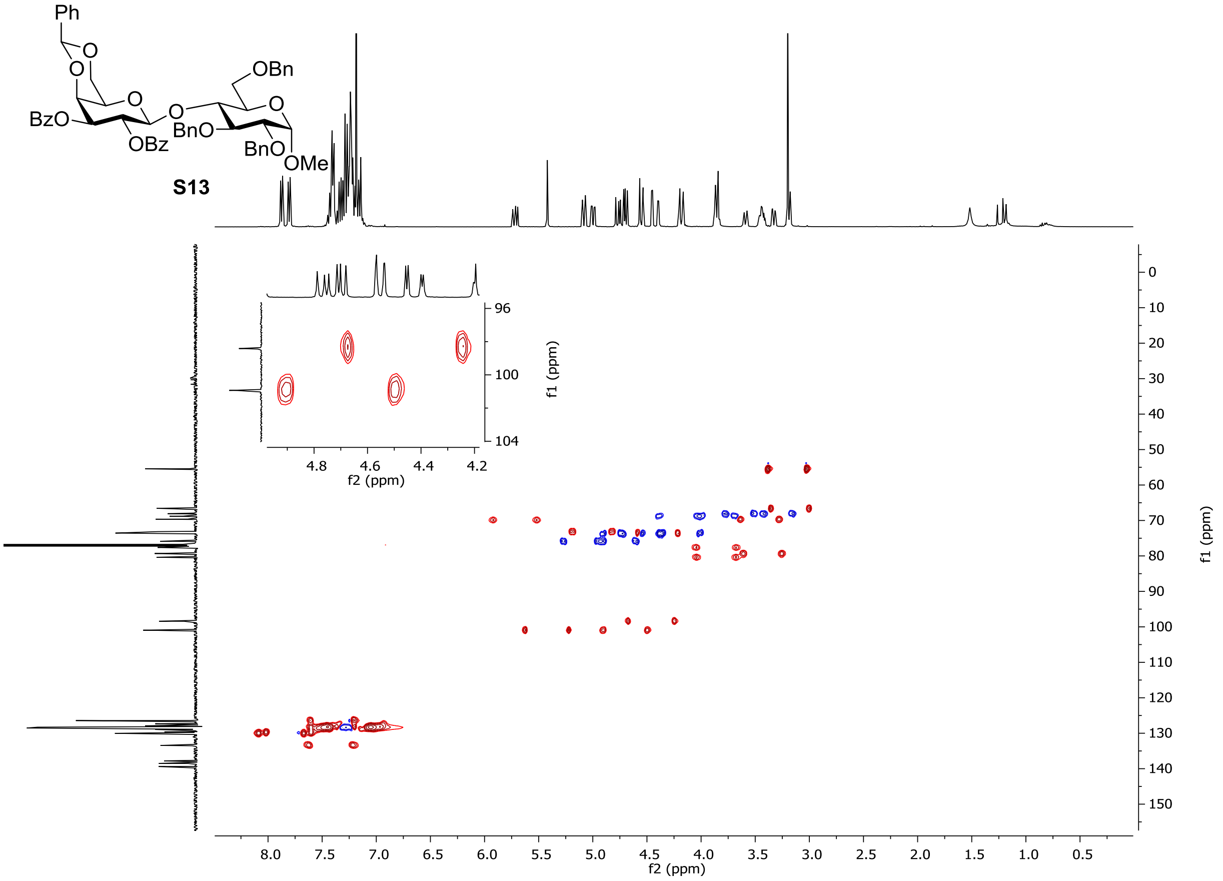

Methyl 2,3-di-*O*-benzoyl-6-*O*-benzyl-β-D-glucopyranosyl-(1→4)-2,3,6-tri-*O*-benzyl-α-D-glucopyranoside (S14)

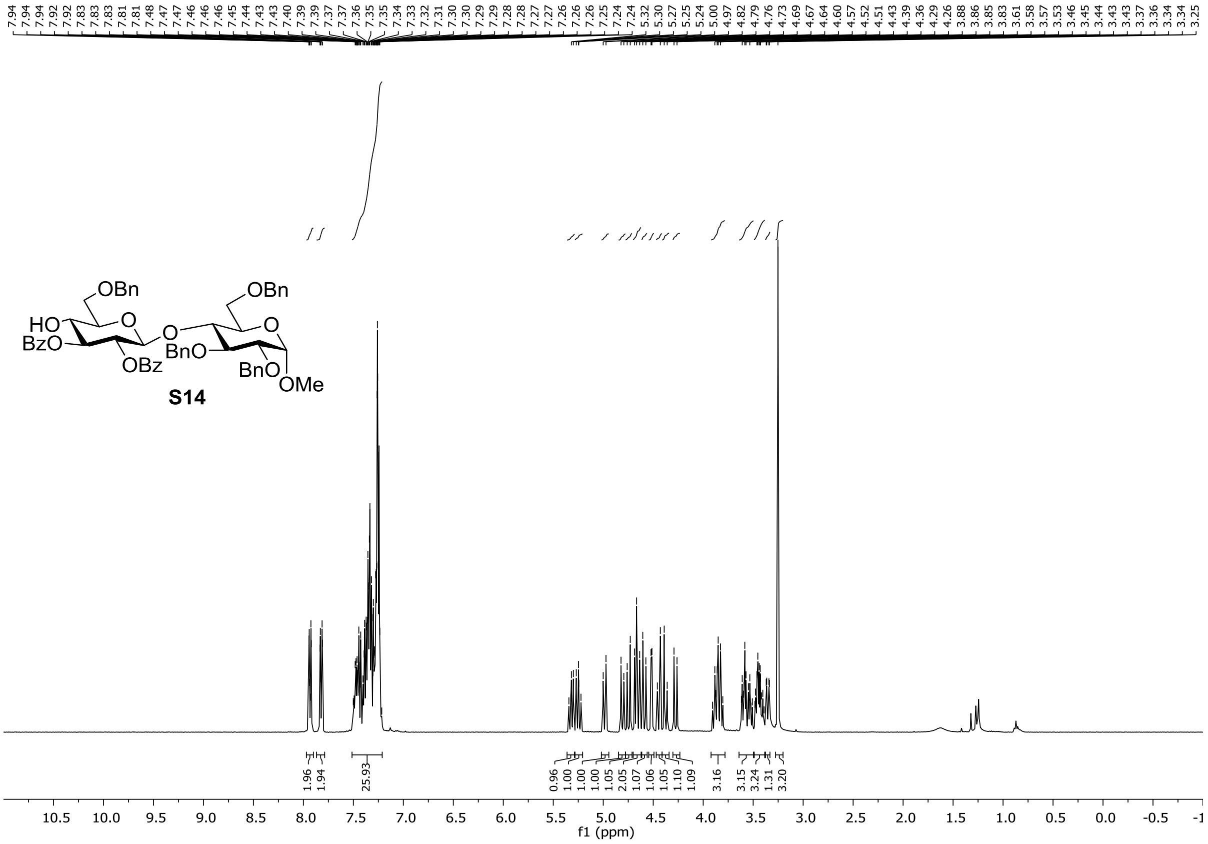

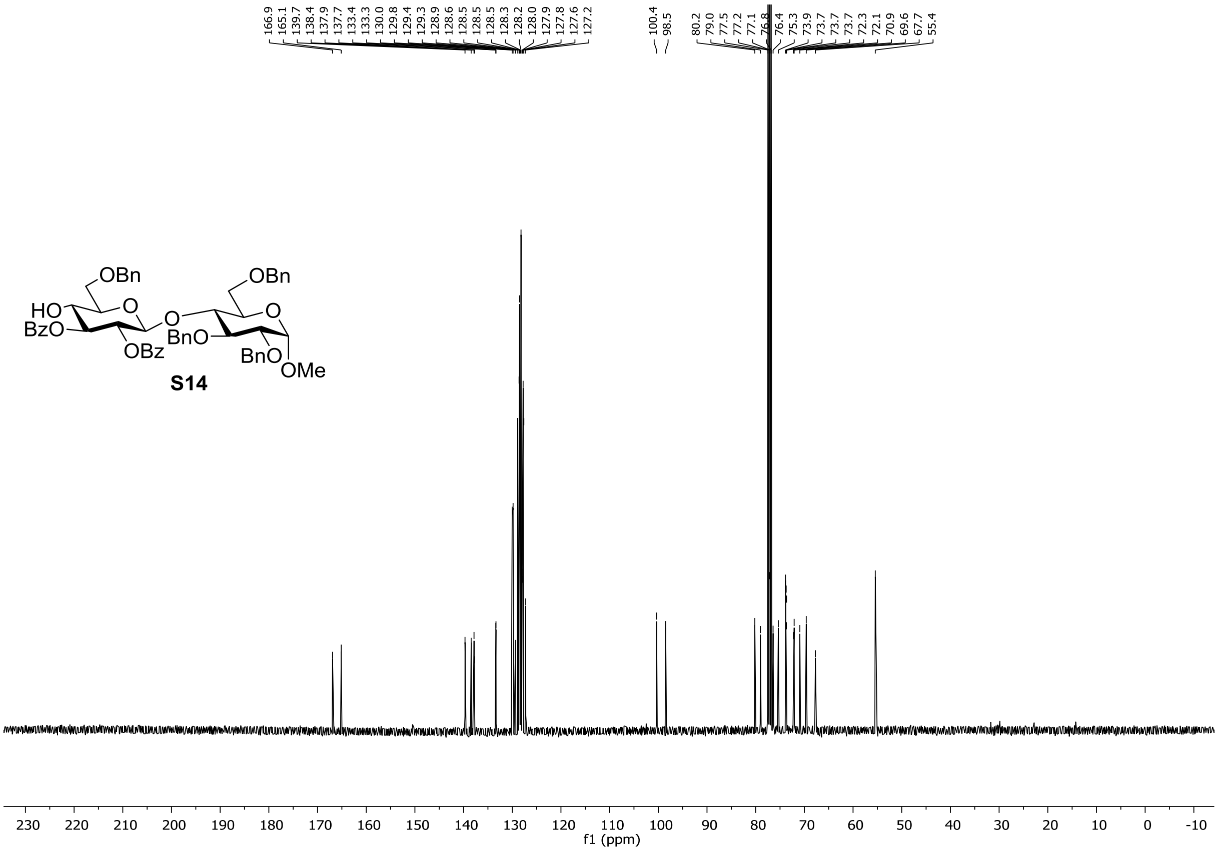

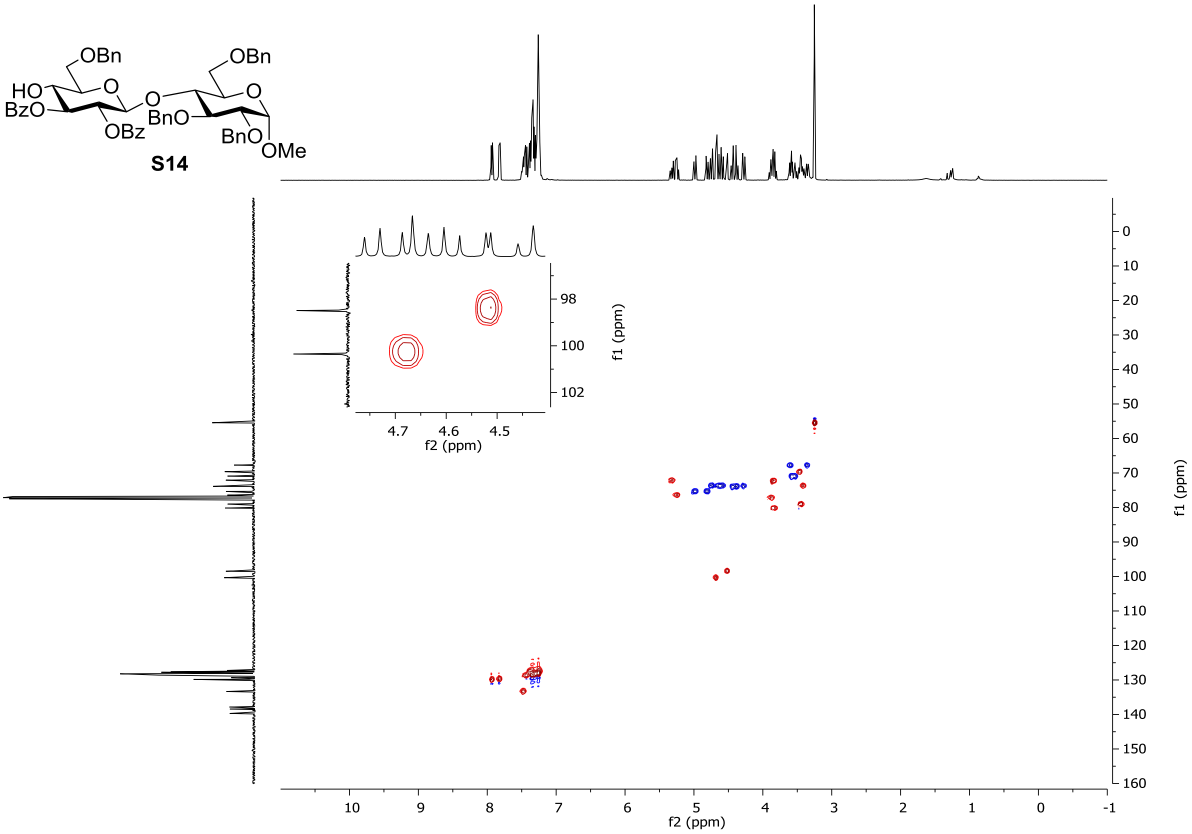

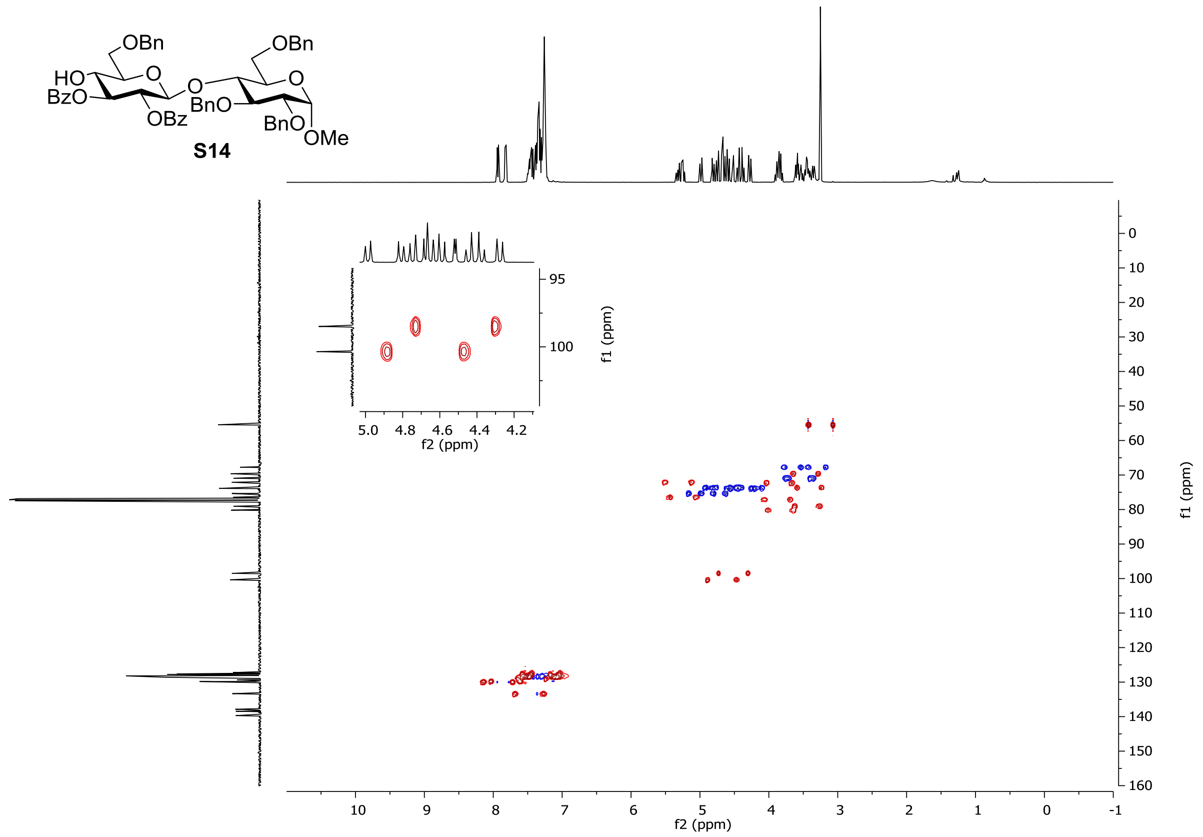

Methyl 3,4,6-tri-*O*-benzyl-2-*O*-levulinoyl-β-D-glucopyranosyl-(1→4)-2,3,6-tri-*O*-benzyl-α-D-glucopyranoside (S15)

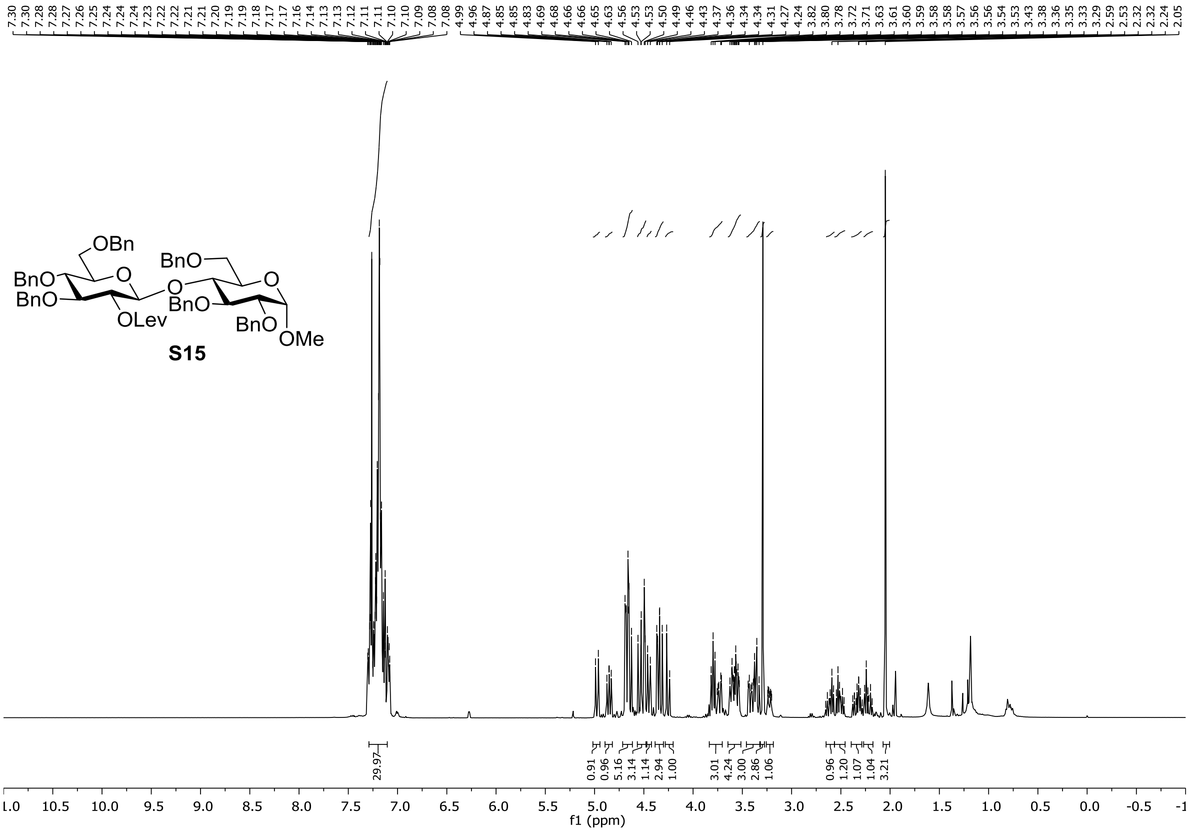

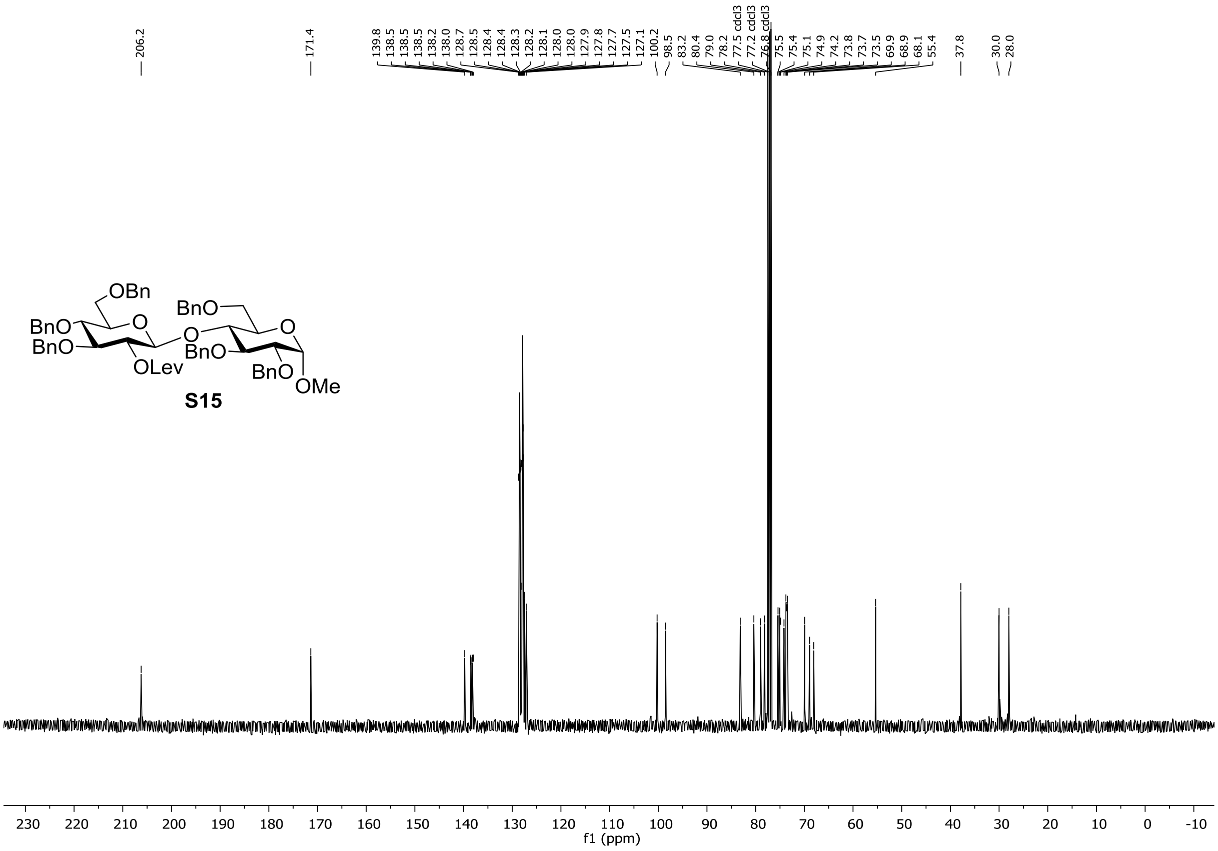
206.2
171.4
139.8
138.5
138.5
138.5
138.2
138.0
128.7
128.5
128.4
128.4
128.3
128.2
128.1
128.0
128.0
127.9
127.8
127.7
127.5
127.1
100.2
98.5
83.2
80.4
79.0
78.2
77.5 cdcl3
77.2 cdcl3
76.8 cdcl3
75.5
75.4
75.1
74.9
74.2
73.8
73.7
73.5
69.9
68.9
68.1
55.4
37.8
30.0
28.0
OBn
BnO
BnO
O
O
BnO
O
BnO
OLev
BnO
OMe
S15
f1 (ppm)

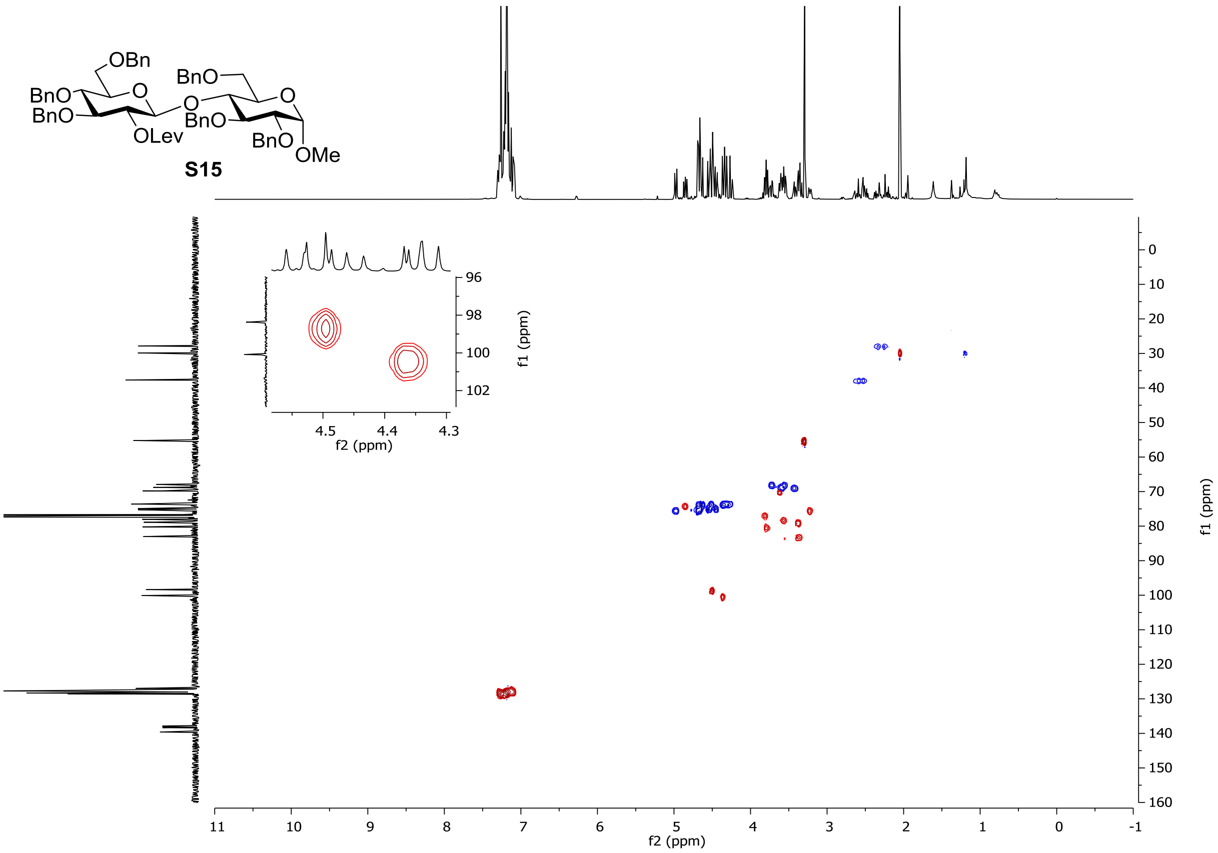
OBn
BnO
BnO
O
O
BnO
O
BnO
OLev
BnO
OMe
S15
f1 (ppm)
f2 (ppm)

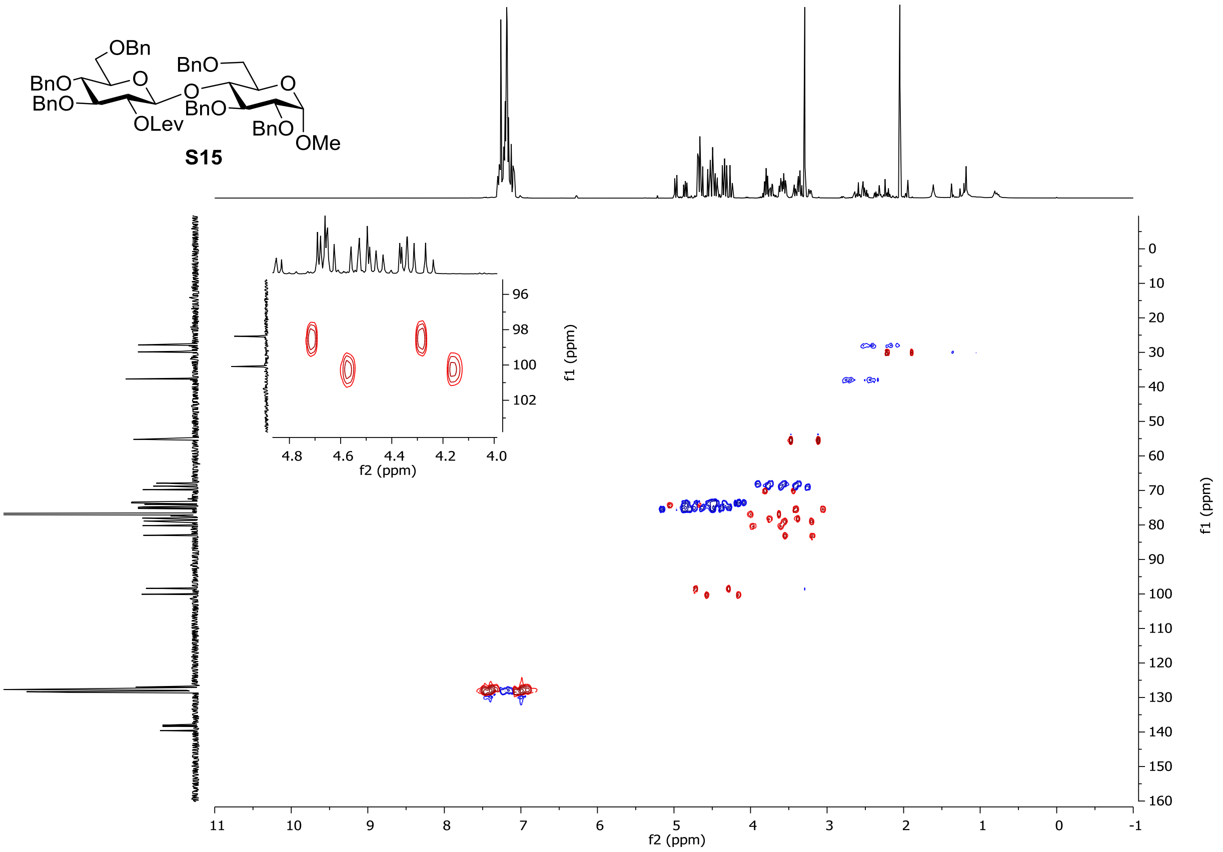

Methyl 3-*O*-benzyl-6-*O*-levulinoyl-2-*N*-trichloroacetyl-β-D-glucosaminopyranosyl-(1→4)-2,3,6-tri-*O*-benzyl-α-D-glucopyranoside (S16)

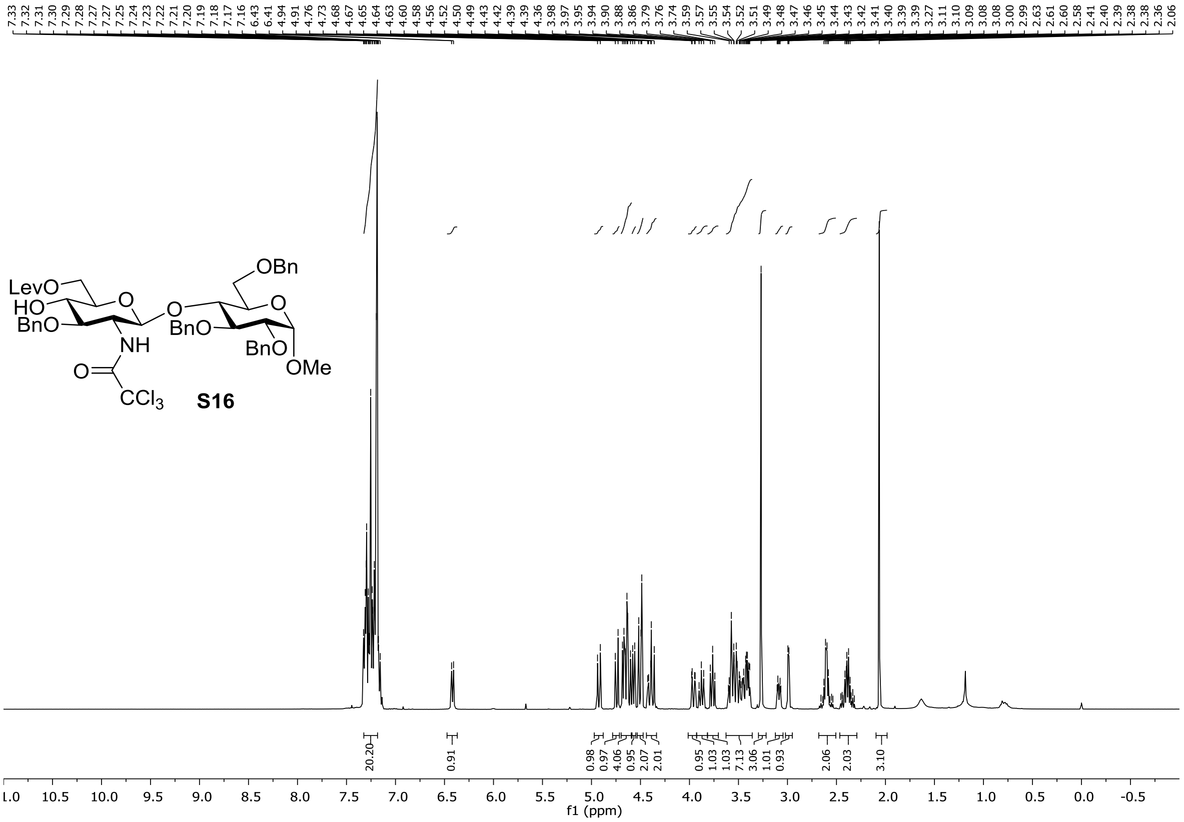

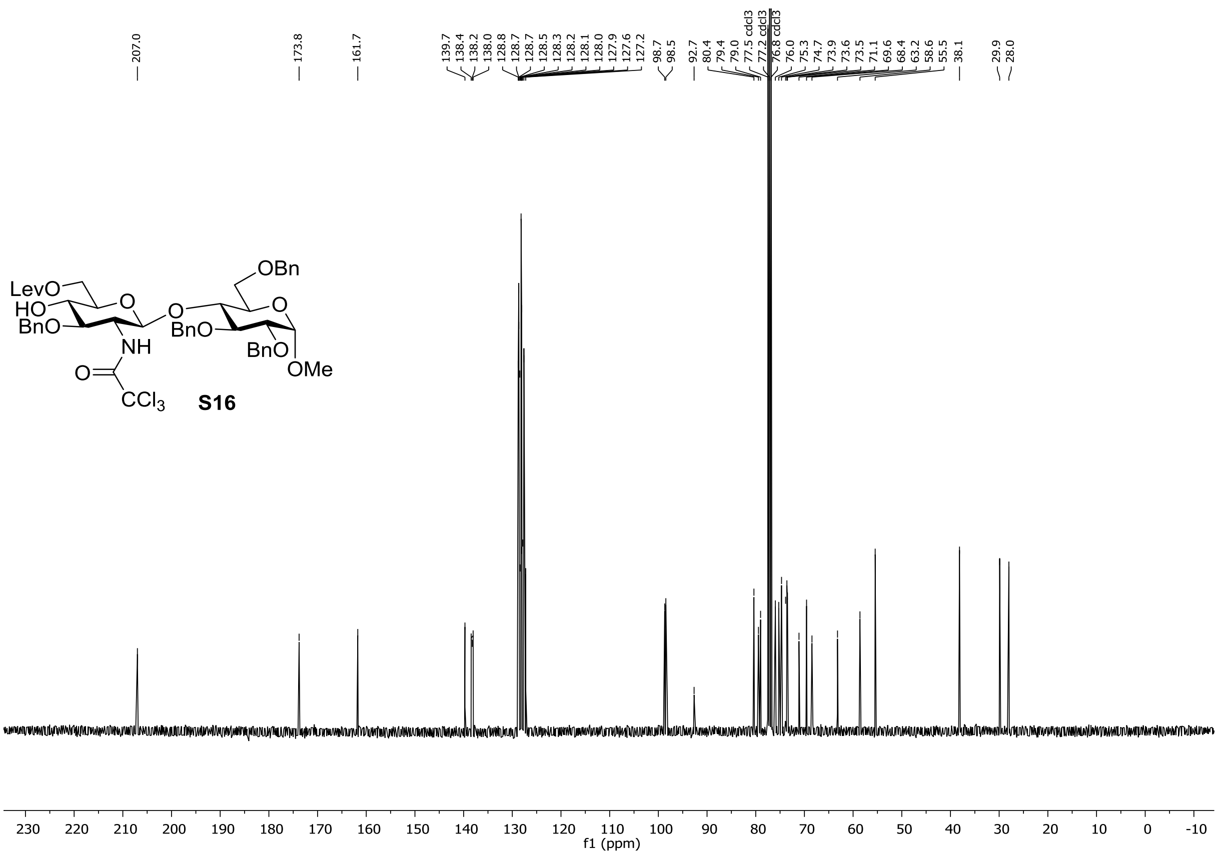

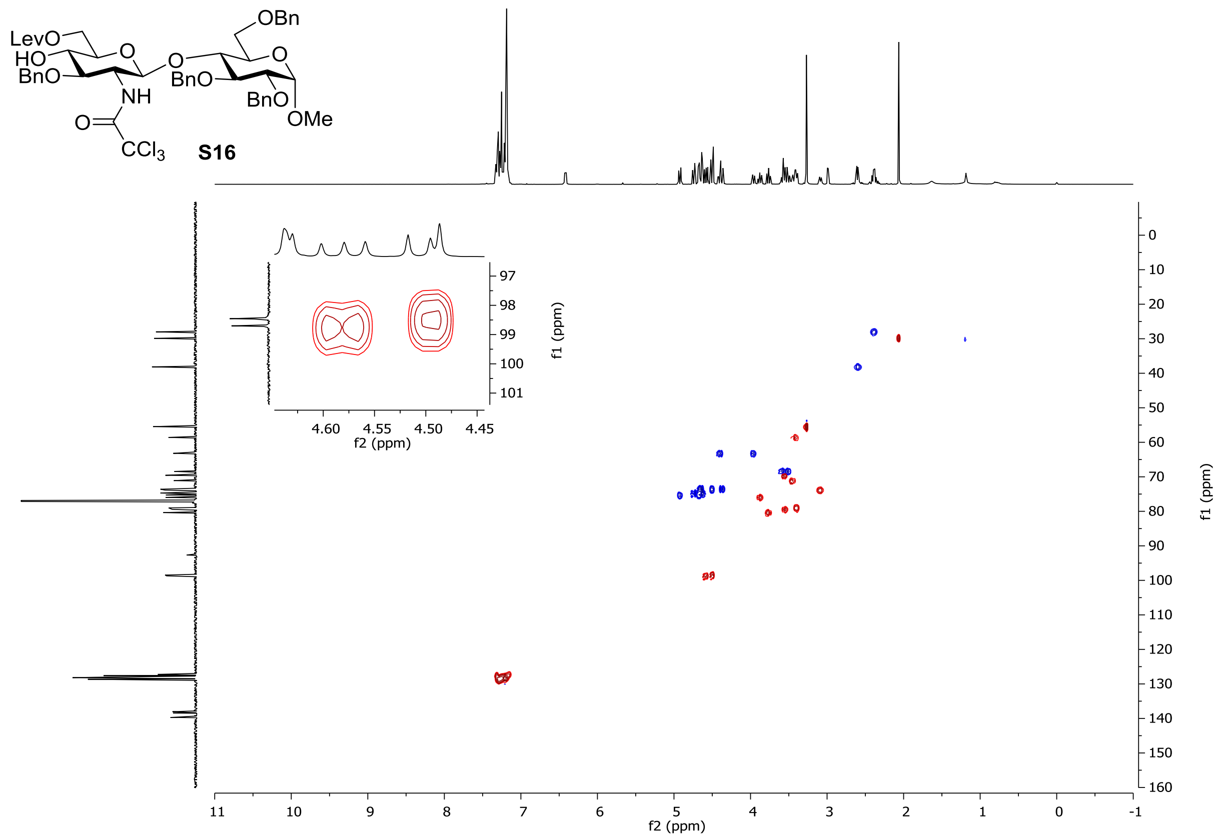

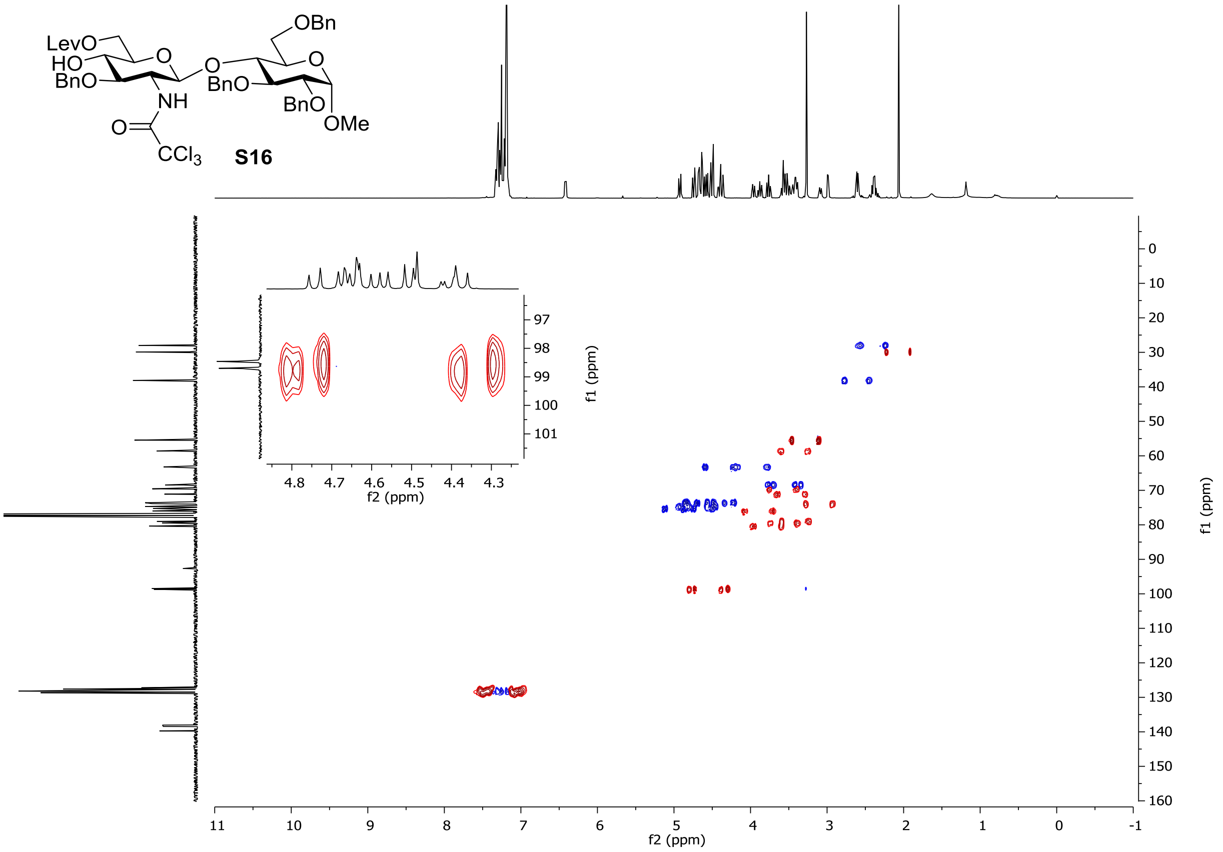

Methyl 2-*O*-benzoyl-3,4-di-*O*-benzyl-α-D-mannopyranosyl-(1→4)-2,3,6-tri-*O*-benzyl-α-D-glucopyranoside (S17)

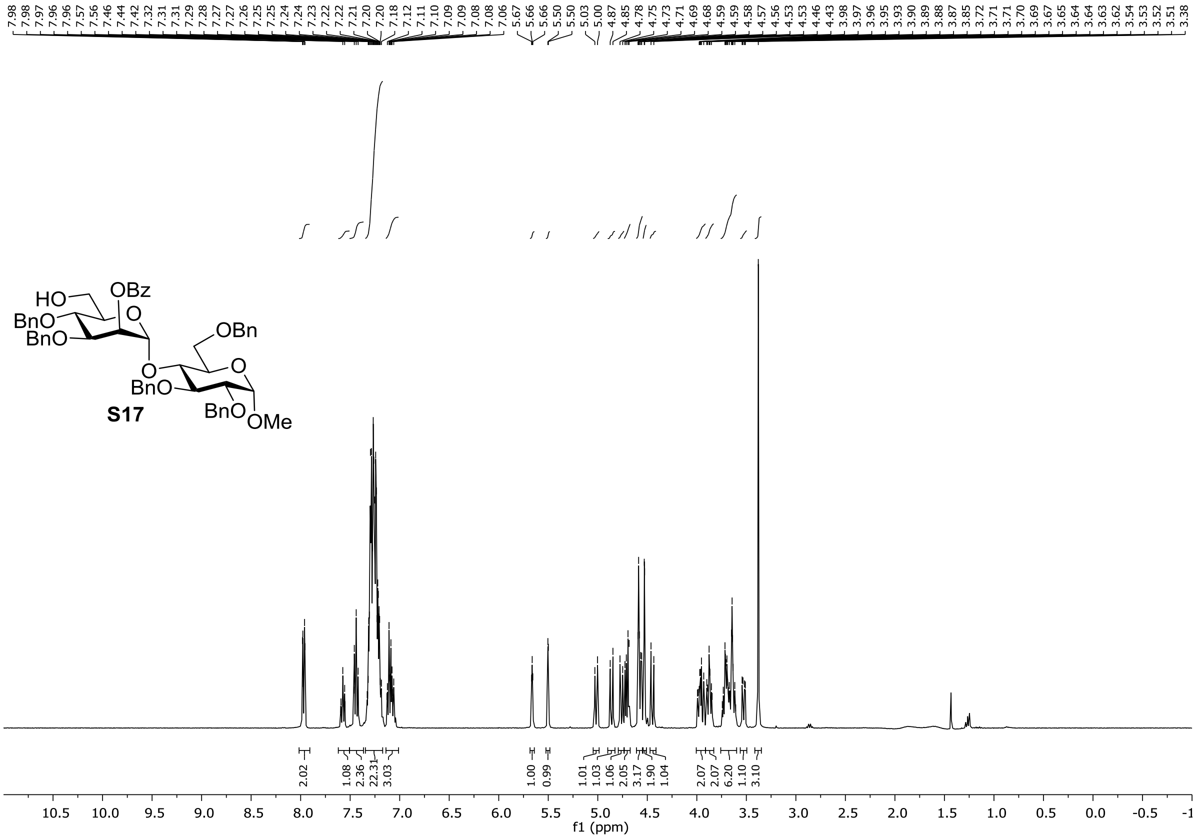

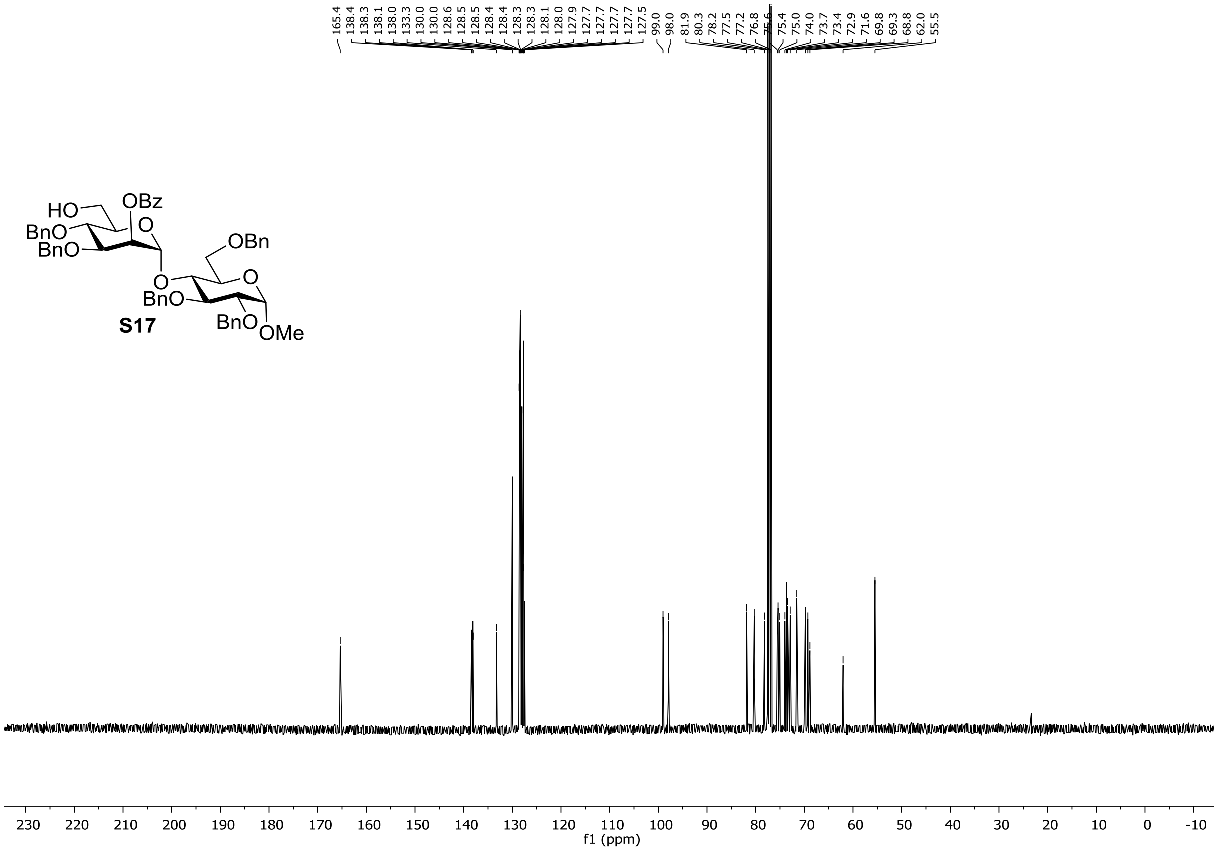
165.4
138.4
138.3
138.1
138.0
133.3
130.0
130.0
128.6
128.5
128.5
128.4
128.3
128.3
128.3
128.1
128.0
127.9
127.7
127.7
127.7
127.7
127.5
99.0
98.0
81.9
80.3
78.2
77.5
77.2
76.8
[illegible]
75.4
75.0
74.0
73.7
73.4
72.9
71.6
69.8
69.3
68.8
62.0
55.5
HO
OBz
BnO
BnO
O
OBn
O
BnO
BnO
OMe
S17
f1 (ppm)

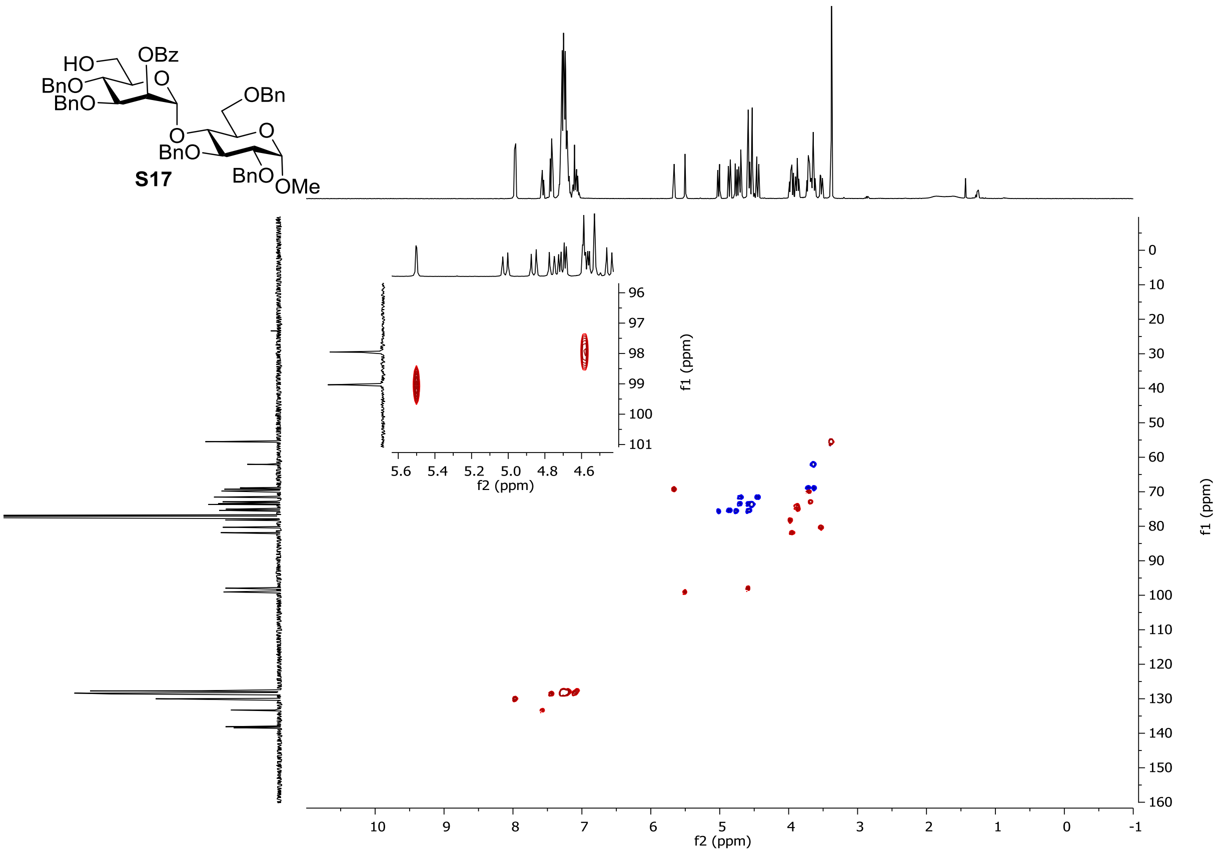
HO
OBz
BnO
BnO
O
OBn
O
BnO
BnO
OMe
S17
f1 (ppm)
f2 (ppm)
f1 (ppm)
f2 (ppm)

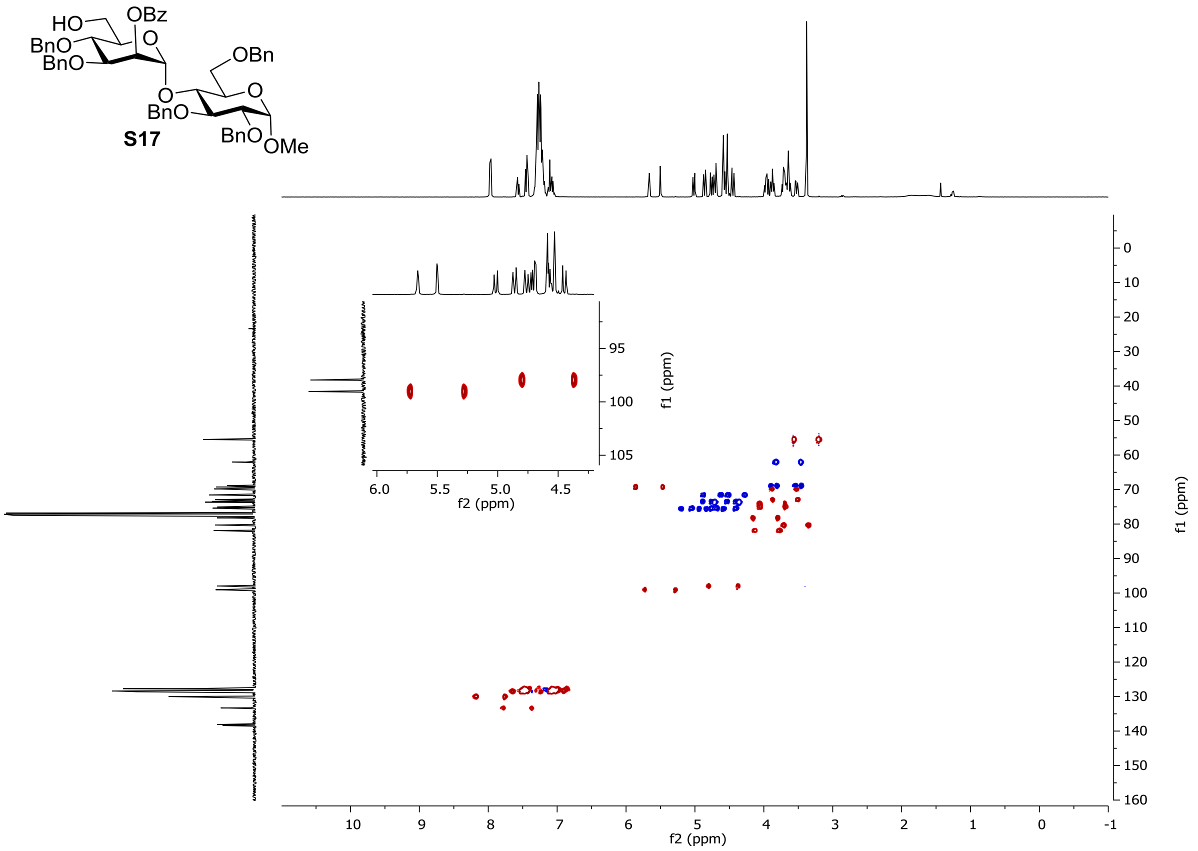

Methyl 2-*O*-benzoyl-4-*O*-benzyl-α-L-rhamnopyranosyl-(1→4)-2,3,6-tri-*O*-benzyl-α-D-glucopyranoside (S18)

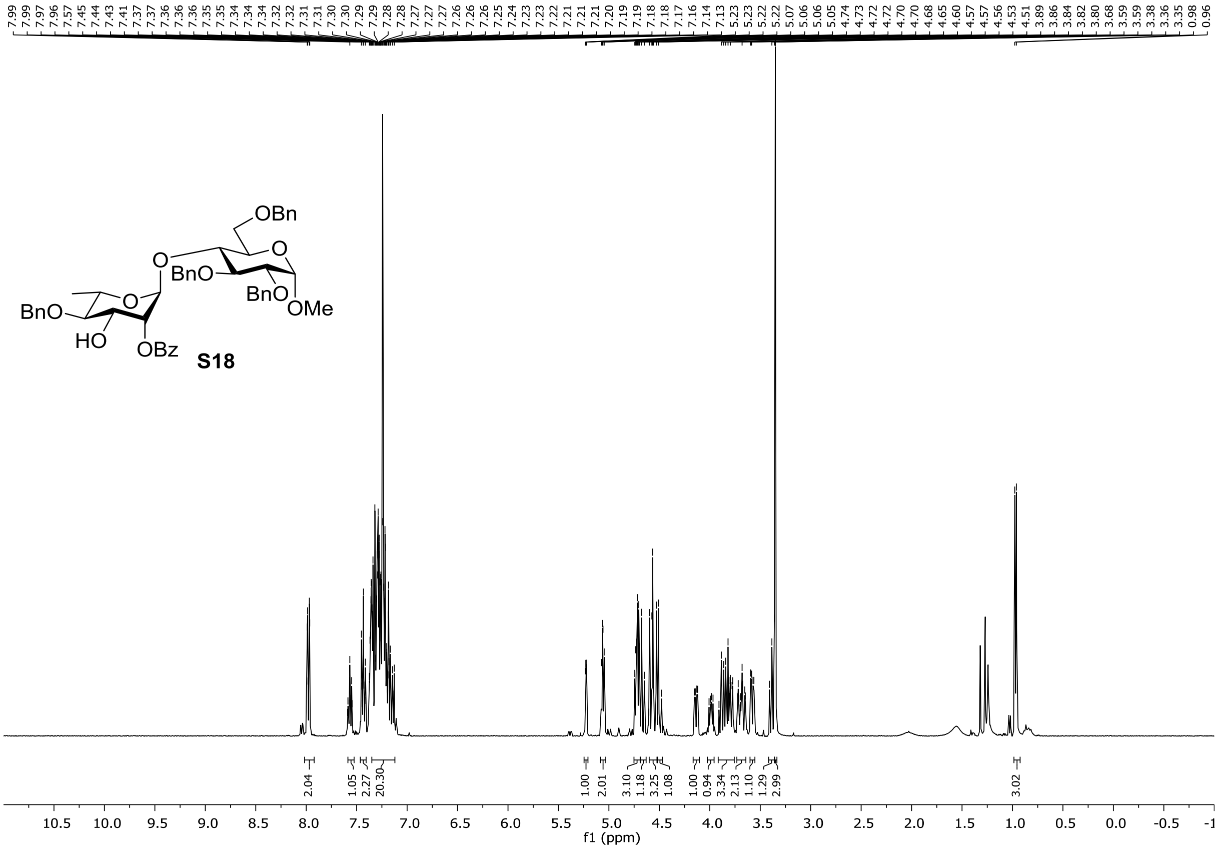

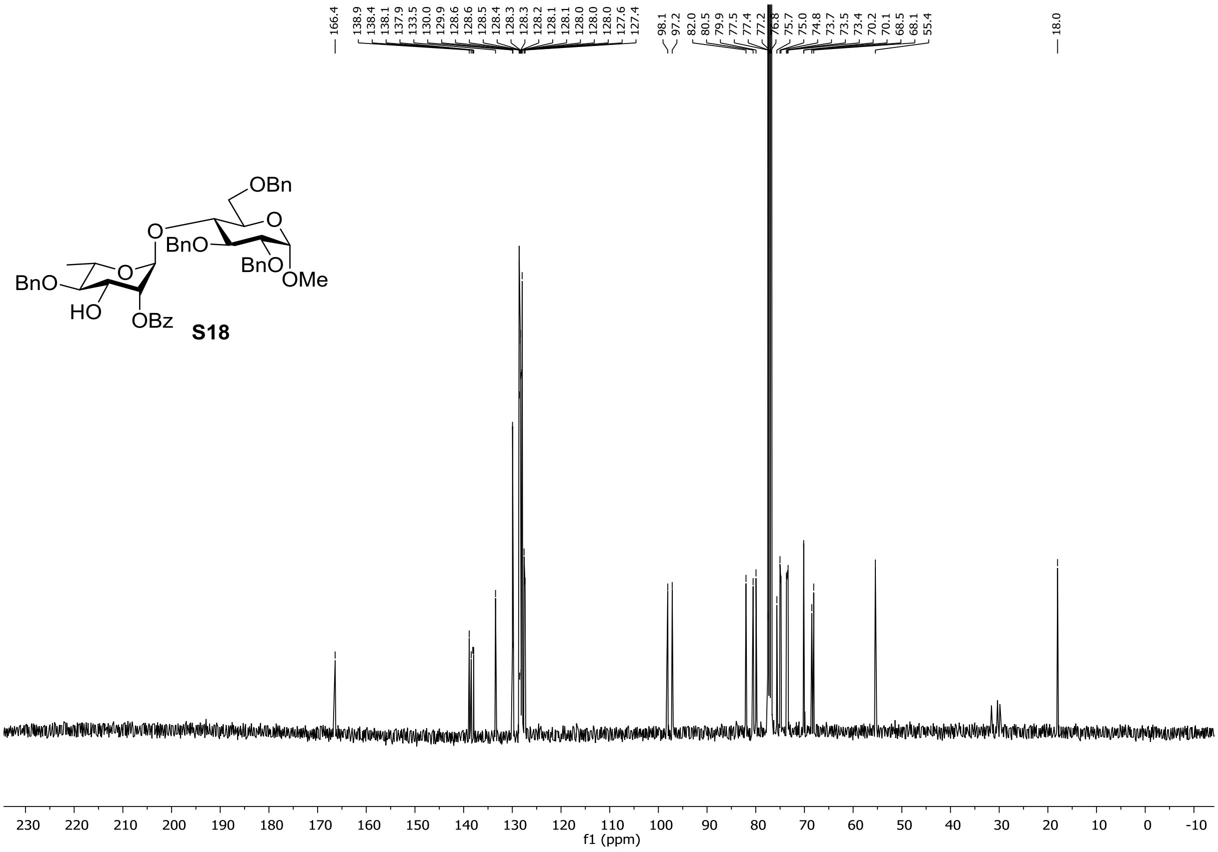
166.4
138.9
138.4
138.1
137.9
133.5
130.0
129.9
128.6
128.5
128.4
128.3
128.3
128.2
128.1
128.1
128.0
128.0
127.6
127.4
98.1
97.2
82.0
80.5
79.9
77.5
77.4
77.2
76.8
75.7
75.0
74.8
73.7
73.5
73.4
70.2
70.1
68.5
68.1
55.4
18.0
OBn
O
O
BnO
BnO
OMe
BnO
O
HO
OBz
S18
f1 (ppm)

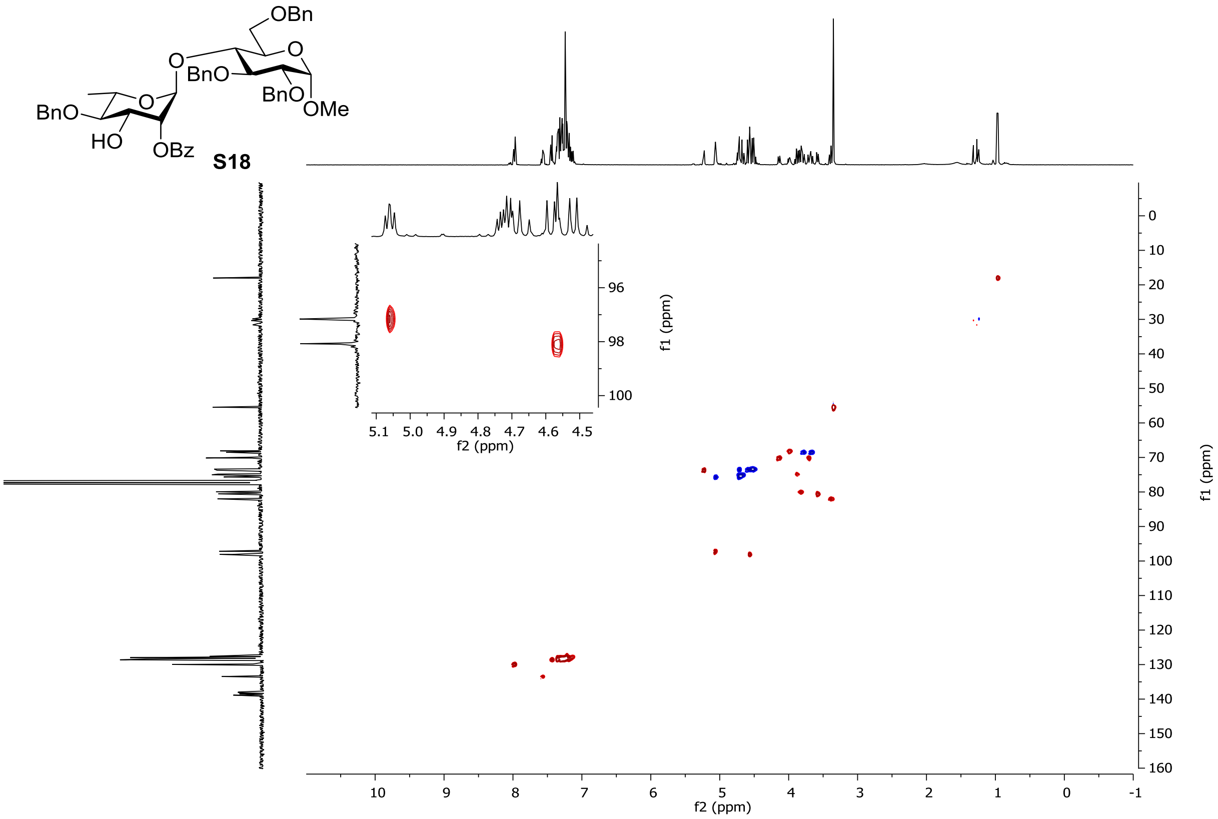
OBn
O
O
BnO
BnO
OMe
BnO
O
HO
OBz
S18
f1 (ppm)
f2 (ppm)

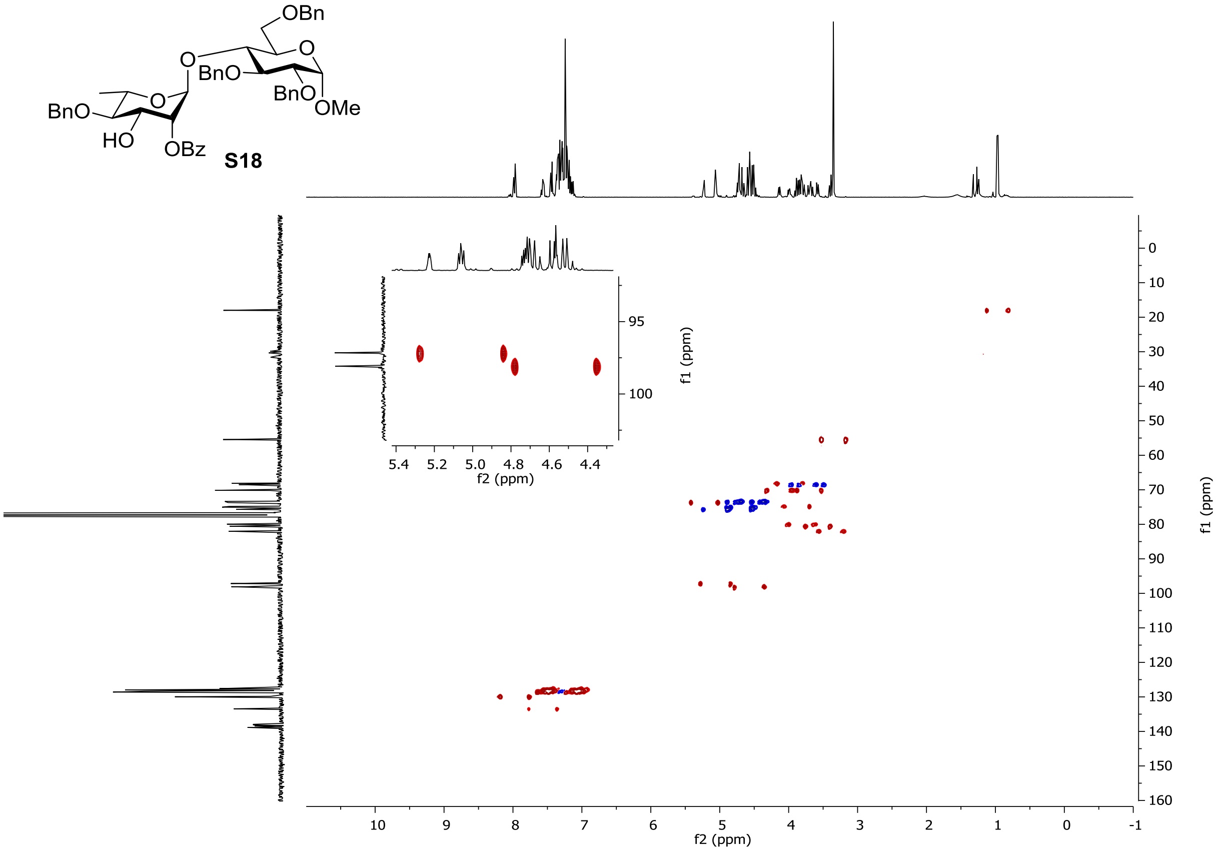

Methyl (benzyl 3,4-di-*O*-benzyl-2-*O*-levulinoyl-β-D-glucopyranosyluronate)-(1→4)-2,3,6-tri-*O*-benzyl-α-D-glucopyranoside (S19)

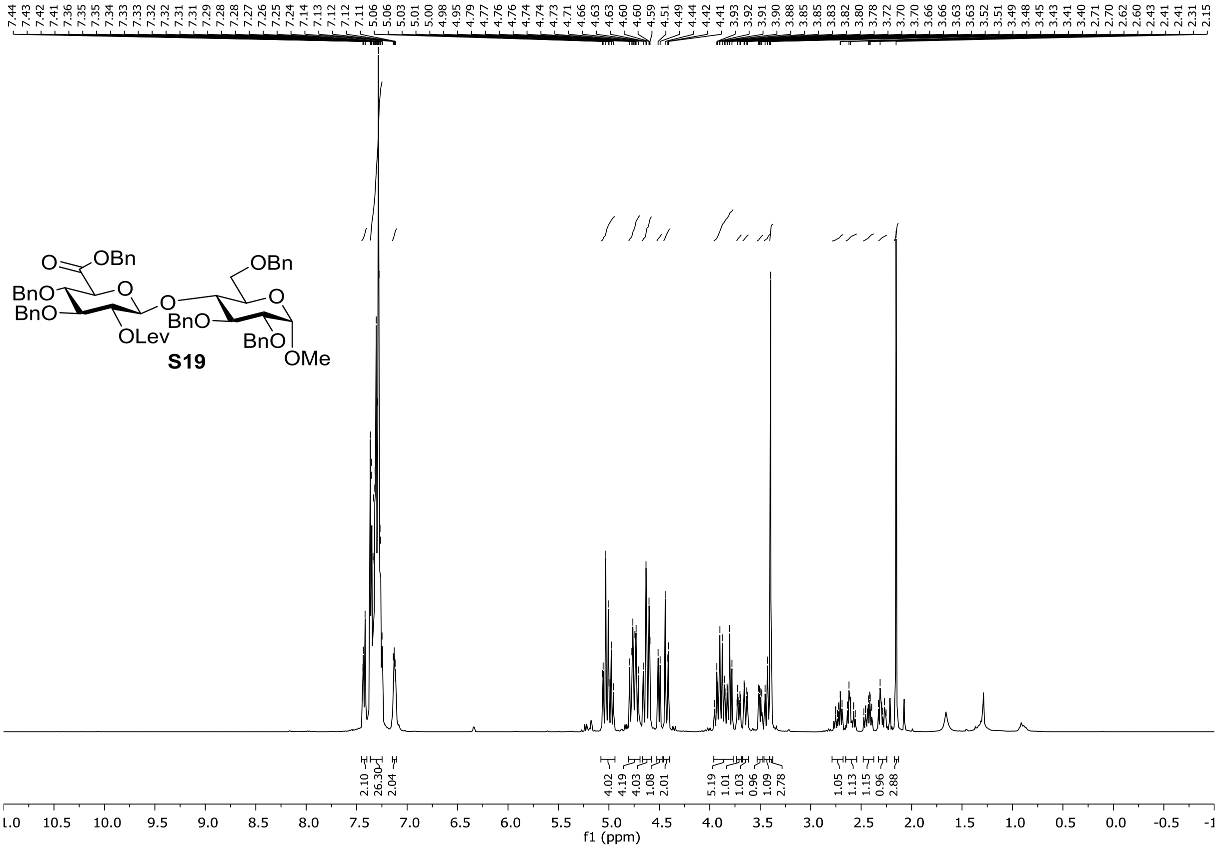

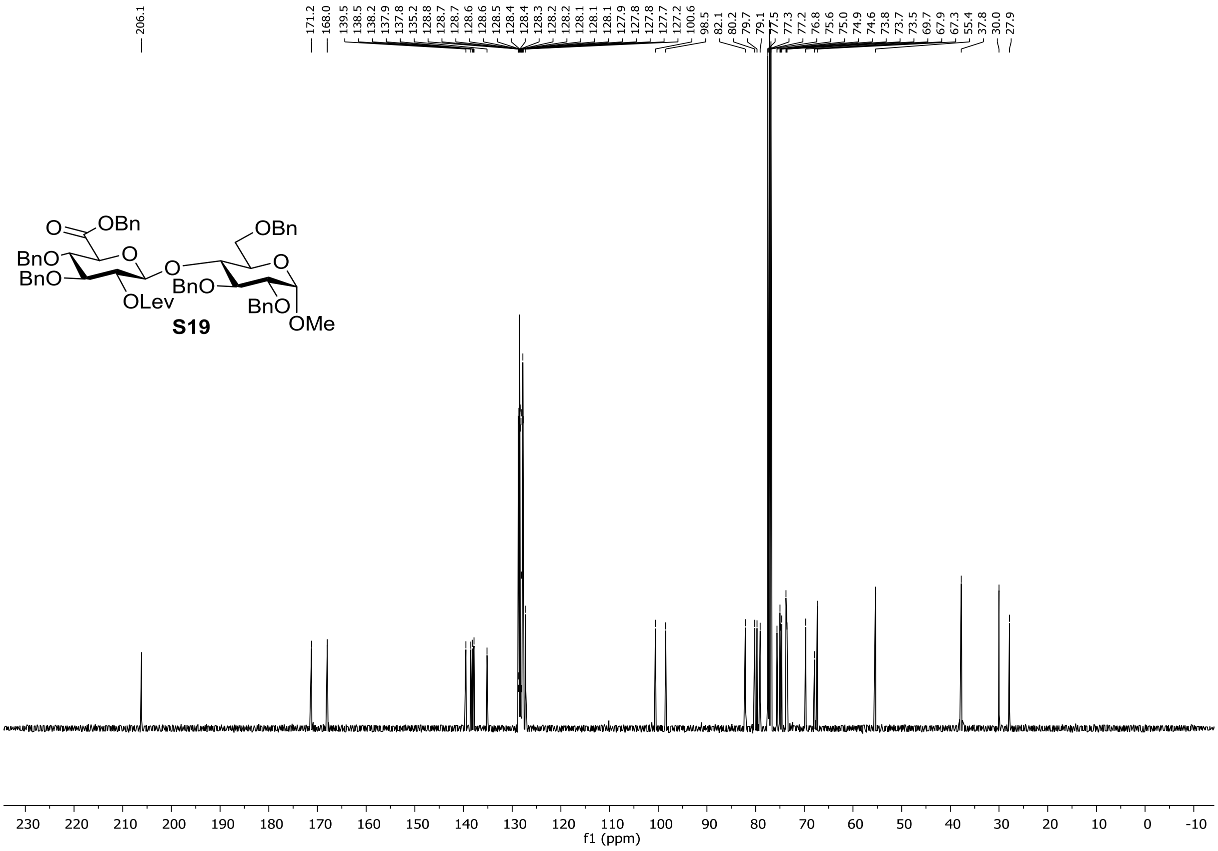

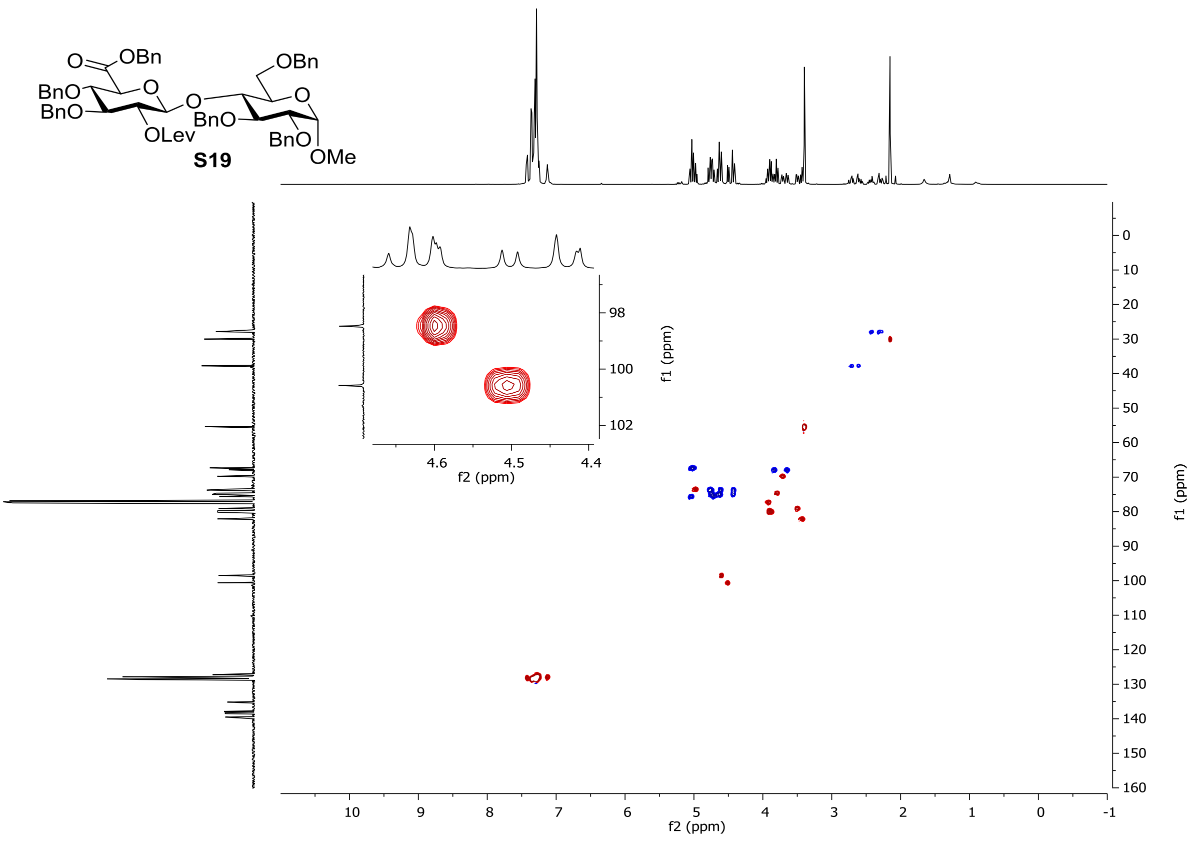

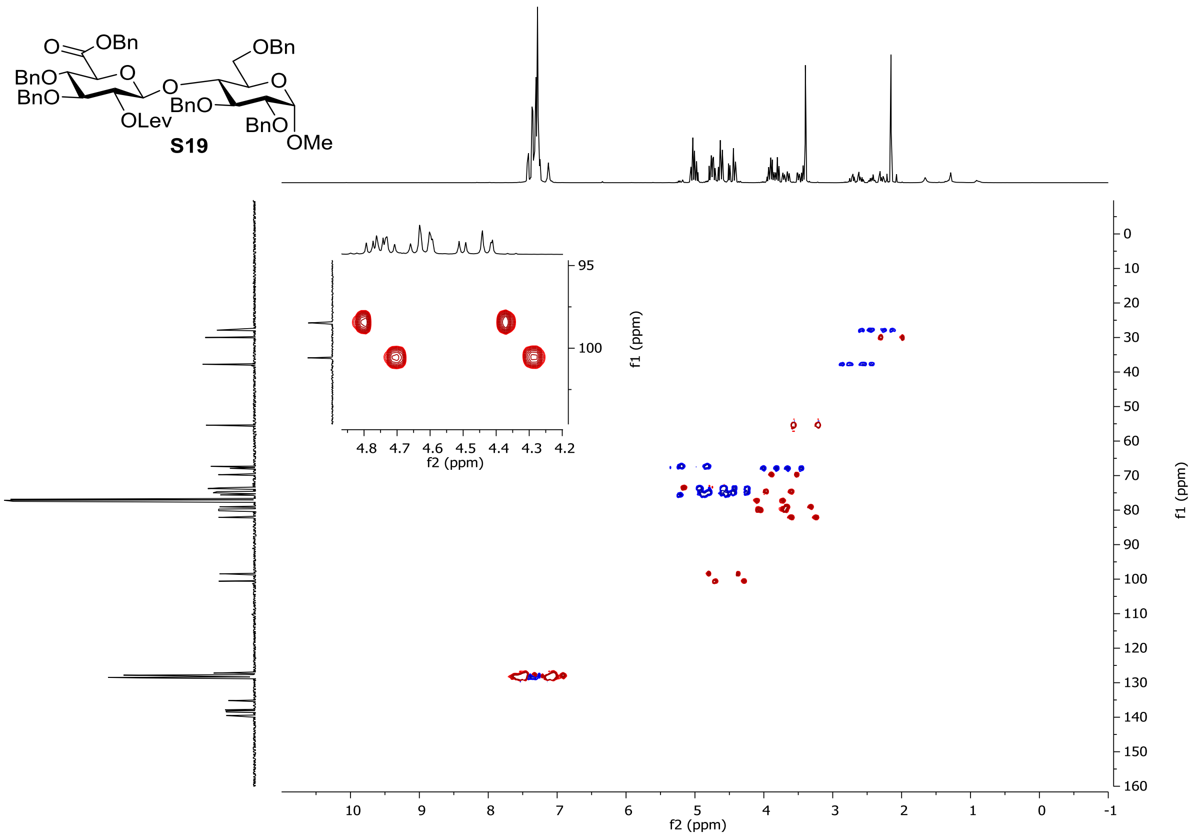

Methyl 2,3,4,6-tetra-*O*-benzyl-D-galactopyranosyl-(1→6)-2,3,4-tri-*O*-benzyl-α-D-glucopyranoside (S20)

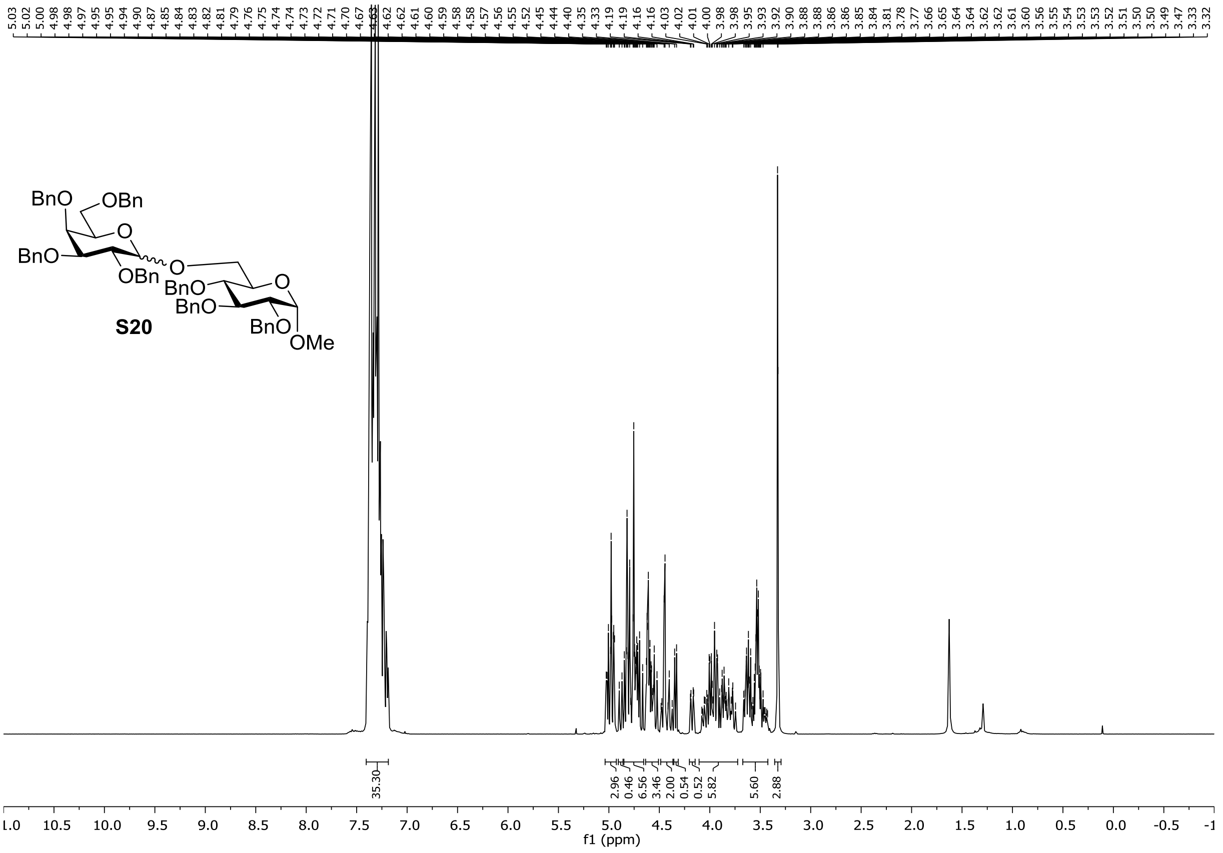

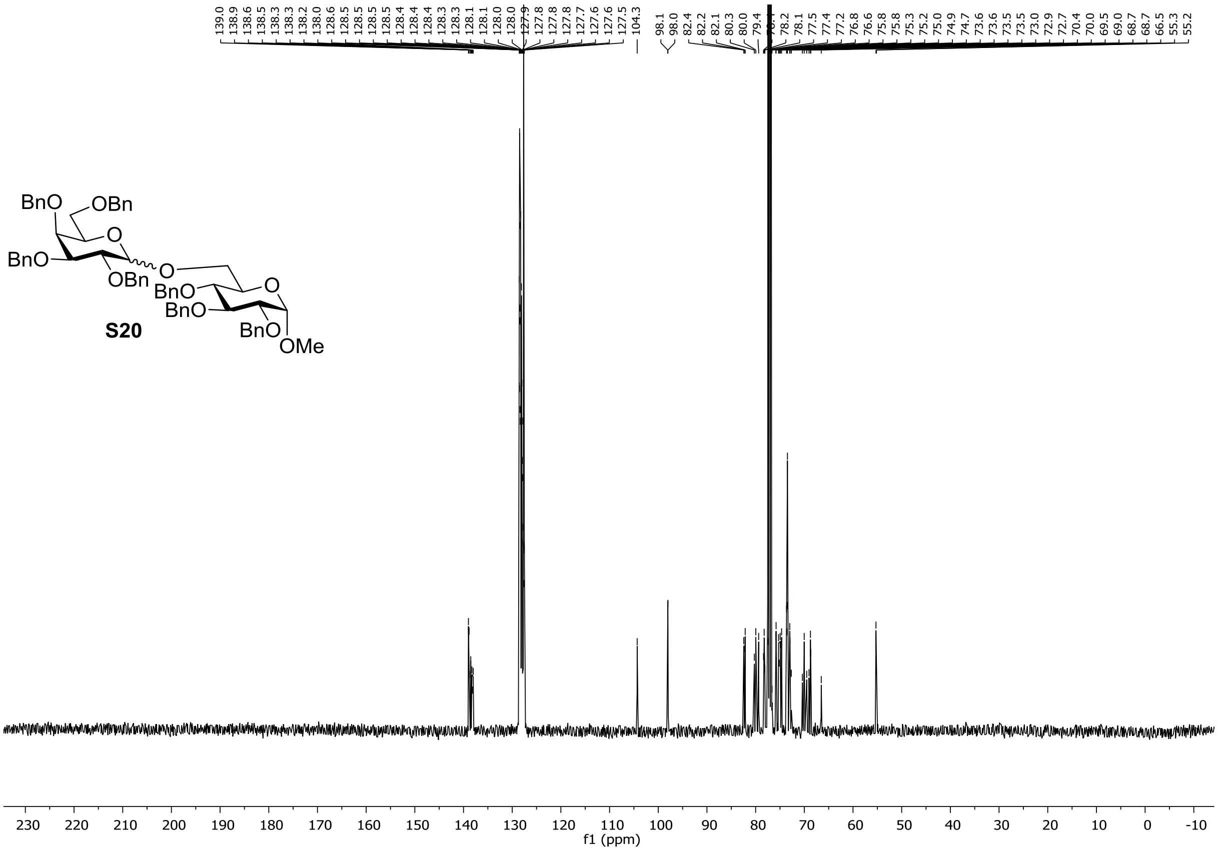
139.0
138.9
138.6
138.5
138.3
138.3
138.2
138.0
128.6
128.5
128.5
128.5
128.4
128.4
128.4
128.3
128.3
128.1
128.1
128.0
128.0
127.9
127.8
127.8
127.8
127.7
127.6
127.6
127.5
104.3
98.1
98.0
82.4
82.2
82.1
80.3
80.0
79.4
78.2
78.1
77.5
77.4
77.2
76.8
76.6
75.8
75.8
75.3
75.2
75.0
74.9
74.7
73.6
73.6
73.5
73.5
73.0
72.9
72.7
70.4
70.0
69.5
69.0
68.7
68.7
66.5
55.3
55.2
BnO
OBn
O
BnO
OBn
O
BnO
O
BnO
BnO
OMe
S20
f1 (ppm)

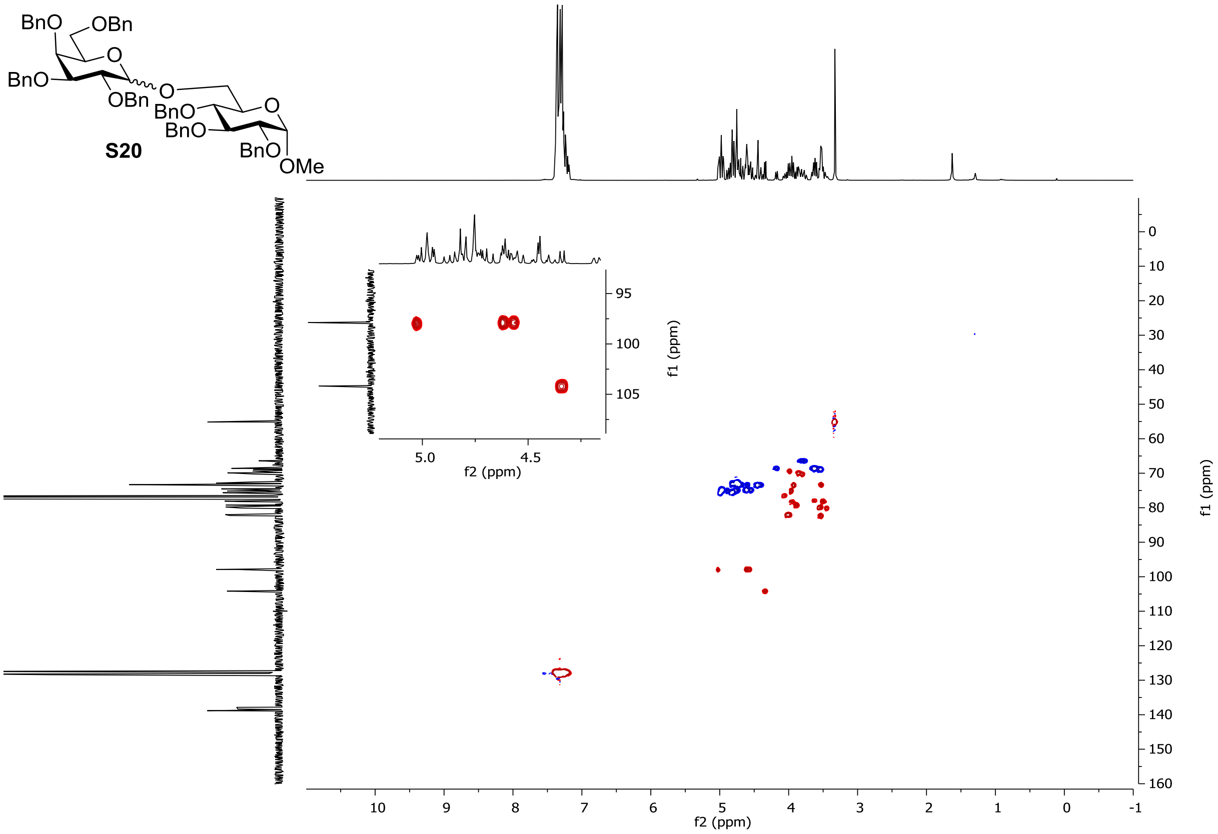
BnO
OBn
O
BnO
OBn
O
BnO
O
BnO
BnO
OMe
S20
f1 (ppm)
f2 (ppm)

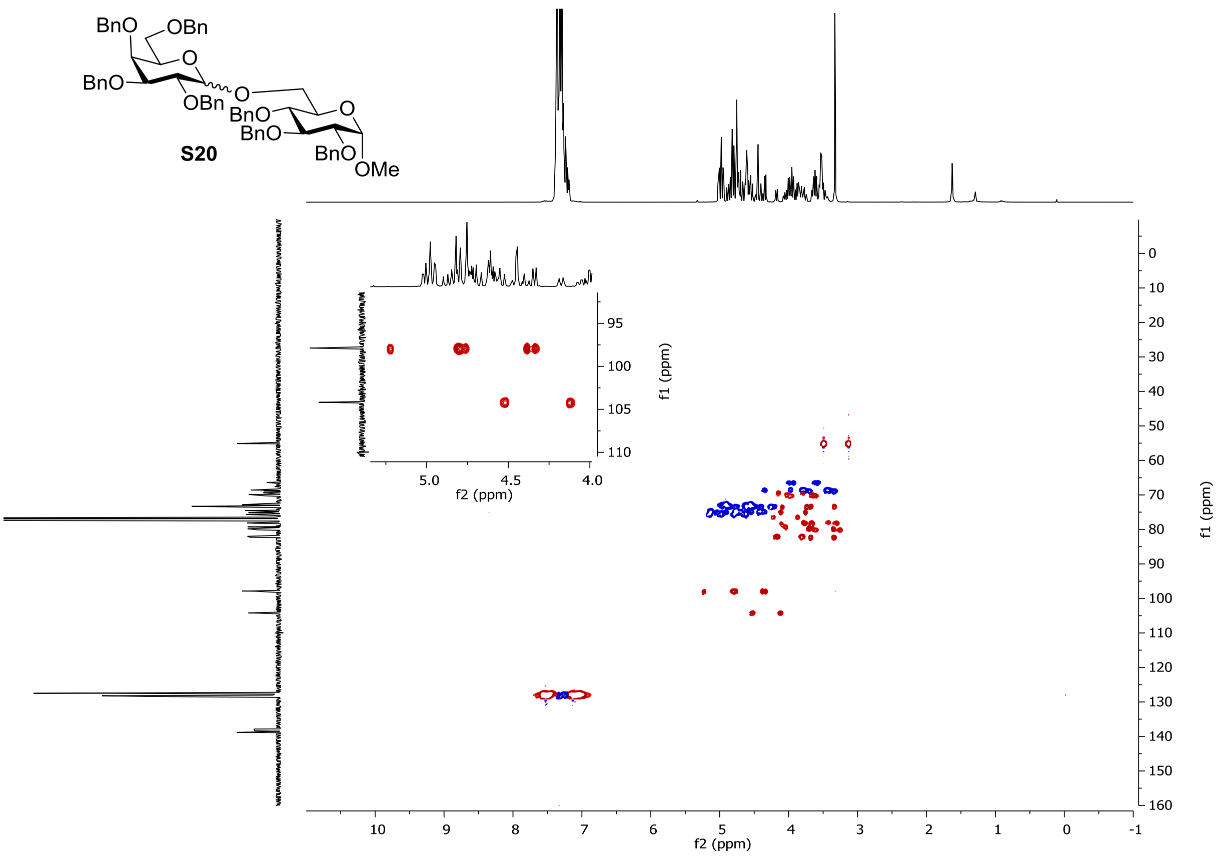

Methyl 4-*O*-benzoyl-2,3,6-tri-*O*-benzyl-D-galactopyranosyl-(1→6)-2,3,4-tri-*O*-benzyl-α-D-glucopyranoside (S21)

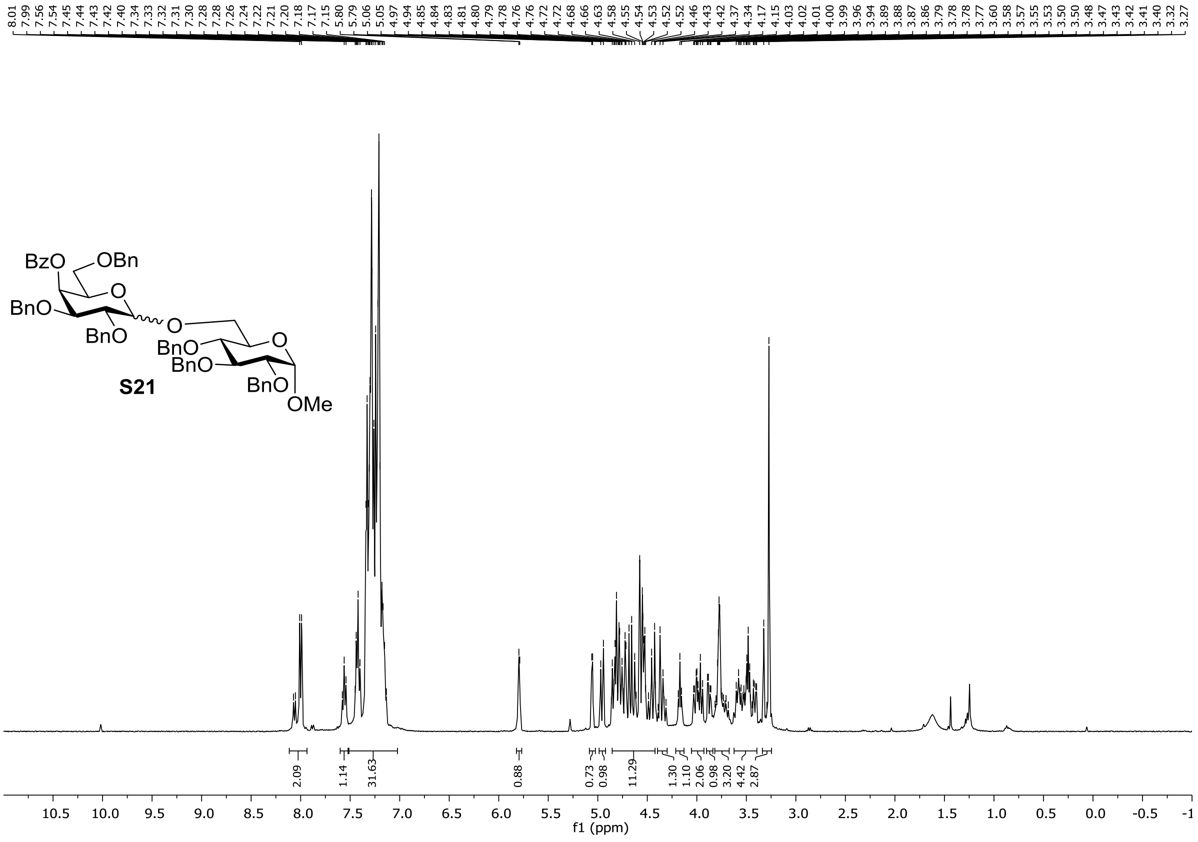

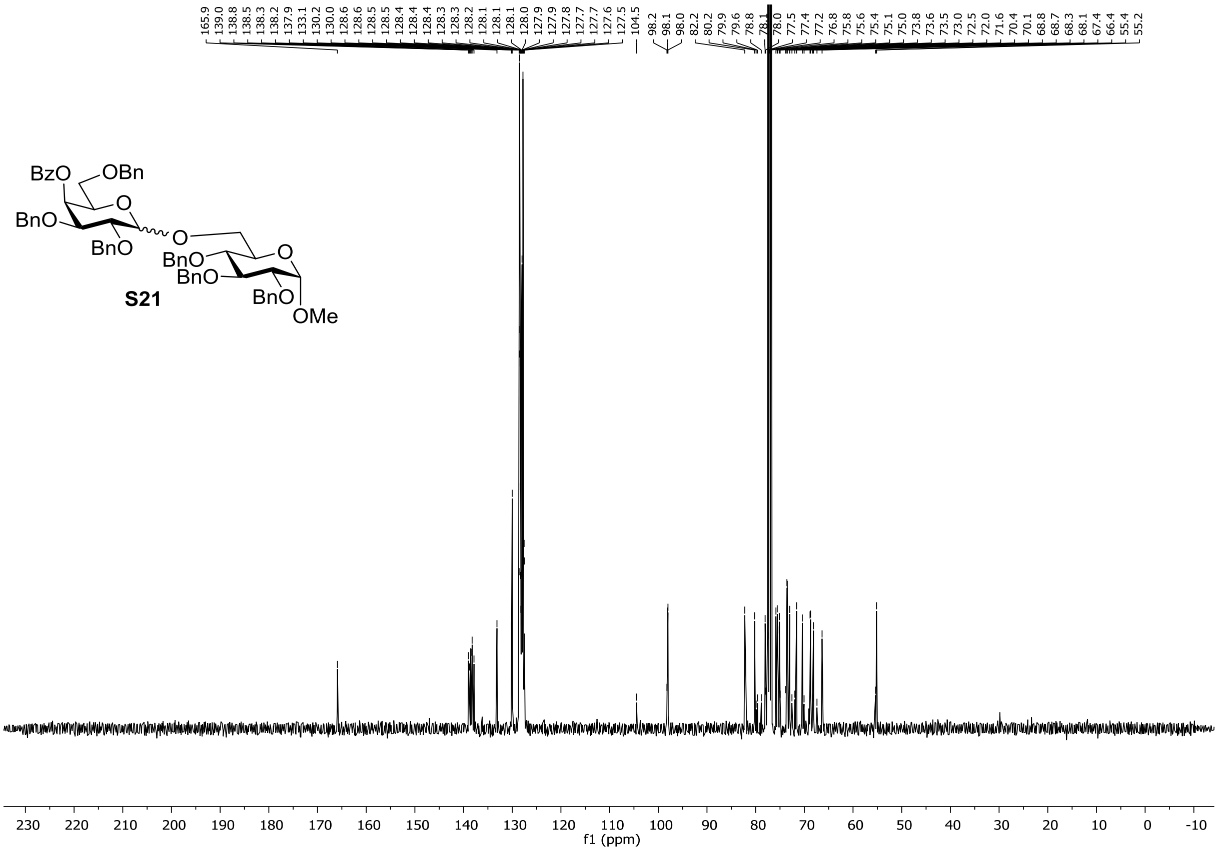

165.9
139.0
138.8
138.5
138.3
138.2
137.9
133.1
130.2
130.0
128.6
128.6
128.5
128.5
128.4
128.4
128.4
128.3
128.3
128.2
128.1
128.1
128.1
128.0
127.9
127.9
127.8
127.7
127.7
127.6
127.5
104.5
98.2
98.1
98.0
82.2
80.2
79.9
79.6
78.8
78.1
78.0
77.5
77.4
77.2
76.8
75.8
75.6
75.4
75.1
75.0
73.8
73.6
73.5
73.0
72.5
72.0
71.6
70.4
70.1
68.8
68.7
68.3
68.1
67.4
66.4
55.4
55.2
BzO
OBn
O
BnO
BnO
O
BnO
BnO
O
BnO
OMe
S21
f1 (ppm)

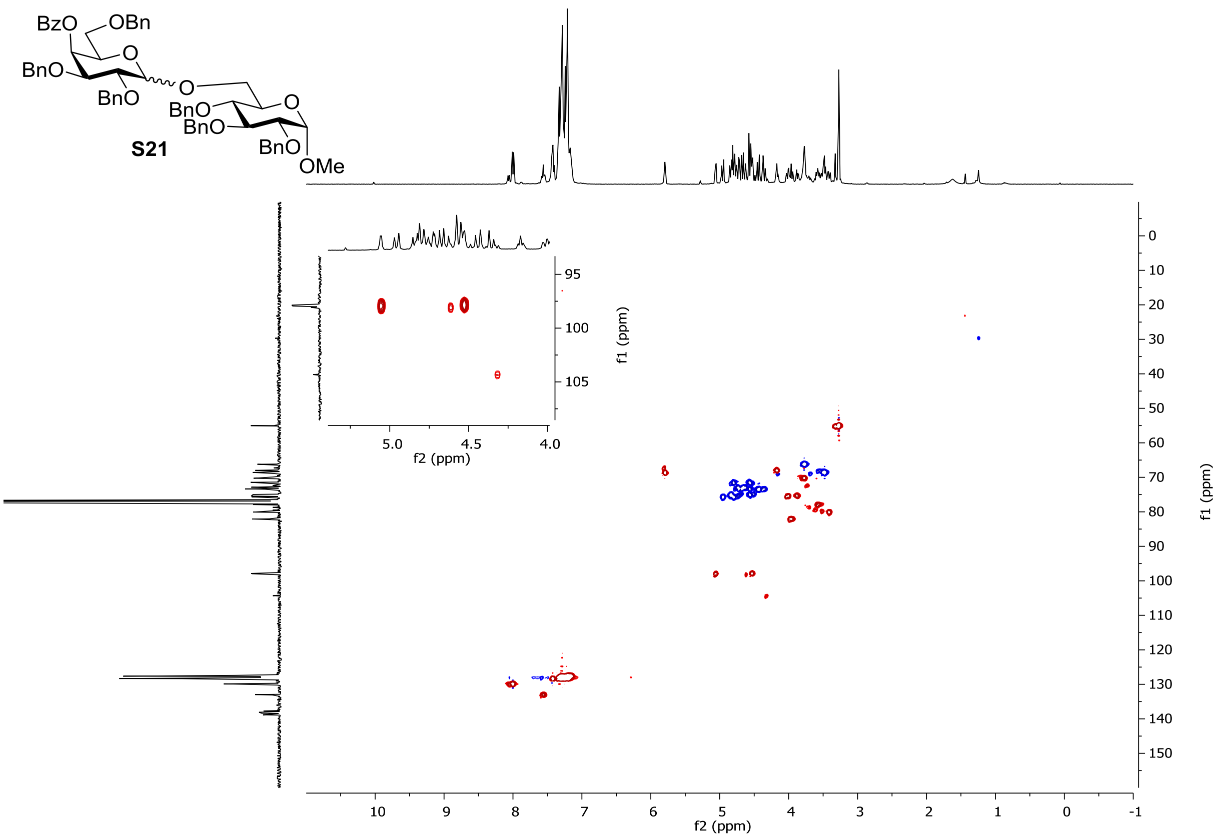

BzO
OBn
O
BnO
BnO
O
BnO
BnO
O
BnO
OMe
S21
f2 (ppm)
f1 (ppm)

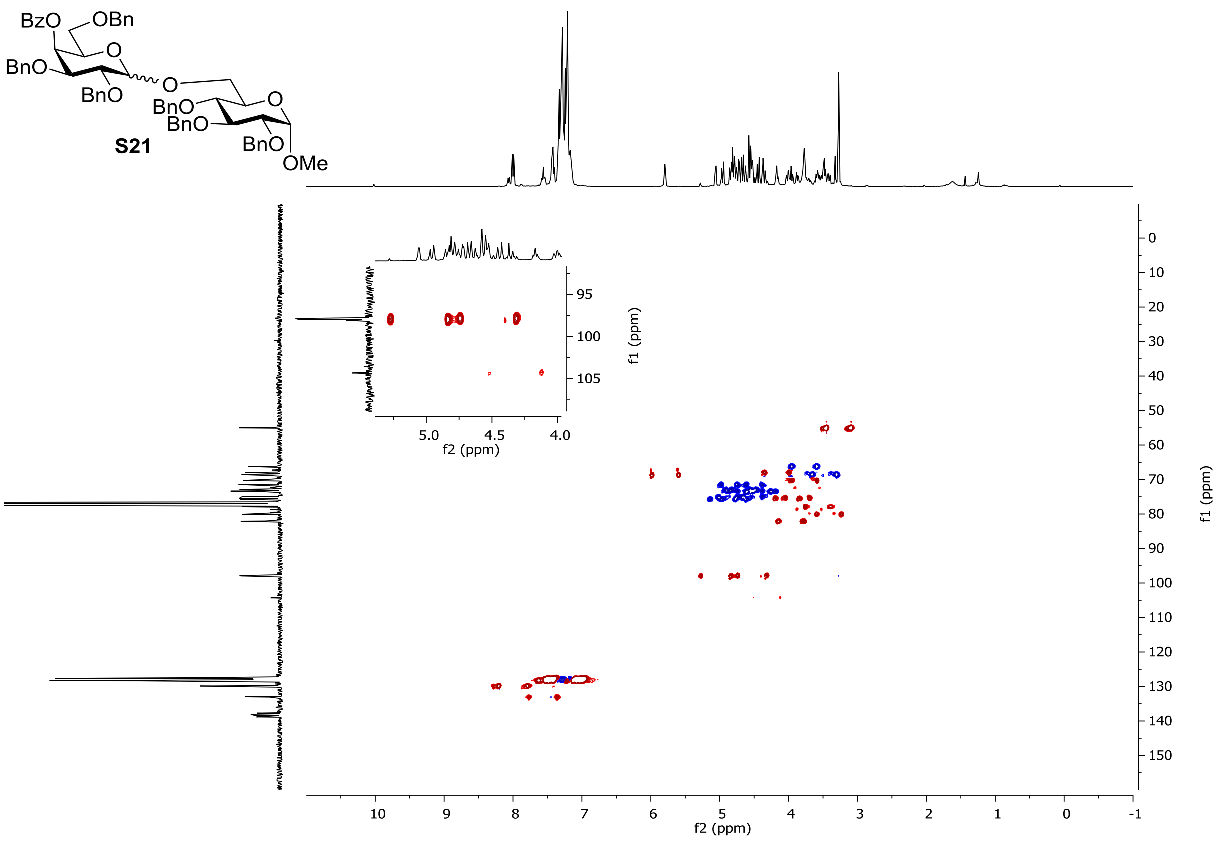

Methyl 4,6-di-*O*-benzoyl-2,3-di-*O*-benzyl-D-glucopyranosyl-(1→6)-2,3,4-tri-*O*-benzyl-α-D-glucopyranoside (S22)

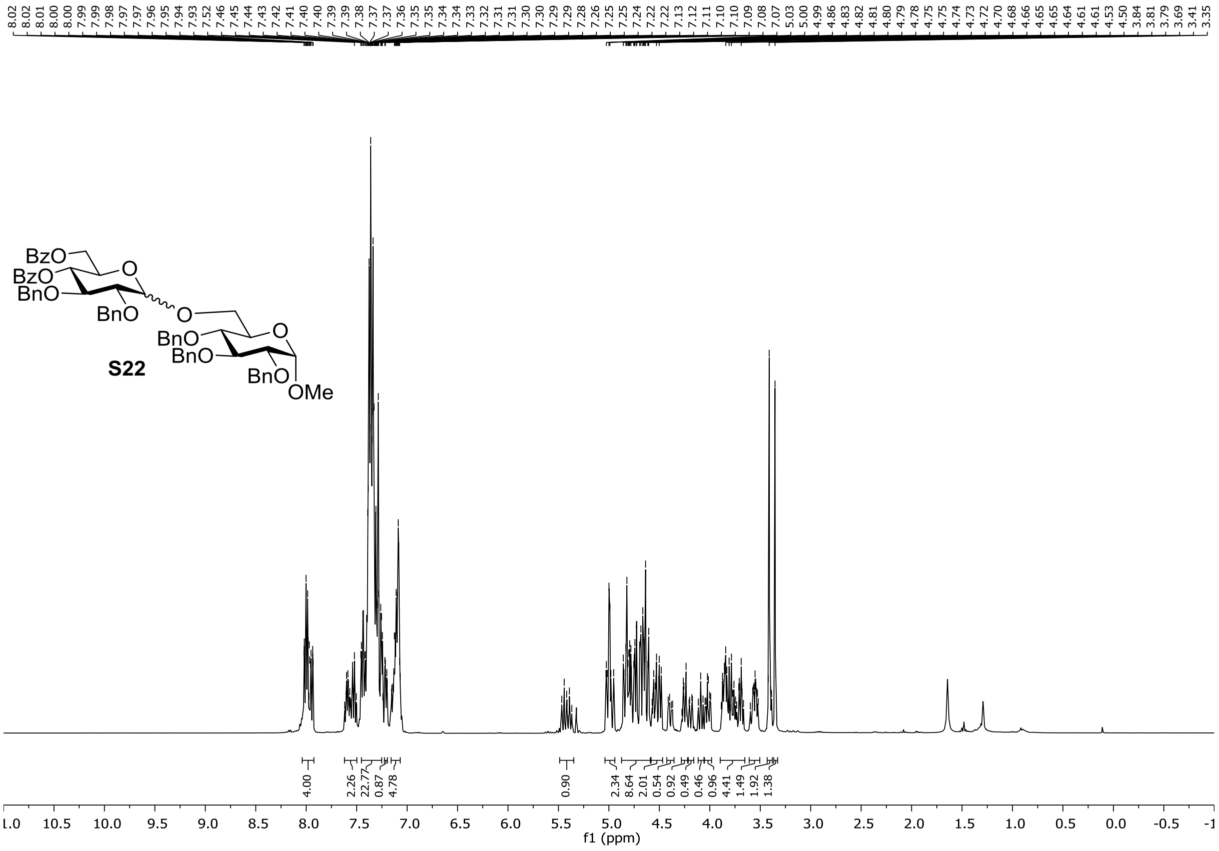

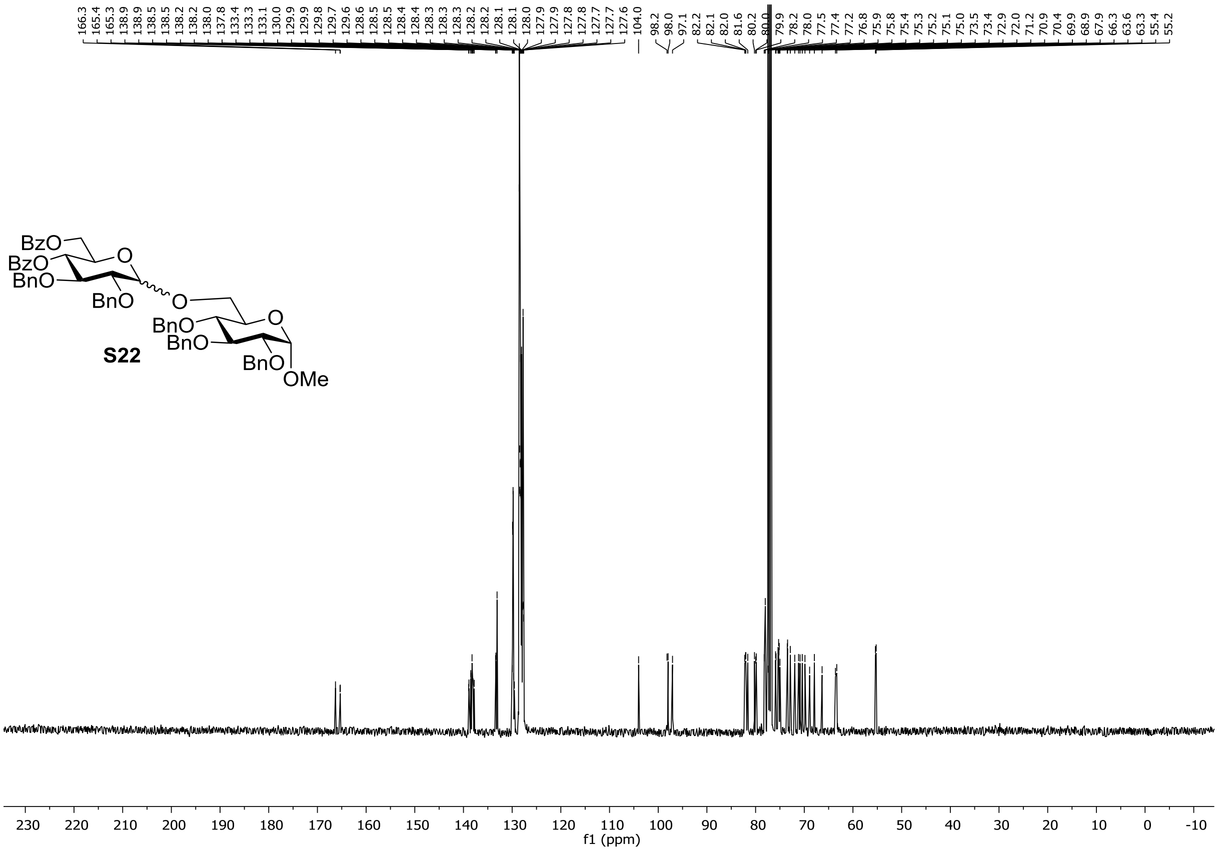
166.3
165.4
165.3
138.9
138.9
138.5
138.5
138.2
138.2
138.0
137.8
133.4
133.3
133.1
130.0
129.9
129.9
129.8
129.7
129.6
128.6
128.5
128.5
128.4
128.4
128.3
128.3
128.3
128.2
128.2
128.1
128.1
128.0
127.9
127.9
127.8
127.8
127.7
127.7
127.6
104.0
98.2
98.0
97.1
82.2
82.1
82.0
81.6
80.2
80.0
79.9
78.2
78.0
77.5
77.4
77.2
76.8
75.9
75.8
75.4
75.3
75.2
75.1
75.0
73.5
73.4
72.9
72.0
71.2
70.9
70.4
69.9
68.9
67.9
66.3
63.6
63.3
55.4
55.2
BzO
BzO
BnO
BnO
O
O
BnO
BnO
BnO
O
OMe
f1 (ppm)

S22

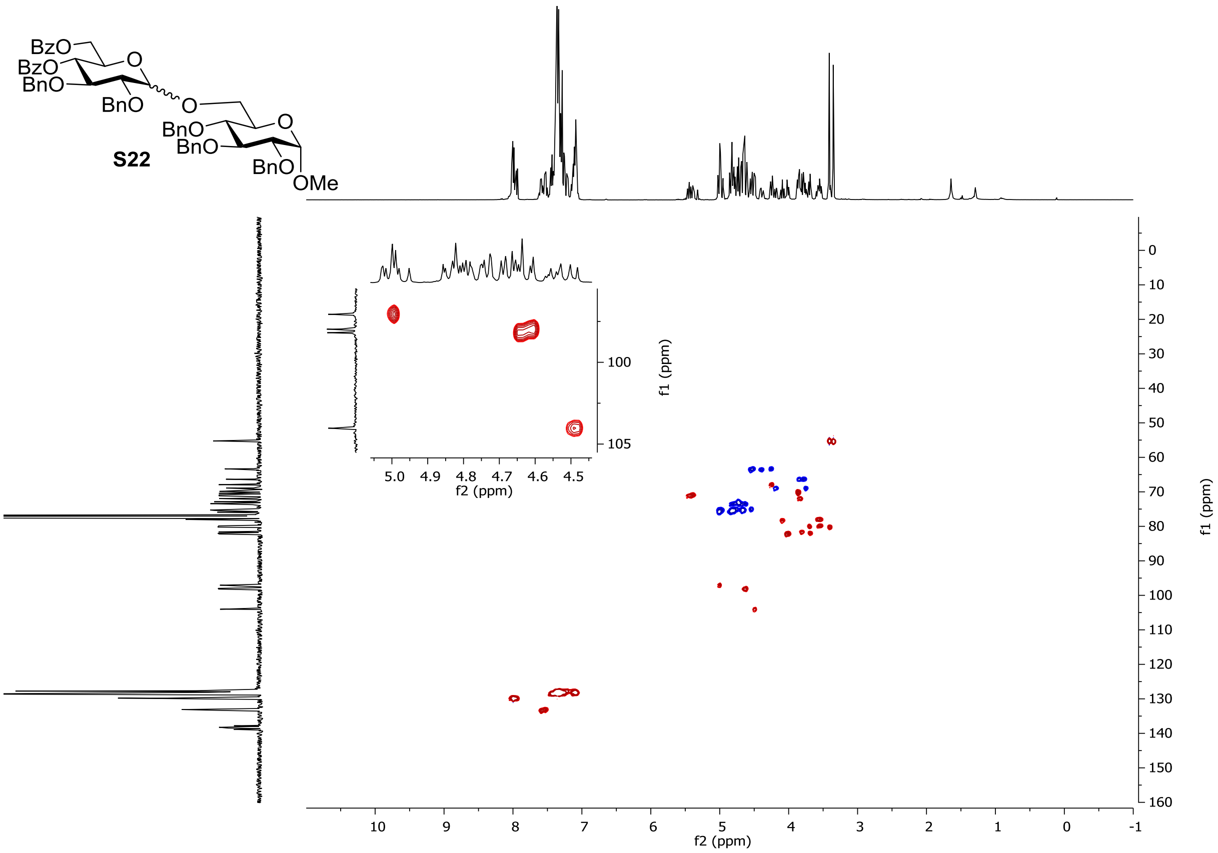
BzO
BzO
BnO
BnO
O
O
BnO
BnO
BnO
O
OMe
f2 (ppm)
f1 (ppm)

S22

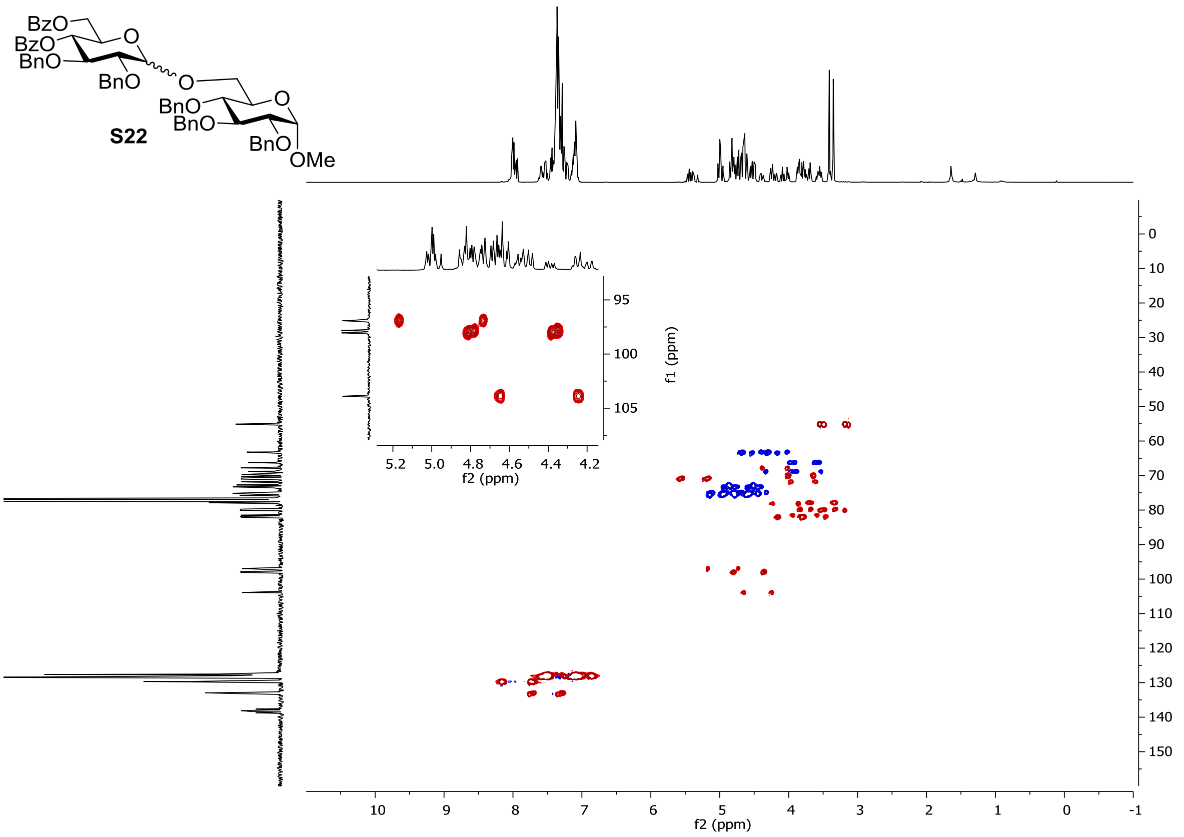

N-benzyloxycarbonyl-5-amino-pentanyl 2-O-benzoyl-3,4-di-O-benzyl-α-D-mannopyranosyl-(1→6)-2-O-benzoyl-3,4-di-O-benzyl-α-D-mannopyranoside (16)

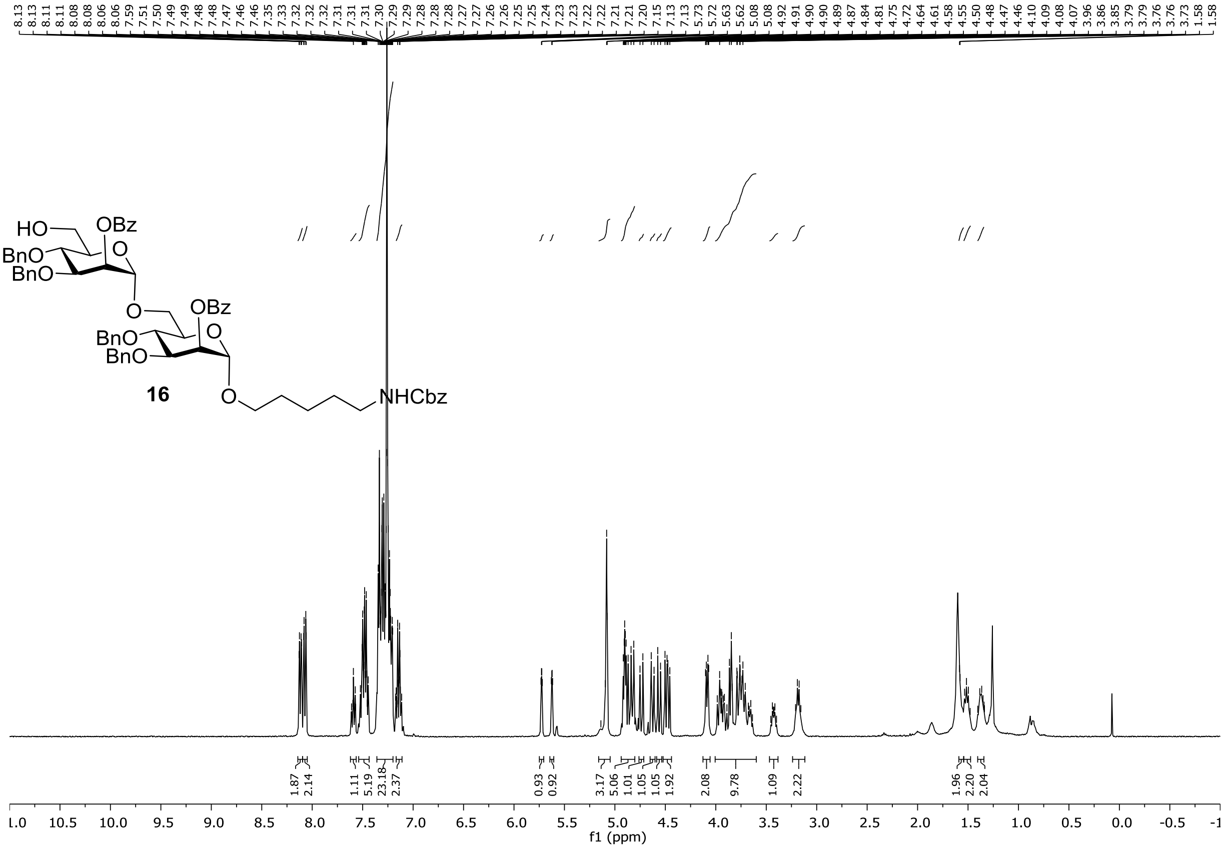

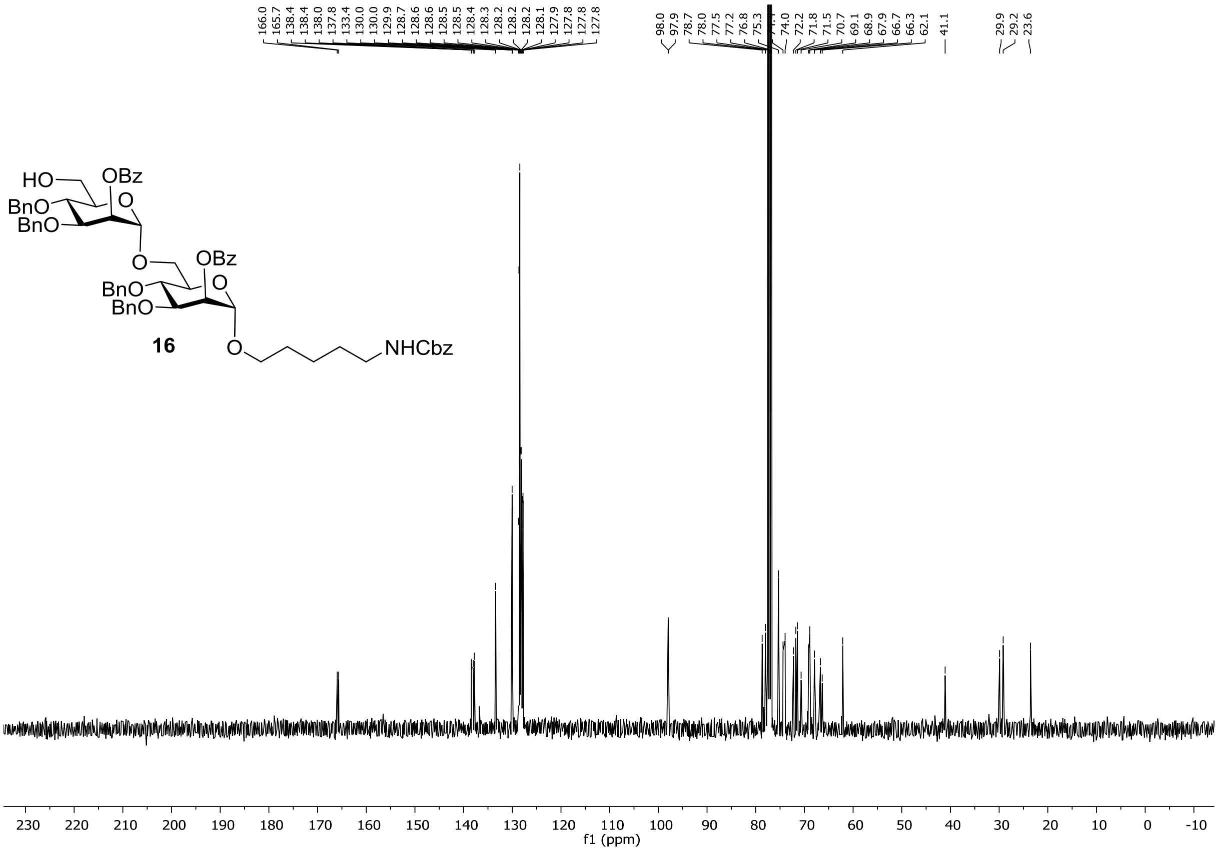

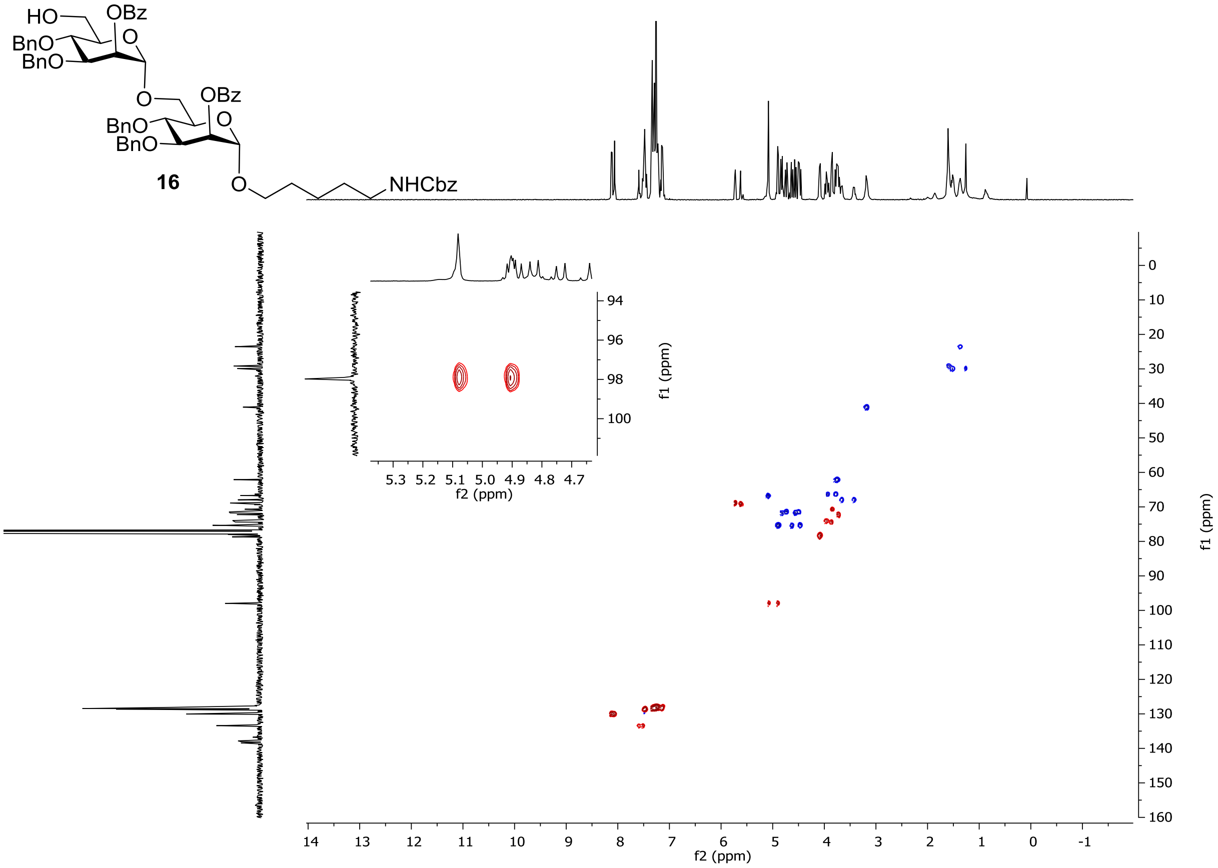

Phenyl 2-azido-2-deoxy-4-*O*-fluorenylmethoxycarbonyl-6-*O*-levulinoyl-3-*O*-(2-naphthalenylmethyl)-1-seleno-α-D-galactopyranoside (17)

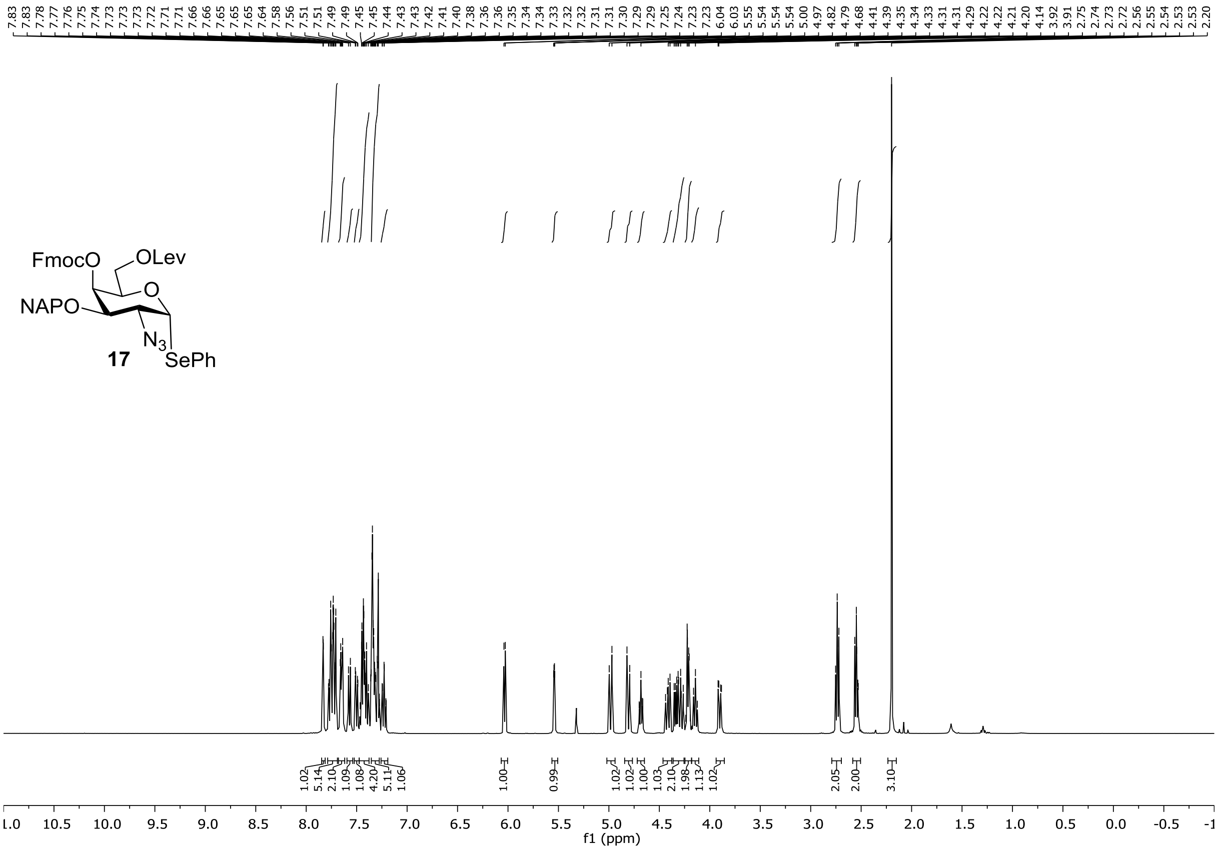
7.83 7.83 7.78 7.77 7.76 7.75 7.74 7.73 7.73 7.73 7.72 7.71 7.66 7.66 7.65 7.65 7.64 7.58 7.56 7.51 7.51 7.49 7.49 7.45 7.45 7.44 7.43 7.43 7.42 7.41 7.40 7.38 7.36 7.36 7.35 7.34 7.34 7.33 7.32 7.32 7.31 7.31 7.30 7.29 7.29 7.29 7.25 7.24 7.23 7.23 6.04 6.03 5.55 5.54 5.54 5.54 5.00 4.97 4.82 4.79 4.68 4.41 4.39 4.35 4.34 4.33 4.31 4.31 4.29 4.22 4.22 4.21 4.20 4.14 3.92 3.91 2.75 2.74 2.73 2.72 2.56 2.55 2.54 2.53 2.20
FmocO
OLev
NAPO
O
N3
SePh
17
1.02 5.14 2.10 1.09 1.08 4.20 5.11 1.06 1.00 0.99 1.02 1.02 1.00 1.03 2.10 1.98 1.13 1.02 2.05 2.00 3.10
f1 (ppm)

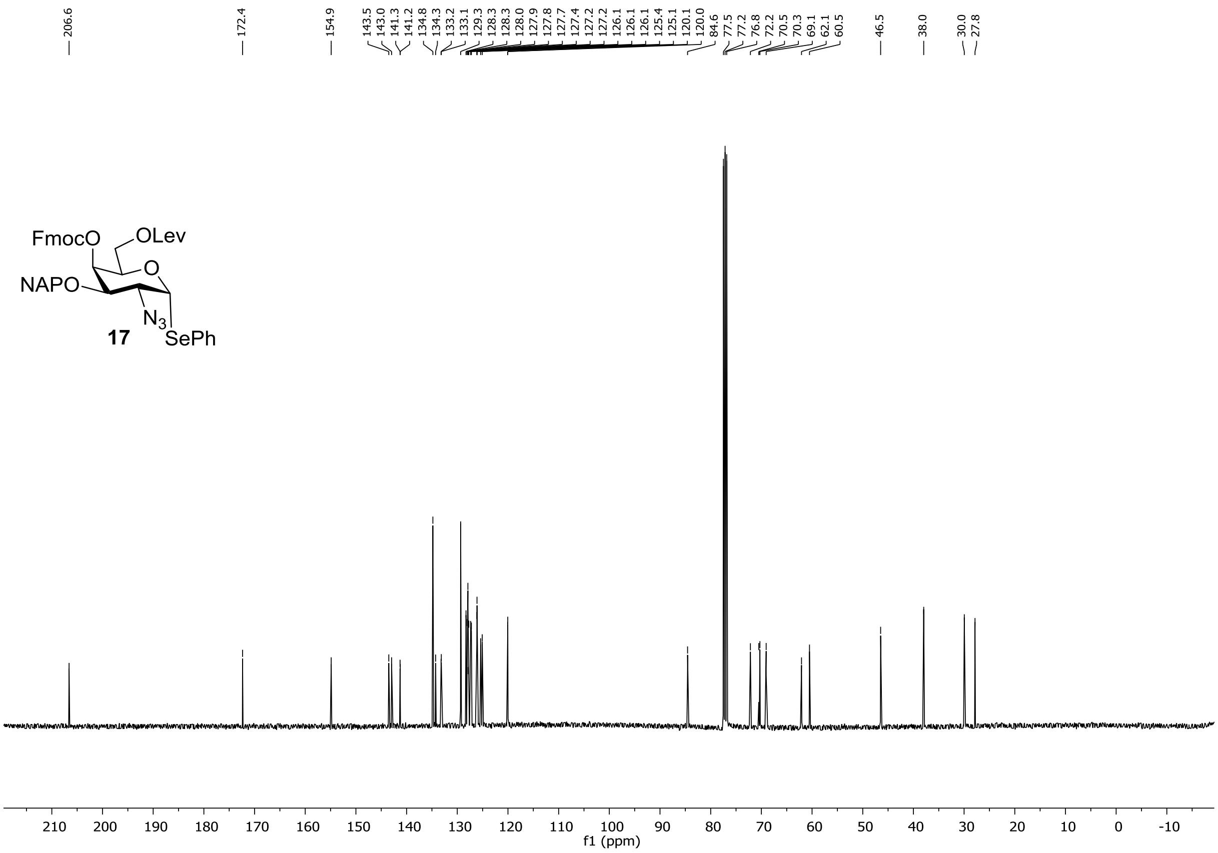
206.6 172.4 154.9 143.5 143.0 141.3 141.2 134.8 134.3 133.2 133.1 129.3 128.3 128.3 128.0 127.9 127.8 127.7 127.4 127.2 127.2 126.1 126.1 126.1 125.4 125.1 120.1 120.0 84.6 77.5 77.2 76.8 72.2 70.5 70.3 69.1 62.1 60.5 46.5 38.0 30.0 27.8
FmocO
OLev
NAPO
O
N3
SePh
17
f1 (ppm)

N-(Phenyl)trifluoroacetimidate 2-azido-2-deoxy-4-*O*-(9-fluorenylmethoxycarbonyl)-6-*O*-levulinoyl-3-*O*-(2-naphthalenylmethyl)-α-D-galactopyranoside (19)

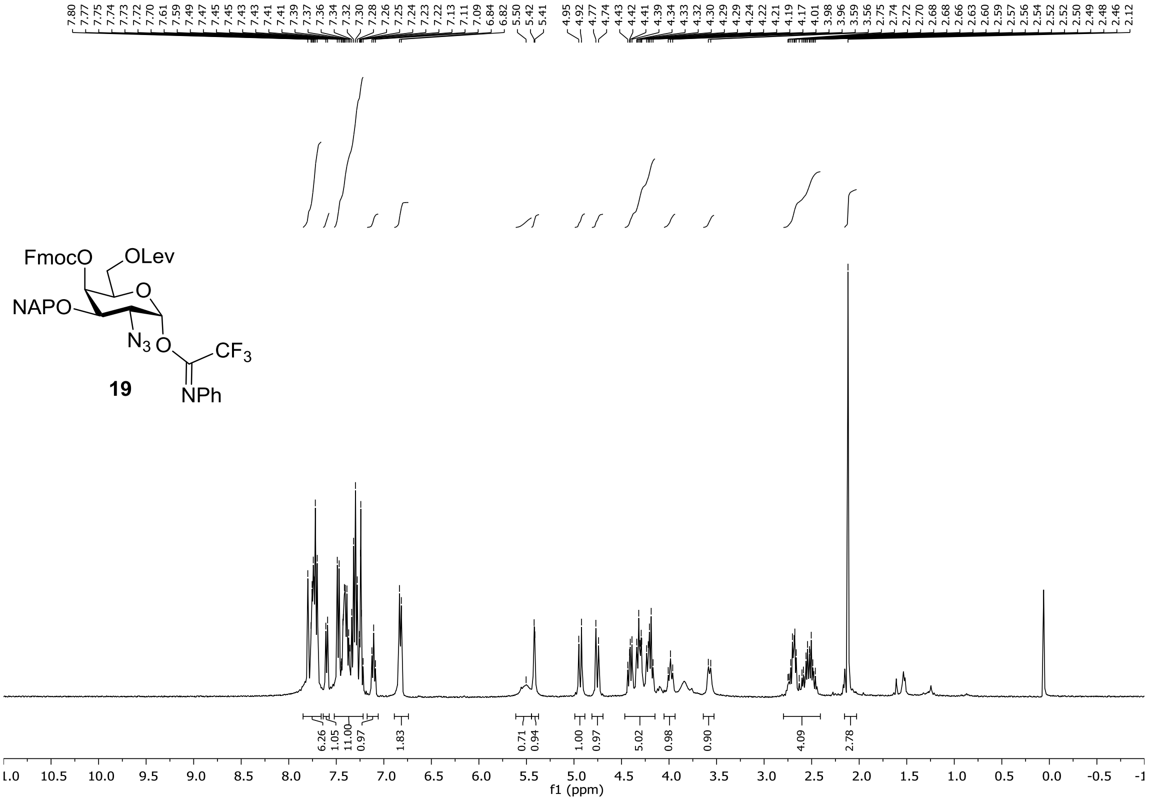

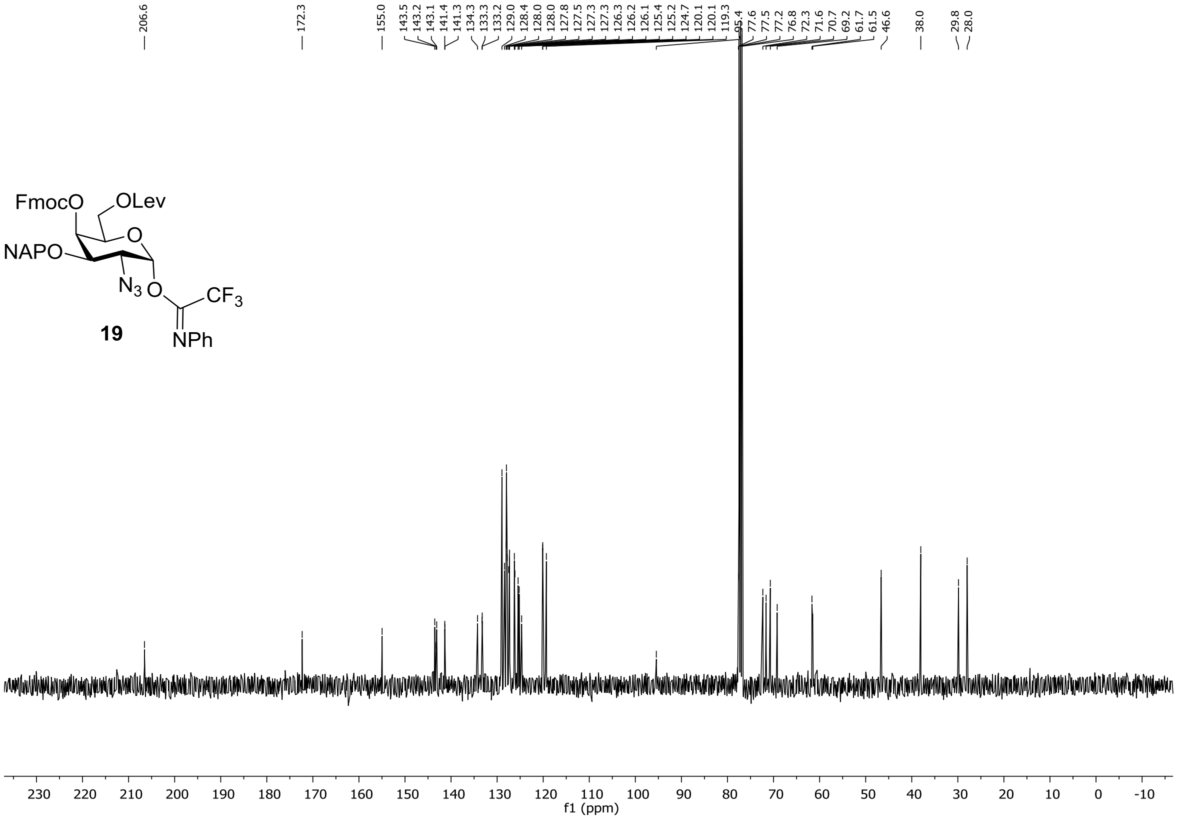

References

1. Hahm, H. S.; Liang, C.-F.; Lai, C.-H.; Fair, R. J.; Schuhmacher, F.; Seeberger, P. H. *J. Org. Chem.* **2016,** *81*, 5866-5877. http://dx.doi.org/10.1021/acs.joc.6b00554
2. Viuff, A. H.; Besenbacher, L. M.; Kamori, A.; Jensen, M. T.; Kilian, M.; Kato, A.; Jensen, H. H. *Org. Biomol. Chem.* **2015,** *13*, 9637-9658. http://dx.doi.org/10.1039/C5OB01281C
3. Lindberg, J.; Svensson, S. C. T.; Påhlsson, P.; Konradsson, P. *Tetrahedron* **2002,** *58*, 5109-5117. http://doi.org/10.1016/S0040-4020(02)00473-8
4. Toshiki, N.; Hiroaki, T.; Akito, S.; Takayuki, N.; Jun-ichi, Y. *Chem. Lett.* **2008,** *37*, 942-943. DOI: 10.1246/cl.2008.942
5. Martin, C. E.; Weishaupt, M. W.; Seeberger, P. H. *Chem. Commun.* **2011,** *47*, 10260-10262. http://dx.doi.org/10.1039/C1CC13614C
6. Hahm, H. S.; Broecker, F.; Kawasaki, F.; Mietzsch, M.; Heilbronn, R.; Fukuda, M.; Seeberger, P. H. *Chem* **2017,** *2*, 114-124. http://dx.doi.org/10.1016/j.chempr.2016.12.004
7. Kapoor, M.; Srinivas, H.; Kandiah, E.; Gemma, E.; Ellgaard, L.; Oscarson, S.; Helenius, A.; Surolia, A. *J. Biol. Chem.* **2003,** *278*, 6194-6200. DOI: 10.1074/jbc.M209132200
8. Dhenin, S. G. Y.; Moreau, V.; Nevers, M.-C.; Creminon, C.; Djedaini-Pilard, F. *Org. Biomol. Chem.* **2009,** *7*, 5184-5199. http://dx.doi.org/10.1039/B914534F
9. Lisboa, M. P.; Khan, N.; Martin, C. E.; Xu, F.-F.; Reppe, K.; Geissner, A.; Govindan, S.; Witzenrath, M.; Pereira, C. L.; Seeberger, P. H. *Proc. Natl. Acad. Sci. USA* **2017,** *Accepted*.
10. DeNinno, M. P.; Etienne, J. B.; Duplantier, K. C. *Tetrahedron Lett.* **1995,** *36*, 669-672. http://dx.doi.org/10.1016/0040-4039(94)02348-F
11. Eller, S.; Collot, M.; Yin, J.; Hahm, H. S.; Seeberger, P. H. *Angew. Chem., Int. Ed.* **2013,** *52*, 5858-5861. http://dx.doi.org/10.1002/anie.201210132
12. van der Es, D.; Groenia, N. A.; Laverde, D.; Overkleeft, H. S.; Huebner, J.; van der Marel, G. A.; Codée, J. D. C. *Bioorg. Med. Chem.* **2016,** *24*, 3893-3907. http://doi.org/10.1016/j.bmc.2016.03.019
